# Supplementary material for: Copper catalyzed late-stage C(sp3)-H functionalization of nitrogen heterocycles
Source: Nat Commun. 2021 Jul 15;12:4342. doi: 10.1038/s41467-021-24671-y (PMC8282657; doi:10.1038/s41467-021-24671-y)
Supplement: Supplementary file 1 — Supplementary Information [file 41467_2021_24671_MOESM1_ESM.pdf]

Supplementary Information

Copper catalyzed late-stage $C(sp^3)$-H functionalization of nitrogen heterocycles

Zhe Chang[1], Jialin Huang[1], Si Wang[1], Geshuyi Chen[2], Heng Zhao[1], Rui Wang[2]✉ and Depeng Zhao[1]✉

[1]*Guangdong Provincial Key Laboratory of Chiral Molecule and Drug Discovery, School of Pharmaceutical Sciences, Sun Yat-Sen University, Guangzhou 510006, China*

[2]*Key Laboratory of Preclinical Study for New Drugs of Gansu Province, School of Basic Medical Sciences, Lanzhou University, Lanzhou 730000, China*

✉Correspondence: wangrui@lzu.edu.cn

✉Correspondence: zhaodp5@mail.sysu.edu.cn

Supplementary Methods……………………………………………………………………………… S3

1. General remarks………………………………………………………………………….… S3

2. Optimization of reaction conditions of pyrrolidine and control experiments……… S4-S8

3. Mechanistic experiments ……………………………………………………………….. S9-S12

4. General procedure for synthesis of substrates and compound characterization …… S13-S26

5. General procedure for C(sp3)–H functionalization of N-heterocycles ……………... S27-S40

6. Synthetic applications of C(sp3)–H hydroxylated product of N-heterocycles ……… S41-S50

7. NMR Spectra ……………………………………………………………………………… S51-S149

Supplementary References ……………………………………………………………..…….. S150

Supplementary Methods

1. General remarks

Column chromatography was performed on silica gel (Silica-P flash silica gel from Silicycle, size 40-63 μm). TLC was performed on silica gel 60/ Kieselguhr F254. Mass spectra were recorded on a AEI-MS-902 mass spectrometer (EI+) or a LTQ Orbitrap XL (ESI+). ^{1}H, ^{13}C, ^{19}F NMR were recorded on a Varian AMX400 (400, 100.6 and 376 MHz, respectively) or a Varian Unity Plus Varian-500 (500, 125 and 471 MHz, respectively). Chemical shift values for ^{1}H and ^{13}C NMR are reported in ppm with the solvent resonance as the internal standard ($CHCl_3$: δ 7.26 ppm for ^{1}H, δ77.0 ppm for ^{13}C; DMSO: δ2.50 ppm for ^{1}H, δ39.52 ppm for ^{13}C; Acetone: δ2.05 ppm for ^{1}H, δ29.84 ppm for ^{13}C). ^{19}F NMR were reported in ppm using $FCCl_3$ (0 ppm) as an external standard. Data are reported as follows: chemical shifts, multiplicity (s = singlet, d = doublet, t = triplet, q = quartet, br = broad, m = multiplet), coupling constants (Hz), and integration. Melting points were determined on a Buchi B–545 melting point apparatus. *In situ* IR experiments were performed using a ReactIR 45m, purchased from Mettler Toledo. Enantiomeric ration were determined by high performance liquid chromatography (HPLC) analysis using a Thermo UltiMate 3000. All reactions were performed under anhydrous conditions and under N_2 atmosphere. All chemicals used were of analytical grade and were used as received without any further purification. All anhydrous solvents used in reactions were purchased in SureSeal bottles or dried over molecular sieves. Flash column chromatography was performed on Biotage Isolelera One with prepacked columns.

2. Optimization of reaction condition of pyrrolidine and control experiments

Example of procedure for optimization:

In a dried Schlenk tube, N- protected pyrrolidine (0.2 mmol), N-F-reagent (0.3 mmol), ligand (0.024 mmol, 12 mol%) and CuOAc (0.02 mmol, 10 mol%) were dissolved in CH_3CN (1.0 mL) under a N_2 atmosphere, then H_2O (0.6 mmol) was added. The reaction mixture was stirred at certain temperature for 18-24 h. The desired product was purified by column chromatography on silica gel with a gradient eluent of petroleum ether and ethyl acetate to give the desired product.

Supplementary Table 1. Evaluation of N-protecting groups.

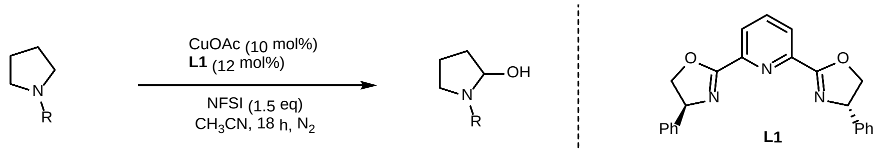

Entry	N-protecting group	yield[a]
**1**	***o*-Ns**	**26%**
2	*p*-Ns	19%
3	Ts	11%
4	Boc	N.R.
5	Cbz	N.R.
6	TFA	N.R.

Reaction conditions: N-heterocyclic substrates (0.2 mmol), H_2O (0.6 mmol), CuOAc (0.02 mmol), ligand (0.022 mmol), NFSI (0.3 mmol), CH_3CN (2.0 ml), 20 ^oC under N_2; [a]isolated yield.

Supplementary Table 2. Evaluation of copper sources and Ligands for C–H oxidation.

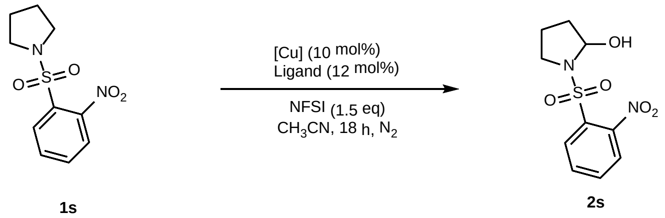

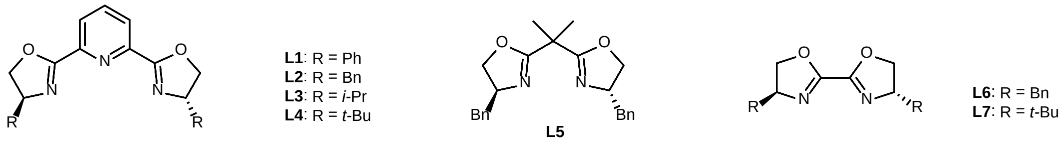

Entry	[Cu]	ligand	yield[a]
1	CuOAc	**L1**	26%
2	CuOAc	**L2**	29%
3	CuOAc	**L3**	N.R.
4	CuOAc	**L4**	17%
5	CuOAc	**L5**	31%
**6**	**CuOAc**	**L6**	**42%**
7	CuOAc	**L7**	N.R.
8	$Cu(MeCN)_4PF_6$	-	28%
9	CuOTf	**L6**	37%
10	CuBr	**L6**	15%
11	CuI	**L6**	N.R.

Reaction conditions: N-heterocyclic substrates (0.2 mmol), H_2O (0.6 mmol), CuOAc (0.02 mmol), ligand (0.022 mmol), NFSI (0.3 mmol), CH_3CN (2.0 ml), 20 ^oC under N_2; [a]isolated yield.

Supplementary Table 3. Evaluation of solvents.

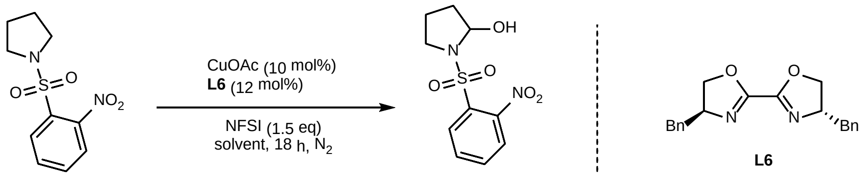

L6

1s 2s

Entry	solvent	yield[a]
1	DCM	16%
2	THF	N.R.
3	DMSO	N.R.
4	DMF	N.R.
5	EtOAc	21%
6	acetone	N.R.
7	chlorobenzene	N.R.
8	$CH_3CN:H_2O$ (95:5)	37%
9	$CH_3CN:H_2O$ (9:1)	N.R.

Reaction conditions: N-heterocyclic substrates (0.2 mmol), H_2O (0.6 mmol), CuOAc (0.02 mmol), ligand (0.022 mmol), NFSI (0.3 mmol), CH_3CN (2.0 ml), 20 ^oC under N_2; [a]isolated yield.

Supplementary Table 4. Evaluation of F-reagents.

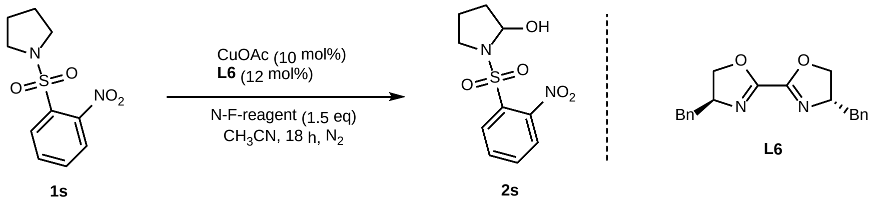

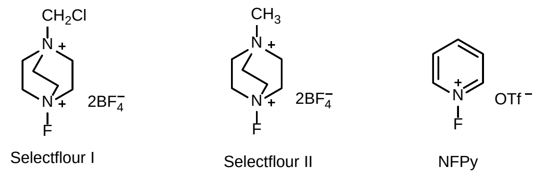

Entry	solvent	yield[a]
1	Selectflour I	61%
**2**	**Selectflour II**	**73%**
3	NFPy	15%

Reaction conditions: N-heterocyclic substrates (0.2 mmol), H_2O (0.6 mmol), CuOAc (0.02mmol), ligand (0.022 mmol), N-F-reagent (0.3 mmol), CH_3CN (2.0 ml), 20 ^{o}C under N_2; [a]isolated yield.

Supplementary Table 5. Control experiments.

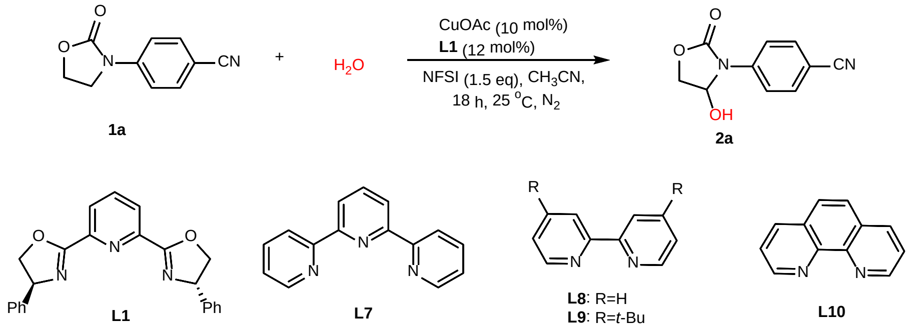

Entry	Condition	yield[a]
1	Without CuOAc	N.R.
2	Without Ligand	15%
3	Without NFSI	N.R.
4	Without N_2	71%
5	**L7** instead of **L1**	N.R.
6	**L8** instead of **L1**	N.R.
7	**L9** instead of **L1**	trace
8	**L10** instead of **L1**	N.R.
9	DDQ instead of NFSI	N.R.
10	TBHP instead of NFSI	N.R.

[a]isolated yield.

3. Mechanistic experiments

Kinetic isotope effects (KIE)

To determine whether the hydrogen atom transfer (HAT) step is involved in the product determining step, an intramolecular KIE study was performed.

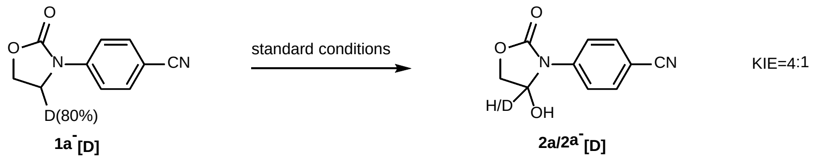

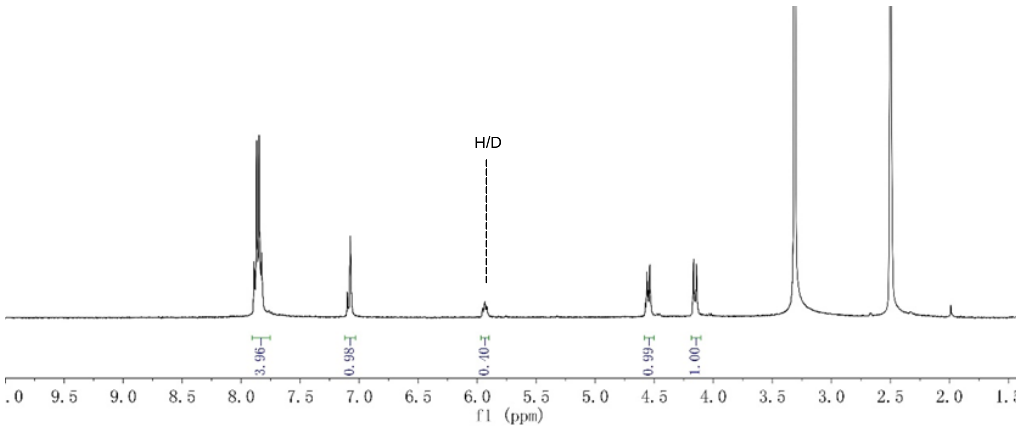

Supplementary Figure 1. ^{1}H NMR of the **2a/2a[D]**

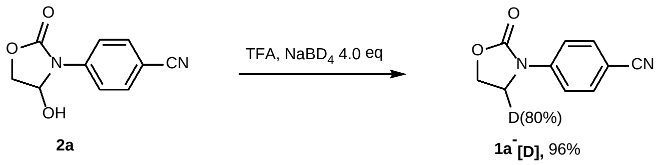

In a dried Schlenk tube, **2a** (1.0 mmol) was dissolved in 2.0 mL TFA under nitrogen and cooled to 0 ^{o}C. $NaBD_4$ (4.0 mmol) was added and the solution was stirred for 2 hours at rt. The reaction was added 10 mL 2M NaOH for quenching. The aqueous layer was extracted with DCM (2 x 30 mL), washed with brine (30 mL), dried over Na_2SO_4 and concentrated in vacuo. The desired product was purified by column chromatography on silica gel with a gradient eluent of petroleum ether and ethyl acetate to provide the desired product **1a-[D]**: white solid; yield 96%. ^{1}H NMR (400 MHz, $CDCl_3$) δ 7.72 – 7.63 (m, 4H), 4.56 - 4.52 (m, 2H), 4.14 – 4.03 (m, 1H). ^{13}C NMR (126 MHz, $CDCl_3$) δ 154.6, 142.06, 133.2, 118.6, 117.8, 106.9, 61.2, 44.7 - 44.2 (m).

Detection of fluorinated intermediate of the C-H functionalization via ^{1}H NMR (400 MHz) NMR

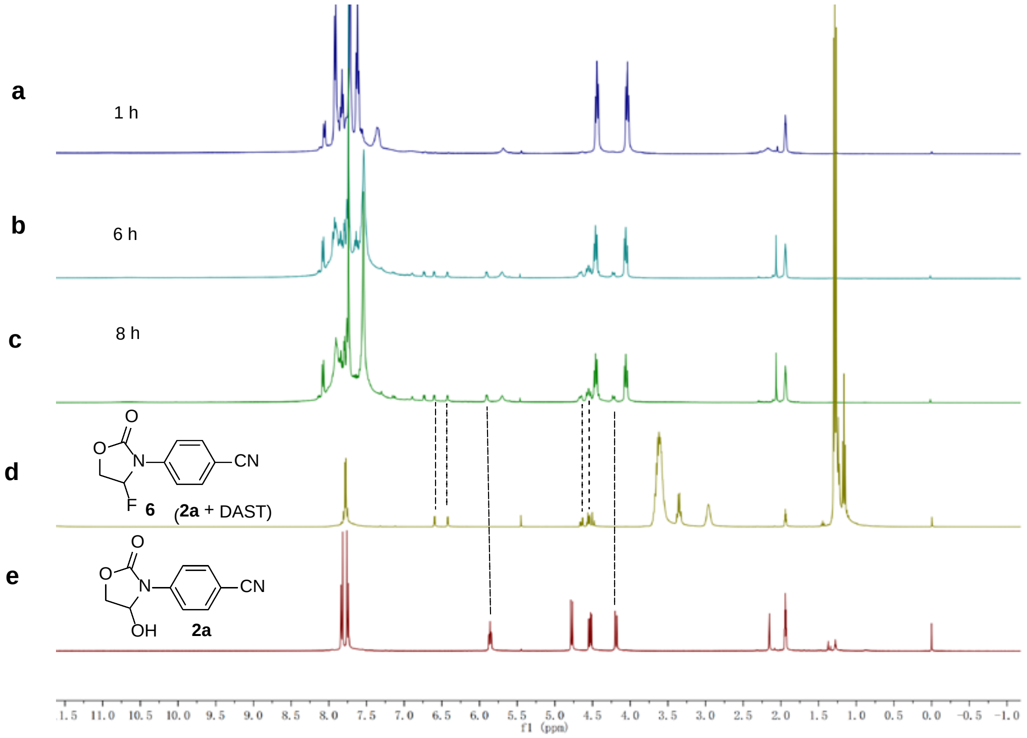

Supplementary Figure 2. Conditions: CD_3CN (0.5 mL), **1a** (0.1 mmol), CuOAc (0.01 mmol), **L1** (0.012 mmol), NFSI (0.15 mmol). **a**. ^{1}H NMR (400 MHz) NMR spectrum of the N-heterocyclic C-H functionalization reaction after 1 h. **b**. ^{1}H NMR (400 MHz) NMR spectrum of the reaction after 6 h. **c**. ^{1}H NMR (400 MHz) NMR spectrum of the reaction after 8 h. **d**. Fluorinated intermediate **6** prepared from **2a** and DAST: CD_3CN (0.5 mL), **2a** (0.1 mmol) and DAST (0.1 mmol). ^{19}F (376 MHz) NMR spectrum of the reaction after 1 h. **e**. ^{1}H NMR (400 MHz) of **2a.**

Detection of fluorinated intermediate of the C-H functionalization via *in situ* ReactIR

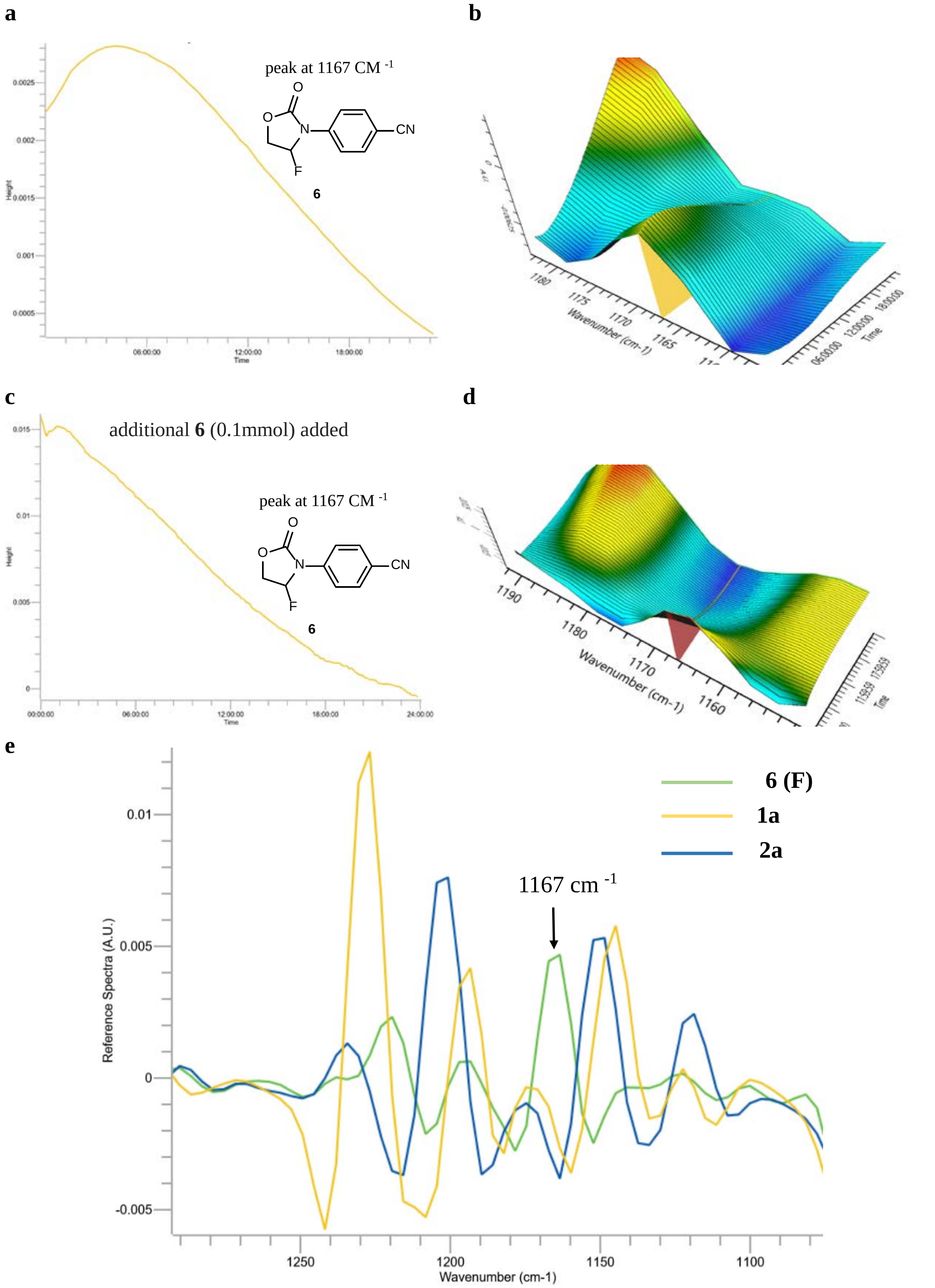

Supplementary Figure 3. Conditions **A**: CH_3CN (0.5 mL), **1a** (0.1 mmol), CuOAc (0.01 mmol), **L1** (0.012 mmol), NFSI (0.15 mmol). **a**. Monitoring trends of **6** (1167 cm $^{-1}$) by *in situ* ReactIR at conditions **A**. **b**. A segment of the ReactIR 3D spectra at conditions **A**. Conditions **B**: CH_3CN (0.5 mL), **1a** (0.1 mmol), **6** (0.1 mmol), CuOAc (0.01 mmol), **L1** (0.012 mmol), NFSI (0.15 mmol).**c**. Monitoring trends of **6** (1167 cm $^{-1}$) by *in situ* ReactIR with additional **6** added at conditions **B**. **d**. A segment of the ReactIR 3D spectra at conditions **B**. **e**. Comparison of **6**, **1a** and **2a** in *in situ* ReactIR**.**

Conversion of hemiaminal 2a to aminal 2aa in the presence of bis(phenyl)sulfonamide

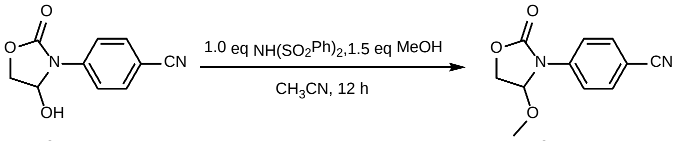

2a **2aa**, 95%

Supplementary Figure 4. Conditions: **2a** (0.1 mmol), $NH(SO_2Ph)_2$ (0.1 mmol) and MeOH (0.15 mmol) in CH_3CN (1.0 mL).

Exposure of the hemiaminal (2a and 2l) to the standard reaction conditions.

a

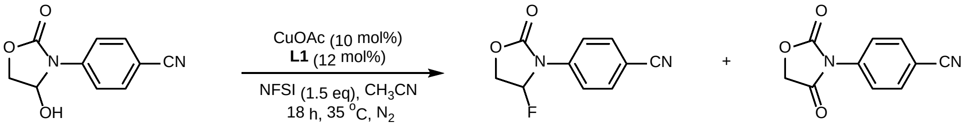

2a **6**, 0% 0%

b

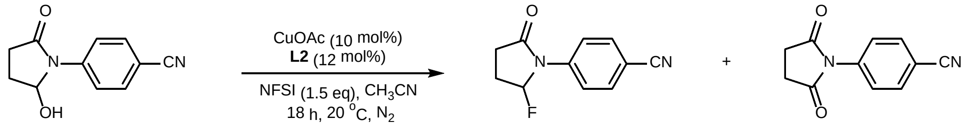

2l 0% 8%

Supplementary Figure 5. a. Conditions: **2a** (0.2 mmol), H_2O (0.6 mmol), CuOAc (0.02 mmol), **L1** (0.024 mmol), NFSI (0.3 mmol), CH_3CN (2.0 mL), 18 h, 35 °C. **b**. Conditions: **2l** (0.2 mmol), H_2O (0.6 mmol), CuOAc (0.02 mmol), **L2** (0.024 mmol), NFSI (0.3 mmol), CH_3CN (2.0 mL), 18 h, 20 °C.

4. General procedure for synthesis of substrates and compound characterization.

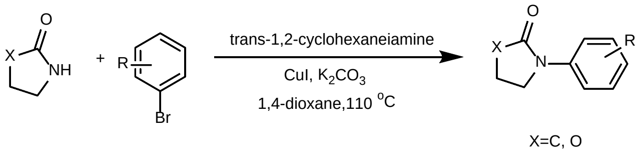

2-Oxazolidone[1] (2.6 g, 30 mmol, 1.0 eq), 4-bromobenzonitrile (1.2 eq), CuI (0.1 eq) and K_2CO_3 (2.0 eq) were added to 100 mL 2 neck thick-walled pressure bottle. The whole system was evacuated and filled with N_2 three times. Under N_2, 45 mL of dry 1,4-dioxane and trans-1,2-cyclohexaneiamine (0.1 eq) were added at room temperature. The reaction mixture was heated at 110 °C for 40 hours and then it was allowed to cool to room temperature. The reaction was quenched by adding water and extracted with DCM three times. The organic layer was washed with brine, dried over anhydrous Na_2SO_4 and concentrated under reduced pressure. The residue was purified by column chromatography to obtain the desired product.

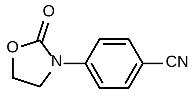

1a: white solid; yield 59%, m.p. = 143-145 °C.

^{1}H NMR (400 MHz, $CDCl_3$) δ 7.78 – 7.61 (m, 4H), 4.54 (dd, *J* = 8.8, 7.2 Hz, 2H), 4.09 (dd, *J* = 8.8, 7.2 Hz, 2H).

^{13}C NMR (101 MHz, $CDCl_3$) δ 154.6, 142.0, 133.1, 118.6, 117.8, 106.9, 61.3, 44.7.

HRMS (ESI+, *m/z*) calculated for $C_{10}H_8N_2O_2$ $[M + H]^+$: 189.0659; found: 189.0660;.

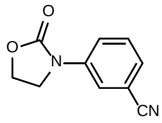

1b: white solid; yield 85%, m.p. = 100-102 °C.

^{1}H NMR (400 MHz, $CDCl_3$) δ 7.89 – 7.77 (m, 2H), 7.47 (t, *J* = 7.9 Hz, 1H), 7.40 (d, *J* = 7.7 Hz, 1H), 4.58 – 4.48(m, 2H), 4.12 – 4.02(m, 2H).

^{13}C NMR (126 MHz, $CDCl_3$) δ 154.8, 139.0, 129.9, 127.2, 121.9, 120.8, 118.3, 113.0, 61.4, 44.7.

HRMS (ESI+, *m/z*) calculated for $C_{10}H_8N_2O_2$ $[M + H]^+$: 189.0659; found: 189.0660;.

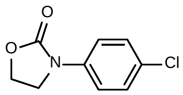

1c: white solid; yield 65%, m.p. = 115-116 °C.

^{1}H NMR (400 MHz, $CDCl_3$) δ 7.54 – 7.45 (m, 2H), 7.37 – 7.29 (m, 2H), 4.48 (dd, *J* = 8.8, 7.2 Hz, 2H), 4.03 (dd, *J* = 8.8, 7.2 Hz, 2H).

^{13}C NMR (126 MHz, $CDCl_3$) δ 155.0, 136.8, 129.2, 129.0, 119.3, 61.2, 45.1.

HRMS (ESI+, *m/z*) calculated for $C_9H_8ClNO_2$ $[M + H]^+$: 198.0316; found: 198.0318;.

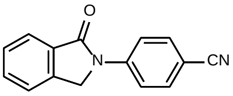

1i: white solid; yield 60%, m.p. = 190-191 °C.

^{1}H NMR (400 MHz, $CDCl_3$) δ 8.08 – 8.01 (m, 2H), 7.96 – 7.90 (m, 1H), 7.73 – 7.67 (m, 2H), 7.67 – 7.62 (m, 1H), 7.58 – 7.50 (m, 2H), 4.87 (s, 2H).

^{13}C NMR (126 MHz, $CDCl_3$) δ 167.9, 143.3, 139.7, 133.3, 132.9, 132.4, 128.8, 124.5, 122.8, 118.8, 118.6, 107.0, 50.3.

HRMS (ESI+, *m/z*) calculated for $C_{15}H_{10}N_2O$ $[M + H]^+$: 235.0866; found: 235.0864;.

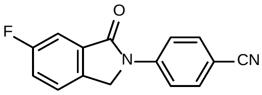

1j: white solid; yield 80%, m.p. = 233-235 °C.

^{1}H NMR (400 MHz, Chloroform-*d*) δ 8.08 – 7.99 (m, 2H), 7.78 – 7.68 (m, 2H), 7.60 (dd, *J* = 7.4, 2.5 Hz, 1H), 7.52 (dd, *J* = 8.3, 4.4 Hz, 1H), 7.36 (td, *J* = 8.6, 2.5 Hz, 1H), 4.86 (s, 2H).

^{13}C NMR (126 MHz, $CDCl_3$) δ 166.8 (d, *J* = 3.8 Hz), 163.1 (d, *J* = 249.5 Hz), 143.1, 135.1 (d, *J* = 2.4 Hz), 134.4, 133.4, 124.4 (d, *J* = 8.4 Hz), 120.7 (d, *J* = 23.9 Hz), 118.7, 118.7, 111.2 (d, *J* =23.9 Hz), 107.4, 49.9.

^{19}F NMR (376 MHz, $CDCl_3$) δ -111.67 (dd, *J* = 12.2, 8.2 Hz).

HRMS (ESI+, *m/z*) calculated for $C_{15}H_9FN_2O$ $[M + H]^+$: 253.0772; found: 253.0772;.

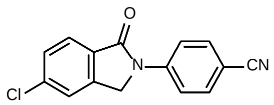

1k: white solid; yield 72%, m.p. = 262-263 °C.

^{1}H NMR (400 MHz, $CDCl_3$) δ 8.08 – 8.00 (m, 2H), 7.87 (d, *J* = 8.1 Hz, 1H), 7.76 – 7.68 (m, 2H), 7.59 – 7.48 (m, 2H), 4.86 (s, 2H).

^{13}C NMR (126 MHz, $CDCl_3$) δ 166.8, 143.0, 141.2, 139.4, 133.4, 130.9, 129.5, 125.8, 123.2, 118.7, 118.6, 107.4, 49.8.

HRMS (ESI+, *m/z*) calculated for $C_{15}H_9ClN_2O$ $[M + H]^+$: 269.0476; found: 269.0477;.

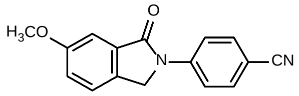

1l: white solid; yield 83%, m.p. = 229-231 °C.

^{1}H NMR (400 MHz, $CDCl_3$) δ 8.08 – 7.98 (m, 2H), 7.75 – 7.64 (m, 2H), 7.42 (d, *J* = 8.4 Hz, 1H), 7.38 (d, *J* = 2.4 Hz, 1H), 7.20 (dd, *J* = 8.3, 2.5 Hz, 1H), 4.80 (s, 2H), 3.89 (s, 3H).

^{13}C NMR (101 MHz, $CDCl_3$) δ 167.9, 160.4, 143.4, 133.7, 133.3, 131.9, 123.6, 121.6, 118.9, 118.5, 106.9, 106.7, 55.7, 49.9.

HRMS (ESI+, *m/z*) calculated for $C_{16}H_{12}N_2O_2$ $[M + H]^+$: 265.0972; found: 265.0973;.

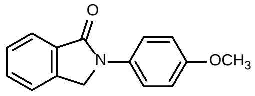

1m: white solid; yield 73%, m.p. = 219-220 °C.

^{1}H NMR (400 MHz, $CDCl_3$) δ 7.97 – 7.84 (m, 1H), 7.80 – 7.65 (m, 2H), 7.60 – 7.52 (m, 1H), 7.52 – 7.43 (m, 2H), 7.01 – 6.88 (m, 2H), 4.78 (s, 2H), 3.81 (s, 3H).

^{13}C NMR (126 MHz, $CDCl_3$) δ 167.2, 156.5, 140.1, 133.2, 132.6, 131.7, 128.2, 123.9, 122.5, 121.4, 114.3, 55.4, 51.1.

HRMS (ESI+, *m/z*) calculated for $C_{15}H_{14}NO_2$ $[M + H]^+$: 240.1025; found: 240.1028;.

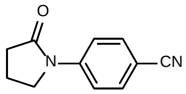

1n: white solid; yield 64%, m.p. = 117-118 °C.

^{1}H NMR (400 MHz, $CDCl_3$) δ 7.85 – 7.72 (m, 2H), 7.70 – 7.57 (m, 2H), 3.87 (t, *J* = 7.1 Hz, 2H), 2.65 (t, *J* = 8.1 Hz, 2H), 2.25 – 2.15(m, 2H).

^{13}C NMR (101 MHz, $CDCl_3$) δ 174.8, 143.1, 132.9, 119.1, 118.8, 106.9, 48.2, 32.7, 17.7.

HRMS (ESI+, *m/z*) calculated for $C_{11}H_{10}N_2O$ [M + H]$^+$: 187.0866; found: 187.0869;.

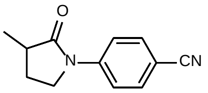

1o: white solid; yield 90%, m.p. = 103-104 °C.

^{1}H NMR (400 MHz, $CDCl_3$) δ 7.84 – 7.76 (m, 2H), 7.66 – 7.59 (m, 2H), 3.82 – 3.74 (m, 2H), 2.77 – 2.64 (m, 1H), 2.46 – 2.36 (m, 1H), 1.86 – 1.74 (m, 1H), 1.30 (d, *J* = 7.1 Hz, 3H).

^{13}C NMR (101 MHz, $CDCl_3$) δ 177.3, 143.3, 132.9, 119.0, 118.9, 106.8, 46.1, 38.4, 26.7, 15.9.

HRMS (ESI+, *m/z*) calculated for $C_{12}H_{12}N_2O$ [M + H]$^+$: 201.1022; found: 201.1021;.

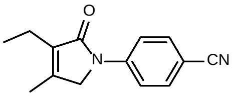

1q: light yellow solid; yield 76%, m.p. = 168-169 °C.

^{1}H NMR (500 MHz, $CDCl_3$) δ 7.89 – 7.80 (m, 2H), 7.62 – 7.53 (m, 2H), 4.14 (s, 2H), 2.29 (q, *J* = 7.6 Hz, 2H), 2.05 (s, 3H), 1.06 (t, *J* = 7.6 Hz, 3H).

^{13}C NMR (126 MHz, $CDCl_3$) δ 171.2, 146.5, 143.1, 135.3, 133.1, 118.9, 117.3, 105.6, 53.7, 16.7, 12.9, 12.8.

HRMS (ESI+, *m/z*) calculated for $C_{14}H_{14}N_2O$ [M + H]$^+$: 227.1179; found: 227.1175;.

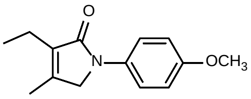

1r: light yellow solid; yield 76%, m.p. = 176-177 °C.

^{1}H NMR (400 MHz, $CDCl_3$) δ 7.58 (d, *J* = 9.0 Hz, 2H), 6.86 (d, *J* = 9.0 Hz, 2H), 4.09 (s, 2H), 3.77 (s, 3H), 2.31 (q, *J* = 7.5 Hz, 2H), 2.01 (s, 3H), 1.09 (t, *J* = 7.6 Hz, 3H).

^{13}C NMR (126 MHz, $CDCl_3$) δ 170.7, 155.7, 144.6, 135.2, 132.9, 119.9, 114.0, 55.3, 54.5, 16.8, 12.9, 12.7.

HRMS (ESI+, *m/z*) calculated for $C_{14}H_{18}NO_2$ [M + H]$^+$: 232.1338; found: 232.1342;.

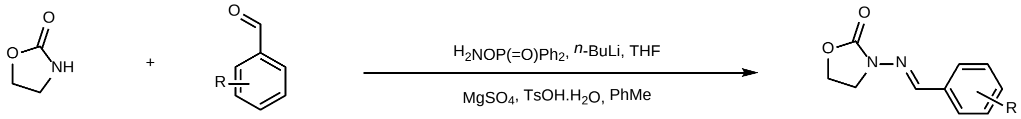

To a solution of the appropriate 2-oxazolidinone[2] in dry THF (0.05 M) was added *n*-BuLi (2.2 M in hexane, 1.1 eq) at -78 °C. After 1 h, *o*-(2,4-diphenylphosphinyl)-hydroxylamine (1.2 eq) was added, the reaction was allowed to warm to room temperature and further stirred overnight. The mixture

was filtered and concentrated to afford the corresponding N-amino-2-oxazolidinone. Without purification, the N-amino-2-oxazolidinone was dissolved in toluene (0.5 M), and aldehyde (2.0 eq), magnesium sulfate (4.0 eq) and TsOH.H_2O (0.05 eq) were added. The mixture was stirred under reflux for 2 hours then concentrated and purified by flash chromatography to afford the corresponding hydrazone.

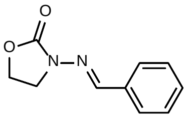

1d: white solid; yield 66%, m.p. = 139-141 °C.

^{1}H NMR (400 MHz, $CDCl_3$) δ 7.80 – 7.69 (m, 3H), 7.42 – 7.36 (m, 3H), 4.58 – 4.52 (m, 2H), 3.96 (t, *J* = 8.0 Hz, 2H).

^{13}C NMR (101 MHz, $CDCl_3$) δ 154.3, 144.2, 133.6, 130.2, 128.6, 127.4, 61.3, 42.4.

HRMS (ESI+, *m/z*) calculated for $C_{10}H_{10}N_2O_2$ [M + H]$^+$: 191.0815; found: 191.0818;.

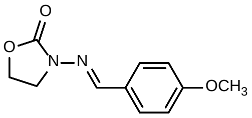

1e: white solid; yield 80%, m.p. = 195-197 °C.

^{1}H NMR (400 MHz, $CDCl_3$) δ 7.72 – 7.62 (m, 3H), 6.91 (d, *J* = 8.7 Hz, 2H), 4.58 – 4.47 (m, 2H), 3.99 – 3.89 (m, 2H), 3.83 (s, 3H).

^{13}C NMR (101 MHz, $CDCl_3$) δ 161.4, 154.4, 144.4, 129.0, 126.5, 114.1, 61.2, 55.4, 42.7.

HRMS (ESI+, *m/z*) calculated for $C_{11}H_{12}N_2O_3$ [M + H]$^+$: 221.0921; found: 221.0923;.

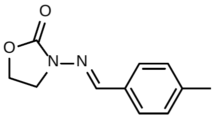

1f: white solid; yield 76%, m.p. = 179-180 °C.

^{1}H NMR (400 MHz, $CDCl_3$) δ 7.66 (s, 1H), 7.62 (d, *J* = 8.0 Hz, 2H), 7.19 (d, *J* = 7.9 Hz, 2H), 4.57 – 4.43 (m, 2H), 3.98 – 3.84 (m, 2H), 2.36 (s, 3H).

^{13}C NMR (101 MHz, $CDCl_3$) δ 154.4, 144.4, 140.6, 130.9, 129.4, 127.4, 61.3, 42.4, 21.4.

HRMS (ESI+, *m/z*) calculated for $C_{11}H_{12}N_2O_2$ [M + H]$^+$: 205.0972; found: 205.0969;.

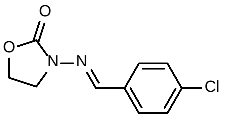

1g: white solid; yield 55%, m.p. = 166-167 °C.

^{1}H NMR (400 MHz, $CDCl_3$) δ 7.72 - 7.64 (m, 2H), 7.66 (s, 1H), 7.36 (d, *J* = 8.5 Hz, 2H), 4.59 – 4.52 (m, 2H), 3.95 (t, *J* = 8.0 Hz, 2H).

^{13}C NMR (101 MHz, $CDCl_3$) δ 154.2, 142.9, 136.1, 132.2, 129.0, 128.6, 61.3, 42.6.

HRMS (ESI+, *m/z*) calculated for $C_{10}H_9ClN_2O_2$ $[M + H]^+$: 225.0425; found: 225.0427;.

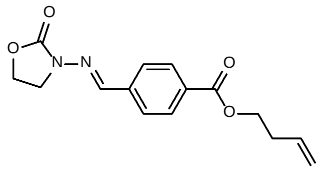

1h: white solid; yield 55%, m.p. = 186-187 °C.

^{1}H NMR (500 MHz, $CDCl_3$) δ 8.05 (d, *J* = 8.0 Hz, 2H), 7.81 (d, *J* = 8.0 Hz, 2H), 7.76 (s, 1H), 5.91 - 5.83 (m, 1H), 5.18 (dd, *J* = 17.2, 1.2 Hz, 1H), 5.12 (d, *J* = 10.2 Hz, 1H), 4.62 – 4.54 (m, 2H), 4.38 (t, *J* = 6.7 Hz, 2H), 4.01 – 3.94 (m, 2H), 2.54 (q, *J* = 6.7 Hz, 2H), 1.59 (d, *J* = 3.2 Hz, 2H).

^{13}C NMR (101 MHz, $CDCl_3$) δ 165.9, 154.1, 142.8, 137.8, 133.8, 131.5, 129.8, 127.2, 117.4, 64.1, 61.4, 42.4, 33.1.

HRMS (ESI+, *m/z*) calculated for $C_{15}H_{17}N_2O_4$ $[M + H]^+$: 289.1188; found: 289.1191;.

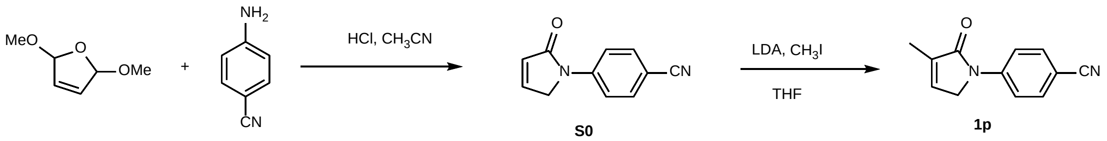

To a solution of 4-aminobenzonitrile[3] (1.0 eq) and 2,5-dimethoxy-2,5-dihydrofuran (2.0 eq) was added. The reaction mixture was stirred at rt for 1 h, quenched with $NaHCO_3$, concentrated under reduced pressure and partitioned between EtOAc and H_2O. The aqueous phase was extracted with EtOAc and the combined organic extracts were washed with brine, dried over Na_2SO_4, concentrated and evaporated to give the residue. The crude product was purified by column chromatography to afford the product **S0**.

To a solution of **S0** (1.0 eq) in THF at -78 ^{o}C, LDA (1.0 eq) was added, and the mixture was stirred for 30 min at -78 ^{o}C and 1 h at 0 ^{o}C. The solution was recooled to -78 ^{o}C and a solution of iodomethane (1.0 eq) in THF was added dropwise. Then the solution was stirred for 5 h at -45 ^{o}C and 30 min at 0 ^{o}C, after which it was poured onto 8 mL of 3 M HCl and stirred for 1 h. The reaction was quenched by adding water and extracted with DCM three times. The organic layer was washed with brine, dried over anhydrous Na_2SO_4 and concentrated under reduced pressure. The residue was purified by column chromatography to obtain the desired product **1p**.

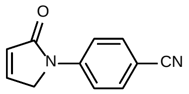

S0: yellow solid; yield 30%.

^{1}H NMR (400 MHz, $CDCl_3$) δ 7.99 – 7.82 (m, 2H), 7.75 – 7.54 (m, 2H), 7.28 (dt, *J* = 6.1, 2.0 Hz, 1H), 6.29 (dt, *J* = 6.1, 2.0 Hz, 1H), 4.48 (t, *J* = 2.0 Hz, 2H).

^{13}C NMR (126 MHz, $CDCl_3$) δ 170.3, 143.0, 142.8, 133.3, 129.1, 118.8, 117.9, 106.7, 52.6.

HRMS (ESI+, *m/z*) calculated for $C_{11}H_8N_2O$ $[M + H]^+$: 207.0529; found: 207.0534;.

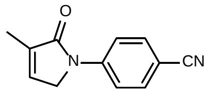

1p: light yellow solid; yield 51%, m.p. = 89-91 °C.

^{1}H NMR (400 MHz, $CDCl_3$) δ 7.89 (d, *J* = 8.9 Hz, 2H), 7.63 (d, *J* = 8.9 Hz, 2H), 6.86 (q, *J* = 1.8 Hz, 1H), 4.31 (d, *J* = 2.1 Hz, 2H), 1.95 (d, *J* = 1.9 Hz, 3H).

^{13}C NMR (101 MHz, $CDCl_3$) δ 171.1, 143.1, 136.8, 135.6, 133.3, 118.9, 117.7, 106.5, 50.4, 11.2.

HRMS (ESI+, *m/z*) calculated for $C_{12}H_{10}N_2O$ $[M + H]^+$: 199.0866; found: 199.0867;.

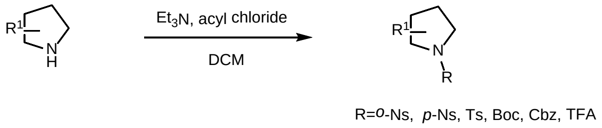

The amine (1.0 eq) and Et_3N (1.1 eq) were dissolved in dry DCM (0.5 M) and cooled to 0 °C. The sulfonyl chloride or acyl chloride (5.0 mmol, 1.2 eq) was then added. The reaction mixture was stirred at room temperature for 16 hours. The solution was then washed with 1 M HCl, dried over Na_2SO_4 and purified by column chromatography.

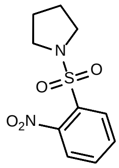

1s: white solid; yield 66%, m.p. = 81-82 °C.

^{1}H NMR (500 MHz, $CDCl_3$) δ 8.00 – 7.95 (m, 1H), 7.72 – 7.64 (m, 2H), 7.61 – 7.55 (m, 1H), 3.44 – 3.37 (m, 4H), 1.93 – 1.86 (m, 4H).

^{13}C NMR (126 MHz, $CDCl_3$) δ 148.2, 133.4, 131.9, 131.4, 130.5, 123.8, 47.9, 25.6.

HRMS (ESI+, *m/z*) calculated for $C_{10}H_{12}N_2O_4S$ $[M + H]^+$: 257.0591; found: 257.0604;.

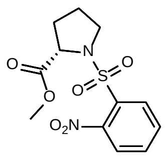

1t: light yellow solid; yield 75%, m.p. = 85-86 °C.

^{1}H NMR (400 MHz, $CDCl_3$) δ 8.10 – 8.03 (m, 1H), 7.73 – 7.65 (m, 2H), 7.64 – 7.58 (m, 1H), 4.56 (dd, *J* = 8.6, 3.1 Hz, 1H), 3.64 (s, 3H), 3.62 – 3.56 (m, 1H), 3.55 – 3.48 (m, 1H), 2.29 – 2.18 (m, 1H), 2.11 – 1.89 (m, 3H).

^{13}C NMR (126 MHz, $CDCl_3$) δ 172.1, 147.8, 133.6, 132.3, 131.6, 130.7, 123.9, 60.7, 52.2, 48.3, 30.7, 24.3.

HRMS (ESI+, *m/z*) calculated for $C_{12}H_{14}N_2O_6S$ $[M + H]^+$: 315.0645; found: 315.0644;.

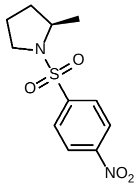

1u: white solid; yield 57%, m.p. = 130-131 °C.

^{1}H NMR (400 MHz, $CDCl_3$) δ 8.41 – 8.30 (m, 2H), 8.07 – 7.95 (m, 2H), 3.79 – 3.69 (m, 1H), 3.50 – 3.42(m, 1H), 3.20 – 3.12 (m, 1H), 1.94 – 1.80 (m, 1H), 1.78 – 1.67 (m, 1H), 1.64 – 1.48 (m, 2H), 1.29 (d, *J* = 6.4 Hz, 3H).

^{13}C NMR (101 MHz, $CDCl_3$) δ 149.9, 143.9, 128.4, 124.2, 56.5, 49.0, 33.4, 23.8, 22.5.

HRMS (ESI+, *m/z*) calculated for $C_{14}H_{10}N_2O_4$ $[M + H]^+$: 271.0713; found: 271.0712;.

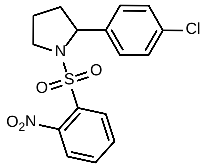

1v: colorless oil; yield 92%.

^{1}H NMR (400 MHz, $CDCl_3$) δ 7.61 – 7.47 (m, 3H), 7.40 – 7.33 (m, 1H), 7.16 – 7.05 (m, 4H), 4.97 (t, *J* = 6.2 Hz, 1H), 3.83 – 3.72 (m, 2H), 2.43 – 2.31 (m, 1H), 2.07 – 1.97 (m, 1H), 1.97 – 1.82 (m, 2H).

^{13}C NMR (126 MHz, $CDCl_3$) δ 147.6, 140.2, 133.1, 133.0, 132.9, 131.1, 130.8, 128.3, 128.0, 123.7, 63.3, 49.9, 36.7, 24.4.

HRMS (ESI+, *m/z*) calculated for $C_{16}H_{15}N_2O_4SCl$ $[M + H]^+$: 367.0514; found: 367.0514;

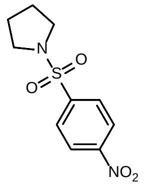

S1: white solid; yield 52%.

^{1}H NMR (400 MHz, $CDCl_3$) δ 8.42 – 8.32 (m, 2H), 8.05 – 7.96 (m, 2H), 3.32 – 3.24 (m, 4H), 1.85 – 1.76 (m, 4H).

^{13}C NMR (101 MHz, $CDCl_3$) δ 150.04, 143.03, 128.55, 124.36, 48.09, 25.38.

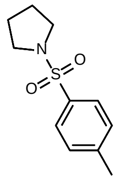

S2: white solid; yield 55%.

^{1}H NMR (400 MHz, $CDCl_3$) δ 7.74 – 7.68 (m, 2H), 7.33 – 7.29 (m, 2H), 3.25 – 3.19 (m, 4H), 2.42 (s, 3H), 1.78 – 1.70 (m, 4H).

^{13}C NMR (126 MHz, $CDCl_3$) δ 143.2, 133.8, 129.5, 127.5, 47.9, 25.1, 21.5.

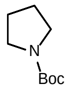

S3: colorless liquid; yield 65%.

^{1}H NMR (400 MHz, $CDCl_3$) δ 3.23 (dt, *J* = 20.2, 6.2 Hz, 4H), 1.81 – 1.69 (m, 4H), 1.38 (s, 9H).

^{13}C NMR (126 MHz, $CDCl_3$) δ 154.6, 78.8, 45.9, 45.5, 28.5, 25.7, 24.9.

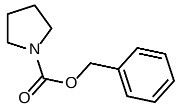

S4: colorless liquid; yield 60%.

^{1}H NMR (400 MHz, $CDCl_3$) δ 7.40 – 7.25 (m, 5H), 5.13 (s, 2H), 3.44 – 3.33 (m, 4H), 1.90 – 1.78(m, 4H).

^{13}C NMR (126 MHz, $CDCl_3$) δ 154.8, 137.0, 128.3, 127.7, 127.7, 66.4, 46.1, 45.7, 25.6, 24.8.

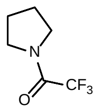

S5: colorless liquid; yield 45%.

^{1}H NMR (400 MHz, $CDCl_3$) δ 3.62 (t, *J* = 6.8 Hz, 2H), 3.55 (t, *J* = 7.0 Hz, 2H), 2.04 – 1.95 (m, 2H), 1.94 – 1.85 (m, 2H).

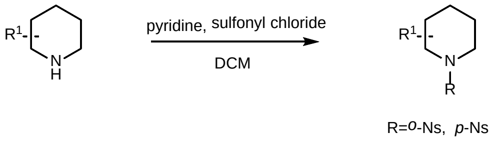

The amine[4] (1.0 eq) and pyridine (1.1 eq) were dissolved in DCM (0.5 M) and cooled to 0 °C. The sulfonyl chloride (5.0 mmol, 1.2 eq) was then added. The reaction mixture was stirred at room temperature for 16 hours. The solution was then washed with 1 M HCl (X2), dried (Na_2SO_4) and purified by column chromatography.

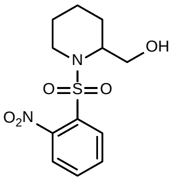

S6: colorless oil; yield 70%.

^{1}H NMR (400 MHz, $CDCl_3$) δ 8.08 – 8.04 (m, 1H), 7.74 – 7.53 (m, 3H), 4.08 – 4.00 (m, 1H), 3.86 – 3.80 (m, 1H), 3.73 (d, *J* = 13.8 Hz, 1H), 3.62 – 3.51 (m, 1H), 3.15 – 3.00 (m, 1H), 2.33 (t, *J* = 6.1 Hz, 1H), 1.77 – 1.32 (m, 6H).

^{13}C NMR (126 MHz, $CDCl_3$) δ 147.4, 133.6, 133.4, 131.8, 130.8, 124.1, 60.1, 54.8, 41.3, 24.8, 18.6.

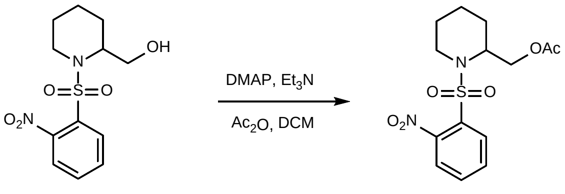

1w: colorless oil; yield 85%.

^{1}H NMR (400 MHz, $CDCl_3$) δ 8.01 – 7.95 (m, 1H), 7.67 – 7.60 (m, 2H), 7.60 – 7.55 (m, 1H), 4.33 – 4.18 (m, 2H), 4.00 (dd, *J* = 10.8, 5.4 Hz, 1H), 3.64 – 3.55 (m, 1H), 3.05 (td, *J* = 14.1, 13.6, 2.4 Hz, 1H), 1.83 (s, 3H), 1.71 – 1.60 (m, 2H), 1.57 – 1.49 (m, 2H), 1.48 – 1.35 (m, 1H), 1.33– 1.20 (m, 1H).

^{13}C NMR (101 MHz, $CDCl_3$) δ 170.2, 147.2, 133.5, 133.3, 131.6, 130.5, 123.9, 60.6, 51.4, 41.1, 24.9, 24.5, 20.3, 18.3.

HRMS (ESI+, *m/z*) calculated for $C_{14}H_{18}N_2O_6S$ [M + H]$^+$: 343.0958; found: 343.0958;

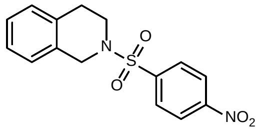

1x: white solid; yield 66%, m.p. = 173-174 °C.

^{1}H NMR (500 MHz, $CDCl_3$) δ 8.38 – 8.34 (m, 2H), 8.04 – 7.99 (m, 2H), 7.20 – 7.13 (m, 2H), 7.11 – 7.01 (m, 2H), 4.36 (s, 2H), 3.47 (t, *J* = 6.0 Hz, 2H), 2.92 (t, *J* = 6.0 Hz, 2H).

^{13}C NMR (126 MHz, $CDCl_3$) δ 150.1, 142.9, 132.7, 130.9, 128.9, 128.7, 127.1, 126.6, 126.2, 124.3, 47.4, 43.7, 28.6.

HRMS (ESI+, *m/z*) calculated for $C_{15}H_{14}N_2O_4S$ [M + H]$^+$: 319.0747; found: 319.0752;

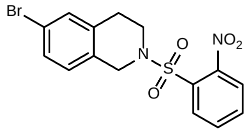

1y: white solid; yield 65%, m.p. = 161-162 °C.

^{1}H NMR (400 MHz, $CDCl_3$) δ 8.10 – 8.00 (m, 1H), 7.75 – 7.66 (m, 2H), 7.66 – 7.60 (m, 1H), 7.34 – 7.25 (m, 2H), 6.96 (d, *J* = 8.2 Hz, 1H), 4.47 (s, 2H), 3.62 (t, *J* = 5.9 Hz, 2H), 2.92 (t, *J* = 5.9 Hz, 2H).

^{13}C NMR (126 MHz, $CDCl_3$) δ 148.2, 135.4, 133.8, 132.0, 131.8, 131.7, 130.9, 130.6, 129.6, 127.8, 124.2, 120.6, 46.7, 43.2, 28.6.

HRMS (ESI+, *m/z*) calculated for $C_{15}H_{13}N_2O_4SBr$ [M + H]$^+$: 396.9852; found: 396.9852;

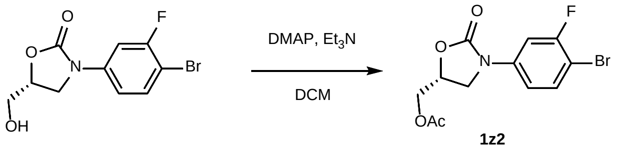

To a solution of (R)-3-(4-bromo-3-fluorophenyl)-5-(hydroxymethyl)oxazolidin-2-one[4] (1.0 eq) in DCM (20 mL), DMAP (0.1 eq), Et_3N (5.0 eq), and Ac_2O (3.0 eq) were added. The reaction was stirred overnight. The reaction mixture was then extracted with DCM (2x30 mL), washed with saturated sodium bicarbonate (30 mL) and brine (30 mL), dried over Na_2SO_4 and concentrated in vacuo. The crude product was purified by column chromatography to afford the product as a white solid (95% yield).

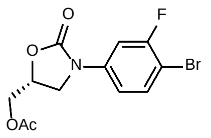

1z2: white solid; yield 95%, m.p. = 89-90 °C.

^{1}H NMR (400 MHz, $CDCl_3$) δ 7.55 – 7.48 (m, 2H), 7.16 – 7.11 (m, 1H), 4.88 (ddt, *J* = 9.0, 6.2, 4.8 Hz, 1H), 4.37 (dd, *J* = 12.3, 3.9 Hz, 1H), 4.29 (dd, *J* = 12.3, 4.9 Hz, 1H), 4.09 (t, *J* = 9.0 Hz, 1H), 3.79 (dd, *J* = 8.9, 6.3 Hz, 1H), 2.09 (s, 3H).

^{13}C NMR (101 MHz, $CDCl_3$) δ 170.4, 159.1 (d, *J* = 246.6 Hz), 153.6, 138.6 (d, *J* = 9.7 Hz), 133.5, 114.3 (d, *J* = 3.4 Hz), 106.6 (d, *J* = 27.9 Hz), 103.4 (d, *J* = 21.1 Hz), 70.0, 63.8, 46.8, 20.6.

^{19}F NMR (376 MHz, $CDCl_3$) δ -104.6.

HRMS (ESI+, *m/z*) calculated for $C_{12}H_{11}NO_4FBr$ $[M + H]^+$: 331.9928; found: 331.9926;

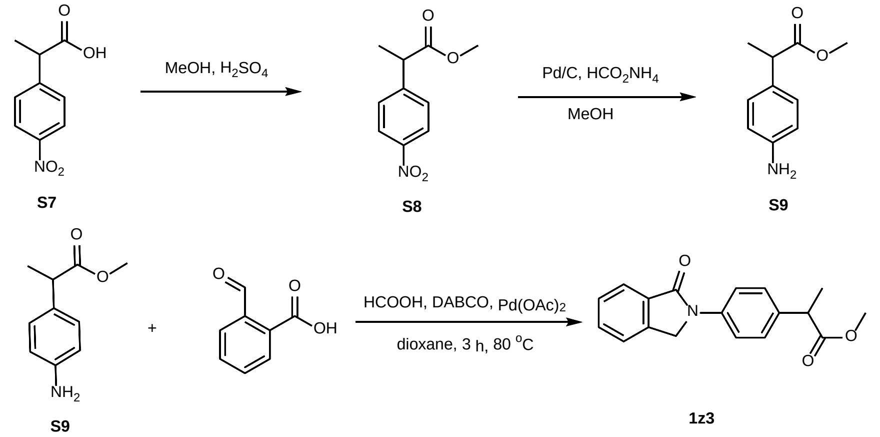

To a solution of **S7**[4] (10.0 mmol) in MeOH (20 mL), concentrated H_2SO_4 (0.05 mL) was added and the resulting solution was stirred at rt overnight. After solvent evaporation, the crude product was purified by column chromatography to afford the product **S8** as orange-brown oil (90%).

To a solution of **5** (1.0 eq) in MeOH was added 10% Pd/C (0.1 eq) followed by addition of HCO_2NH_4 (6.0 eq). The solution was stirred at rt for 3 h, then filtered through a pad of Celite and the filtrate was concentrated under reduced pressure. The residue was purified by column chromatography to afford the product **S9** as an orange oil (89%).

A mixture of 2-formylbenzoic acid (1.0 eq), the **S9** (1.2 eq), DABCO (2.0 eq), HCOOH (6.0 eq), and $Pd(OAc)_2$ (5 mol%) in 1,4-dioxane was heated in an oil bath at 80 °C for 3 h. After completion of the reaction, the mixture was cooled to room temperature and diluted with DCM (50 mL). The solid was removed by filtration, and the filtrate was washed with water (50 mL) and brine (50 mL).

The organic layer was dried over Na_2SO_4, filtered, and concentrated under reduced pressure. The residue was purified by column chromatography on silica gel to afford the desired product **1z3.**

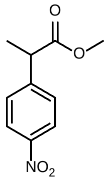

S8: orange-brown oil; yield 90%.

^{1}H NMR (400 MHz, $CDCl_3$) δ 8.20 – 8.15 (m, 2H), 7.49– 7.44 (m, 2H), 3.84 (q, *J* = 7.2 Hz, 1H), 3.68 (s, 3H), 1.54 (d, *J* = 7.2 Hz, 3H).

^{13}C NMR (126 MHz, $CDCl_3$) δ 173.6, 147.6, 147.1, 128.5, 123.8, 52.4, 45.3, 18.4.

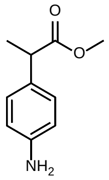

S9: orange oil; yield 89%.

^{1}H NMR (400 MHz, $CDCl_3$) δ 7.16 – 7.04 (m, 2H), 6.70 – 6.58 (m, 2H), 3.64 (s, 3H), 3.62 (q, *J* = 7.2 Hz, 1H), 3.52 (s, 2H), 1.45 (d, *J* = 7.1 Hz, 3H).

^{13}C NMR (126 MHz, $CDCl_3$) δ 175.4, 145.4, 130.3, 128.2, 115.1, 51.8, 44.4, 18.5.

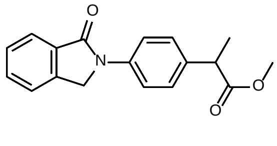

1z3: white solid; yield 95%, m.p. = 132-133 °C.

^{1}H NMR (500 MHz, $CDCl_3$) δ 7.92 (d, *J* = 7.5 Hz, 1H), 7.82 (d, *J* = 8.3 Hz, 2H), 7.62 – 7.57 (m, 1H), 7.54 – 7.48 (m, 2H), 7.36 (d, *J* = 8.3 Hz, 2H), 4.84 (s, 2H), 3.74 (q, *J* = 7.2 Hz, 1H), 3.67 (s, 3H), 1.52 (d, *J* = 7.2 Hz, 3H).

^{13}C NMR (126 MHz, $CDCl_3$) δ 174.9, 167.4, 140.0, 138.4, 136.6, 133.1, 132.0, 128.3, 128.2, 124.1, 122.6, 119.7, 52.0, 50.7, 44.8, 18.5.

HRMS (ESI+, *m/z*) calculated for $C_{18}H_{17}NO_3$ $[M + H]^+$: 296.1281; found: 296.1282;

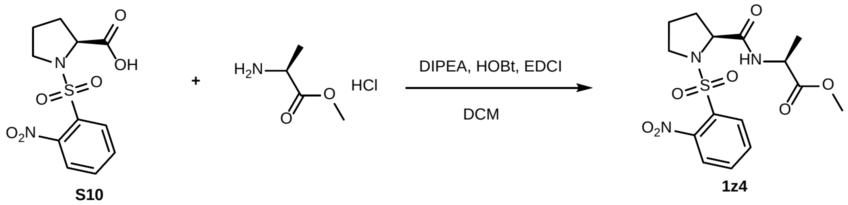

To a solution of **S10**[4] (1.0 eq) and L-alanine methyl ester hydrochloride (1.0 eq) in DCM. The mixture was cooled to 0 °C, and DIPEA (2.0 eq) was added dropwise, followed by HOBt (1.1 eq) and EDCI (1.0 eq). The reaction was stirred overnight and extracted with DCM (2x30 mL), washed with saturated sodium bicarbonate (30 mL) and brine (30 mL), dried over Na_2SO_4 and concentrated in vacuo. The crude product was purified by column chromatography to afford the product.

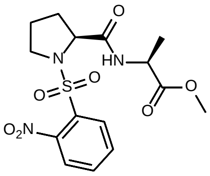

1z4: white crystal; yield 46%.

^{1}H NMR (400 MHz, $CDCl_3$) δ 8.09– 8.04 (m, 1H), 7.75 – 7.68 (m, 2H), 7.68 – 7.63 (m, 1H), 7.02 (d, J = 7.3 Hz, 1H), 4.43 (dd, J = 8.4, 2.7 Hz, 1H), 4.39 (q, J = 7.2 Hz, 1H), 3.66 (s, 3H), 3.62 – 3.55 (m, 2H), 2.29 – 2.20 (m, 1H), 2.13 – 2.00 (m, 1H), 1.98 – 1.88 (m, 2H), 1.32 (d, J = 7.2 Hz, 3H).

^{13}C NMR (126 MHz, $CDCl_3$) δ 172.5, 170.4, 148.3, 134.1, 131.9, 131.4, 131.3, 124.5, 62.2, 52.3, 49.3, 48.2, 30.9, 24.5, 18.0.

HRMS (ESI+, *m/z*) calculated for $C_{15}H_{19}N_3O_7S$ $[M + H]^+$: 386.1016; found: 386.1017;

5. General procedure for C(sp3)–H functionalization of N-heterocycles

In a dried Schlenk tube, N-heterocyclic compounds (0.2 mmol), NFSI/Selectflour II (0.3 mmol), ligand (0.024 mmol, 12 mol%) and CuOAc (0.02 mmol, 10 mol%) were dissolved in CH_3CN (***analytical grade without drying***, 1.0 mL) under a N_2 atmosphere, then ROH (0.6 mmol) was added. The reaction mixture was stirred at certain temperature for 18-24 h. Upon completion, saturated sodium bicarbonate (30 mL) was added and the reaction was extracted with DCM (2x30 mL), washed with and brine (30 mL), dried over Na_2SO_4 and concentrated in vacuo. The residue was purified by column chromatography on silica gel with a gradient eluent of petroleum ether and ethyl acetate to provide the desired product.

Note: *The C-H functionalization reaction with alcohols requires additional 1-3 eq H_2O to accelerate the reaction.*

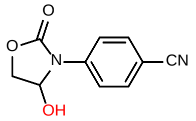

2a: white solid; yield 89%.

^{1}H NMR (400 MHz, Acetone) δ 8.03 – 7.87 (m, 2H), 7.87 – 7.66 (m, 2H), 6.19 (d, *J* = 8.5 Hz, 1H), 6.14 – 6.03 (m, 1H), 4.66 (dd, *J* = 9.8, 6.0 Hz, 1H), 4.29 (dd, *J* = 9.8, 1.6 Hz, 1H).

^{13}C NMR (101 MHz, Acetone) δ 154.8, 142.5, 133.7, 120.6, 119.2, 107.8, 80.6, 71.3.

HRMS (ESI+, *m/z*) calculated for $C_{10}H_8N_2O_3$ $[M + H]^+$: 205.0608; found: 205.0607;.

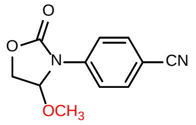

2aa: white solid; yield 95%.

^{1}H NMR (400 MHz, $CDCl_3$) δ 7.85 – 7.79 (m, 2H), 7.71 – 7.65 (m, 2H), 5.71 (dd, *J* = 6.0, 1.7 Hz, 1H), 4.49 (dd, *J* = 10.5, 5.9 Hz, 1H), 4.42 (dd, *J* = 10.5, 1.7 Hz, 1H), 3.32 (s, 3H).

^{13}C NMR (126 MHz, $CDCl_3$) δ 154.0, 140.8, 133.2, 119.7, 118.5, 108.3, 85.4, 66.2, 50.7.

HRMS (ESI+, *m/z*) calculated for $C_{11}H_{10}N_2O_3$ $[M + H]^+$: 219.0764; found: 219.0764;.

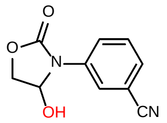

2b: colorless oil; yield 77%.

^{1}H NMR (500 MHz, DMSO) δ 8.05 – 7.95 (m, 2H), 7.68 – 7.60 (m, 2H), 7.09 (d, *J* = 8.2 Hz, 1H), 5.94 (t, *J* = 7.0 Hz, 1H), 4.56 (dd, *J* = 9.0, 6.7 Hz, 1H), 4.16 (d, *J* = 9.7 Hz, 1H).

^{13}C NMR (126 MHz, DMSO) δ 154.2, 137.9, 130.3, 127.9, 124.9, 123.0, 118.5, 111.8, 79.4, 70.4.

HRMS (ESI+, *m/z*) calculated for $C_{10}H_8N_2O_3$ $[M + H]^+$: 205.0608; found: 205.0610;.

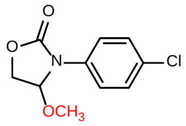

2c: colorless oil; yield 56%.

^{1}H NMR (400 MHz, $CDCl_3$) δ 7.61 – 7.53 (m, 2H), 7.38 – 7.31 (m, 2H), 5.60 (dd, *J* = 6.1, 1.6 Hz, 1H), 4.46 (dd, *J* = 10.4, 6.1 Hz, 1H), 4.37 (dd, *J* = 10.4, 1.6 Hz, 1H), 3.30 (s, 3H).

^{13}C NMR (101 MHz, $CDCl_3$) δ 154.7, 135.3, 131.1, 129.3, 122.2, 86.2, 66.5, 51.3.

HRMS (ESI+, *m/z*) calculated for $C_{10}H_{10}NO_3Cl$ $[M + H]^+$: 228.0422; found: 228.0427;.

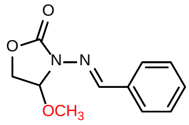

2d: white solid; yield 97%.

^{1}H NMR (400 MHz, $CDCl_3$) δ 8.79 (s, 1H), 7.77 – 7.71 (m, 2H), 7.44 – 7.38 (m, 3H), 5.60 (dd, *J* = 6.5, 2.2 Hz, 1H), 4.49 (dd, *J* = 10.3, 6.6 Hz, 1H), 4.30 (dd, *J* = 10.4, 2.2 Hz, 1H), 3.46 (s, 3H).

^{13}C NMR (101 MHz, $CDCl_3$) δ 153.0, 149.8, 134.0, 130.6, 128.7, 127.5, 85.4, 66.7, 53.5.

HRMS (ESI+, *m/z*) calculated for $C_{11}H_{12}N_2O_3$ $[M+Na]^+$: 243.0740; found: 243.0747;.

Enantiomeric excess was determined by HPLC (Chiralpak IC), *n*-hexane/*i*-propanol =85: 15, 25 °C, 254 nm, 1.0 mL/min, retention times: t_R (minor) 3.8 min, t_R (major) 7.9 min, ee = racemate;

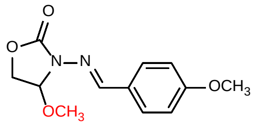

2e: colorless oil; yield 95%.

^{1}H NMR (400 MHz, $CDCl_3$) δ 8.64 (s, 1H), 7.66 (d, *J* = 8.8 Hz, 2H), 6.90 (d, *J* = 8.8 Hz, 2H), 5.58 (dd, *J* = 6.5, 2.2 Hz, 1H), 4.46 (dd, *J* = 10.4, 6.6 Hz, 1H), 4.26 (dd, *J* = 10.4, 2.2 Hz, 1H), 3.82 (s, 3H), 3.42 (s, 3H).

^{13}C NMR (101 MHz, $CDCl_3$) δ 161.5, 153.1, 149.5, 129.1, 126.6, 114.0, 85.2, 66.5, 55.3, 53.1.

HRMS (ESI+, *m/z*) calculated for $C_{12}H_{14}N_2O_4$ $[M + H]^+$: 251.1026; found: 251.1028;.

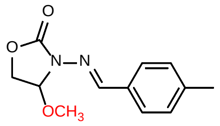

2f: white solid; yield 92%.

^{1}H NMR (400 MHz, $CDCl_3$) δ 8.69 (s, 1H), 7.63 (d, *J* = 8.1 Hz, 2H), 7.21 (d, *J* = 8.0 Hz, 2H), 5.60 (dd, *J* = 6.5, 2.1 Hz, 1H), 4.47 (dd, *J* = 10.4, 6.5 Hz, 1H), 4.28 (dd, *J* = 10.4, 2.1 Hz, 1H), 3.44 (s, 3H), 2.38 (s, 3H).

^{13}C NMR (101 MHz, $CDCl_3$) δ 153.2, 149.8, 141.1, 131.3, 129.5, 127.6, 85.3, 66.6, 53.3, 21.5.

HRMS (ESI+, *m/z*) calculated for $C_{12}H_{14}N_2O_3$ $[M + H]^+$: 235.1077; found: 235.1079;.

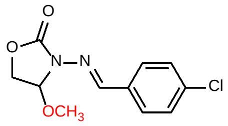

2g: white solid; yield 85%.

^{1}H NMR (400 MHz, $CDCl_3$) δ 8.73 (s, 1H), 7.65 (d, *J* = 8.5 Hz, 2H), 7.36 (d, *J* = 8.5 Hz, 2H), 5.58 (dd, *J* = 6.5, 2.1 Hz, 1H), 4.49 (dd, *J* = 10.4, 6.5 Hz, 1H), 4.29 (dd, *J* = 10.4, 2.1 Hz, 1H), 3.44 (s, 3H).

^{13}C NMR (101 MHz, $CDCl_3$) δ 152.9, 148.2, 136.5, 132.5, 128.9, 128.6, 85.3, 66.6, 53.4.

HRMS (ESI+, *m/z*) calculated for $C_{11}H_{11}N_2O_3Cl$ $[M + H]^+$: 255.0531; found: 255.0530;.

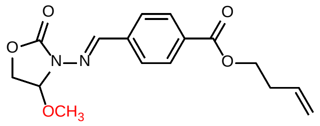

2h: white solid; yield 89%.

^{1}H NMR (400 MHz, $CDCl_3$) δ 8.88 (s, 1H), 8.06 (d, *J* = 8.1 Hz, 2H), 7.80 (d, *J* = 8.1 Hz, 2H), 5.93 – 5.79 (m, 1H), 5.60 (d, *J* = 5.3 Hz, 1H), 5.20 – 5.10 (m, 2H), 4.51 (dd, *J* = 10.3, 6.5 Hz, 1H), 4.39 (t, *J* = 6.7 Hz, 2H), 4.32 (d, *J* = 10.2 Hz, 1H), 3.48 (s, 3H), 2.54 (q, *J* = 6.6 Hz, 2H).

^{13}C NMR (101 MHz, $CDCl_3$) δ 166.0, 152.7, 148.4, 138.2, 133.9, 131.9, 129.9, 127.3, 117.4, 85.6, 66.8, 64.2, 53.8, 33.1.

HRMS (ESI+, *m/z*) calculated for $C_{16}H_{18}N_2O_5$ $[M + Na]^+$: 341.1113; found: 341.1114;.

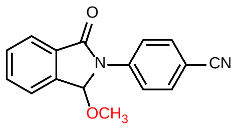

2i: white solid; yield 80%.

^{1}H NMR (400 MHz, $CDCl_3$) δ 8.12 (d, *J* = 8.9 Hz, 2H), 7.93 (d, *J* = 8.9 Hz, 1H), 7.73-7.69 (m, 3H), 7.64-7.60 (m, 2H), 6.52 (s, 1H), 2.88 (s, 3H).

^{13}C NMR (101 MHz, $CDCl_3$) δ 166.8, 141.6, 139.2, 133.6, 133.1, 132.2, 130.7, 124.2, 123.5, 120.1, 118.8, 107.6, 86.8, 48.9.

HRMS (ESI+, *m/z*) calculated for $C_{16}H_{12}N_2O_2$ $[M + H]^+$: 265.0972; found: 265.0971;.

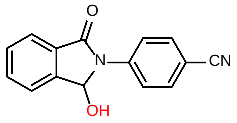

2ia: white solid; yield 86%.

^{1}H NMR (400 MHz, DMSO) δ 8.09 (d, *J* = 8.8 Hz, 2H), 7.92 (d, *J* = 8.8 Hz, 2H), 7.82 (d, *J* = 7.5 Hz, 1H), 7.79 – 7.71 (m, 2H), 7.64 (t, *J* = 7.3 Hz, 1H), 7.03 (d, *J* = 10.3 Hz, 1H), 6.65 (d, *J* = 10.2 Hz, 1H).

^{13}C NMR (101 MHz, DMSO) δ 166.0, 144.2, 141.8, 133.4, 133.0, 130.6, 129.9, 123.8, 123.2, 120.9, 119.0, 105.9, 81.7.

HRMS (ESI+, *m/z*) calculated for $C_{15}H_{10}N_2O_2$ $[M + H]^+$: 251.0815; found: 251.0815;.

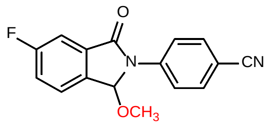

2j: colorless oil; yield 82%.

^{1}H NMR (500 MHz, $CDCl_3$) δ 8.10 (d, *J* = 8.5 Hz, 2H), 7.72 (d, *J* = 8.5 Hz, 2H), 7.66 – 7.57 (m, 2H), 7.41 (t, *J* = 8.4 Hz, 1H), 6.51 (s, 1H), 2.89 (s, 3H).

^{13}C NMR (126 MHz, $CDCl_3$) δ 165.6 (d, *J* = 3.4 Hz), 164.2 (d, *J* = 252.0 Hz), 141.3, 134.7 (d, *J* = 2.6 Hz), 134.5 (d, *J* = 8.9 Hz), 133.2, 125.4 (d, *J* = 8.6 Hz), 121.1 (d, *J* = 22.7 Hz), 120.2, 118.7, 111.2 (d, *J* = 23.94 Hz), 108.0, 86.4, 48.9.

^{19}F NMR (471 MHz, $CDCl_3$) δ -108.51 (td, *J* = 8.0, 4.5 Hz).

HRMS (ESI+, *m/z*) calculated for $C_{16}H_{11}N_2O_2F$ $[M + H]^+$: 283.0877; found: 283.0879;.

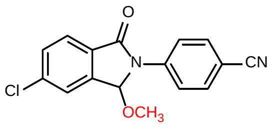

2k: colorless oil; yield 85%.

^{1}H NMR (400 MHz, $CDCl_3$) δ 8.10 (d, *J* = 8.8 Hz, 2H), 7.87 (d, *J* = 8.0 Hz, 1H), 7.72 (d, *J* = 8.8 Hz, 2H), 7.64 – 7.58 (m, 2H), 6.49 (s, 1H), 2.91 (s, 3H).

^{13}C NMR (101 MHz, $CDCl_3$) δ 165.7, 141.3, 141.0, 140.2, 133.2, 131.3, 130.6, 125.5, 124.0, 120.1, 118.7, 108.0, 86.2, 49.1.

HRMS (ESI+, *m/z*) calculated for $C_{16}H_{11}N_2O_2Cl$ $[M + H]^+$: 299.0582; found: 299.0581;.

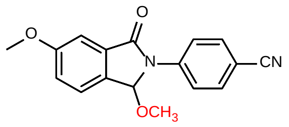

2l: colorless oil; yield 92%.

^{1}H NMR (500 MHz, $CDCl_3$) δ 7.98 (d, *J* = 7.9 Hz, 2H), 7.58 (d, *J* = 7.9 Hz, 2H), 7.38 (d, *J* = 7.9 Hz, 1H), 7.14 – 7.10 (m, 1H), 6.34 (s, 1H), 3.78 (s, 3H), 2.74 (s, 3H).

^{13}C NMR (101 MHz, $CDCl_3$) δ 166.8, 161.8, 141.6, 133.8, 133.1, 131.2, 124.6, 121.6, 120.1, 118.8, 107.56, 106.9, 86.6, 55.8, 48.7.

HRMS (ESI+, *m/z*) calculated for $C_{17}H_{14}N_2O_3$ $[M + H]^+$: 295.1077; found: 295.1077;.

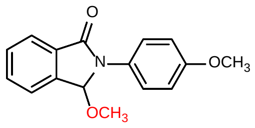

2m: colorless oil; yield 90%.

^{1}H NMR (400 MHz, $CDCl_3$) δ 7.92 (d, *J* = 7.3 Hz, 1H), 7.70 – 7.54 (m, 5H), 6.98 (d, *J* = 8.9 Hz, 2H), 6.37 (s, 1H), 3.84 (s, 3H), 2.94 (s, 3H).

^{13}C NMR (126 MHz, $CDCl_3$) δ 166.7, 157.3, 139.8, 133.0, 132.6, 130.2, 130.1, 123.9, 123.9, 123.4, 114.4, 87.8, 55.5, 49.3.

HRMS (ESI+, *m/z*) calculated for $C_{16}H_{16}NO_3$ $[M + H]^+$: 270.1130; found: 270.1129;.

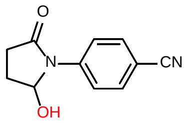

2n: light yellow crystal; yield 92%.

^{1}H NMR (400 MHz, $CDCl_3$) δ 7.76 (d, *J* = 8.7 Hz, 2H), 7.58 (d, *J* = 8.7 Hz, 2H), 5.69 (t, *J* = 6.4 Hz, 1H), 4.16 (d, *J* = 8.1 Hz, 1H), 2.82 - 2.72 (m, 1H), 2.58 – 2.29 (m, 2H), 2.12 – 1.98 (m, 1H).

^{13}C NMR (101 MHz, $CDCl_3$) δ 174.9, 141.5, 132.7, 121.7, 118.5, 107.8, 84.5, 29.9, 28.1.

HRMS (ESI+, *m/z*) calculated for $C_{11}H_{10}N_2O_2$ $[M+Na]^+$: 225.0634; found: 225.0639;.

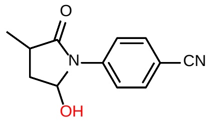

2o: light yellow oil; yield 72%.

^{1}H NMR (400 MHz, DMSO) δ 7.96 – 7.88 (m, 2H), 7.87 – 7.79 (m, 2H), 6.44 (d, *J* = 7.9 Hz, 1H), 5.70 – 5.61 (m, 1H), 2.98 – 2.86 (m, 1H), 2.16 (dd, *J* = 13.0, 8.3 Hz, 1H), 1.93 (ddd, *J* = 13.0, 10.5, 6.0 Hz, 1H), 1.14 (d, *J* = 7.1 Hz, 3H).

^{13}C NMR (101 MHz, DMSO) δ 176.9, 142.5, 132.9, 120.6, 118.9, 106.1, 81.4, 36.7, 35.0, 15.59.HRMS (ESI+, *m/z*) calculated for $C_{12}H_{12}N_2O_2$ $[M + H]^+$: 217.0972; found: 217.0976;.

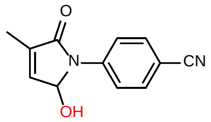

2p: white solid; yield 66%.

^{1}H NMR (400 MHz, DMSO) δ7.94 (d, *J* = 8.9 Hz, 2H), 7.84 (d, *J* = 8.9 Hz, 2H), 6.96 (t, *J* = 1.8 Hz, 1H), 6.58 (d, *J* = 9.9 Hz, 1H), 6.05 (d, *J* = 9.4 Hz, 1H), 1.86 (s, 3H).

^{13}C NMR (126 MHz, DMSO) δ 169.3, 141.6, 141.2, 134.5, 133.0, 119.3, 119.0, 105.1, 81.0, 10.5.

HRMS (ESI+, *m/z*) calculated for $C_{12}H_{10}N_2O_2$ $[M + H]^+$: 215.0815; found: 215.0814;.

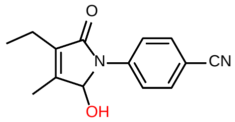

2q: white solid; yield 59%.

^{1}H NMR (400 MHz, DMSO) δ 7.97 – 7.90 (m, 2H), 7.86 – 7.76 (m, 2H), 6.60 (d, *J* = 10.0 Hz, 1H), 5.83 (d, *J* = 10.0 Hz, 1H), 2.27-2.21 (m, 2H), 1.99 (s, 3H), 1.03 (t, *J* = 7.5 Hz, 3H).

^{13}C NMR (101 MHz, DMSO) δ 169.2, 150.8, 141.8, 133.0, 132.9, 119.1, 119.0, 104.7, 82.8, 16.1, 12.8, 11.2.

HRMS (ESI+, *m/z*) calculated for $C_{14}H_{14}N_2O_2$ $[M + H]^+$: 243.1128; found: 243.1127;.

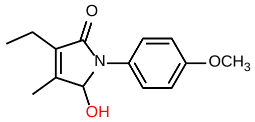

2r: white solid; yield 56%.

^{1}H NMR (400 MHz, $CDCl_3$) δ 7.51 (d, *J* = 8.5 Hz, 2H), 6.86 (d, *J* = 8.5 Hz, 2H), 5.51 (d, *J* = 7.3 Hz, 1H), 3.78 (s, 3H), 3.27 (d, *J* = 8.5 Hz, 1H), 2.28 – 2.12 (m, 2H), 2.00 (s, 3H), 1.04 (t, *J* = 7.5 Hz, 3H).

^{13}C NMR (126 MHz, $CDCl_3$) δ 169.5, 156.5, 147.6, 135.2, 130.4, 122.6, 114.1, 84.7, 55.4, 16.6, 12.9, 11.1.

HRMS (ESI+, *m/z*) calculated for $C_{14}H_{18}NO_3$ $[M + H]^+$: 248.1287; found: 248.1288;.

Note: Hemiacetals **2s-2y** were found to exist in equilibrium with their aldehyde form in solution confirmed by ^{1}H-NMR spectroscopy. So the hemiaminals **2s-2y** were convert to aminal form for characterization purpose.

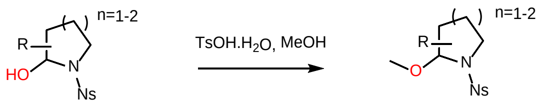

To a solution of **2s-2y**[5] (1.0 mmol) in CH_3OH (5.0 mL) was added *p*-toluenesulfonic acid (0.5 mmol) and the reaction was stirred at room temperature overnight. Upon consumption of starting material, the reaction was quenched with aqueous $NaHCO_3$ and extracted with DCM and dried with $NaSO_4$, filtered and concentrated to afford the crude product. The crude was purified via column chromatography to afford the product **2sa-2ya.**

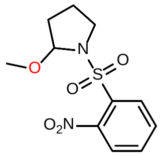

2sa: colorless oil; yield of **2s** 73 %.

^{1}H NMR (400 MHz, $CDCl_3$) δ 8.14 – 8.03 (m, 1H), 7.73 – 7.60 (m, 3H), 5.23 (d, *J* = 4.8 Hz, 1H), 3.52 (td, *J* = 8.9, 2.6 Hz, 1H), 3.35 (s, 3H), 3.29-3.22 (m, 1H), 2.18-2.08 (m, 1H), 2.02 – 1.89 (m, 2H), 1.89-1.78 (m, 1H).

^{13}C NMR (101 MHz, $CDCl_3$) δ 148.4, 133.6, 132.4, 131.6, 131.3, 124.1, 92.3, 55.5, 46.9, 32.4, 23.2.

HRMS (ESI+, *m/z*) calculated for $C_{11}H_{14}N_2O_5S$ $[M+Na]^+$: 309.0516; found: 309.0515;.

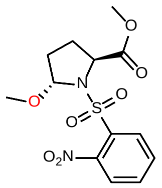

2ta: colorless oil; yield of **2t** 51%.

^{1}H NMR (400 MHz, $CDCl_3$) δ 8.15 – 8.11 (m, 1H), 7.74 – 7.70 (m, 2H), 7.63 – 7.58 (m, 1H), 5.31 (d, *J* = 4.6 Hz, 1H), 4.34 (t, *J* = 8.3 Hz, 1H), 3.74 (d, *J* = 2.8 Hz, 3H), 3.42 (s, 3H), 2.38 – 2.23 (m, 2H), 2.04-1.96 (m, 1H), 1.80 – 1.68 (m, 1H).

^{13}C NMR (101 MHz, $CDCl_3$) δ 171.9, 148.2, 134.1, 131.8, 131.6, 124.1, 92.9, 60.3, 55.3, 52.6, 32.4, 28.6.

HRMS (ESI+, *m/z*) calculated for $C_{13}H_{16}N_2O_7S$ $[M+Na]^+$: 367.0570; found: 367.0571;.

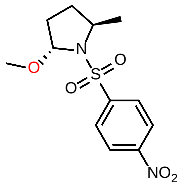

2ua: colorless oil; yield of **2u** 63%.

^{1}H NMR (400 MHz, $CDCl_3$) δ 8.38 – 8.33 (m, 2H), 8.09 – 7.98 (m, 2H), 5.11 (d, *J* = 4.9 Hz, 1H), 3.63-3.58 (m, 1H), 3.43 (s, 3H), 2.01 - 1.94 (m, 1H), 1.90 – 1.81 (m, 1H), 1.81 – 1.70 (m, 1H), 1.36 (d, *J* = 6.2 Hz, 3H), 1.29 – 1.20 (m, 1H).

^{13}C NMR (126 MHz, $CDCl_3$) δ 150.0, 145.2, 128.4, 124.3, 92.7, 57.3, 55.0, 32.0, 31.9, 23.2.

HRMS (ESI+, *m/z*) calculated for $C_{12}H_{16}N_2O_5S$ $[M+Na]^+$: 323.0672; found: 323.0671;.

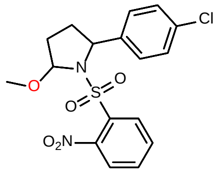

2va: colorless oil; yield of **2v** 60%.

^{1}H NMR (400 MHz, $CDCl_3$) δ 7.82 (d, *J* = 7.8 Hz, 1H), 7.72 (t, *J* = 7.5 Hz, 1H), 7.65 – 7.60 (m, 2H), 7.31 – 7.25 (m, 4H), 5.40 (d, *J* = 4.5 Hz, 1H), 4.57 (t, *J* = 8.3 Hz, 1H), 3.58 (s, 3H), 2.46 – 2.31 (m, 1H), 2.23 - 2.15 (m, 1H), 2.13 – 2.04 (m, 1H), 1.91 - 1.82 (m, 1H).

^{13}C NMR (101 MHz, $CDCl_3$) δ 148.4, 140.3, 134.0, 133.3, 131.9, 131.3, 131.2, 128.6, 128.4, 124.0, 93.5, 64.0, 55.6, 35.1, 32.4.

HRMS (ESI+, *m/z*) calculated for $C_{16}H_{15}N_2O_4SCl$ $[M+H]^+$: 367.0514; found: 367.0513;.

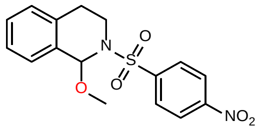

2xa: colorless oil; yield of **2x** 52%.

^{1}H NMR (400 MHz, Acetone) δ 8.33 (d, *J* = 8.7 Hz, 2H), 8.12 (d, *J* = 8.7 Hz, 2H), 7.41 – 7.34 (m, 1H), 7.25 – 7.18 (m, 2H), 7.08 – 6.99 (m, 1H), 5.99 (s, 1H), 3.92 - 3.86 (m, 1H), 3.62 – 3.51 (m, 1H), 2.77 – 2.69 (m, 1H), 2.67 – 2.59 (m, 1H).

^{13}C NMR (126 MHz, Acetone) δ 151.0, 147.3, 134.5, 134.0, 129.5, 129.5, 129.4, 129.3, 127.1, 125.2, 85.6, 55.7, 39.9, 27.5.

HRMS (ESI+, *m/z*) calculated for $C_{16}H_{16}N_2O_5S$ $[M+Na]^+$: 371.0672; found: 371.0671;.

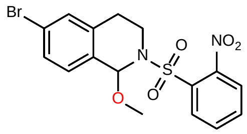

2ya: colorless oil; yield of **2y** 65%.

^{1}H NMR (500 MHz, Acetone) δ 8.17 (d, *J* = 8.0 Hz, 1H), 7.92 – 7.86 (m, 1H), 7.86 – 7.80 (m, 2H), 7.43 (d, *J* = 8.3 Hz, 1H), 7.38 (d, *J* = 8.2 Hz, 1H), 7.29 (s, 1H), 5.91 (s, 1H), 3.95 (dd, *J* = 13.9, 5.4 Hz, 1H), 3.68 – 3.56 (m, 1H), 3.41 (s, 3H), 2.81 – 2.73 (m, 1H), 2.69 – 2.55 (m, 1H).

^{13}C NMR (101 MHz, Acetone) δ 148.8, 137.3, 135.5, 133.9, 133.8, 133.2, 132.1, 131.6, 131.6, 130.2, 125.3, 122.6, 85.4, 55.9, 39.1, 27.6.

HRMS (ESI+, *m/z*) calculated for $C_{16}H_{15}N_2O_5S$ Br $[M+Na]^+$: 448.9777; found: 448.9775;.

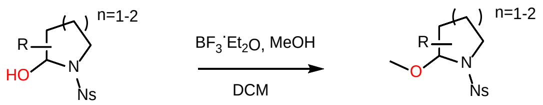

In a dried Schlenk tube, **2w** or **2z4** (0.1 mmol) was dissolved in 1.0 mL of DCM under nitrogen and cooled to -78 ^{o}C. $BF_3{\cdot}OEt_2$ (0.2 mmol) and MeOH (0.3 mmol) was added and the solution was stirred for an hour. Then it was allowed to warm to room temperature while stirring for 3 h. Upon completion, the reaction was added 10 mL H_2O for quenching. The aqueous layer was extracted with DCM (2x10 mL). The organic layers were combined, dried over anhydrous Na_2SO_4, and concentrated in vacuo. The crude product was purified by column chromatography.

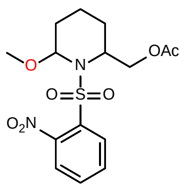

2wa: colorless oil; yield of **2w** 41%.

^{1}H NMR (400 MHz, $CDCl_3$) δ 8.12 – 8.08 (m, 1H), 7.75 – 7.64 (m, 3H), 5.13 (s, 1H), 4.52 (dd, *J* = 10.8, 8.7 Hz, 1H), 4.28 (dd, *J* = 10.8, 6.3 Hz, 1H), 4.05 – 4.00 (m, 1H), 3.42 (s, 3H), 2.08 (s, 3H), 1.95 - 1.80 (m, 2H), 1.72 - 1.66 (m, 2H), 1.44 – 1.31 (m, 2H).

^{13}C NMR (126 MHz, $CDCl_3$) δ 170.8, 147.7, 133.6, 133.6, 131.9, 131.3, 124.5, 84.9, 63.8, 55.6, 51.1, 29.9, 24.5, 21.0, 13.1.

HRMS (ESI+, *m/z*) calculated for $C_{15}H_{20}N_2O_7S$ $[M+Na]^+$: 395.0883; found: 395.0883;.

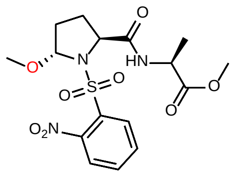

2z4a: colorless oil; yield of **2z4** 61%.

^{1}H NMR (400 MHz, $CDCl_3$) δ 8.15 – 8.08 (m, 1H), 7.74 (td, *J* = 7.4, 1.4 Hz, 2H), 7.69 - 7.64 (m, 1H), 5.40 (d, *J* = 4.5 Hz, 1H), 5.36 (d, *J* = 4.4 Hz, 1H), 4.56 – 4.47 (m, 1H), 4.08 (t, *J* = 7.8 Hz, 1H), 3.76 (s, 3H), 3.71 (s, 1H), 3.56 (s, 3H), 2.32 - 2.26 (m, 2H), 2.04 – 1.91 (m, 2H), 1.41 (d, *J* = 7.1 Hz, 3H).

^{13}C NMR (126 MHz, $CDCl_3$) δ 172.7, 170.9, 148.3, 134.5, 132.8, 131.9, 130.4, 124.6, 94.8, 62.6, 56.2, 52.5, 48.3, 32.2, 29.5, 18.3.

HRMS (ESI+, *m/z*) calculated for $C_{16}H_{21}N_3O_8S$ $[M+Na]^+$: 438.0942; found: 438.0965;.

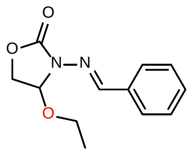

2da: white solid; yield 85%.

^{1}H NMR (500 MHz, $CDCl_3$) δ 8.85 (s, 1H), 7.76 – 7.71 (m, 2H), 7.42 - 7.41 (m, 3H), 5.62 (dd, *J* = 6.6, 2.3 Hz, 1H), 4.50 (dd, *J* = 10.2, 6.6 Hz, 1H), 4.29 (dd, *J* = 10.2, 2.3 Hz, 1H), 3.85 (dq, *J* = 9.2, 7.0 Hz, 1H), 3.62 (dq, *J* = 9.3, 7.0 Hz, 1H), 1.24 (t, *J* = 7.0 Hz, 3H).

^{13}C NMR (126 MHz, $CDCl_3$) δ 152.9, 150.1, 134.2, 130.6, 128.7, 127.5, 85.1, 67.3, 62.5, 15.2.

HRMS (ESI+, *m/z*) calculated for $C_{12}H_{14}N_2O_3$ $[M + H]^+$: 235.1078; found: 235.1077;.

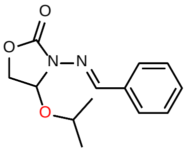

2db: colorless oil; yield 70%.

^{1}H NMR (500 MHz, $CDCl_3$) δ 8.96 (s, 1H), 7.76 – 7.64 (m, 2H), 7.48 – 7.37 (m, 3H), 5.59 (dd, *J* = 6.3, 2.0 Hz, 1H), 4.50 (dd, *J* = 9.8, 6.5 Hz, 1H), 4.23 (dd, *J* = 9.8, 2.0 Hz, 1H), 4.16 (dt, *J* = 12.2, 6.1 Hz, 1H), 1.23 (dd, *J* = 11.3, 6.2 Hz, 6H).

^{13}C NMR (126 MHz, $CDCl_3$) δ 152.8, 150.7, 134.3, 130.5, 128.7, 127.4, 84.4, 70.8, 68.4, 23.2, 22.5.

HRMS (ESI+, *m/z*) calculated for $C_{13}H_{16}N_2O_3$ $[M + H]^+$: 229.1236; found: 249.1234;.

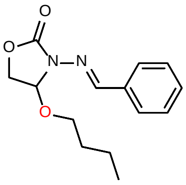

2dc: colorless oil; yield 81%.

^{1}H NMR (500 MHz, $CDCl_3$) δ 8.77 (s, 1H), 7.76 – 7.71 (m, 2H), 7.42 – 7.40 (m, 3H), 5.64 (dd, *J* = 6.5, 1.6 Hz, 1H), 4.51 – 4.44 (m, 1H), 4.32 – 4.25 (m, 1H), 3.75 (dt, *J* = 8.8, 6.4 Hz, 1H), 3.52 (dt, *J* = 8.9, 6.5 Hz, 1H), 1.60 – 1.54 (m, 2H), 1.41 – 1.32 (m, 2H), 0.89 (dd, *J* = 7.7, 7.1 Hz, 3H).

^{13}C NMR (126 MHz, $CDCl_3$) δ 153.0, 149.7, 134.1, 130.6, 128.7, 127.5, 84.7, 67.1, 66.2, 31.5, 19.2, 13.7.

HRMS (ESI+, *m/z*) calculated for $C_{14}H_{18}N_2O_3$ $[M + H]^+$: 263.1392; found: 263.1390;.

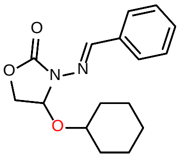

2dd: colorless oil; yield 82%.

^{1}H NMR (400 MHz, $CDCl_3$) δ 8.91 (s, 1H), 7.71 (dd, *J* = 6.1, 2.5 Hz, 2H), 7.47 – 7.33 (m, 3H), 5.63 (dd, *J* = 6.4, 2.1 Hz, 1H), 4.50 (dd, *J* = 9.8, 6.5 Hz, 1H), 4.24 (dd, *J* = 9.9, 2.2 Hz, 1H), 3.83 – 3.77 (m, 1H), 1.88 (s, 2H), 1.75 – 1.73 (m, 2H), 1.48 – 1.16 (m, 6H).

^{13}C NMR (126 MHz, $CDCl_3$) δ 152.9, 150.3, 134.3, 130.5, 128.7, 127.4, 84.1, 76.4, 68.4, 33.2, 32.6, 25.4, 24.0, 23.9.

HRMS (ESI+, *m/z*) calculated for $C_{16}H_{20}N_2O_3$ $[M + Na]^+$: 311.1372; found: 311.1374;.

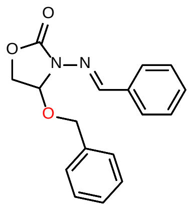

2de: white solid; yield 84%.

^{1}H NMR (400 MHz, $CDCl_3$) δ 8.89 (s, 1H), 7.75 – 7.69 (m, 2H), 7.45 – 7.39 (m, 3H), 7.37 – 7.30 (m, 5H), 5.70 (dd, *J* = 6.5, 2.2 Hz, 1H), 4.86 (d, *J* = 11.6 Hz, 1H), 4.74 – 4.69 (m, 1H), 4.50 (dd, *J* = 10.2, 6.5 Hz, 1H), 4.34 (dd, *J* = 10.2, 2.2 Hz, 1H).

^{13}C NMR (126 MHz, $CDCl_3$) δ 152.9, 150.5, 136.6, 134.1, 130.6, 128.7, 128.6, 128.2, 128.0, 127.5, 84.8, 69.4, 67.4.

HRMS (ESI+, *m/z*) calculated for $C_{17}H_{16}N_2O_3$ $[M + H]^+$: 297.1234; found: 297.1234;.

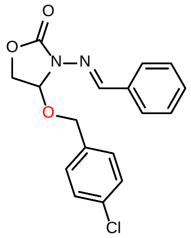

2df: colorless oil; yield 77%.

^{1}H NMR (500 MHz, $CDCl_3$) δ 8.89 (s, 1H), 7.65 – 7.62 (m, 2H), 7.38 – 7.33 (m, 3H), 7.25 – 7.22 (m, 2H), 7.20 (d, *J* = 9.2 Hz, 2H), 5.60 (dd, *J* = 6.5, 2.2 Hz, 1H), 4.77 (d, *J* = 11.8 Hz, 1H), 4.61 (d, *J* = 11.8 Hz, 1H), 4.44 (dd, *J* = 10.2, 6.5 Hz, 1H), 4.26 (dd, *J* = 10.2, 2.2 Hz, 1H).

^{13}C NMR (126 MHz, $CDCl_3$) δ 152.8, 151.0, 135.2, 134.0, 134.0, 130.7, 129.2, 128.8, 127.5, 85.3, 68.8, 67.4.

HRMS (ESI+, *m/z*) calculated for $C_{17}H_{15}N_2O_3Cl$ $[M + H]^+$: 331.0844; found: 331.0843;.

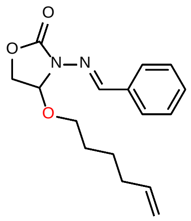

2dg: colorless oil; yield 76%.

^{1}H NMR (400 MHz, $CDCl_3$) δ 8.79 (s, 1H), 7.74 – 7.72 (m, 2H), 7.43 – 7.40 (m, 3H), 5.75 (ddt, *J* = 16.9, 10.2, 6.7 Hz, 1H), 5.63 (dd, *J* = 6.5, 2.1 Hz, 1H), 5.00 – 4.89 (m, 2H), 4.49 (dd, *J* = 10.2, 6.5 Hz, 1H), 4.29 (dd, *J* = 10.2, 2.2 Hz, 1H), 3.76 (dt, *J* = 9.2, 6.4 Hz, 1H), 3.53 (dt, *J* = 9.2, 6.4 Hz,

1H), 2.04 (dd, J = 14.2, 7.2 Hz, 2H), 1.64 – 1.57 (m, 2H), 1.49 – 1.40 (m, 2H).

^{13}C NMR (126 MHz, $CDCl_3$) δ 153.0, 149.8, 138.3, 134.1, 130.6, 128.7, 127.5, 114.7, 84.8, 67.1, 66.3, 33.3, 28.9, 25.2.

HRMS (ESI+, m/z) calculated for $C_{16}H_{20}N_2O_3$ $[M + H]^+$: 289.1546; found: 289.1547;.

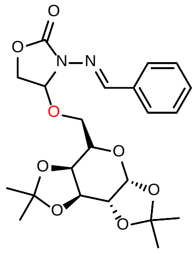

2dh: colorless oil; yield 85%.

^{1}H NMR (400 MHz, $CDCl_3$) δ 8.70 (s, 1H), 7.75 (dd, J = 6.5, 2.9 Hz, 2H), 7.38 (dd, J = 6.8, 3.6 Hz, 3H), 5.74 (dd, J = 6.3, 1.9 Hz, 1H), 5.53 (d, J = 5.0 Hz, 1H), 4.58 (dd, J = 7.9, 2.3 Hz, 1H), 4.48 (dd, J = 10.3, 6.4 Hz, 1H), 4.39 (dd, J = 10.4, 2.0 Hz, 1H), 4.31 (dd, J = 5.0, 2.4 Hz, 1H), 4.19 (dd, J = 7.9, 1.7 Hz, 1H), 3.98 (t, J = 6.3 Hz, 1H), 3.83 (dd, J = 10.2, 7.1 Hz, 1H), 3.71 (dd, J = 10.2, 5.5 Hz, 1H), 1.46 (s, 3H), 1.40 (s, 3H), 1.31 (s, 3H), 1.27 (s, 3H).

^{13}C NMR (126 MHz, $CDCl_3$) δ 153.0, 149.3, 134.0, 130.5, 128.6, 127.6, 109.4, 108.7, 96.3, 84.4, 70.7, 70.5, 70.4, 66.9, 66.4, 64.7, 26.0, 25.9, 24.8, 24.3.

HRMS (ESI+, m/z) calculated for $C_{22}H_{28}N_2O_8$ $[M+Na]^+$: 471.1738; found: 471.1740;.

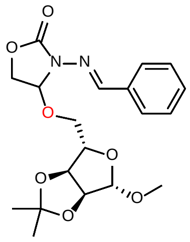

2di: colorless oil; yield 83%.

^{1}H NMR (400 MHz, $CDCl_3$) δ 8.98 (d, J = 14.6 Hz, 1H), 7.71 (dt, J = 7.3, 2.1 Hz, 2H), 7.48 – 7.33 (m, 3H), 5.61 (ddd, J = 6.6, 4.6, 2.2 Hz, 1H), 4.95 (d, J = 2.9 Hz, 1H), 4.64 (dd, J = 17.1, 5.9 Hz, 1H), 4.58 – 4.46 (m, 2H), 4.37 – 4.26 (m, 2H), 3.89 (ddd, J = 10.0, 7.3, 5.4 Hz, 1H), 3.66 (ddd, J = 17.2, 9.8, 6.8 Hz, 1H), 3.29 (d, J = 10.3 Hz, 3H), 1.46 (d, J = 3.1 Hz, 3H), 1.27 (d, J = 6.4 Hz, 3H).

^{13}C NMR (126 MHz, $CDCl_3$) δ 152.7, 152.6, 150.8, 150.7, 134.1, 134.0, 130.7, 128.7, 127.5, 127.5, 112.5, 112.5, 109.6, 109.3, 85.9, 85.9, 85.1, 85.0, 84.9, 84.9, 81.8, 81.7, 68.6, 68.4, 67.3, 67.1, 55.0, 54.9, 26.4, 26.4, 24.8.

HRMS (ESI+, *m/z*) calculated for $C_{19}H_{24}N_2O_7$ [M+Na]$^+$: 415.1476; found: 415.1474;.

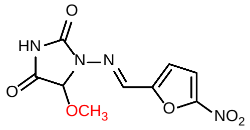

2z1: light yellow solid; yield 51%.

^{1}H NMR (400 MHz, DMSO) δ 11.81 (s, 1H), 8.18 (s, 1H), 7.79 (d, *J* = 3.9 Hz, 1H), 7.27 (d, *J* = 3.9 Hz, 1H), 5.83 (s, 1H), 3.27 (s, 3H).

^{13}C NMR (126 MHz, DMSO) δ 167.9, 152.1, 152.0, 151.2, 133.9, 116.2, 114.5, 83.4, 52.2.

HRMS (ESI+, *m/z*) calculated for $C_9H_8N_4O_6$ [M + H]$^+$: 269.0517; found: 269.0510;.

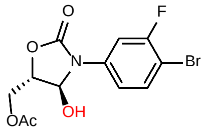

2z2: colorless oil; yield 72%.

^{1}H NMR (500 MHz, DMSO) δ 7.75 (t, *J* = 8.5 Hz, 1H), 7.67 - 7.64 (m, 1H), 7.45 (d, *J* = 8.8 Hz, 1H), 7.26 (s, 1H), 5.92 (d, *J* = 5.2 Hz, 1H), 5.70 (s, 1H), 4.84 – 4.80 (m, 1H), 4.57 (s, 1H), 4.32 - 4.25 (m, 2H), 2.09 (s, 1H), 2.00 (s, 3H).

^{13}C NMR (126 MHz, DMSO) δ 170.1, 158.2 (d, J = 244.44 Hz), 153.2, 137.9 (d, *J* = 9.9 Hz), 133.5, 117.8 (d, *J* = 3.2 Hz), 108.7 (d, J = 27.72 Hz), 102.8 (d, J = 21.42 Hz), 81.1, 79.0, 62.9, 20.5.

^{19}F NMR (376 MHz, $CDCl_3$) δ -104.26 – -104.45 (m).

HRMS (ESI+, *m/z*) calculated for $C_{12}H_{11}NO_5FBr$ [M + H]$^+$: 347.9877; found: 347.9876;.

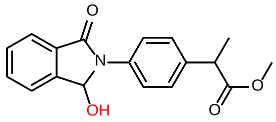

2z3: white solid; yield 73%.

^{1}H NMR (400 MHz, $CDCl_3$) δ 7.66 – 7.53 (m, 5H), 7.37 (t, *J* = 7.5 Hz, 1H), 7.26 – 7.24 (m, 2H), 6.29 (d, *J* = 1.7 Hz, 1H), 4.01 (s, 1H), 3.69 (dd, *J* = 14.3, 7.1 Hz, 1H), 3.62 (d, *J* = 1.5 Hz, 3H), 1.47 (d, *J* = 7.2 Hz, 3H).

^{13}C NMR (101 MHz, $CDCl_3$) δ 174.9, 166.5, 142.8, 137.3, 136.0, 132.8, 131.2, 130.1, 128.0, 123.7, 123.2, 122.0, 82.9, 52.1, 44.9, 18.5.

HRMS (ESI+, *m/z*) calculated for $C_{18}H_{17}NO_4$ [M + H]$^+$: 312.1230; found: 312.1233;.

6. Synthetic applications of C(sp3)–H hydroxylated product of N-heterocycles

$AlMe_3$ as the nucleophile[4]: in a dried Schlenk tube, the the C-H hydroxylated product **2** (0.1 mmol) was dissolved in 1.0 mL of DCM under nitrogen and cooled to -78 °C. DAST (0.1 mmol) was added and the solution was stirred for an hour at rt. The reaction was then placed back into -78 °C cold bath, where nucleophilic reagent (0.3 mmol) was then added dropwise. The reaction mixture was stirred at -78 °C for 2 h, then allowed to warm to room temperature while stirring for 1 h. Upon completion, the reaction was added 10 mL H_2O for quenching. The aqueous layer was extracted with DCM (2x10 mL). The organic layers were combined, dried over anhydrous Na_2SO_4, and concentrated in vacuo. The crude product was purified by column chromatography (eluting with petroleum ether/ethyl acetate 10 % - 30 %).

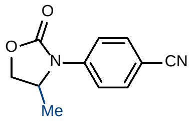

3a: colorless oil; yield 82%.

^{1}H NMR (400 MHz, $CDCl_3$) δ 7.71 – 7.59 (m, 4H), 4.65 – 4.54 (m, 2H), 4.14 – 4.04 (m, 1H), 1.42 (d, *J* = 6.1 Hz, 3H).

^{13}C NMR (126 MHz, $CDCl_3$) δ 154.6, 140.8, 133.2, 120.1, 118.6, 107.5, 68.6, 51.6, 18.3.

HRMS (ESI+, *m/z*) calculated for $C_{11}H_{10}N_2O_2$ $[M+Na]^+$: 225.0634; found: 225.0624;.

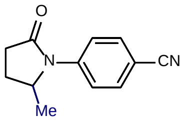

3b: colorless oil; yield 91%.

^{1}H NMR (400 MHz, $CDCl_3$) δ 7.72 – 7.58 (m, 4H), 4.47 – 4.35 (m, 1H), 2.70 (ddd, *J* = 16.9, 9.3, 7.4 Hz, 1H), 2.55 (ddd, *J* = 17.3, 9.5, 6.2 Hz, 1H), 2.39 (ddt, *J* = 12.8, 9.5, 7.5 Hz, 1H), 1.84-1.76 (m, 1H), 1.27 (d, *J* = 6.2 Hz, 3H).

^{13}C NMR (126 MHz, $CDCl_3$) δ 174.4, 141.8, 132.9, 122.2, 118.7, 107.8, 54.7, 31.3, 26.2, 19.6.

HRMS (ESI+, *m/z*) calculated for $C_{12}H_{12}N_2O$ $[M + H]^+$: 201.1022; found: 201.1024;.

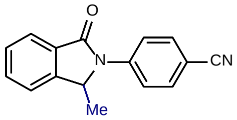

3c: white solid; yield 93%.

^1H NMR (400 MHz, $CDCl_3$) δ 7.93 (d, *J* = 7.5 Hz, 1H), 7.84 (d, *J* = 8.6 Hz, 2H), 7.73 (d, *J* = 8.6 Hz, 2H), 7.66 (t, *J* = 7.4 Hz, 1H), 7.55 - 751 (m, 2H), 5.27 (dd, *J* = 12.9, 6.3 Hz, 1H), 1.52 (d, *J* = 6.6 Hz, 3H).

^{13}C NMR (126 MHz, $CDCl_3$) δ 167.1, 145.9, 141.4, 133.1, 132.9, 130.9, 128.8, 124.5, 122.1, 121.9, 118.8, 107.6, 56.2, 18.8.

HRMS (ESI+, *m/z*) calculated for $C_{16}H_{12}N_2O$ $[M+H]^+$: 249.1022; found: 249.1025;.

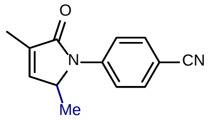

3d: colorless oil; yield 87%.

^1H NMR (400 MHz, $CDCl_3$) δ 7.74 – 7.63 (m, 4H), 6.82 (dd, *J* = 3.4, 1.6 Hz, 1H), 4.70 – 4.60 (m, 1H), 1.95 (t, *J* = 1.7 Hz, 3H), 1.29 (d, *J* = 6.7 Hz, 3H).

^{13}C NMR (101 MHz, $CDCl_3$) δ 170.5, 142.5, 141.3, 134.5, 133.0, 120.6, 118.8, 106.9, 55.8, 17.2, 11.0.

HRMS (ESI+, *m/z*) calculated for $C_{13}H_{12}N_2O$ $[M+H]^+$: 213.1022; found: 213.1022;.

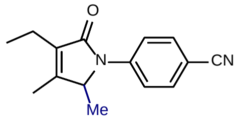

3e: white solid; yield 94%.

^1H NMR (400 MHz, $CDCl_3$) δ 7.72 (d, *J* = 8.9 Hz, 2H), 7.65 (d, *J* = 8.9 Hz, 2H), 4.47 (q, *J* = 6.6 Hz, 1H), 2.33 (q, *J* = 7.5 Hz, 2H), 2.04 (s, 3H), 1.30 (d, *J* = 6.7 Hz, 3H), 1.10 (t, *J* = 7.6 Hz, 3H).

^{13}C NMR (126 MHz, $CDCl_3$) δ 170.3, 151.9, 141.5, 133.9, 133.1, 120.3, 119.0, 106.4, 58.6, 16.9, 16.7, 13.0, 11.9.

HRMS (ESI+, *m/z*) calculated for $C_{15}H_{16}N_2O$ $[M+H]^+$: 241.1335; found: 241.1337;.

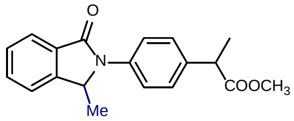

3f: white solid; yield 94%.

^1H NMR (400 MHz, $CDCl_3$) δ 7.93 (d, *J* = 7.5 Hz, 1H), 7.61 (t, *J* = 7.4 Hz, 1H), 7.58 – 7.47 (m, 4H), 7.38 (d, *J* = 8.3 Hz, 2H), 5.19 (q, *J* = 6.7 Hz, 1H), 3.75 (dt, *J* = 7.2, 5.4 Hz, 1H), 3.68 (d, *J* = 2.1 Hz, 3H), 1.52 (dd, *J* = 7.1, 3.8 Hz, 3H), 1.46 (d, *J* = 6.7 Hz, 3H).

^{13}C NMR (101 MHz, $CDCl_3$) δ 174.9, 166.9, 146.3, 137.5, 137.4, 136.1, 132.1, 131.7, 128.4, 128.2, 128.2, 124.2, 123.4, 121.9, 56.9, 52.1, 44.9, 18.8, 18.5.

HRMS (ESI+, *m/z*) calculated for $C_{19}H_{19}NO_3$ $[M+Na]^+$: 332.1257; found: 332.1267;.

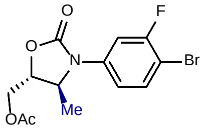

3g: colorless oil; yield 76%.

^{1}H NMR (400 MHz, $CDCl_3$) δ 7.55 (dd, *J* = 8.6, 7.8 Hz, 1H), 7.38 (dd, *J* = 10.3, 2.5 Hz, 1H), 7.14 – 7.07 (m, 1H), 4.39 (dd, *J* = 8.8, 4.9 Hz, 1H), 4.33 – 4.29 (m, 2H), 4.28 – 4.22 (m, 1H), 2.08 (s, 3H), 1.40 (d, *J* = 6.2 Hz, 3H).

^{13}C NMR (101 MHz, $CDCl_3$) δ 170.4, 160.2 (d, *J* = 248.5 Hz), 153.8, 137.15 (d, *J* = 9.6 Hz), 133.6, 117.27 (d, *J* = 3.3 Hz), 109.6 (d, *J* = 26.3 Hz), 104.5 (d, *J* = 21.2 Hz), 77.5, 63.4, 54.0, 20.6, 18.4.

^{19}F NMR (376 MHz, $CDCl_3$) δ -104.56 (dd, *J* = 10.5, 7.7 Hz).

HRMS (ESI+, *m/z*) calculated for $C_{13}H_{13}NO_4FBr$ $[M+H]^+$: 346.0085; found: 346.0087;.

C(sp3)–H methylation via a one-pot procedure: In a dried Schlenk tube, N-heterocycle **1** (0.2 mmol), NFSI (0.3 mmol), ligand (0.024 mmol, 12 mol%) and CuOAc (0.02 mmol, 10 mol%) were dissolved in CH_3CN (1.0 mL) under a N_2 atmosphere, then H_2O (0.3 mmol) were added. The reaction mixture was stirred at 20 ^oC. After NFSI consumption, the solvent was evaporated. Then dry DCM (1.0 mL) and DAST (0.2 mmol) was added to the mixture at -78 ^oC under nitrogen and the solution was stirred for an hour at rt. The reaction was then placed back into -78 °C cold bath, where $AlMe_3$ (0.6 mmol) was then added dropwise. The reaction mixture was stirred at -78 °C for 2 h, then allowed to warm to room temperature while stirring for 1 h. The desired product was purified by column chromatography on silica gel with a gradient eluent of petroleum ether and ethyl acetate to provide the desired product.

RMgBr as the nucleophile: in a dried Schlenk tube, the C-H hydroxylated products **2** (0.1 mmol) was dissolved in 1 mL of DCM under nitrogen and cooled to -78 ^oC. DAST (0.1 mmol) was added and the solution was stirred for an hour at rt. The reaction was then placed back into -78 °C cold bath, where nucleophilic reagent (0.3 mmol) was then added dropwise. The reaction mixture was stirred at -78 °C for 5 h, then allowed to warm to room temperature while stirring for 1 h. Upon completion, the reaction was added 10 mL H_2O for quenching. The aqueous layer was extracted

with DCM (2x10 mL). The organic layers were combined, dried over anhydrous Na_2SO_4, and concentrated in vacuo. The crude product was purified by column chromatography (eluting with petroleum ether/ethyl acetate 10% - 30%).

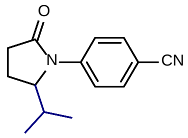

3h: colorless oil; yield 93%.

^{1}H NMR (400 MHz, $CDCl_3$) δ 7.67 (d, *J* = 8.7 Hz, 2H), 7.59 (d, *J* = 8.7 Hz, 2H), 4.32 (dt, *J* = 8.5, 4.2 Hz, 1H), 2.69 – 2.47 (m, 2H), 2.18 – 2.11 (m, 1H), 2.11 – 2.01 (m, 1H), 1.98 – 1.88 (m, 1H), 0.94 (d, *J* = 7.0 Hz, 3H), 0.72 (d, *J* = 6.8 Hz, 3H).

^{13}C NMR (126 MHz, $CDCl_3$) δ 174.8, 141.8, 132.9, 123.4, 118.6, 108.3, 63.2, 31.7, 28.3, 18.4, 17.4, 14.2.

HRMS (ESI+, *m/z*) calculated for $C_{14}H_{16}N_2O$ $[M+H]^+$: 229.1335; found: 229.1333;.

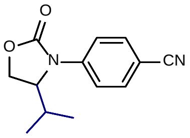

3i: colorless oil; yield 53%.

^{1}H NMR (400 MHz, $CDCl_3$) δ 7.72 – 7.61 (m, 4H), 4.52 – 4.41 (m, 2H), 4.30 (dd, *J* = 7.9, 3.0 Hz, 1H), 2.25 - 217 (m, 1H), 0.96 (d, *J* = 7.0 Hz, 3H), 0.85 (d, *J* = 6.8 Hz, 3H).

^{13}C NMR (126 MHz, $CDCl_3$) δ 155.0, 141.0, 133.2, 120.7, 118.5, 107.7, 62.4, 59.7, 27.4, 17.7, 14.0.

HRMS (ESI+, *m/z*) calculated for $C_{13}H_{14}N_2O_2$ $[M+H]^+$: 231.1128; found: 231.1127;.

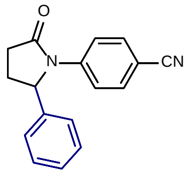

3j: colorless oil; yield 78%.

^{1}H NMR (400 MHz, $CDCl_3$) δ 7.63 (d, *J* = 8.7 Hz, 2H), 7.51 (d, *J* = 8.8 Hz, 2H), 7.36 – 7.27 (m, 3H), 7.19 – 7.17 (m, 2H), 5.28 (dd, *J* = 6.9, 3.9 Hz, 1H), 2.81 – 2.73 (m, 1H), 2.72 – 2.58 (m, 2H), 2.09 – 2.00 (m, 1H).

^{13}C NMR (126 MHz, $CDCl_3$) δ 175.2, 142.2, 140.2, 132.7, 129.3, 128.1, 125.5, 121.1, 118.7, 107.4, 63.3, 31.1, 29.0.

HRMS (ESI+, *m/z*) calculated for $C_{17}H_{14}N_2O$ $[M+H]^+$: 263.1179; found: 263.1179;.

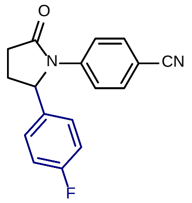

3k: colorless oil; yield 88%.

^{1}H NMR (400 MHz, $CDCl_3$) δ 7.59 (d, *J* = 8.8 Hz, 2H), 7.51 (d, *J* = 8.8 Hz, 2H), 7.16 (dd, *J* = 8.4, 5.2 Hz, 2H), 7.01 (t, *J* = 8.5 Hz, 2H), 5.32 – 5.22 (m, 1H), 2.79 – 2.58 (m, 3H), 2.04 – 1.96 (m, 1H).

^{13}C NMR (126 MHz, $CDCl_3$) δ 175.0, 162.2 (d, *J* = 248.2 Hz), 141.9, 135.9 (d, *J* = 3.2 Hz), 132.6, 127.20 (d, *J* = 8.2 Hz), 121.2, 118.6, 116.2 (d, *J* = 21.4 Hz), 107.4, 62.5, 31.0, 29.0.

^{19}F NMR (376 MHz, $CDCl_3$) δ -113.52 (ddd, *J* = 13.6, 8.4, 5.0 Hz).

HRMS (ESI+, *m/z*) calculated for $C_{17}H_{13}FN_2O$ $[M+H]^+$: 281.1085; found: 281.1086;.

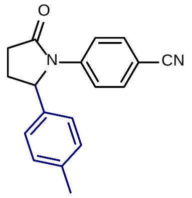

3m: colorless oil; yield 57%.

^{1}H NMR (400 MHz, $CDCl_3$) δ 7.62 (d, *J* = 8.8 Hz, 2H), 7.51 (d, *J* = 8.8 Hz, 2H), 7.13 (d, *J* = 7.9 Hz, 2H), 7.06 (d, *J* = 8.0 Hz, 2H), 5.25 – 5.22 (m, 1H), 2.81 – 2.57 (m, 3H), 2.05 – 1.99 (m, 1H).

^{13}C NMR (101 MHz, $CDCl_3$) δ 175.3, 142.3, 137.9, 137.2, 132.7, 129.9, 125.4, 121.2, 118.7, 107.3, 63.2, 31.2, 29.2, 21.0.

HRMS (ESI+, *m/z*) calculated for $C_{18}H_{16}N_2O$ $[M+H]^+$: 277.1335; found: 277.1335;.

Bipyridine (bpy)/RMgBr complex as the nucleophile[6]: to a dried Schlenk tube, bpy (0.4 mmol), THF (0.5 mL), RMgBr (0.4 mmol) were added under nitrogen at -40 °C. The reaction was stirred at -40 °C for 0.5 h and rt for 0.5 h. In another dried sealed tube, the C-H hydroxylated product **2** (0.1 mmol) was dissolved in 1.0 mL of DCM under nitrogen and cooled to -78 °C. DAST (0.1 mmol) was added and the solution was stirred for an hour at rt. The DAST reaction solution was then added dropwise to the the tube of (bpy)/RMgBr. The reaction mixture was stirred at -40 °C for 5 h, then allowed to warm to room temperature while stirring for 1 h. Upon completion, the reaction was added 10 mL H_2O for quenching. The aqueous layer was extracted with DCM (2x10 mL). The

organic layers were combined, dried over anhydrous Na_2SO_4, and concentrated in vacuo. The crude product was purified by column chromatography (eluting with petroleum ether/ethyl acetate 10 % - 30 %).

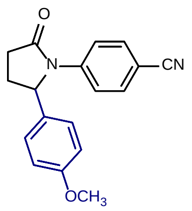

3l: white solid; yield 67%.

^{1}H NMR (400 MHz, $CDCl_3$) δ 7.68 – 7.58 (m, 2H), 7.56 – 7.47 (m, 2H), 7.09 (d, *J* = 8.6 Hz, 2H), 6.84 (d, *J* = 8.7 Hz, 2H), 5.23 (dd, *J* = 7.4, 4.4 Hz, 1H), 3.77 (s, 3H), 2.83 – 2.68 (m, 1H), 2.68 – 2.54 (m, 2H), 2.07 – 1.94 (m, 1H).

^{13}C NMR (101 MHz, $CDCl_3$) δ 175.2, 159.3, 142.2, 132.7, 132.0, 126.8, 121.3, 118.7, 114.6, 107.4, 62.9, 55.3, 31.2, 29.2.

HRMS (ESI+, *m/z*) calculated for $C_{18}H_{16}N_2O_2$ $[M+H]^+$: 293.1285; found: 293.1285;.

CuBr.Me$_2$S/RMgBr as the nucleophile complex[7]: in a dried Schlenk tube, CuBr.Me_2S (0.4 mmol), THF (0.5 mL), RMgBr (0.4 mmol) added at -40 °C for 0.5 h; rt for 0.5 h. In another dried sealed tube, the the C-H hydroxylated product **2** (0.1 mmol) was dissolved in 1 mL of DCM under nitrogen and cooled to -78 °C. DAST (0.1 mmol) was added and the solution was stirred for an hour at rt. The DAST reaction solution was then added dropwise to the solution of CuBr.Me_2S/RMgBr reaction. The reaction mixture was stirred at -40 °C for 5 h, then allowed to warm to room temperature while stirring for 1 h. Upon completion, the reaction was added 10 mL H_2O for quenching. The aqueous layer was extracted with DCM (2x10 mL). The organic layers were combined, dried over anhydrous Na_2SO_4, and concentrated in vacuo. The crude product was purified by column chromatography (eluting with petroleum ether/ethyl acetate 10%-30%).

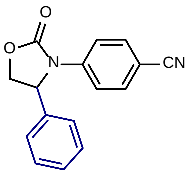

3n: colorless oil; yield 91%.

^1H NMR (400 MHz, $CDCl_3$) δ 7.61 - 7.55 (m, 4H), 7.46 – 7.37 (m, 3H), 7.34 – 7.26 (m, 2H), 5.43 (dd, J = 8.6, 5.6 Hz, 1H), 4.84 (t, J = 8.7 Hz, 1H), 4.26 (dd, J = 8.3, 5.9 Hz, 1H).

^{13}C NMR (101 MHz, $CDCl_3$) δ 155.1, 141.1, 137.3, 133.0, 129.7, 129.3, 125.8, 119.8, 118.5, 107.4, 69.8, 60.1.

HRMS (ESI+, *m/z*) calculated for $C_{16}H_{12}N_2O_2$ $[M+H]^+$: 265.0972; found: 265.0971;.

Synthetic applications of hydroxylated pyrrolidines 2s: in a dried Schlenk tube, **2s** (0.1 mmol) was dissolved in 1.0 mL of DCM under nitrogen and cooled to -78 °C. $BF_3·OEt_2$ (0.2 mmol) and nucleophilic reagent (0.3 mmol) was added and the solution was stirred for an hour. The reaction was allowed to warm to room temperature while stirring for 3 h. Upon completion, the reaction was added 10 mL H_2O for quenching. The aqueous layer was extracted with DCM (2x10 mL). The organic layers were combined, dried over anhydrous Na_2SO_4, and concentrated in vacuo. The crude product was purified by column chromatography (eluting with petroleum ether/ethyl acetate 10 % - 30 %).

2s with 1,2-dimethylindole as the nucleophile: in a dried Schlenk tube, the **2s** (0.1 mmol) was dissolved in 1.0 mL of DCM under nitrogen and cooled to -78 °C. $BF_3·OEt_2$ (0.2 mmol) and nucleophilic reagent (0.3 mmol) was added and the solution was stirred at -78 °C for 3 h. Upon completion, the reaction was added 10 mL H_2O for quenching. The aqueous layer was extracted with DCM (2x10 mL). The organic layers were combined, dried over anhydrous Na_2SO_4, and concentrated in vacuo. The crude product was purified by column chromatography (eluting with petroleum ether/ethyl acetate 10 % - 30 %).

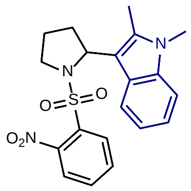

3o: yellow solid; yield 59%.

^1H NMR (400 MHz, $CDCl_3$) δ 7.32 – 7.24 (m, 1H), 7.22 – 7.16 (m, 1H), 7.09 – 7.03 (m, 1H), 7.02 – 6.97 (m, 1H), 6.97 – 6.91 (m, 1H), 6.90 – 6.85 (m, 1H), 6.79 – 6.66 (m, 2H), 5.23 – 5.16 (m, 1H), 4.20 – 4.12 (m, 1H), 4.02 – 3.92 (m, 1H), 3.58 (s, 3H), 2.42 (s, 3H), 2.41 – 2.26 (m, 2H), 2.24– 2.15 (m, 1H), 2.15 – 2.02 (m, 1H).

^{13}C NMR (126 MHz, $CDCl_3$) δ 146.7, 136.5, 135.0, 133.6, 131.5, 129.7, 129.5, 125.5, 122.9, 120.6, 118.9, 118.5, 109.0, 108.3, 57.4, 49.9, 34.2, 29.3, 25.7, 10.1.

HRMS (ESI+, *m/z*) calculated for $C_{20}H_{21}N_3O_4S$ $[M + H]^+$: 400.1326; found: 400.1327;.

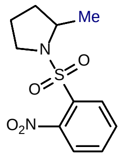

3p: yellow liquid; yield 90%.

^1H NMR (400 MHz, $CDCl_3$) δ 8.09 – 7.94 (m, 1H), 7.71 – 7.63 (m, 2H), 7.62 – 7.53 (m, 1H), 4.14 – 3.95 (m, 1H), 3.55 – 3.33 (m, 2H), 2.04 – 1.88 (m, 2H), 1.86 – 1.71 (m, 1H), 1.64 – 1.54 (m, 1H), 1.24 (d, J = 6.4 Hz, 3H).

^{13}C NMR (126 MHz, $CDCl_3$) δ 148.3, 133.4, 132.5, 131.4, 130.5, 123.8, 56.5, 48.8, 33.7, 24.0, 22.0.

HRMS (ESI+, *m/z*) calculated for $C_{11}H_{14}N_2O_4S$ $[M + H]^+$: 271.0747; found: 271.0748;.

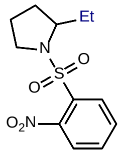

3q: yellow liquid; yield 78%.

^1H NMR (400 MHz, $CDCl_3$) δ 8.04 – 7.96 (m, 1H), 7.73 – 7.62 (m, 2H), 7.62 – 7.54 (m, 1H), 3.90 – 3.81 (m, 1H), 3.51 – 3.37 (m, 2H), 1.96 – 1.83 (m, 2H), 1.83 – 1.64 (m, 3H), 1.52 – 1.39 (m, 1H), 0.87 (t, J = 7.4 Hz, 3H).

^{13}C NMR (126 MHz, $CDCl_3$) δ 148.4, 133.4, 132.4, 131.3, 130.6, 123.8, 62.2, 48.9, 30.3, 28.4, 24.2, 10.2.

HRMS (ESI+, *m/z*) calculated for C12$H_{16}N_2O_4S$ $[M + H]^+$: 285.0904; found: 285.0909;.

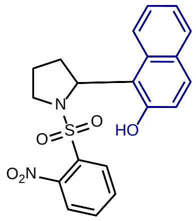

3r: yellow solid; yield 71%.

^1H NMR (400 MHz, DMSO) δ 9.83 (s, 1H), 8.00 (d, J = 8.8 Hz, 1H), 7.72 (dd, J = 16.8, 8.0 Hz, 2H), 7.64 (d, J = 8.8 Hz, 1H), 7.59 (t, J = 7.8 Hz, 1H), 7.38 – 7.13 (m, 3H), 7.03 (dd, J = 14.4, 8.4

Hz, 2H), 5.82 (t, J = 8.6 Hz, 1H), 3.86 (dd, J = 10.2, 4.1 Hz, 2H), 2.35– 2.19 (m, 2H), 2.17 – 2.07 (m, 1H), 1.96 – 1.81 (m, 1H).

^{13}C NMR (101 MHz, DMSO) δ 153.3, 147.2, 133.5, 132.1, 131.4, 131.0, 129.4, 129.2, 128.4, 128.2, 125.9, 123.6, 122.4, 122.1, 118.1, 116.2, 56.9, 49.5, 32.5, 25.7.

HRMS (ESI+, *m/z*) calculated for $C_{20}H_{18}N_2O_6S$ [M + H]$^+$: 437.0778; found: 437.0777;.

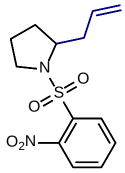

3s: yellow liquid; yield 85%.

^{1}H NMR (400 MHz, $CDCl_3$) δ 8.06 – 7.96 (m, 1H), 7.73 – 7.63 (m, 2H), 7.63 – 7.55 (m, 1H), 5.79 – 5.67(m, 1H), 5.14 – 4.98 (m, 2H), 4.05 – 3.96 (m, 1H), 3.45 (t, J = 6.4 Hz, 2H), 2.56 – 2.46 (m, 1H), 2.31 – 2.19 (m, 1H), 1.98 – 1.83 (m, 2H), 1.82 – 1.72 (m, 2H).

^{13}C NMR (126 MHz, $CDCl_3$) δ 148.4, 134.0, 133.5, 132.2, 131.4, 130.7, 123.9, 117.9, 60.0, 49.1, 40.0, 30.3, 24.0.

HRMS (ESI+, *m/z*) calculated for $C_{13}H_{16}N_2O_4S$ [M + H]$^+$: 297.0904; found: 297.0908;.

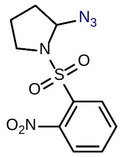

3t: yellow liquid; yield 76%.

^{1}H NMR (400 MHz, $CDCl_3$) δ 8.19 – 8.05 (m, 1H), 7.78 – 7.69 (m, 2H), 7.69 – 7.64 (m, 1H), 5.63 – 5.51 (m, 1H), 3.65 (td, J = 8.8, 8.1, 2.6 Hz, 1H), 3.32 (td, J = 8.9, 8.4, 5.3 Hz, 1H), 2.18 – 1.95 (m, 4H).

^{13}C NMR (101 MHz, $CDCl_3$) δ 148.2, 134.0, 132.1, 131.8, 131.3, 124.3, 77.0, 47.7, 33.4, 23.4.

HRMS (ESI+, *m/z*) calculated for $C_{10}H_{11}N_5O_4S$ [M+Na]$^+$: 320.0424; found: 320.0434;.

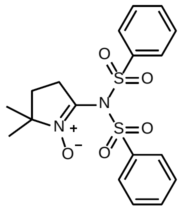

4: yellow liquid; yield 82%.

^{1}H NMR (400 MHz, $CDCl_3$) δ 7.97 – 7.84 (m, 1H), 7.80 – 7.65 (m, 2H), 7.60 – 7.52 (m, 1H), 7.52 – 7.43 (m, 2H), 7.01 – 6.88 (m, 2H), 4.78 (s, 2H), 3.81 (s, 3H).

^{13}C NMR (101 MHz, $CDCl_3$) δ 167.5, 141.7, 134.7, 134.5, 132.2, 129.3, 128.8, 128.6, 126.8, 65.6, 31.6, 25.7, 24.5.

HRMS (ESI+, *m/z*) calculated for $C_{18}H_{21}N_2O_5S_2$ $[M+H]^+$: 409.0892; found: 409.0891;.

7. NMR Spectra

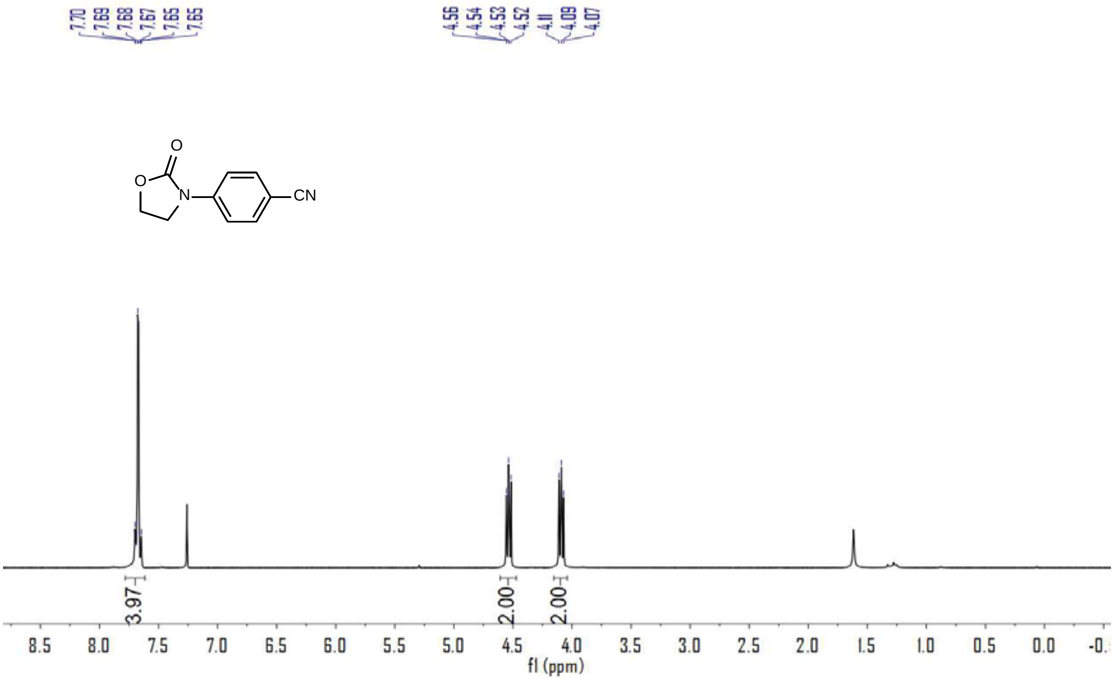

Supplementary Fig. 6. ^{1}**H NMR spectrum of compound 1a.**

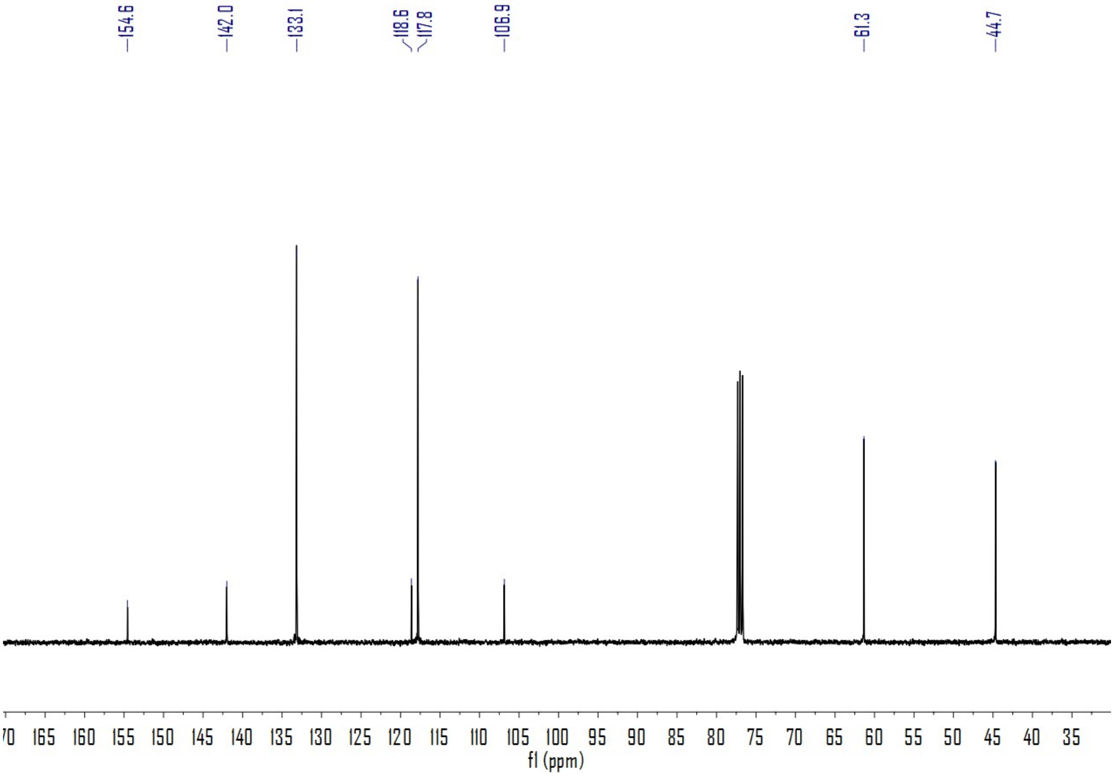

Supplementary Fig. 7. ^{13}**C NMR spectrum of compound 1a.**

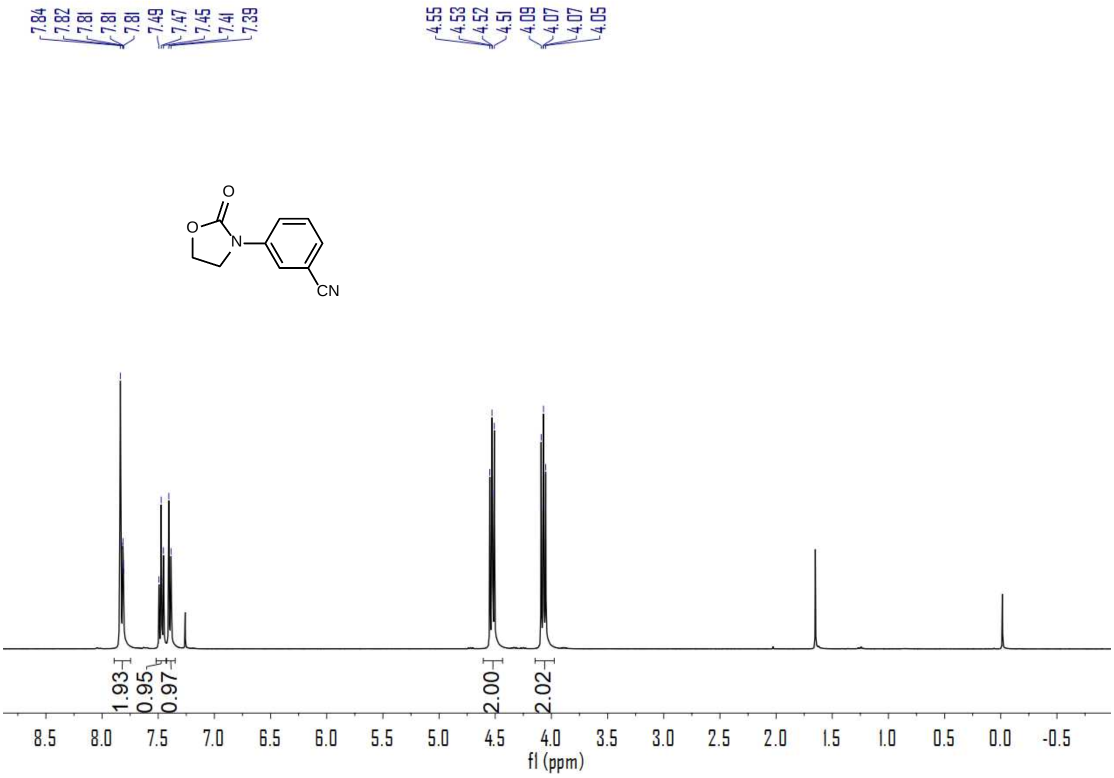

Supplementary Fig. 8. ^{1}H NMR spectrum of compound 1b.

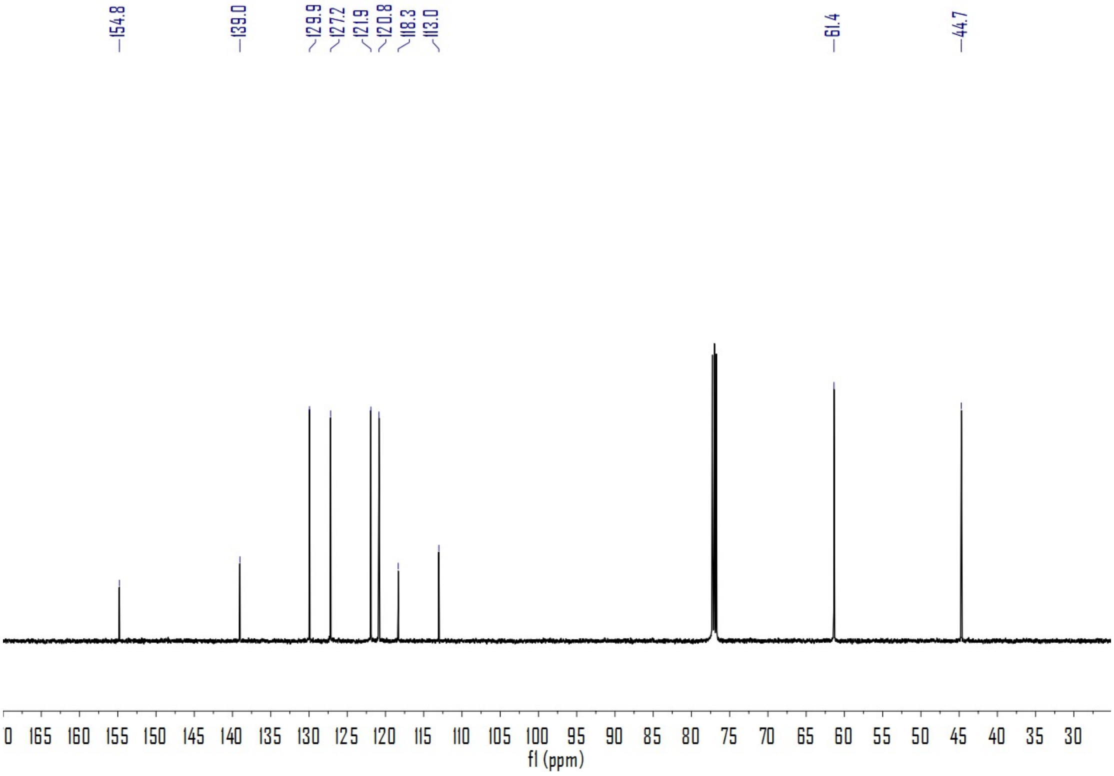

Supplementary Fig. 9. ^{13}C NMR spectrum of compound 1b.

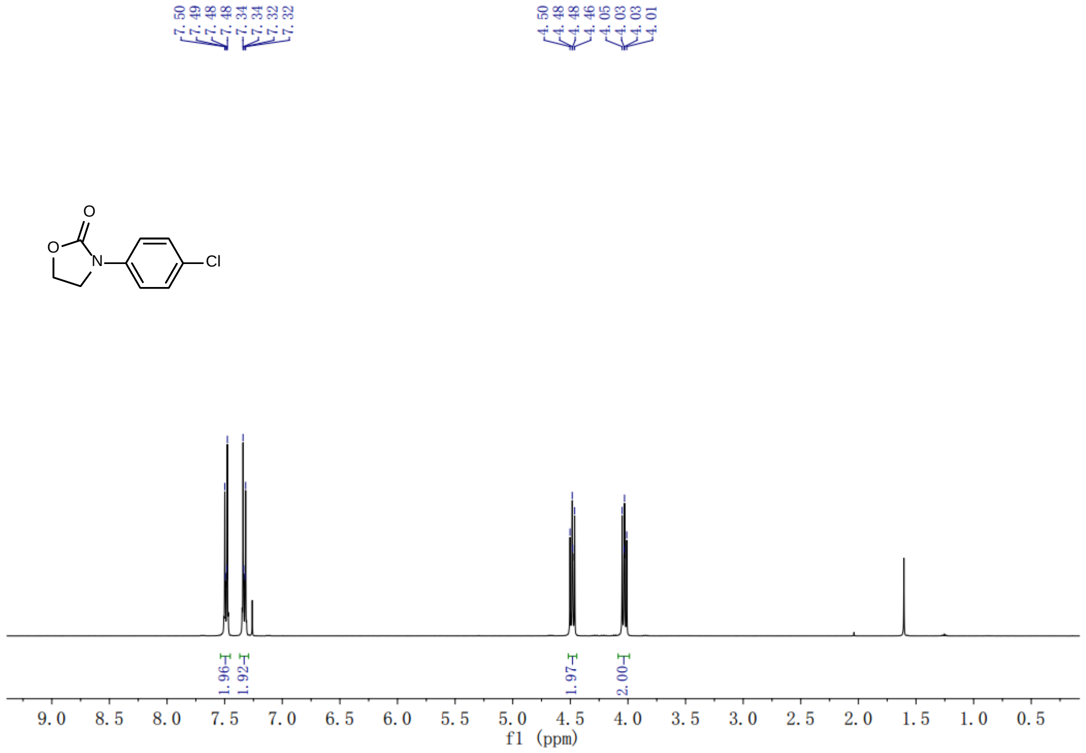

Supplementary Fig. 10. ^{1}H NMR spectrum of compound 1c.

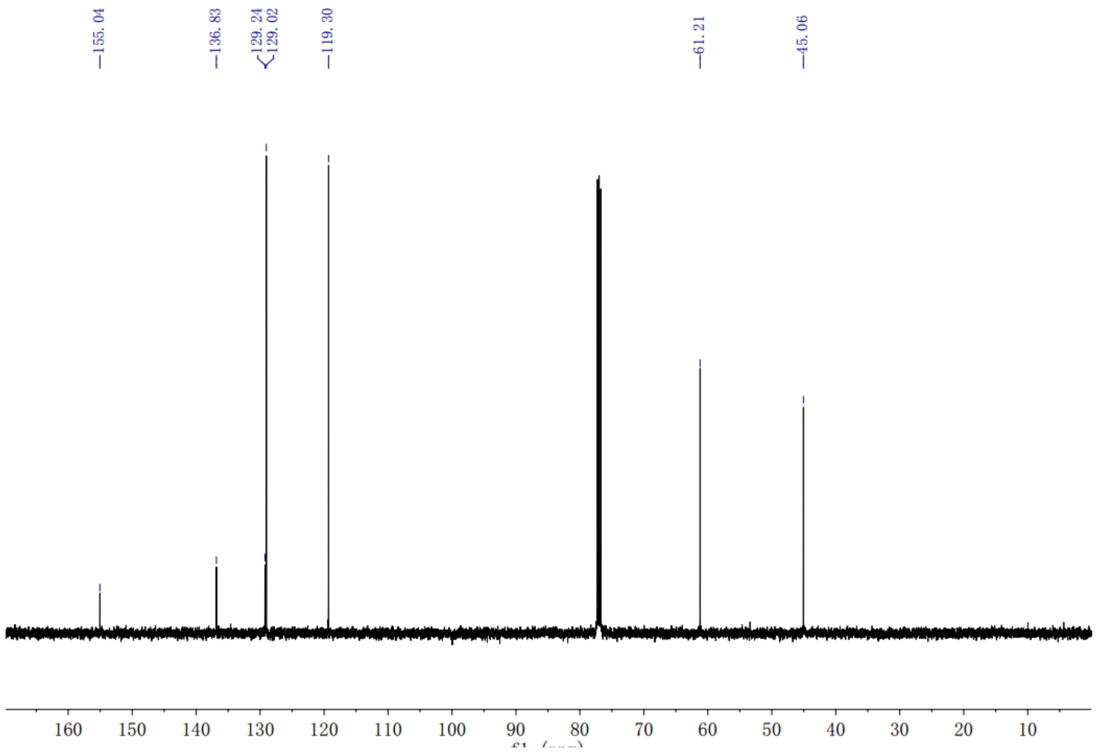

Supplementary Fig. 11. ^{13}C NMR spectrum of compound 1c.

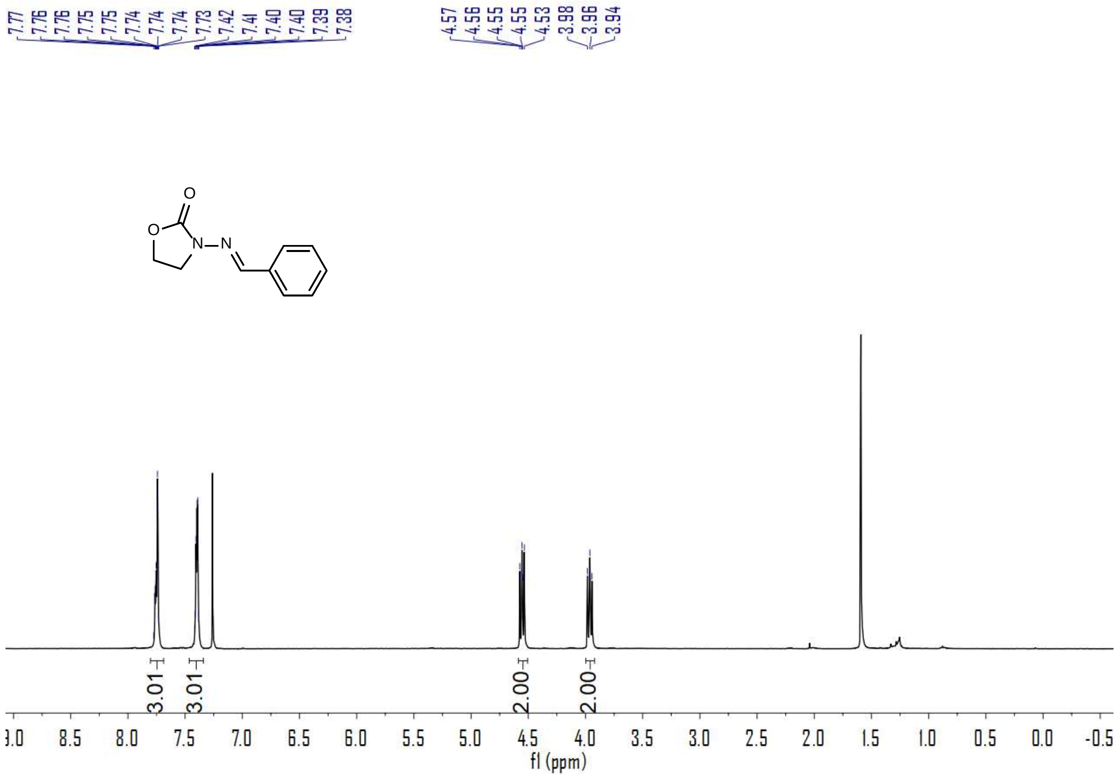

Supplementary Fig. 12. ^{1}H NMR spectrum of compound 1d.

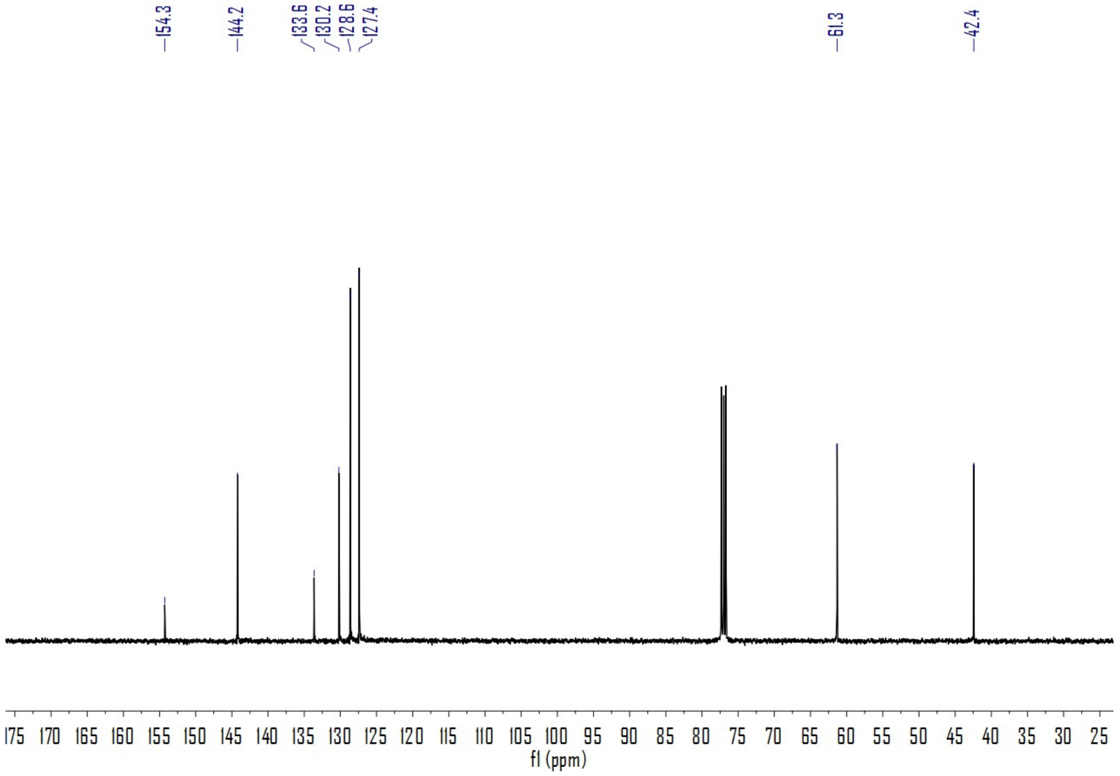

Supplementary Fig. 13. ^{13}C NMR spectrum of compound 1d.

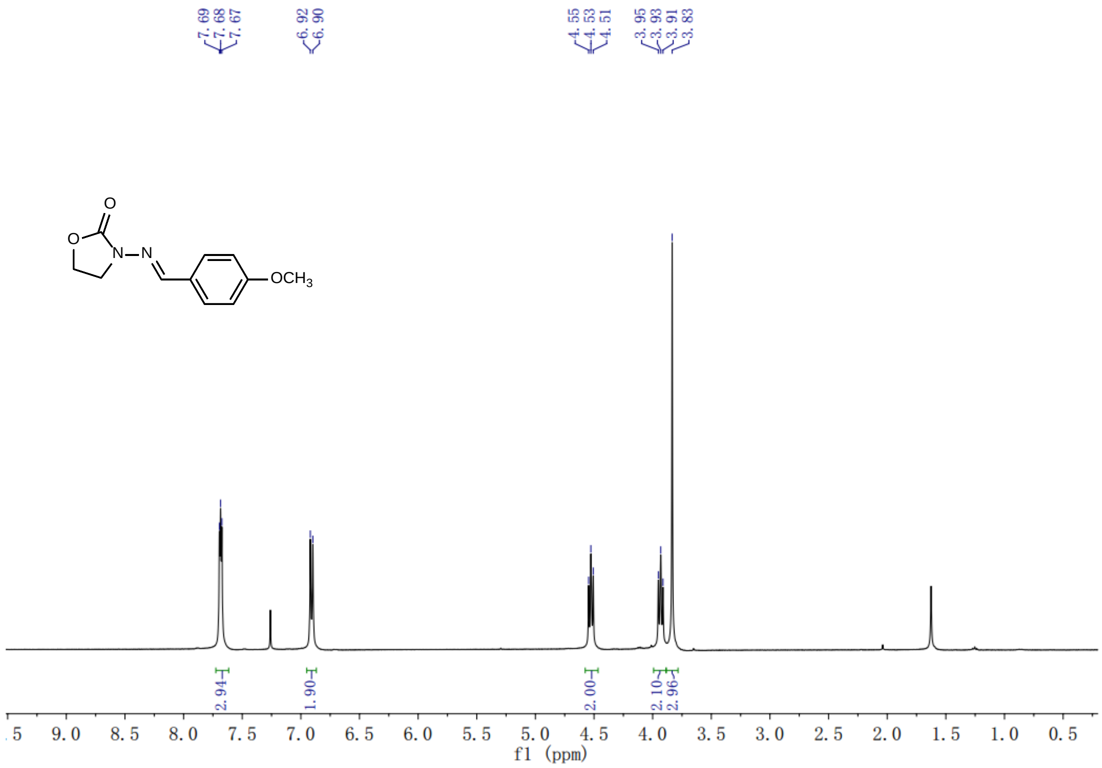

Supplementary Fig. 14. ^{1}H NMR spectrum of compound 1e.

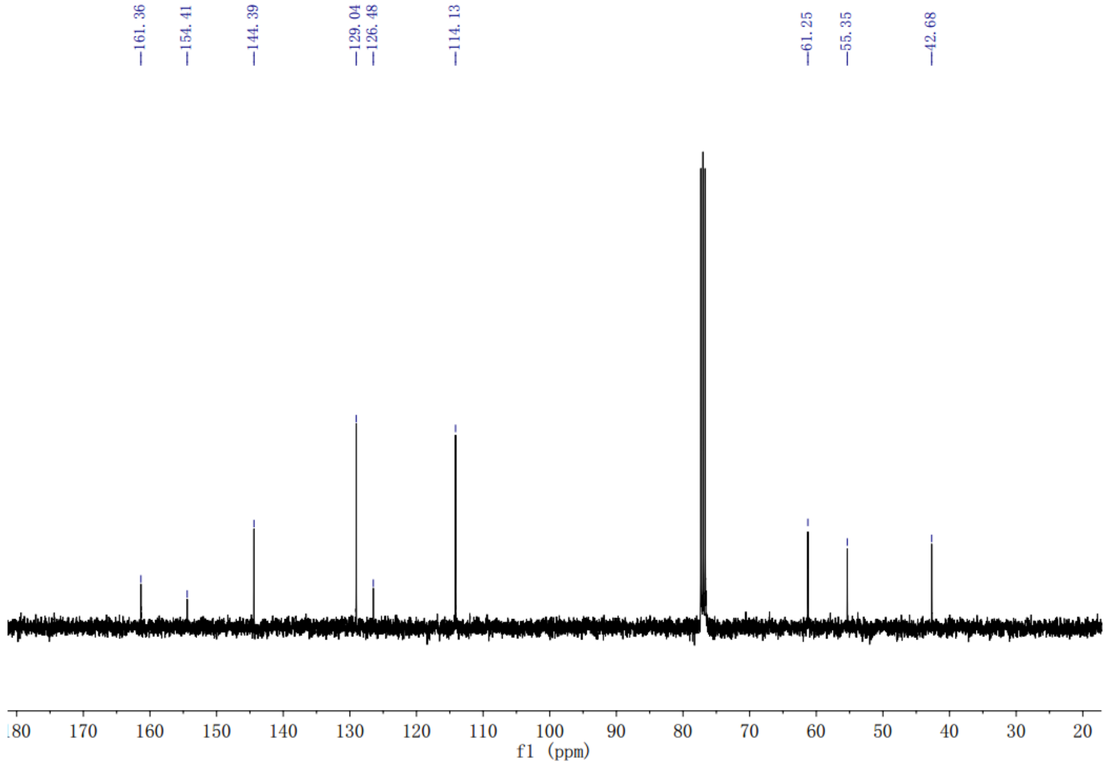

Supplementary Fig. 15. ^{13}C NMR spectrum of compound 1e.

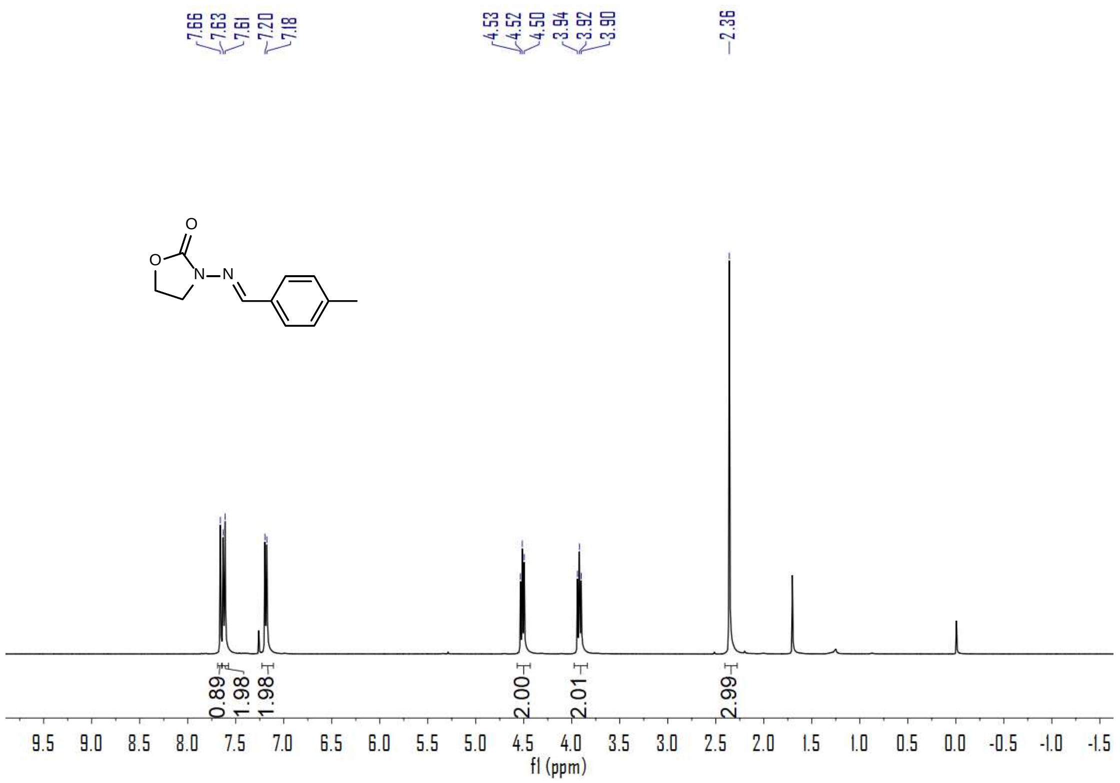

Supplementary Fig. 16. ^{1}H NMR spectrum of compound 1f.

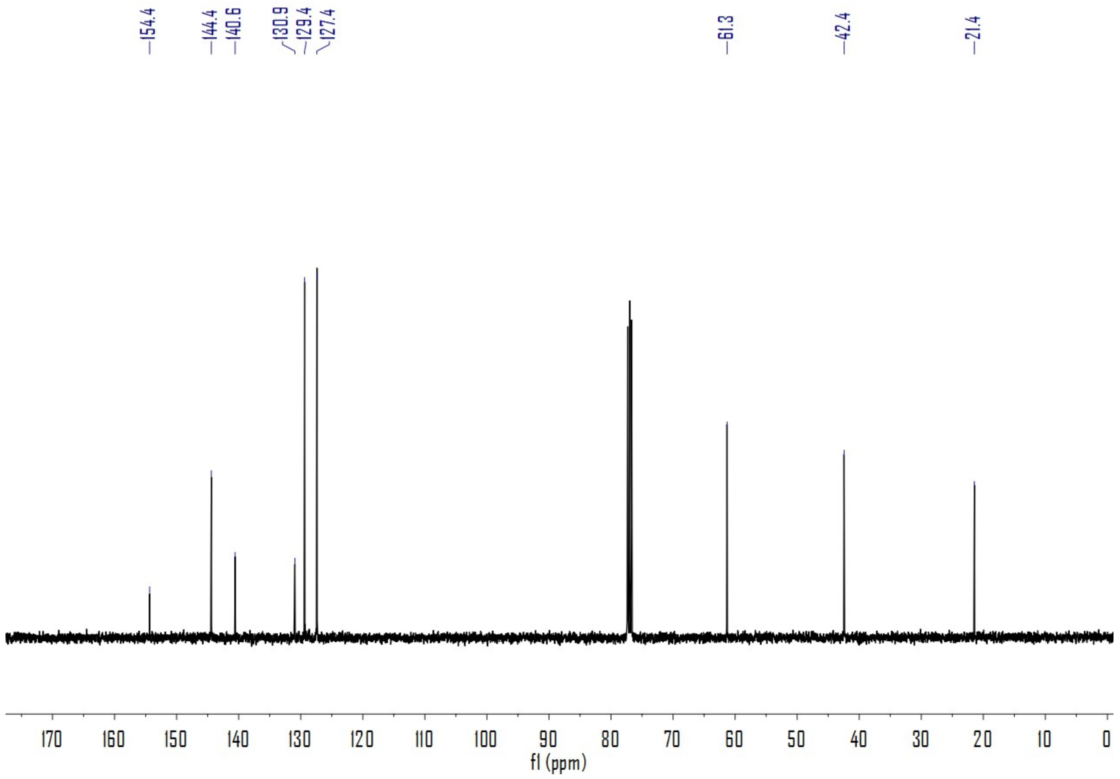

Supplementary Fig. 17. ^{13}C NMR spectrum of compound 1f.

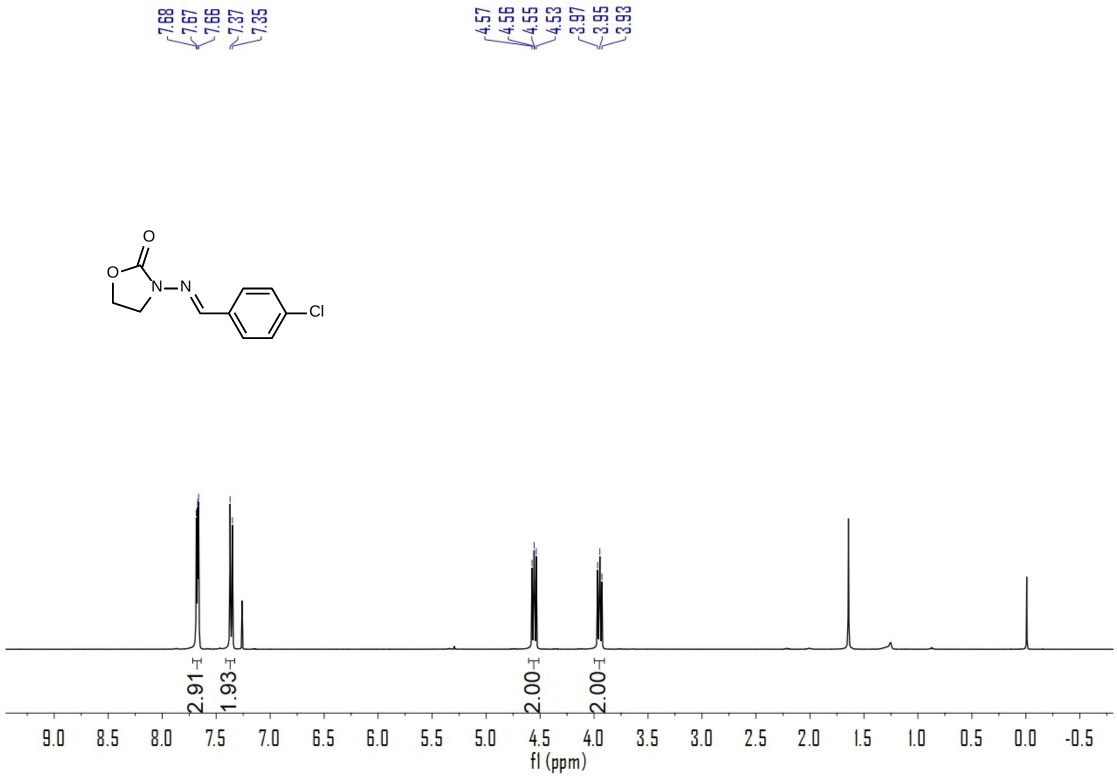

Supplementary Fig. 18. ^{1}H NMR spectrum of compound 1g.

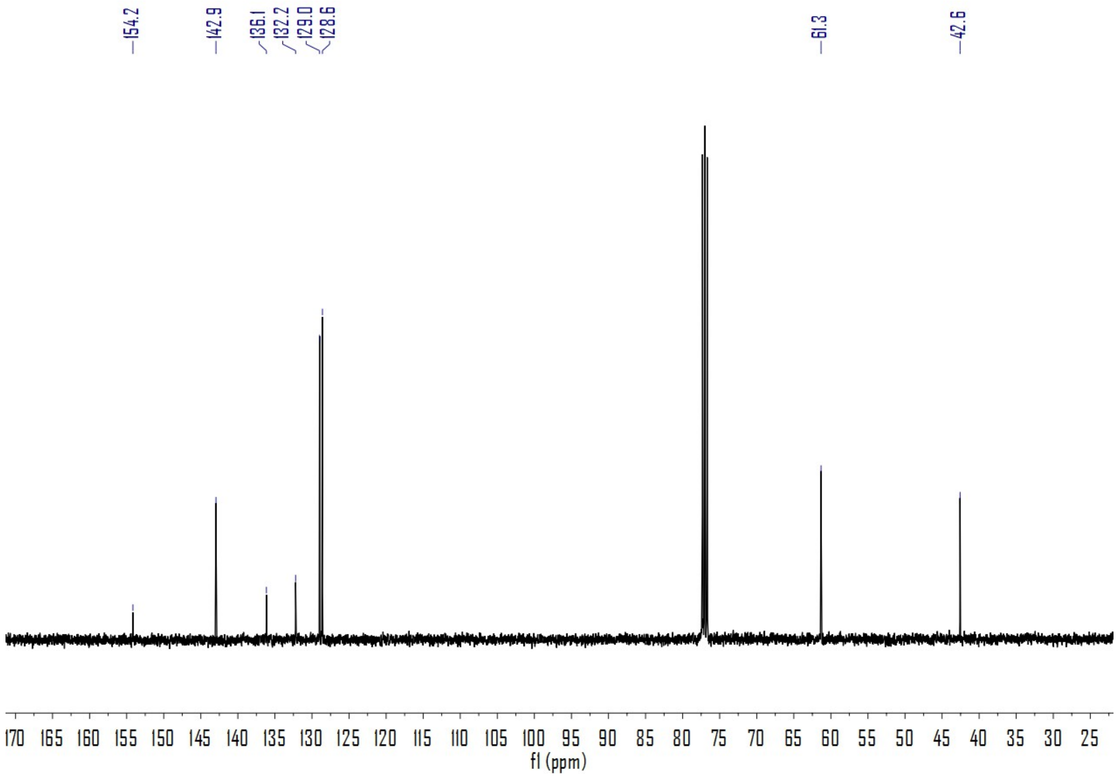

Supplementary Fig. 19. ^{13}C NMR spectrum of compound 1g.

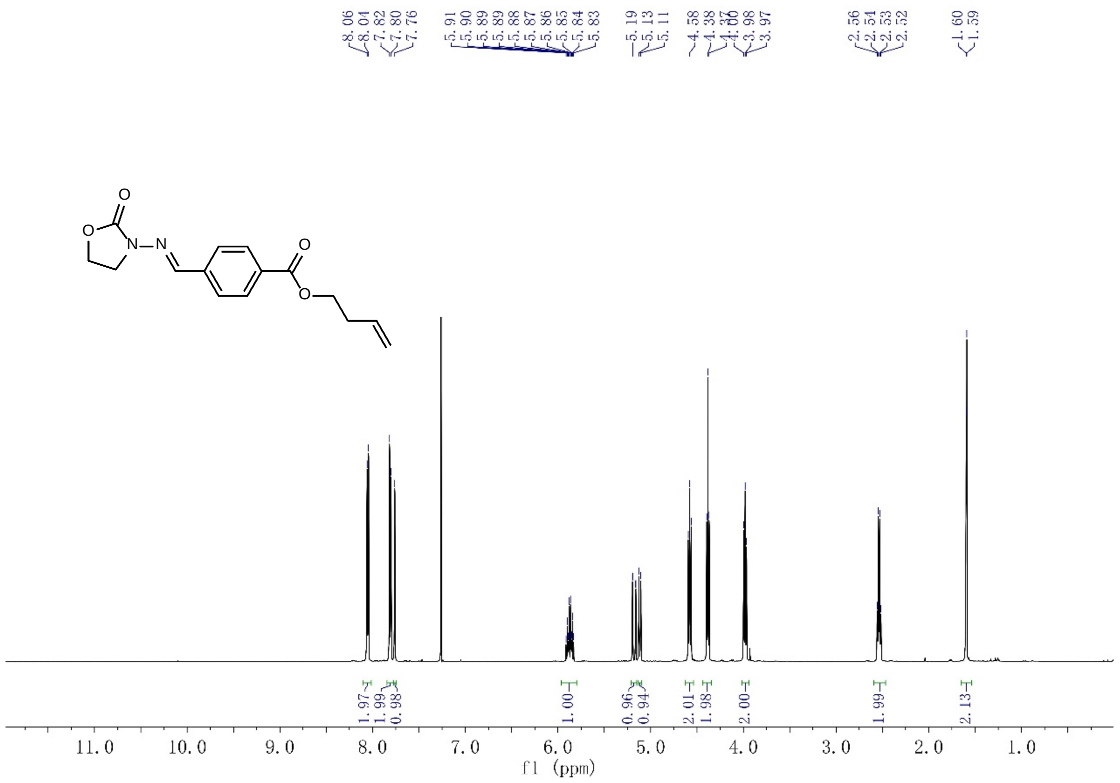

Supplementary Fig. 20. ^{1}H NMR spectrum of compound 1h.

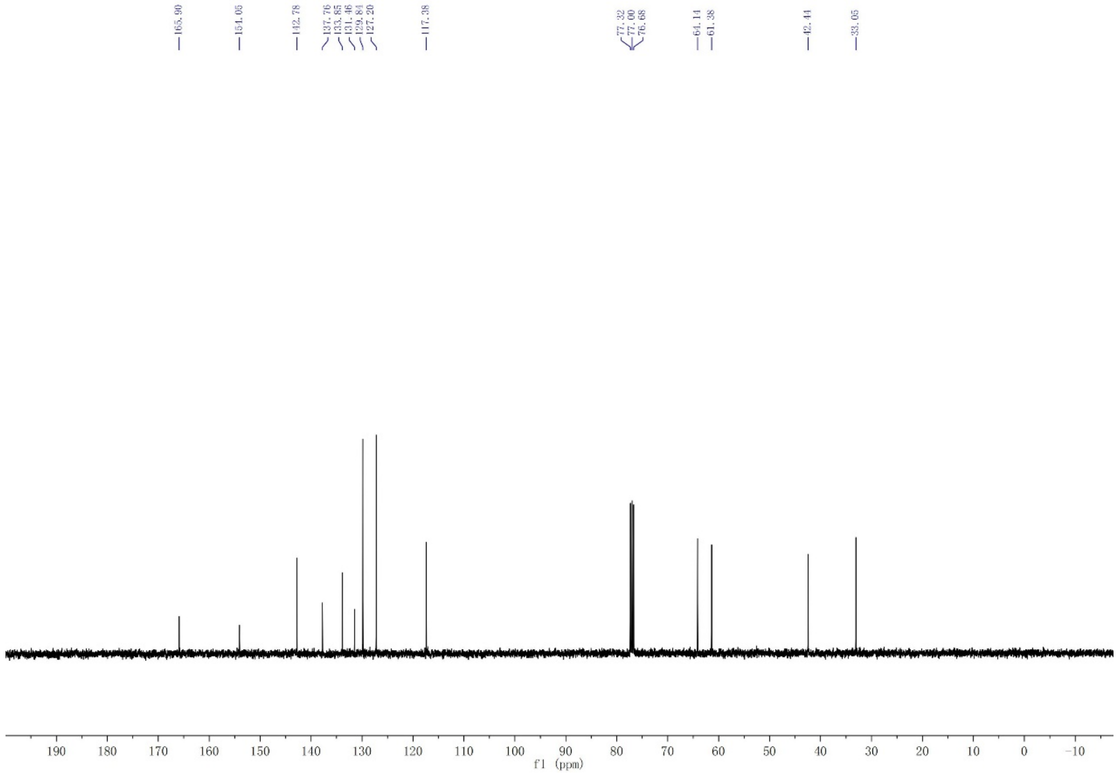

Supplementary Fig. 21. ^{13}C NMR spectrum of compound 1h.

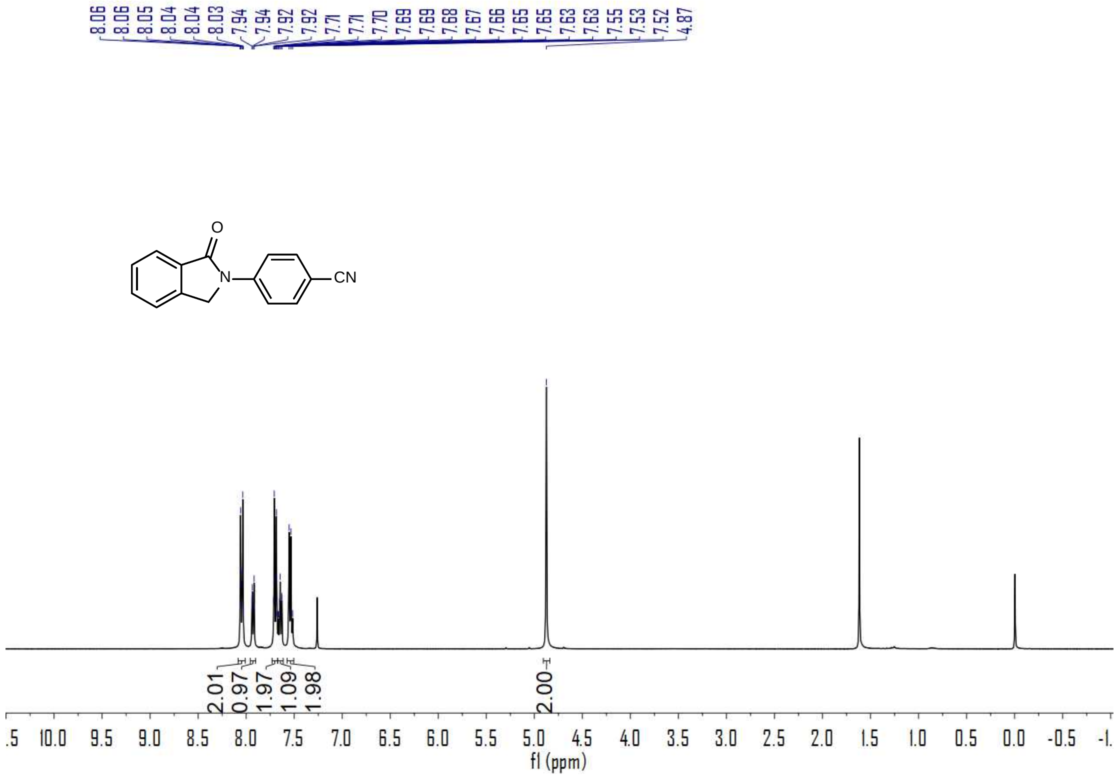

Supplementary Fig. 22. ^{1}H NMR spectrum of compound 1i.

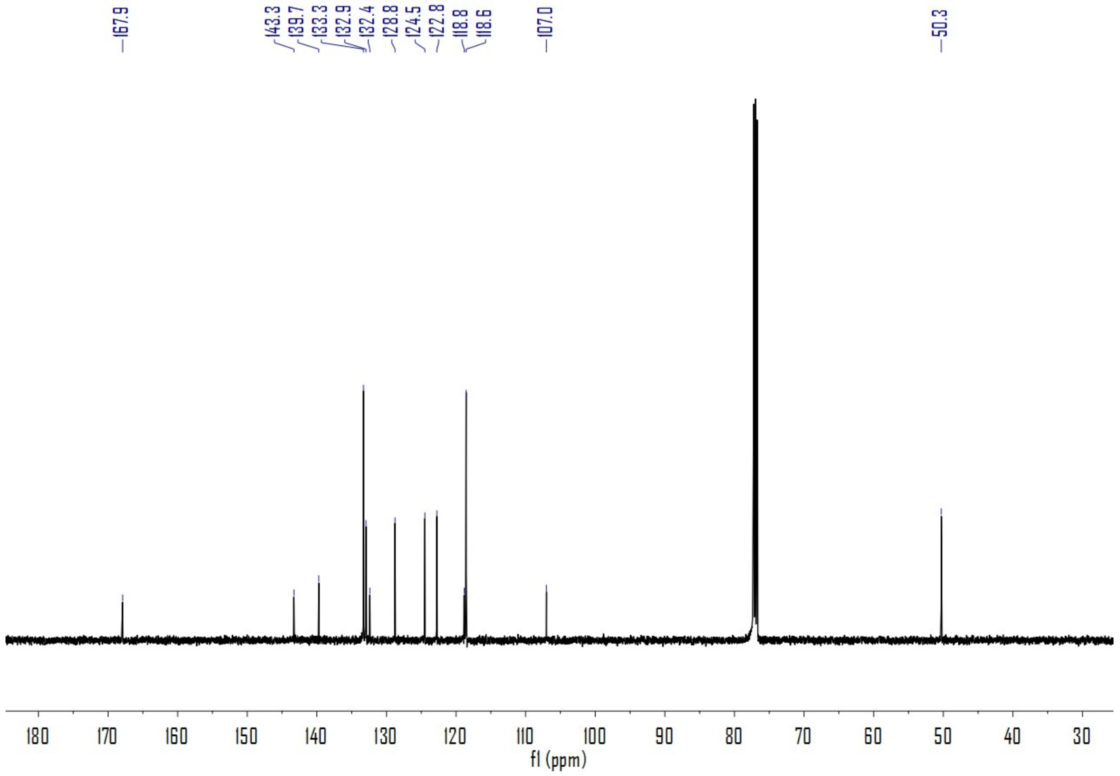

Supplementary Fig. 23. ^{13}C NMR spectrum of compound 1i.

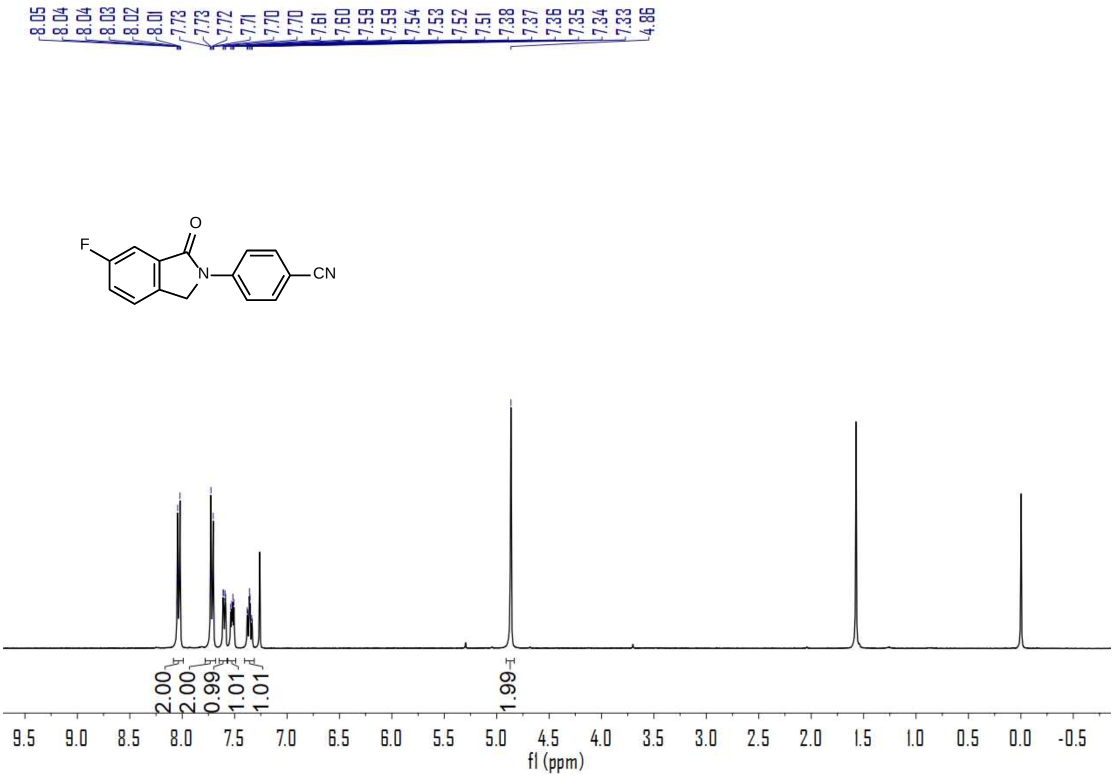

Supplementary Fig. 24. ^{1}H NMR spectrum of compound 1j.

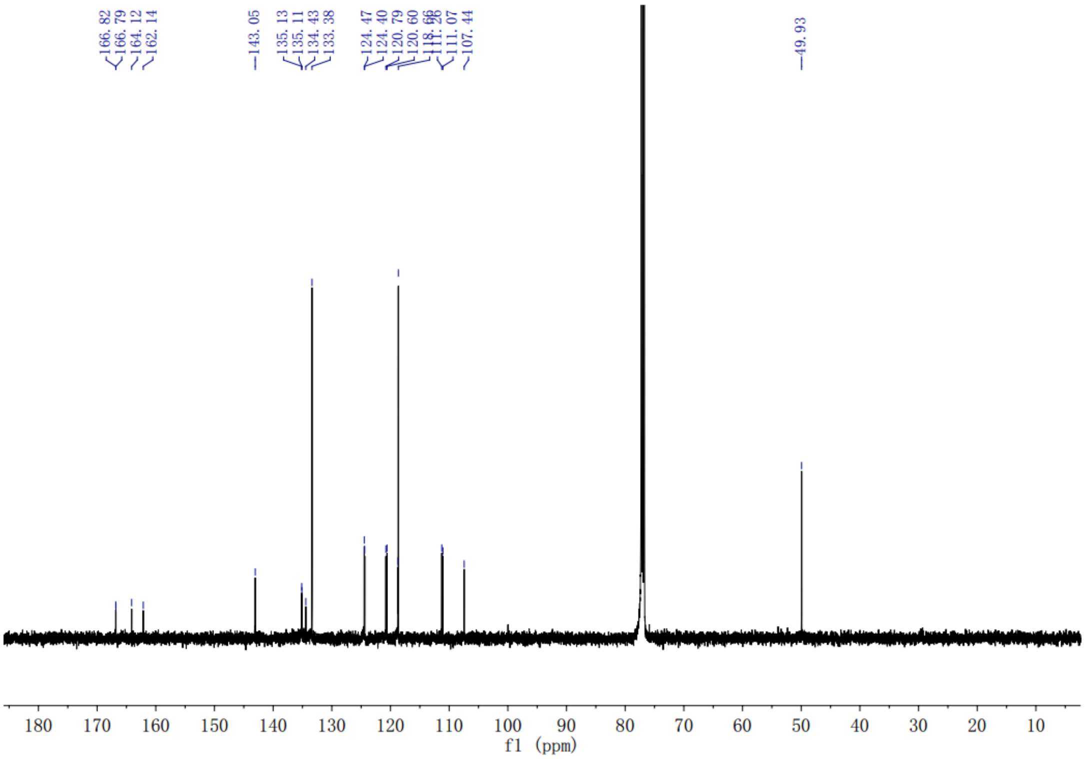

Supplementary Fig. 25. ^{13}C NMR spectrum of compound 1j.

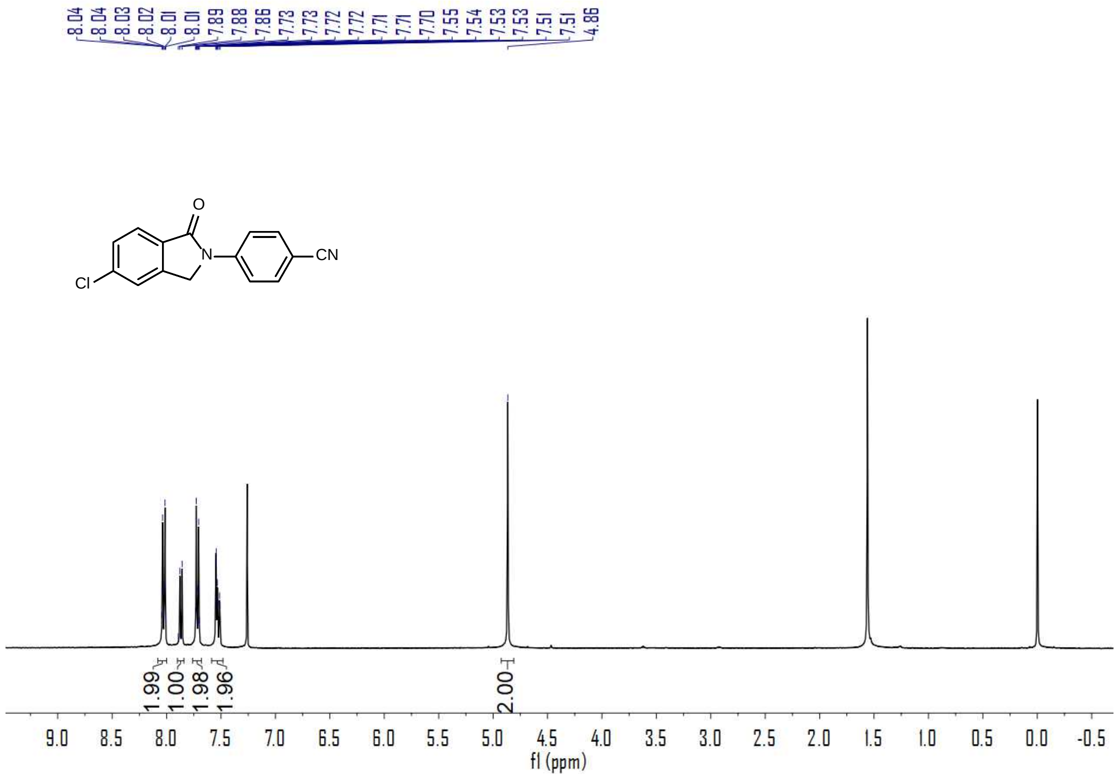

Supplementary Fig. 26. ^{1}H NMR spectrum of compound 1k.

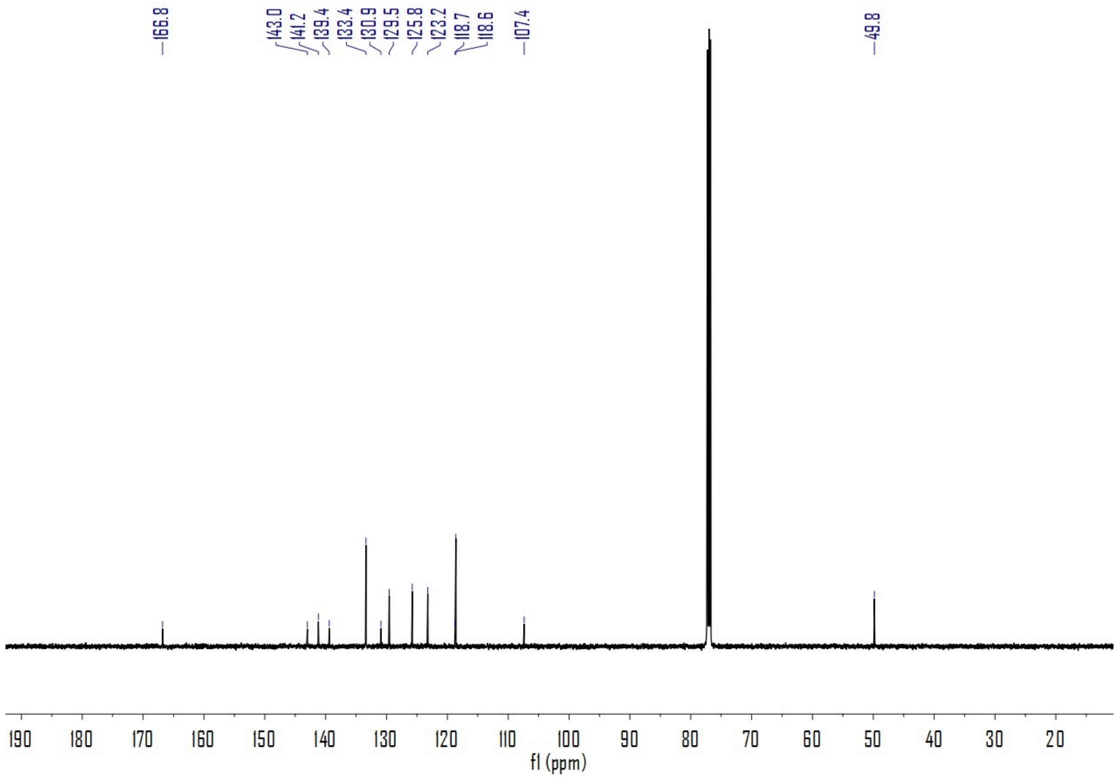

Supplementary Fig. 27. ^{13}C NMR spectrum of compound 1k.

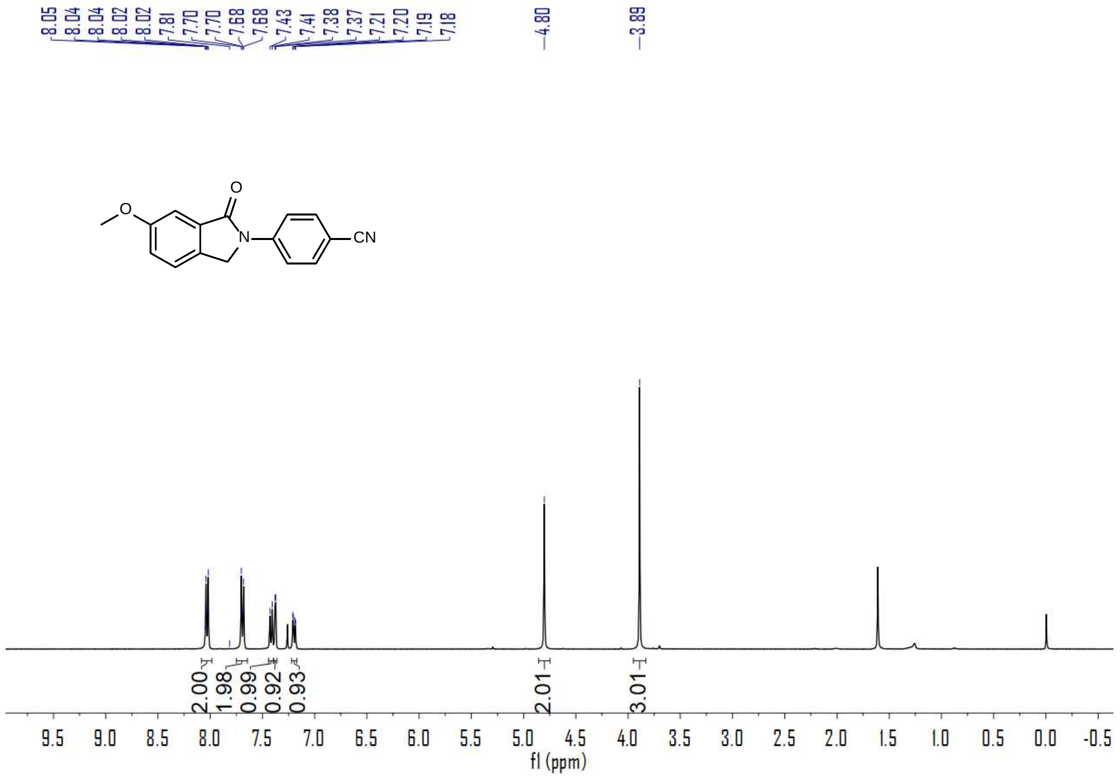

Supplementary Fig. 28. ^{1}**H NMR spectrum of compound 1l.**

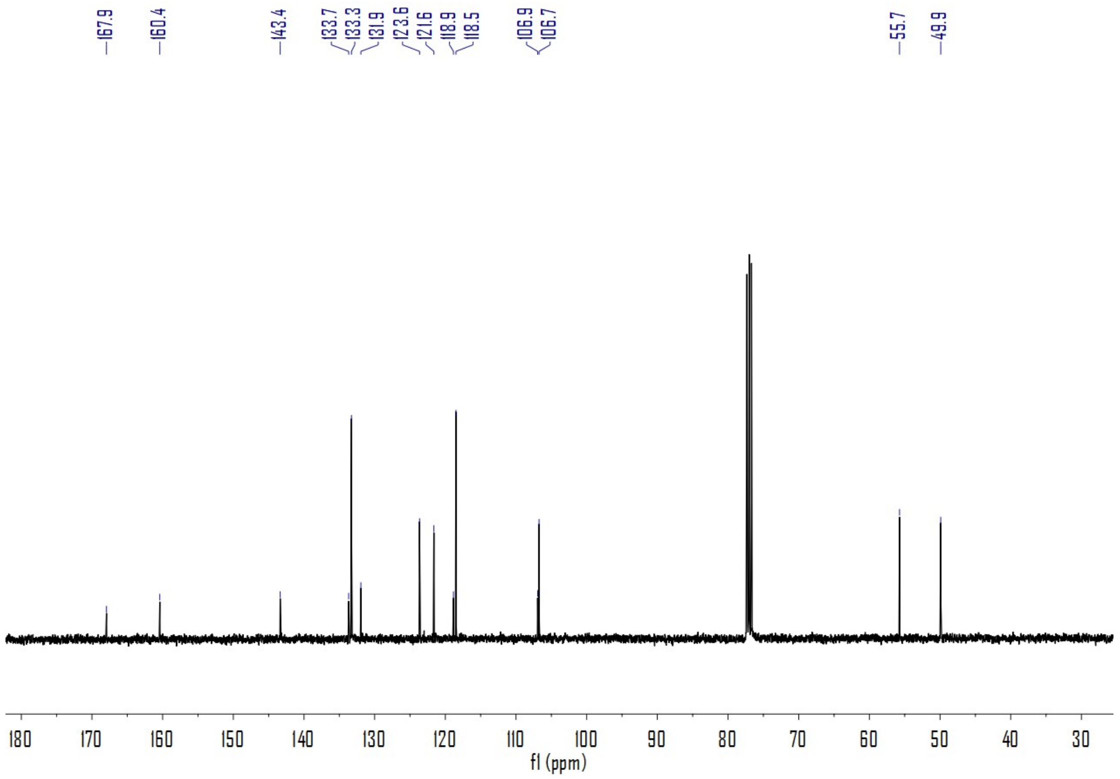

Supplementary Fig. 29. ^{13}**C NMR spectrum of compound 1l.**

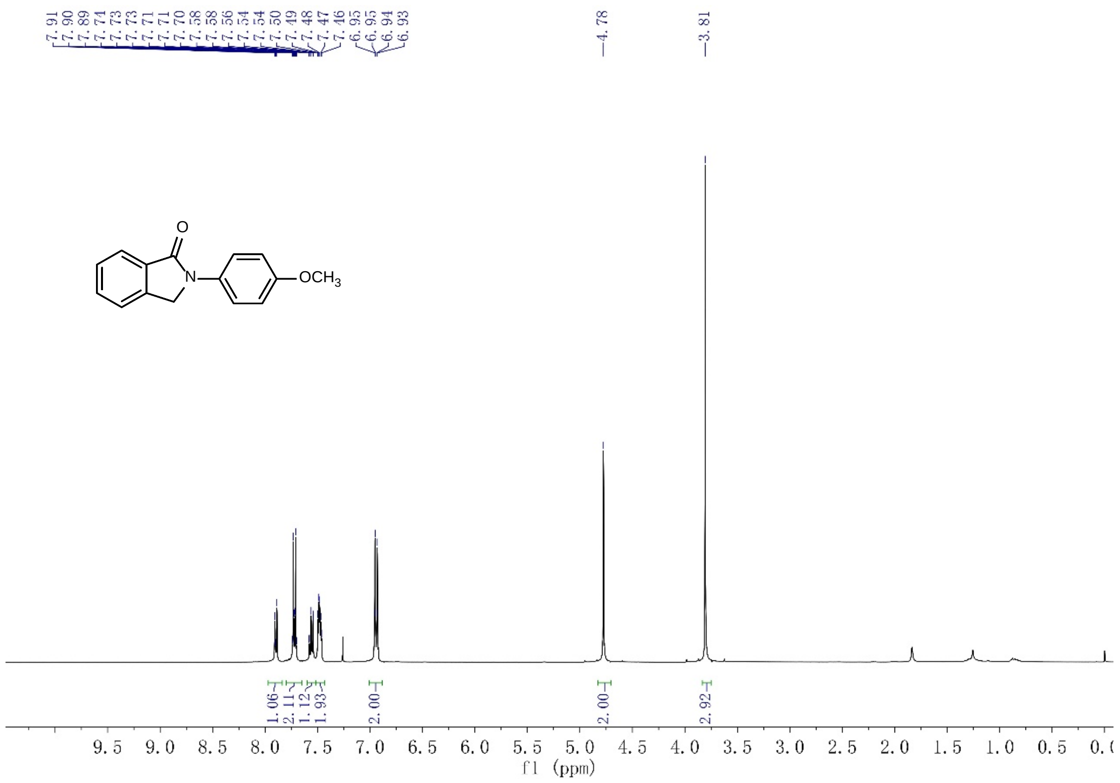

Supplementary Fig. 30. ^{1}H NMR spectrum of compound 1m.

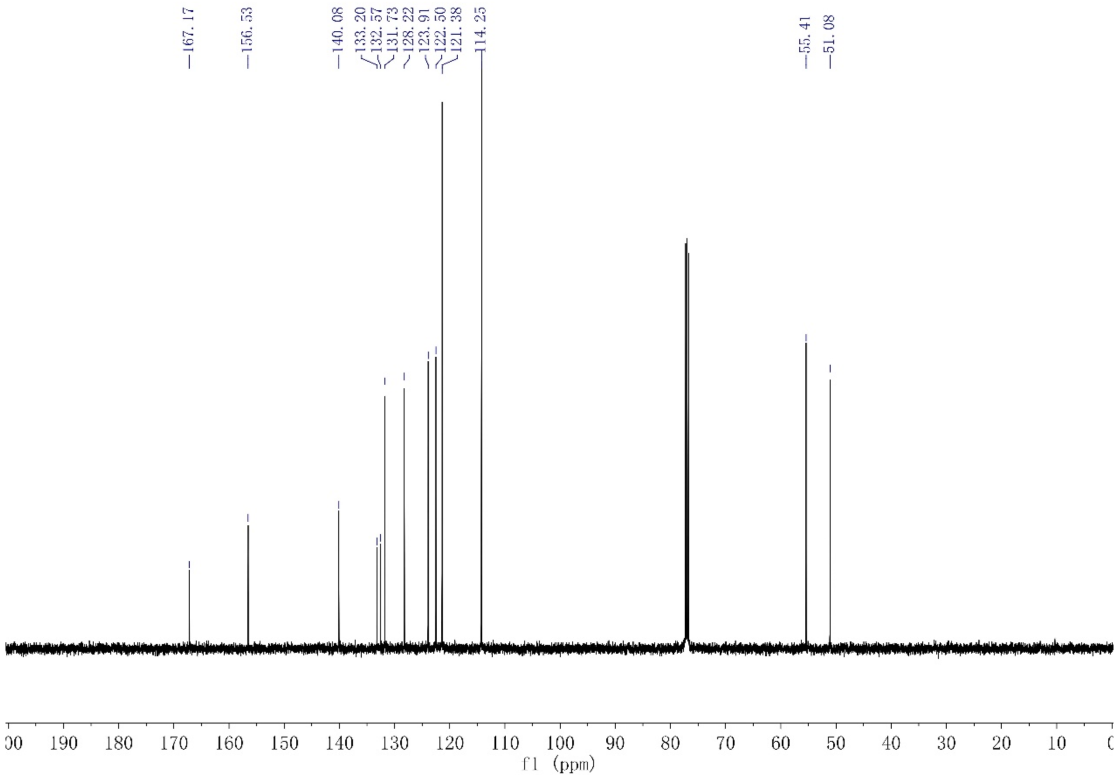

Supplementary Fig. 31. ^{13}C NMR spectrum of compound 1m.

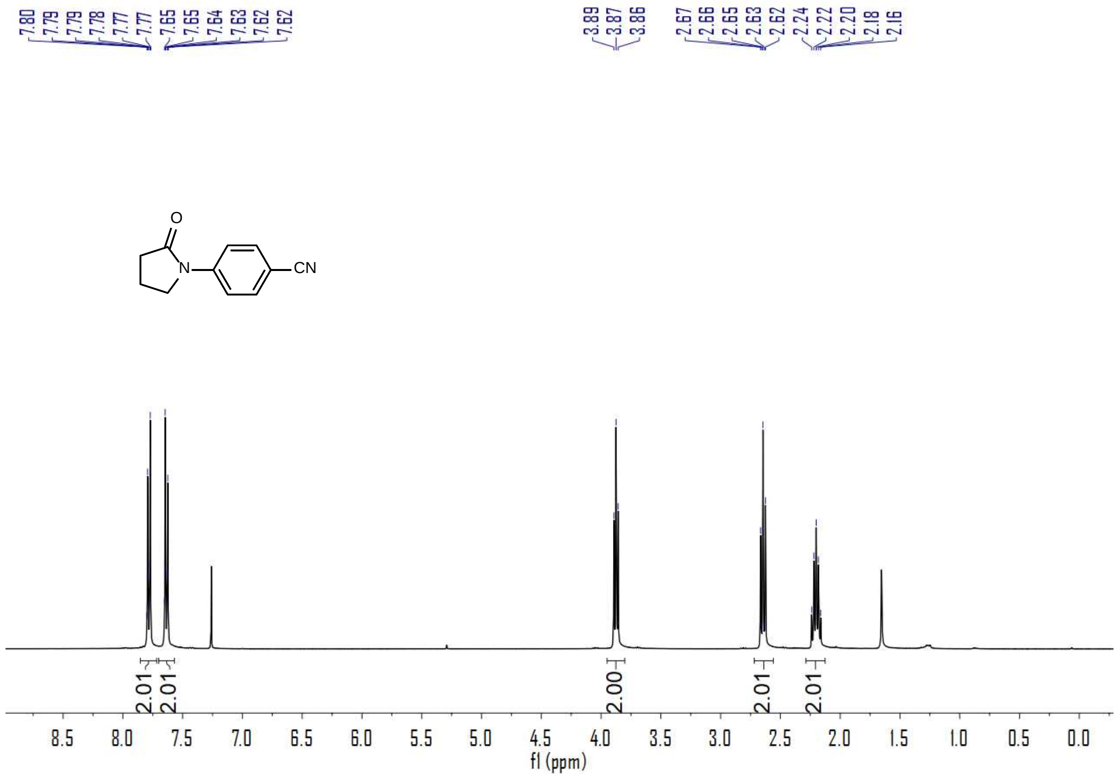

Supplementary Fig. 32. ^{1}H NMR spectrum of compound 1n.

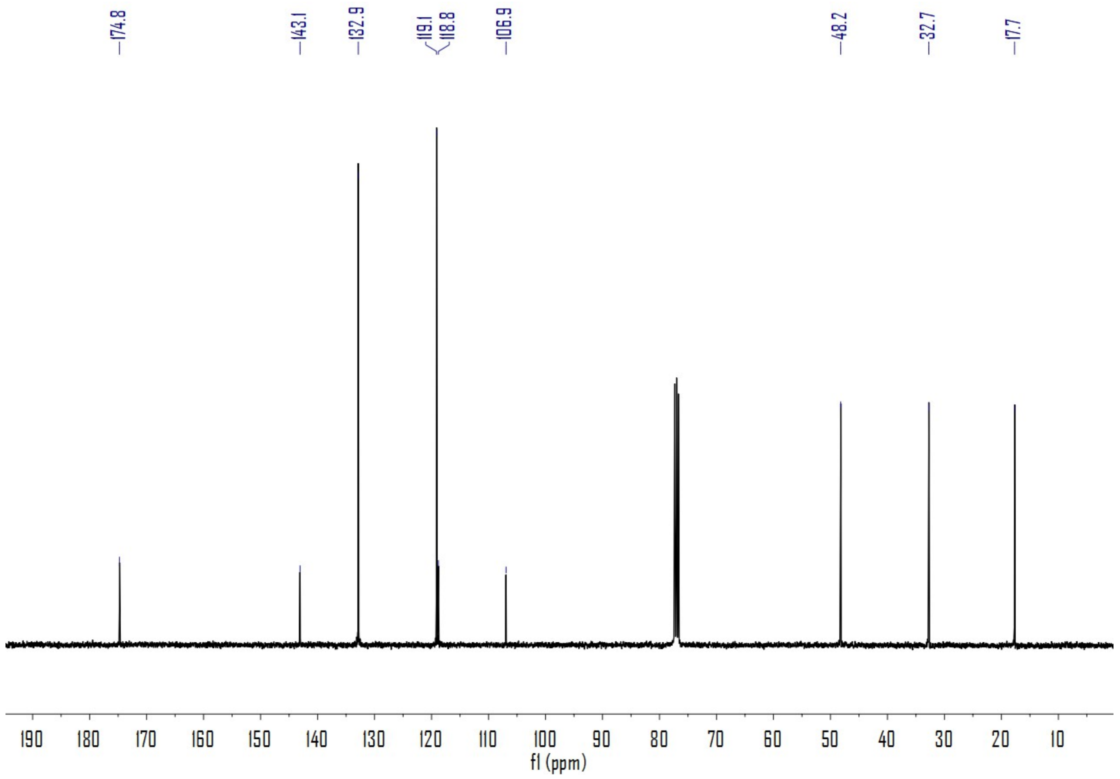

Supplementary Fig. 33. ^{13}C NMR spectrum of compound 1n.

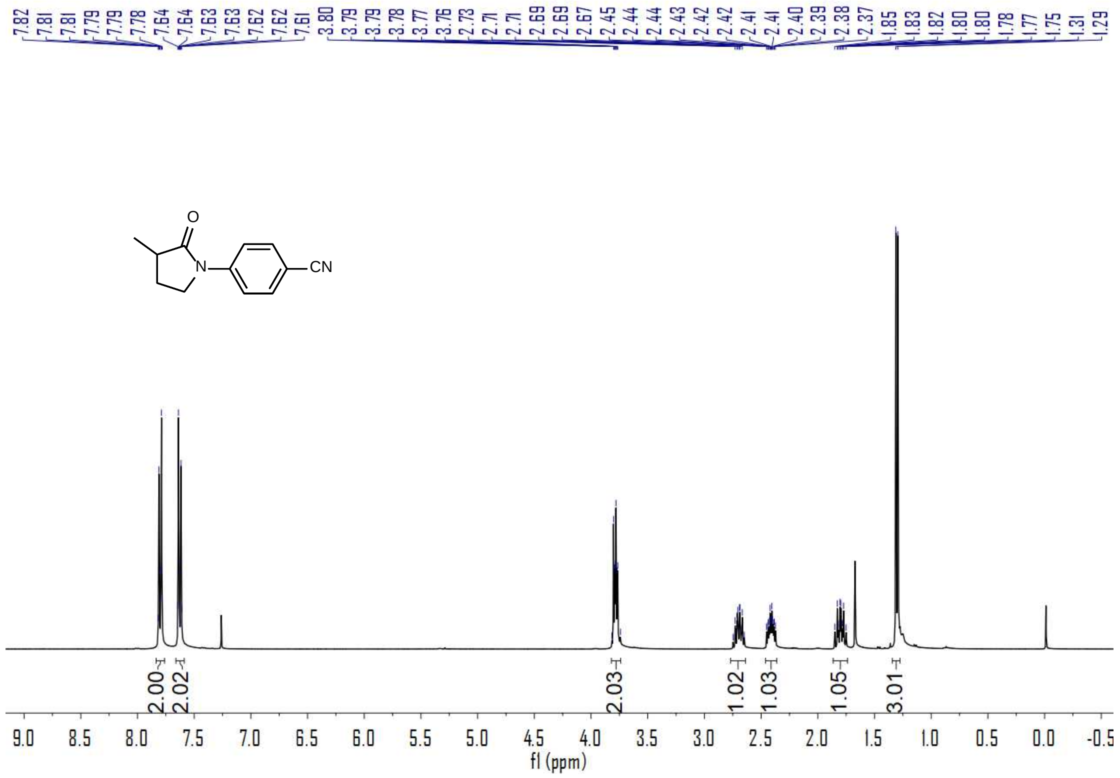

Supplementary Fig. 34. ^{1}H NMR spectrum of compound 1o.

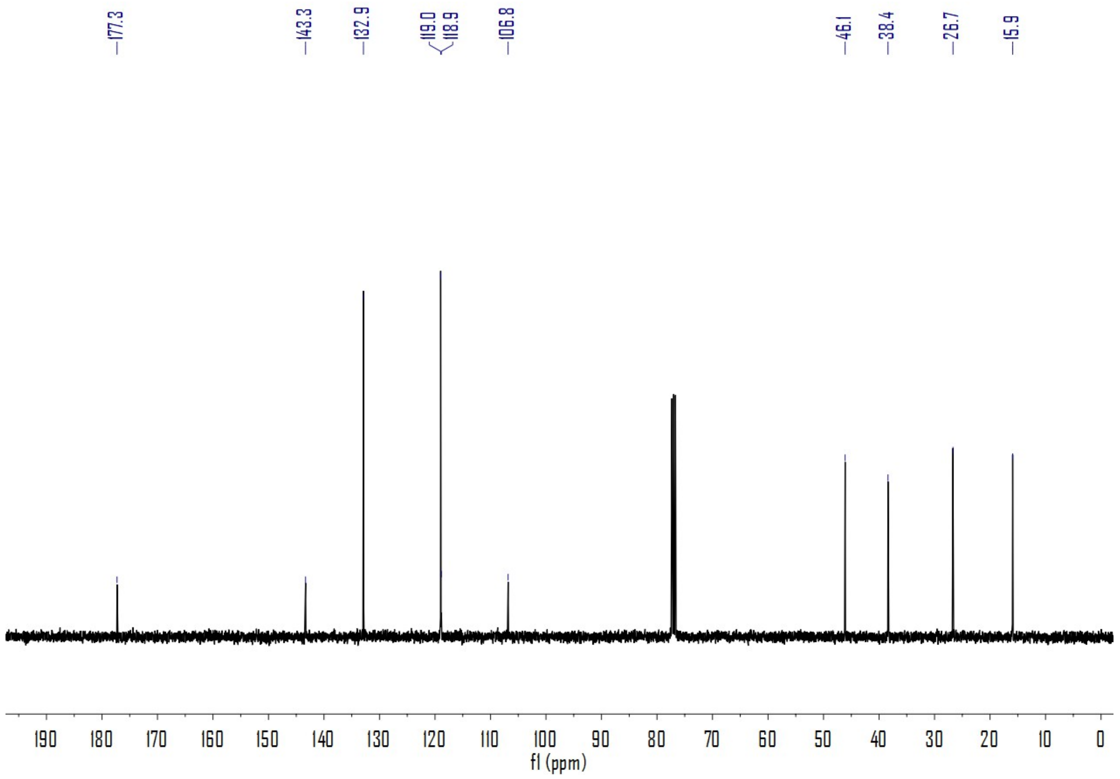

Supplementary Fig. 35. ^{13}C NMR spectrum of compound 1o.

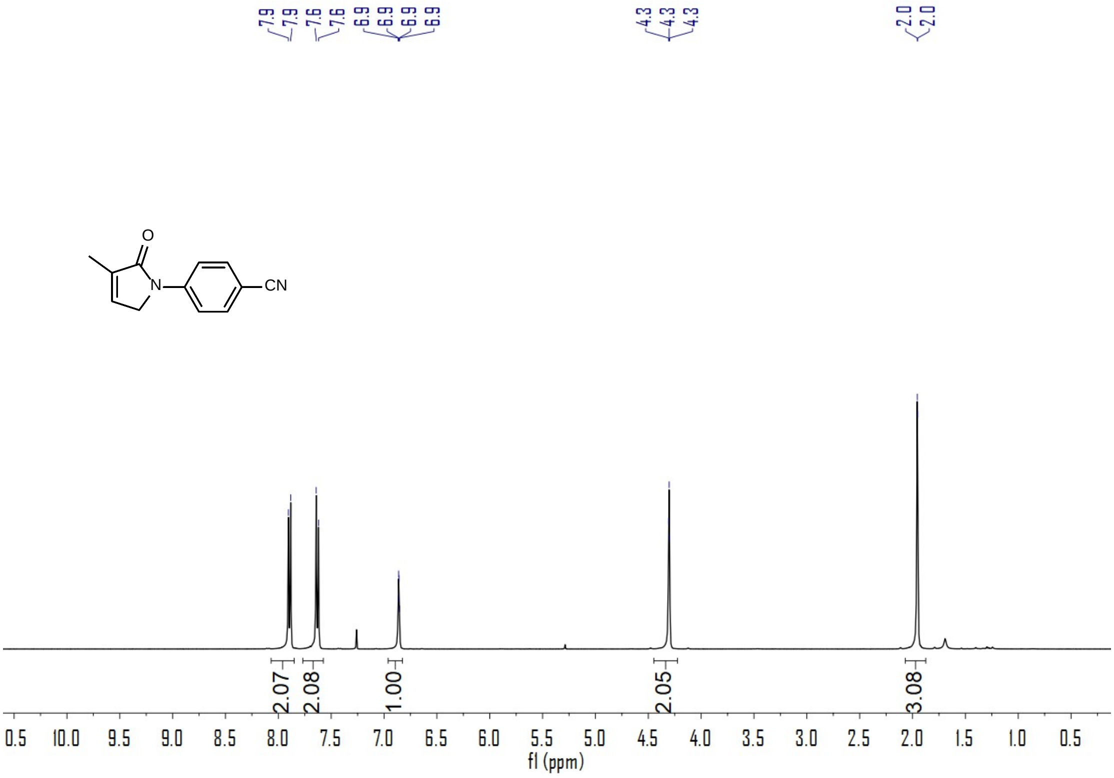

Supplementary Fig. 36. ^{1}H NMR spectrum of compound 1p.

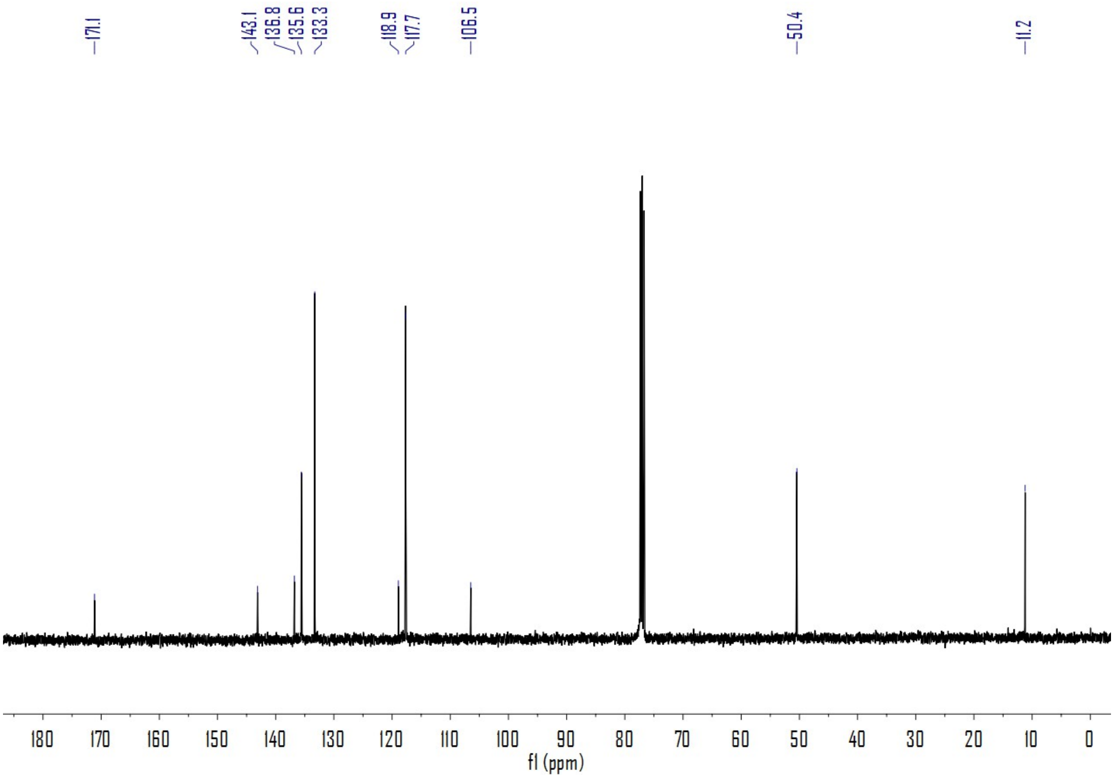

Supplementary Fig. 37. ^{13}C NMR spectrum of compound 1p.

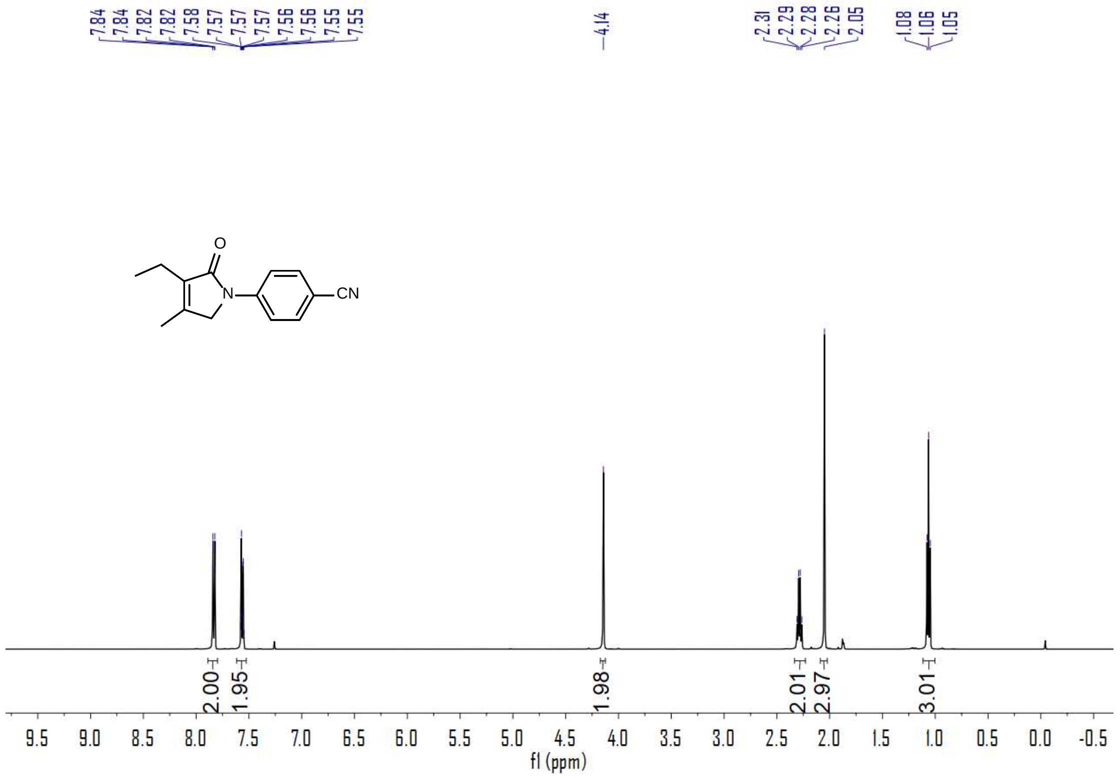

Supplementary Fig. 38. ^{1}H NMR spectrum of compound 1q.

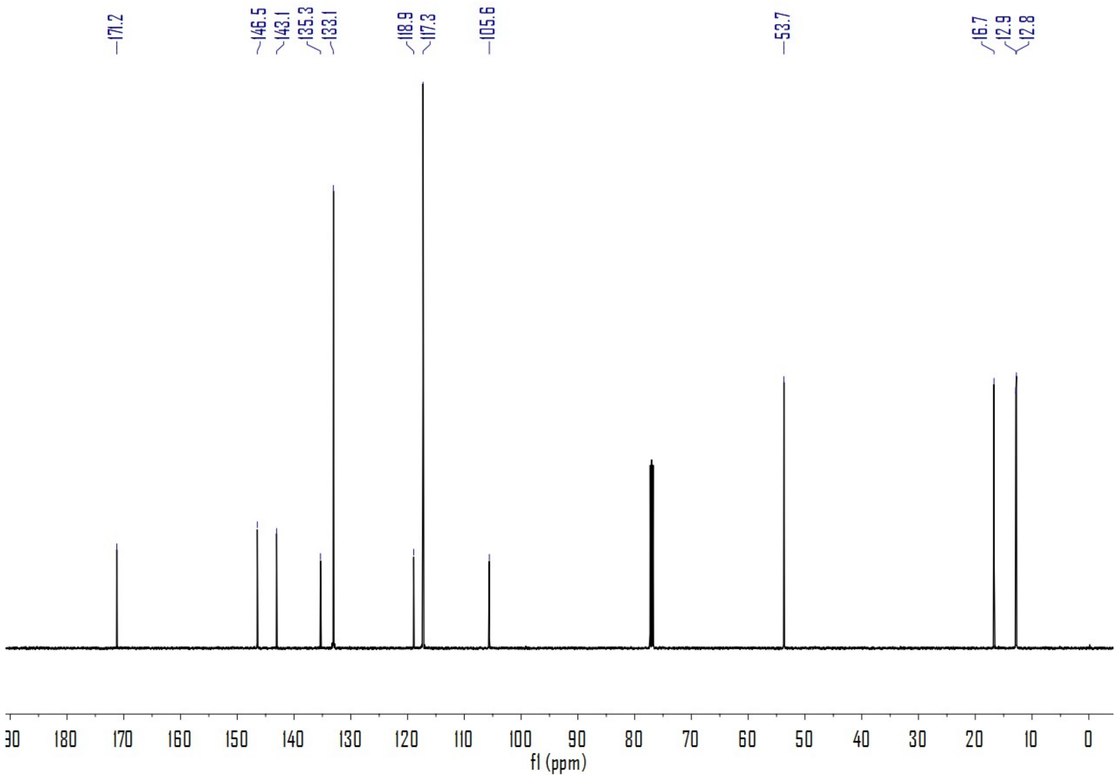

Supplementary Fig. 39. ^{13}C NMR spectrum of compound 1q.

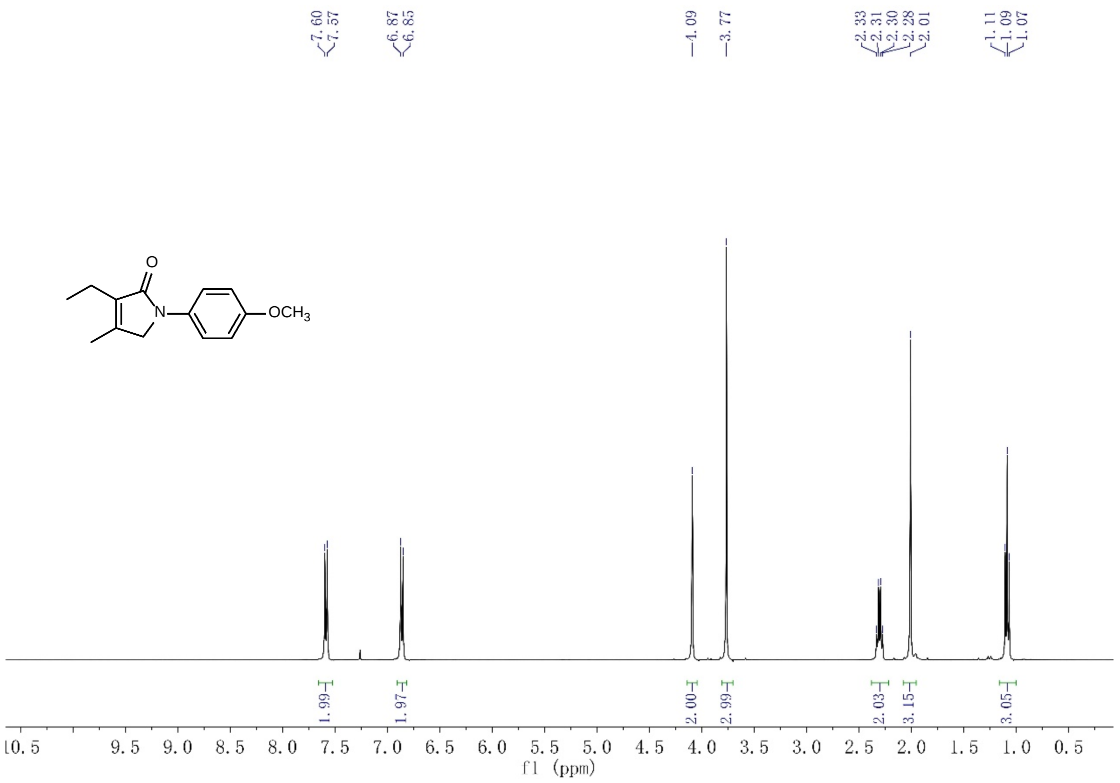

Supplementary Fig. 40. ^{1}H NMR spectrum of compound 1r.

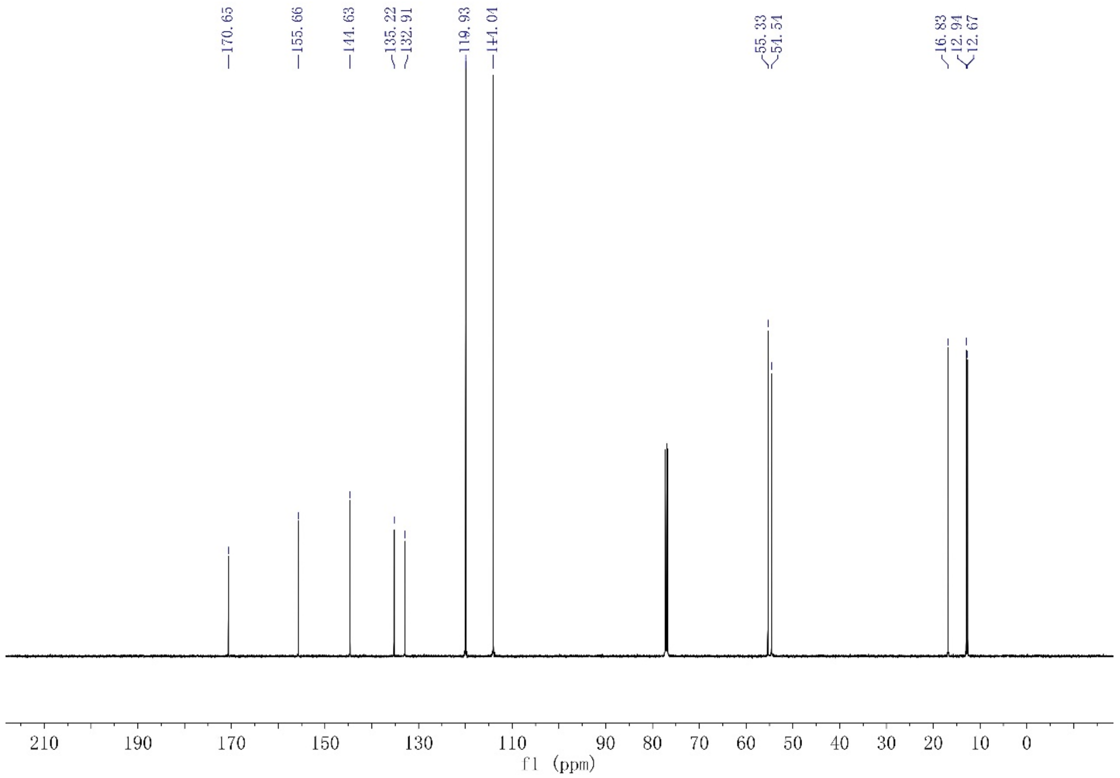

Supplementary Fig. 41. ^{13}C NMR spectrum of compound 1r.

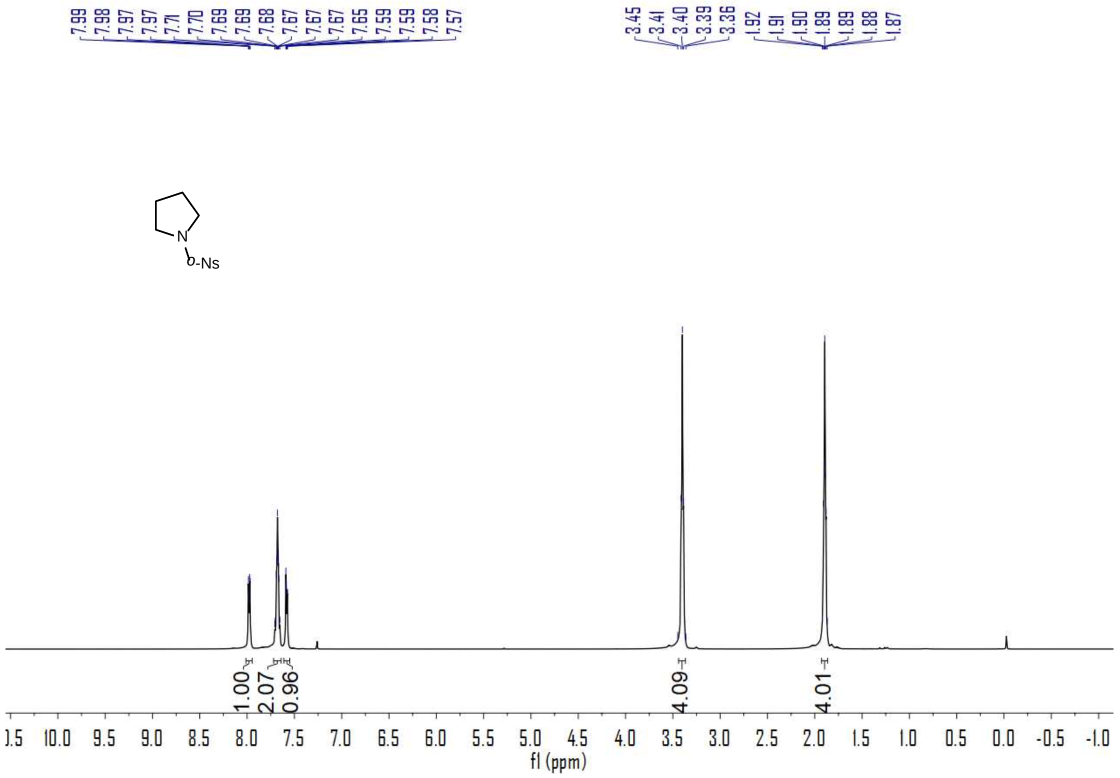

Supplementary Fig. 42. ^{1}H NMR spectrum of compound 1s.

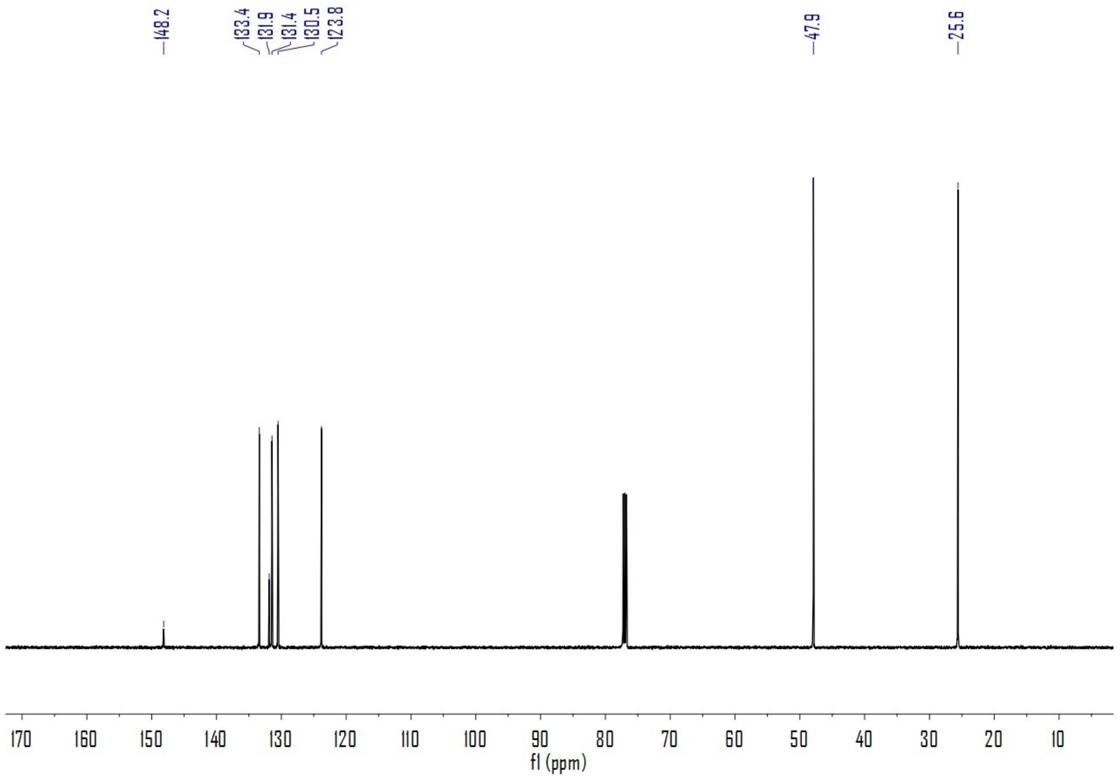

Supplementary Fig. 43. ^{13}C NMR spectrum of compound 1s.

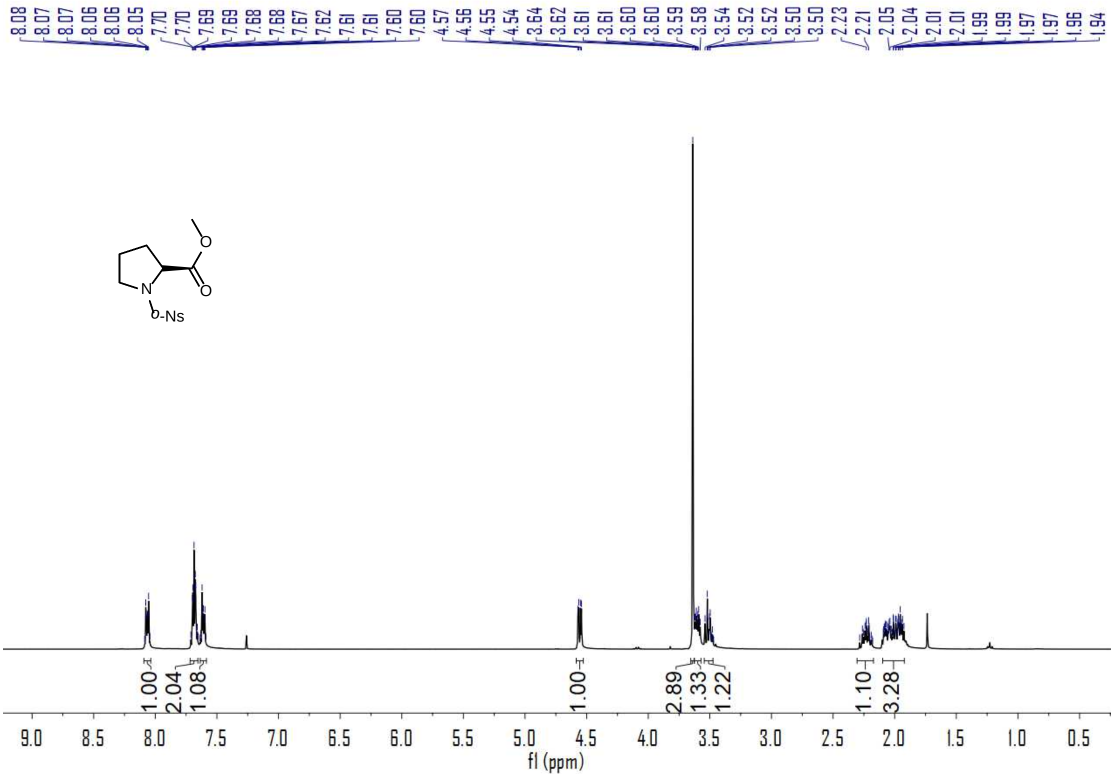

Supplementary Fig. 44. ^{1}H NMR spectrum of compound 1t.

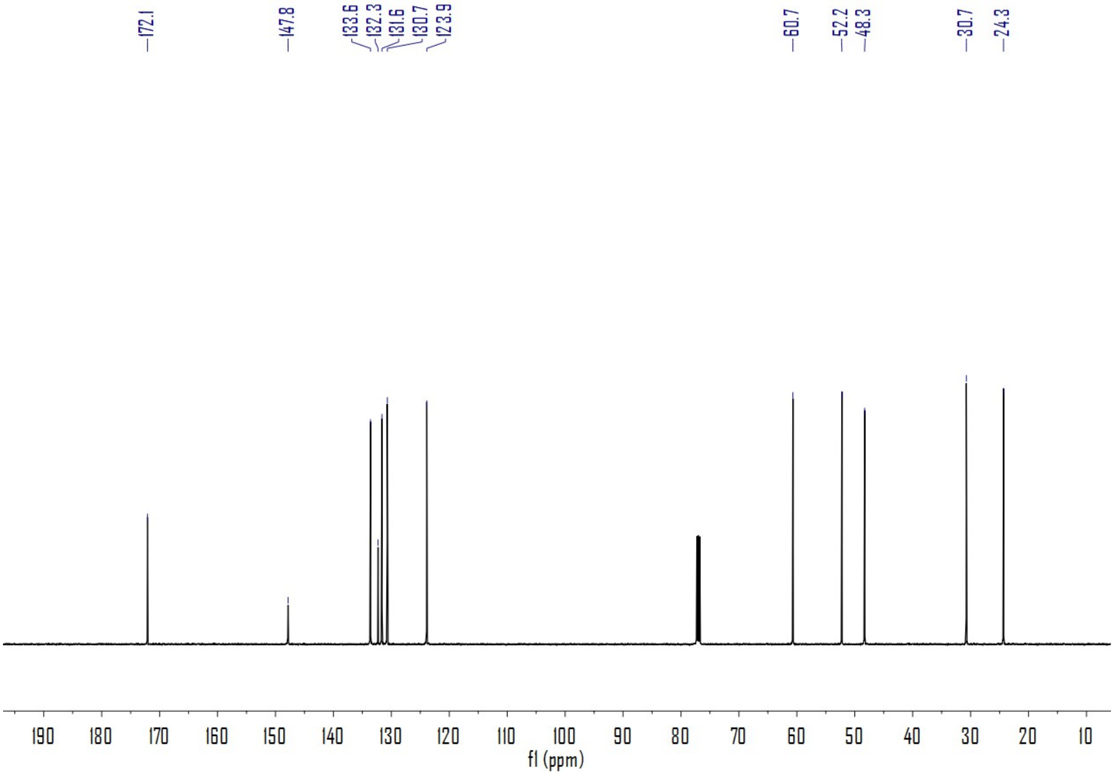

Supplementary Fig. 45. ^{13}C NMR spectrum of compound 1t.

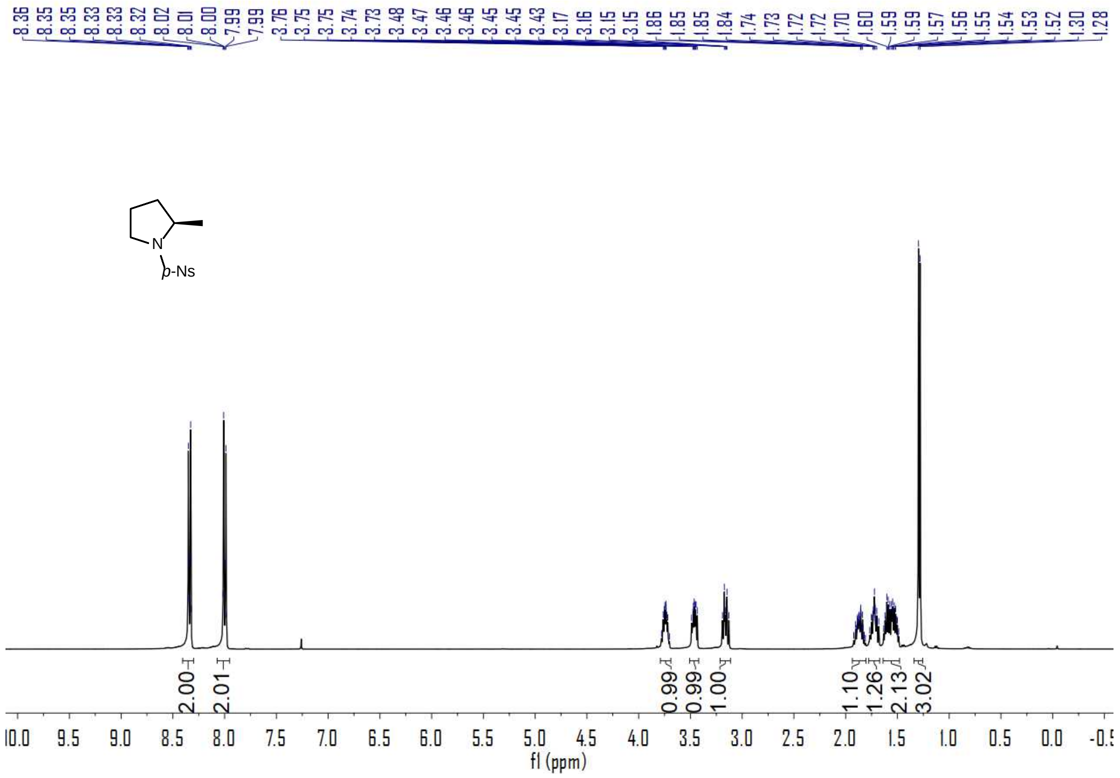

Supplementary Fig. 46. ^{1}H NMR spectrum of compound 1u.

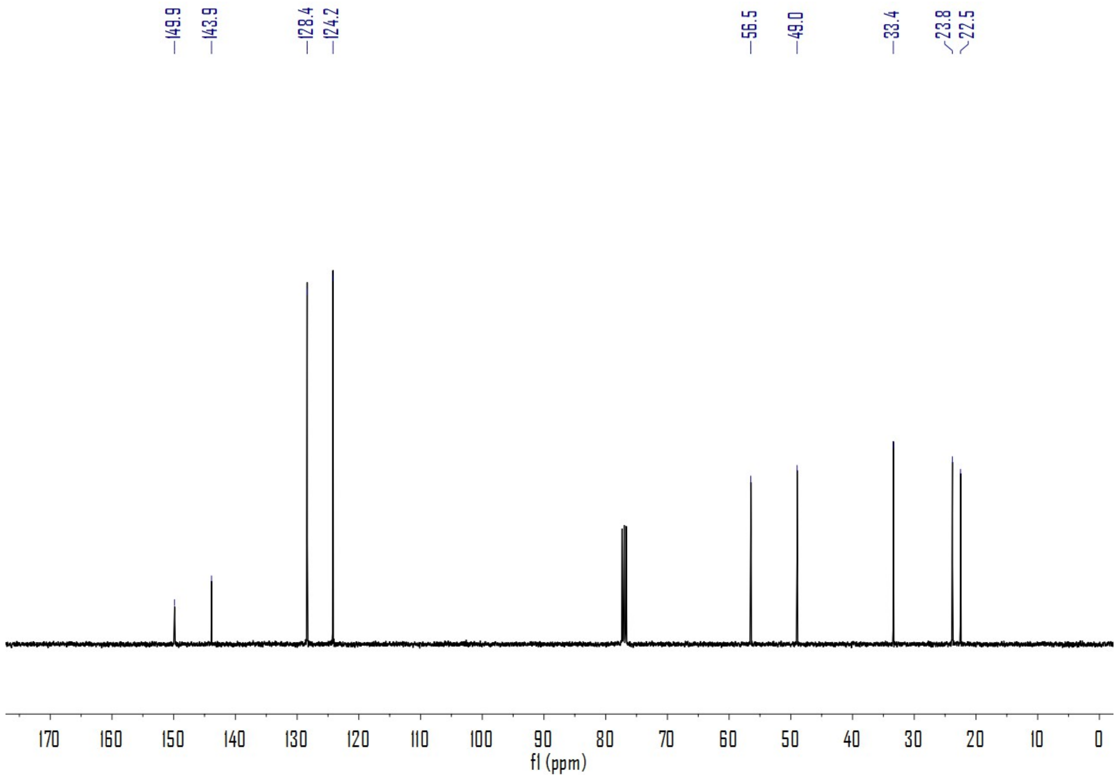

Supplementary Fig. 47. ^{13}C NMR spectrum of compound 1u.

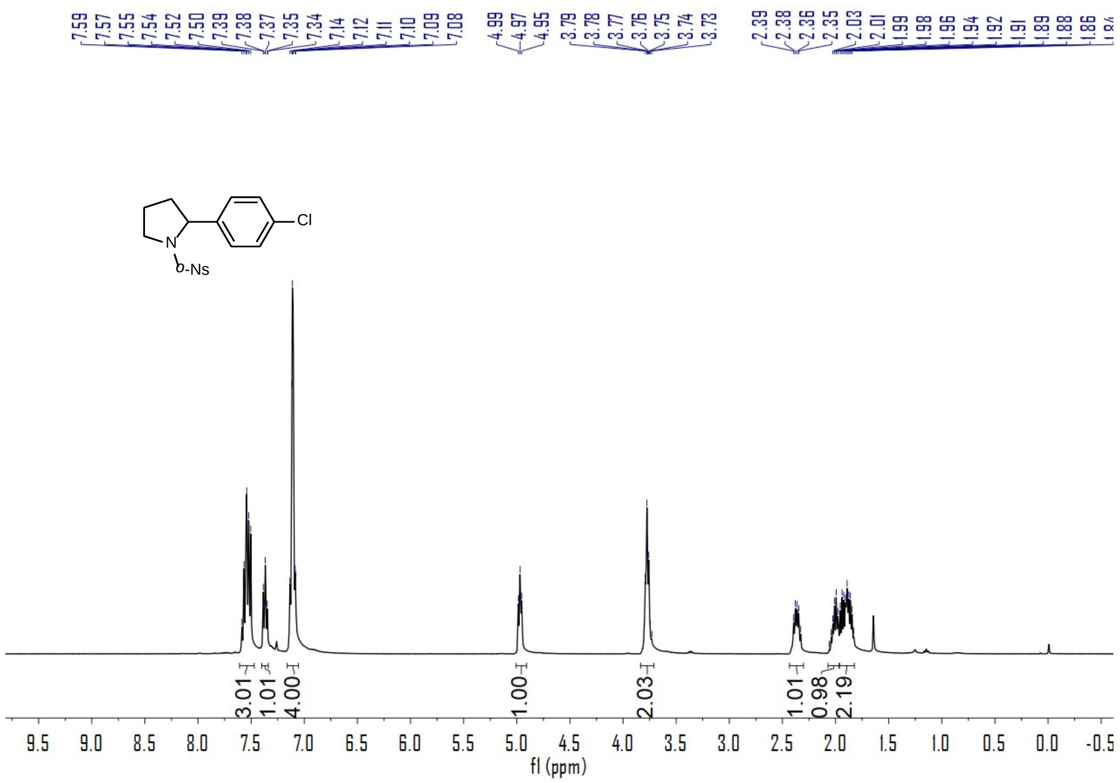

Supplementary Fig. 48. ^{1}H NMR spectrum of compound 1v.

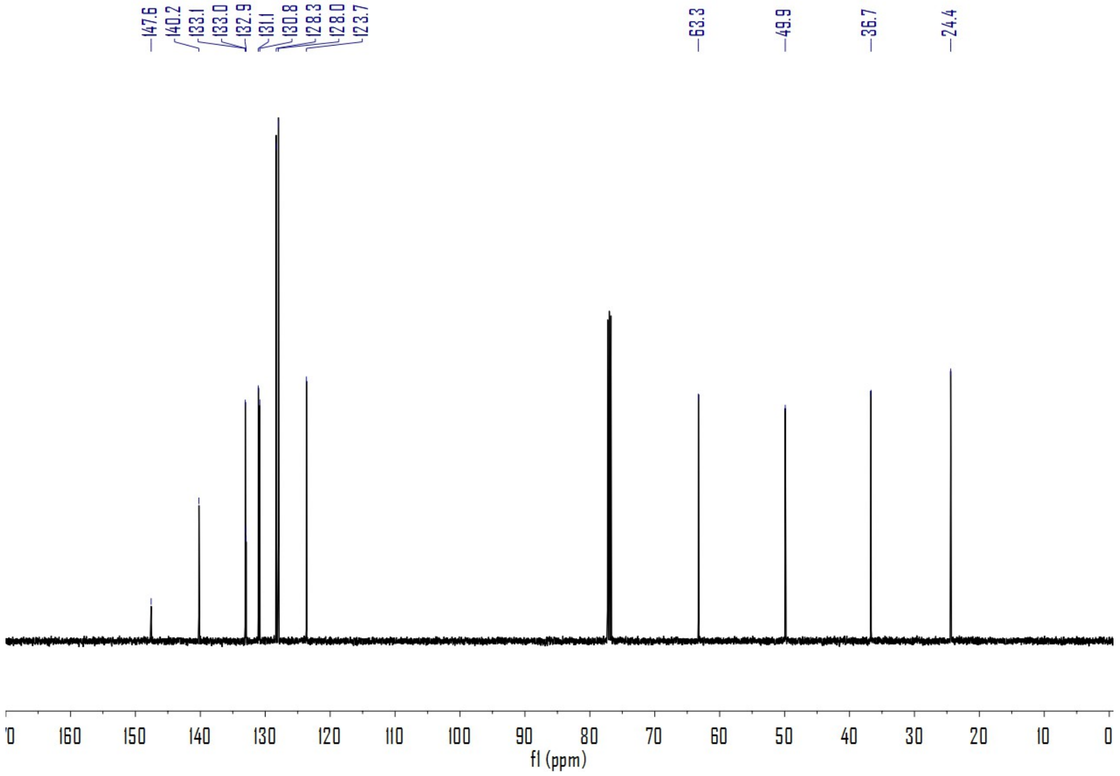

Supplementary Fig. 49. ^{13}C NMR spectrum of compound 1v.

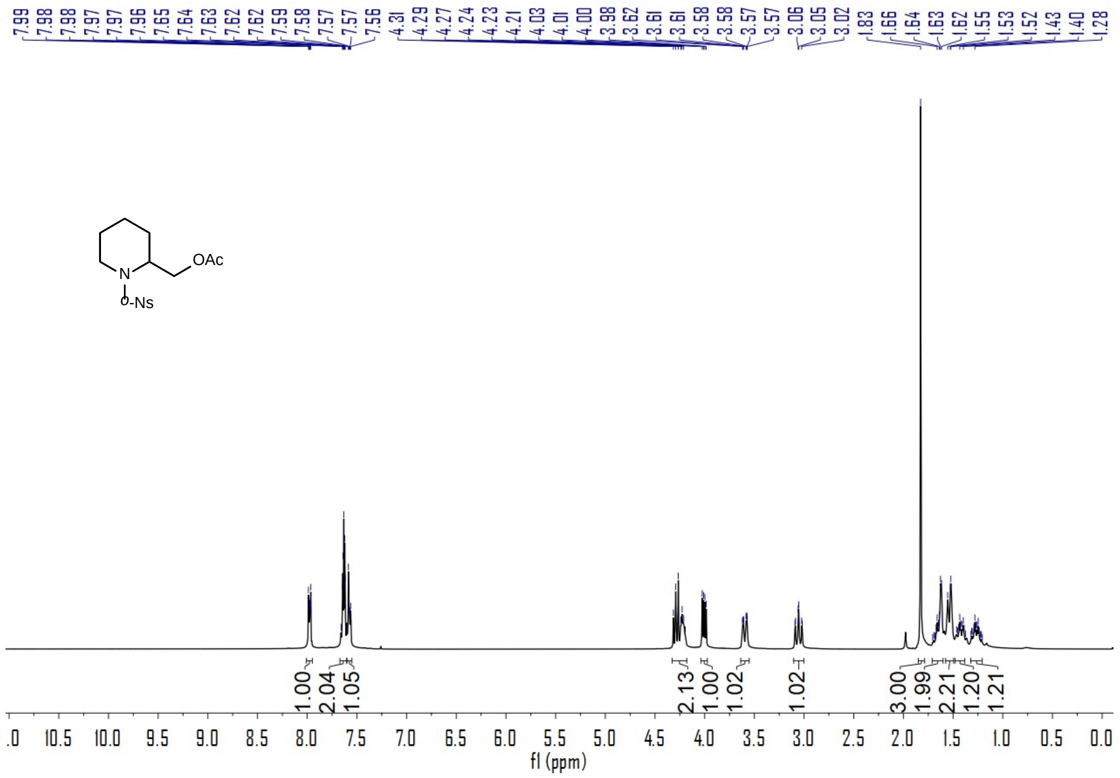

Supplementary Fig. 50. ^{1}**H NMR spectrum of compound 1w.**

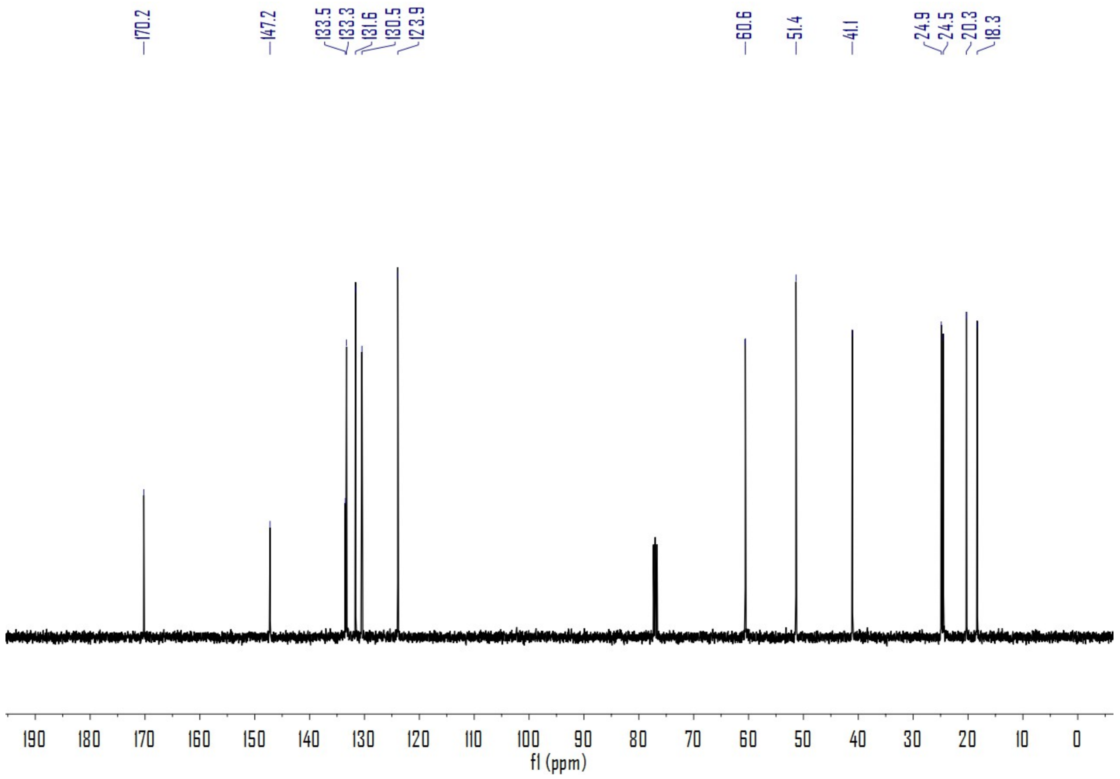

Supplementary Fig. 51. ^{13}**C NMR spectrum of compound 1w.**

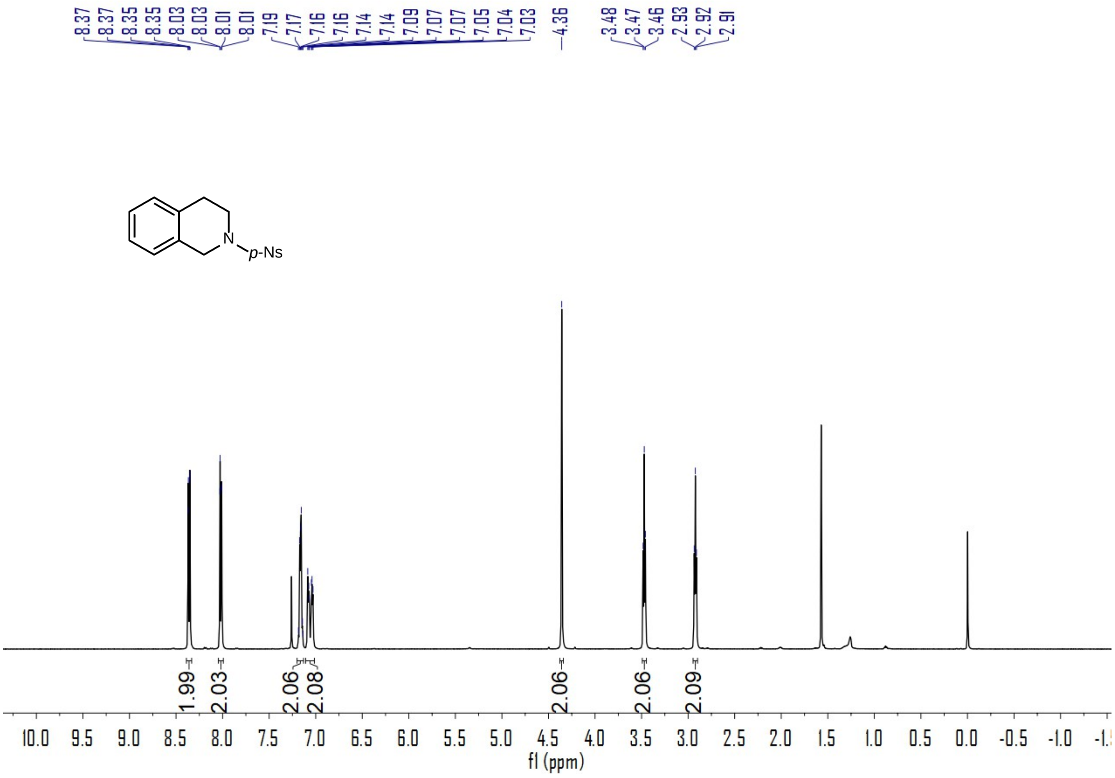

Supplementary Fig. 52. ^{1}H NMR spectrum of compound 1x.

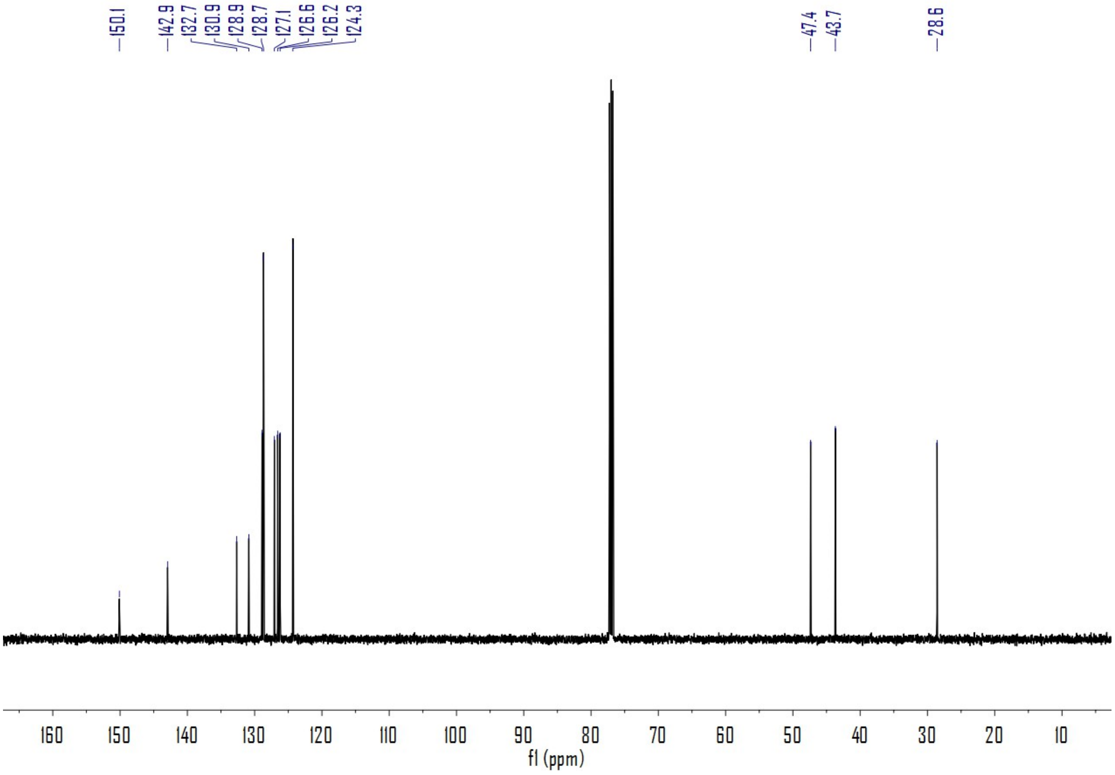

Supplementary Fig. 53. ^{13}C NMR spectrum of compound 1x.

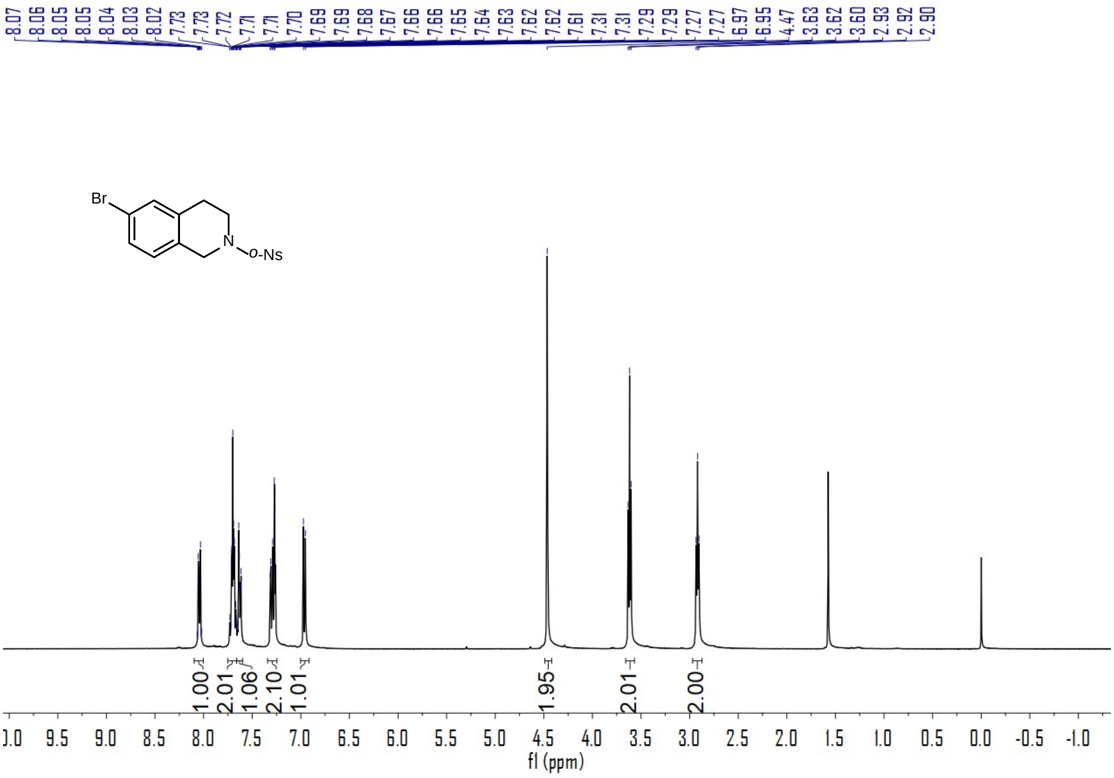

Supplementary Fig. 54. ^{1}H NMR spectrum of compound 1y.

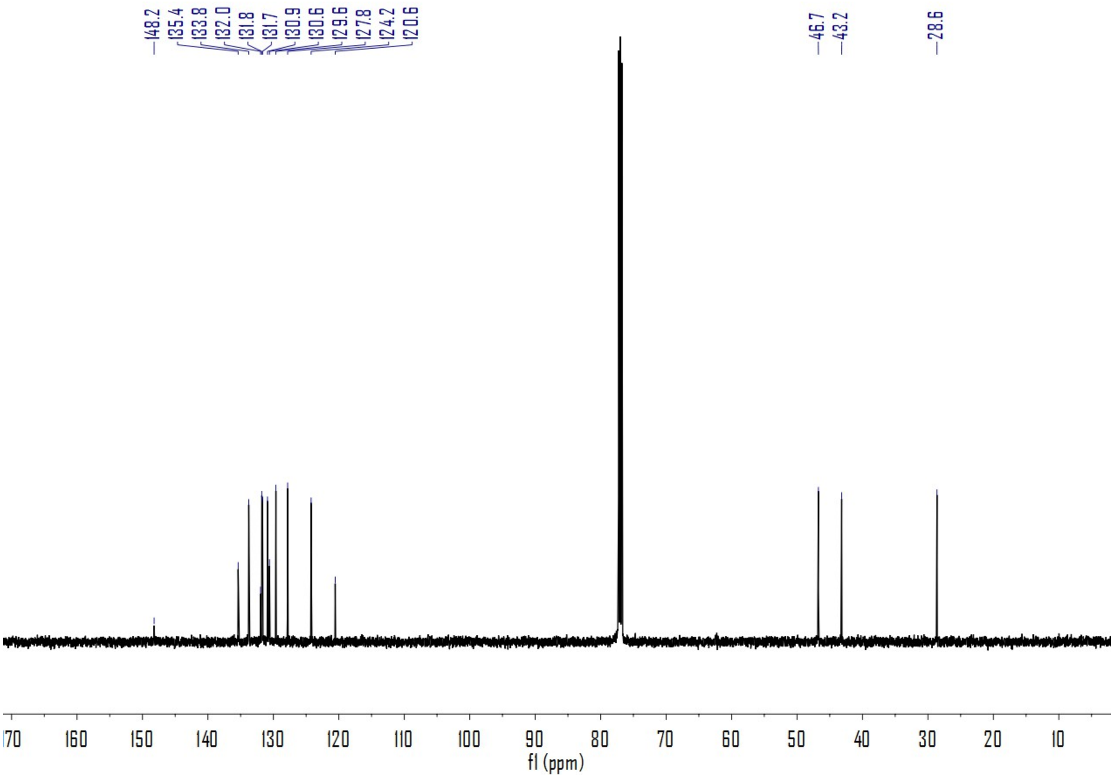

Supplementary Fig. 55. ^{13}C NMR spectrum of compound 1y.

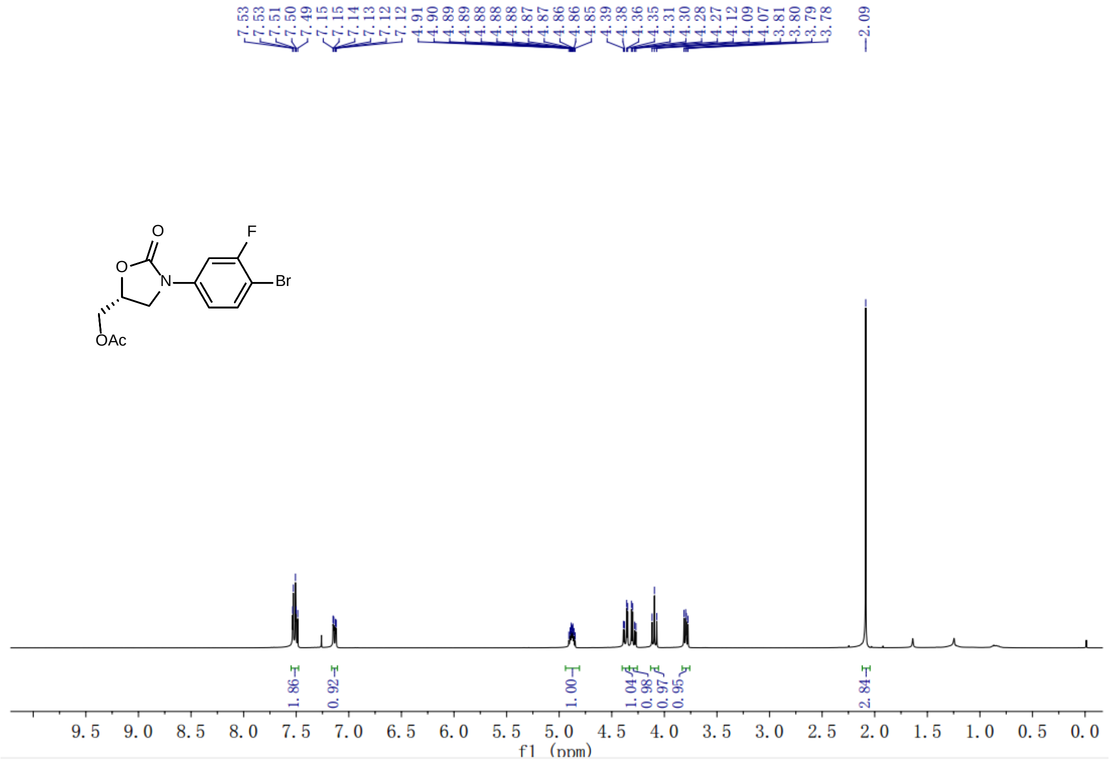

Supplementary Fig. 56. ^{1}H NMR spectrum of compound 1z2.

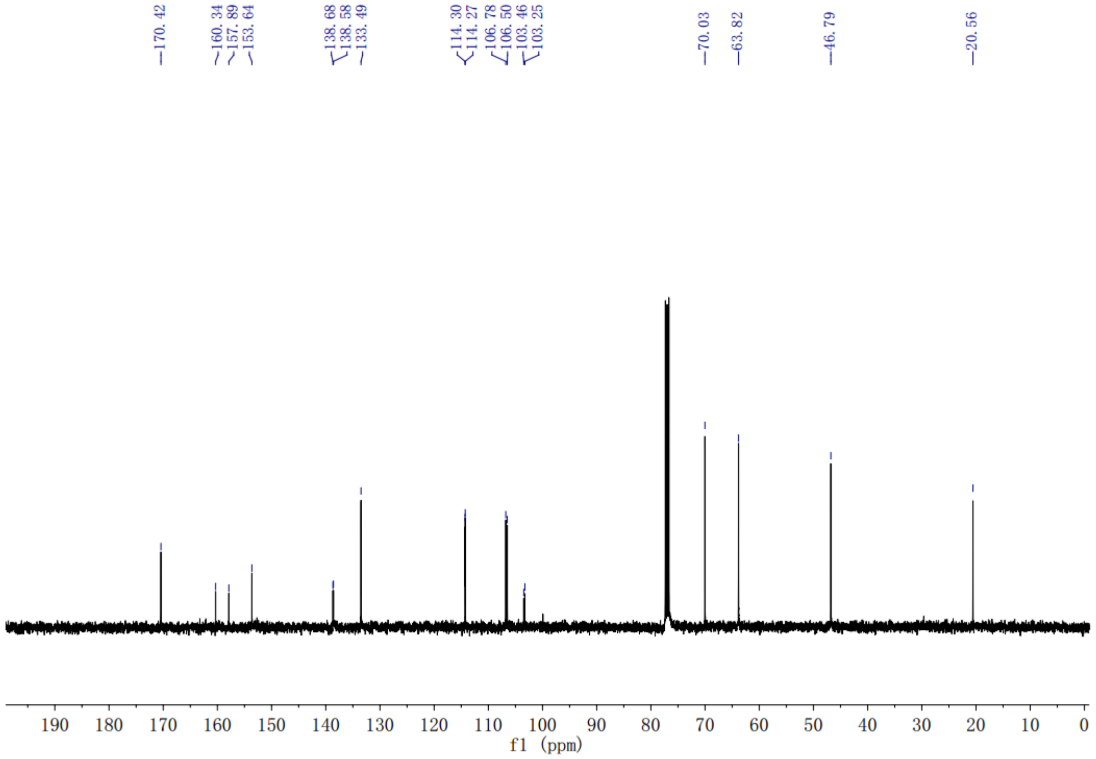

Supplementary Fig. 57. ^{13}C NMR spectrum of compound 1z2.

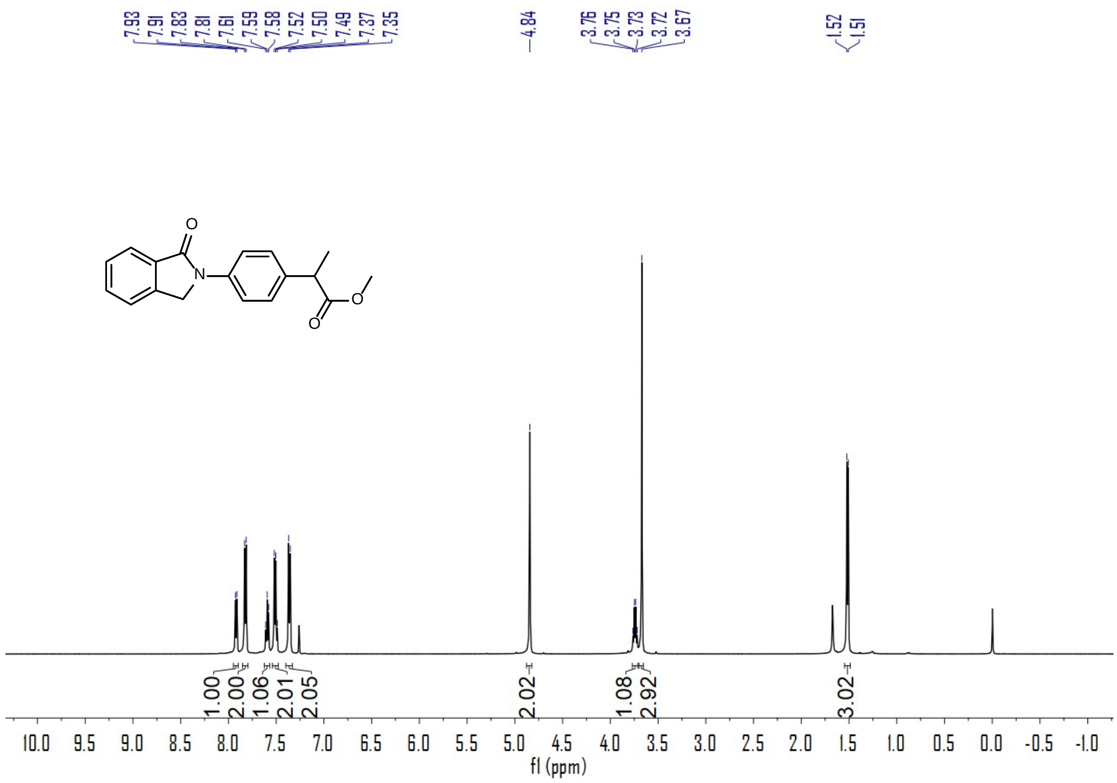

Supplementary Fig. 58. ^{1}H NMR spectrum of compound 1z3.

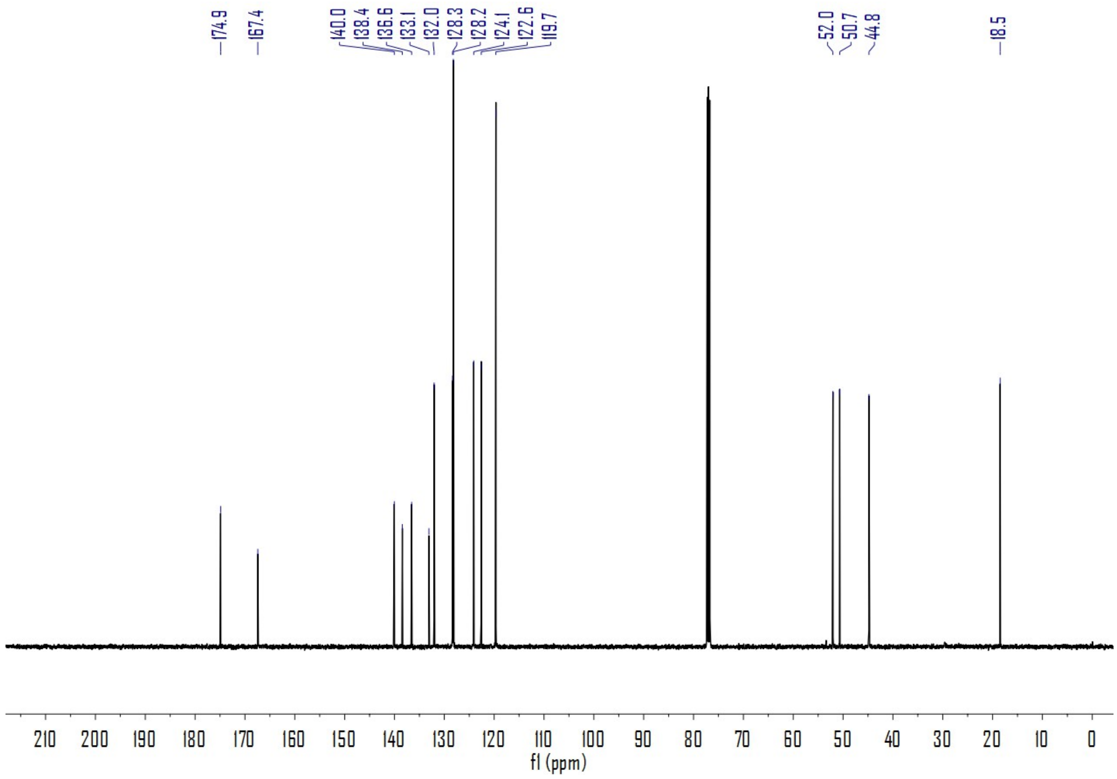

Supplementary Fig. 59. ^{13}C NMR spectrum of compound 1z3.

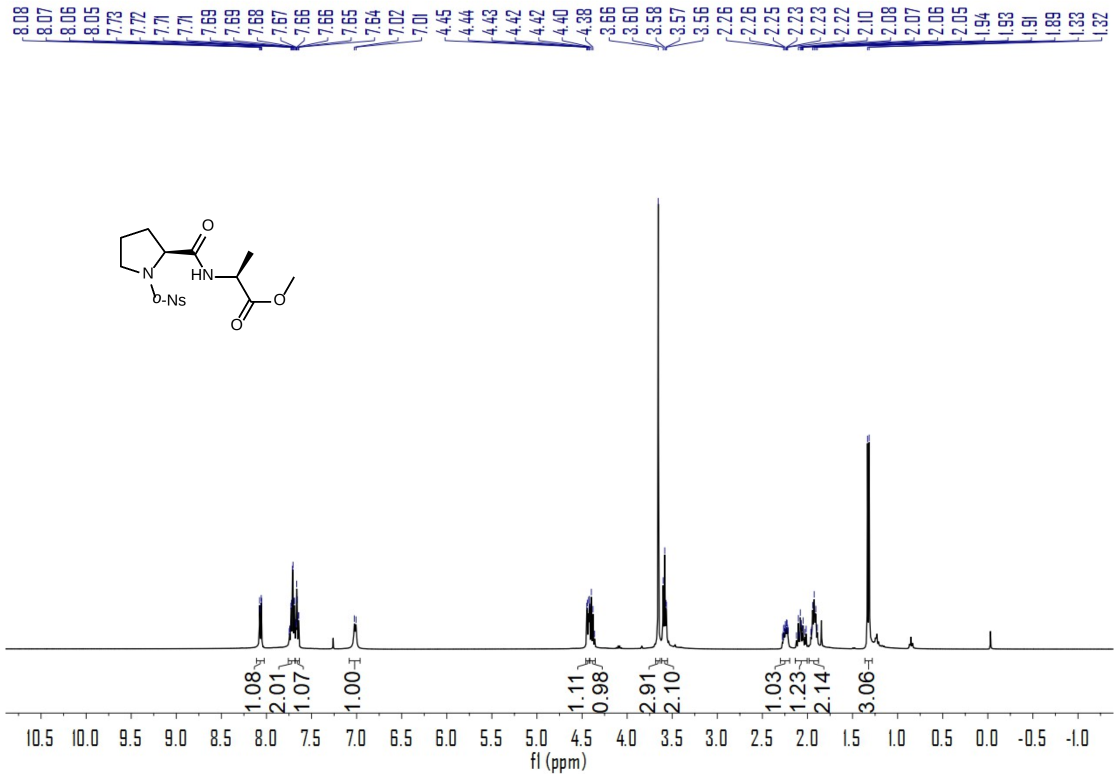

Supplementary Fig. 60. ^{1}H NMR spectrum of compound 1z4.

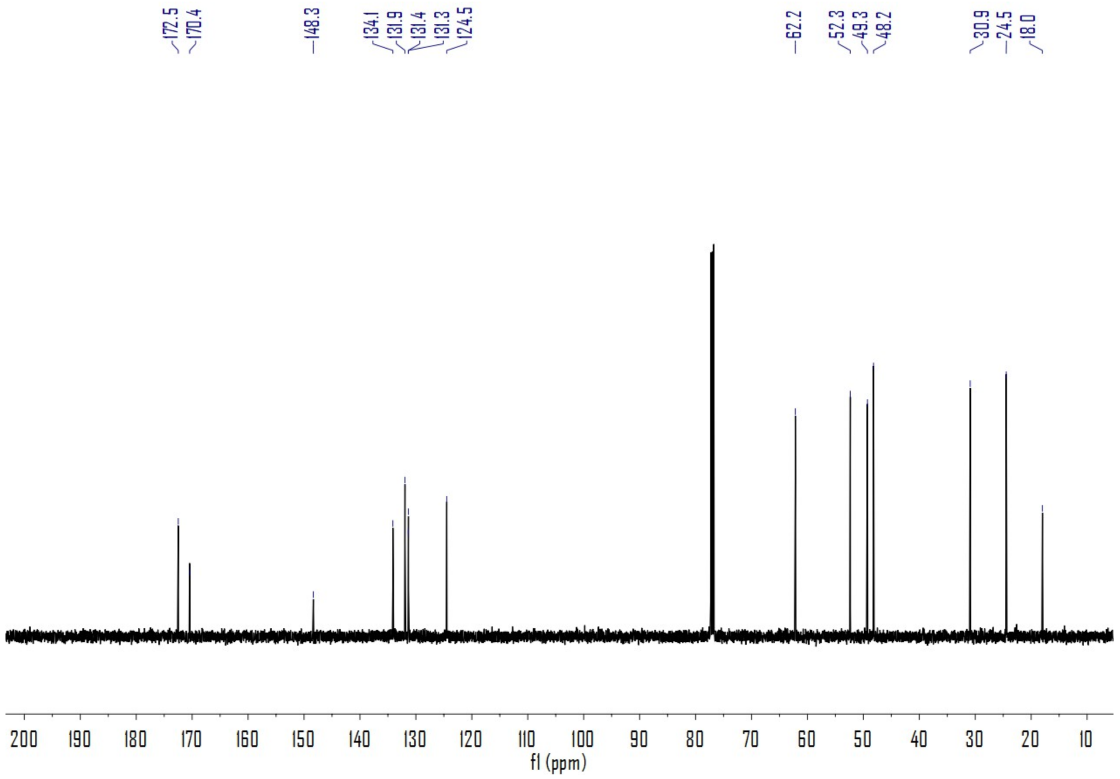

Supplementary Fig. 61. ^{13}C NMR spectrum of compound 1z4.

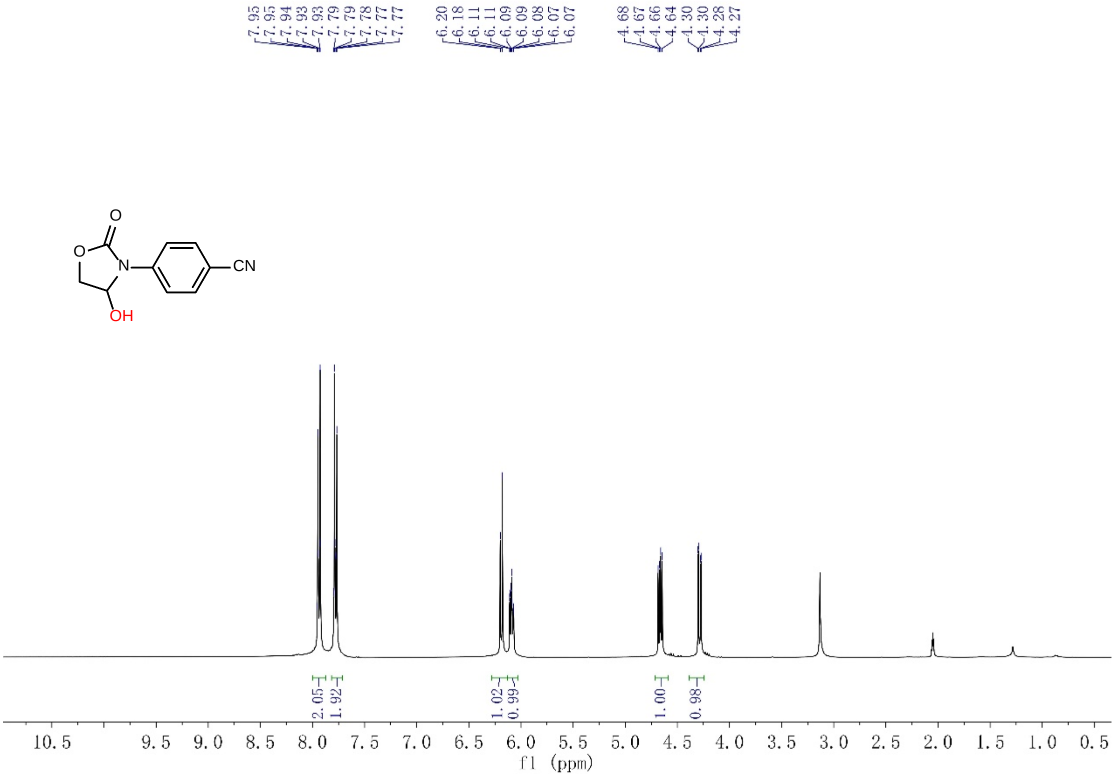

Supplementary Fig. 62. ^{1}H NMR spectrum of compound 2a.

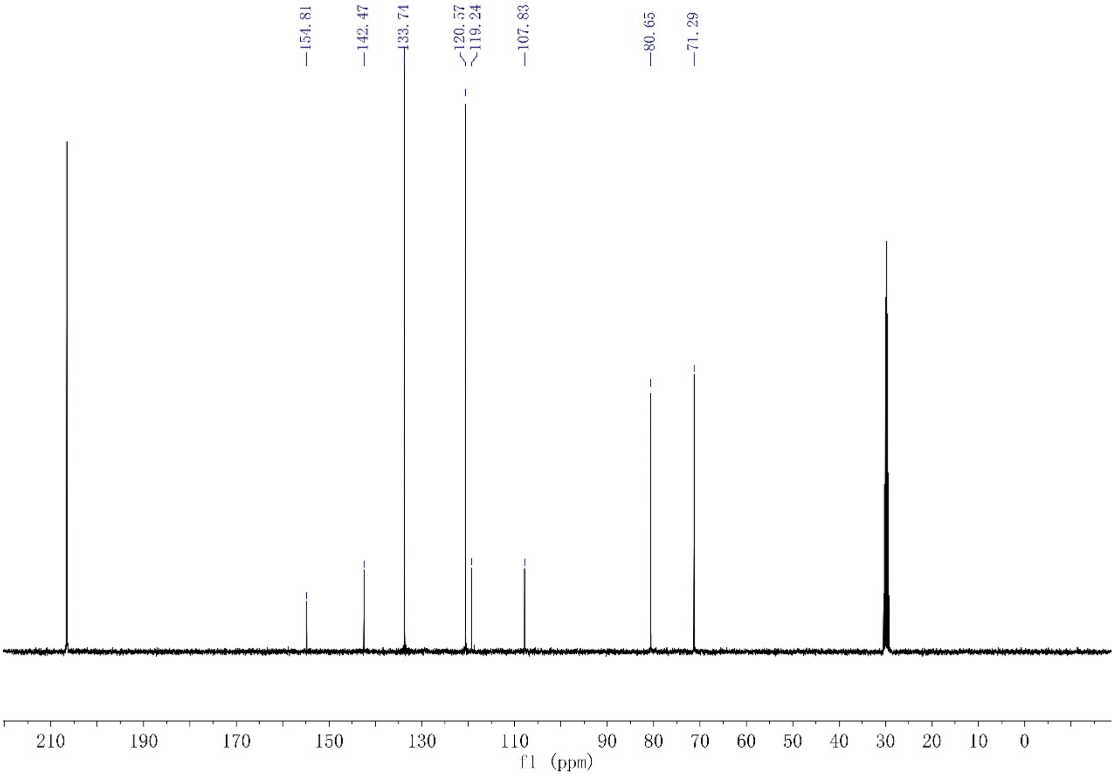

Supplementary Fig. 63. ^{13}C NMR spectrum of compound 2a.

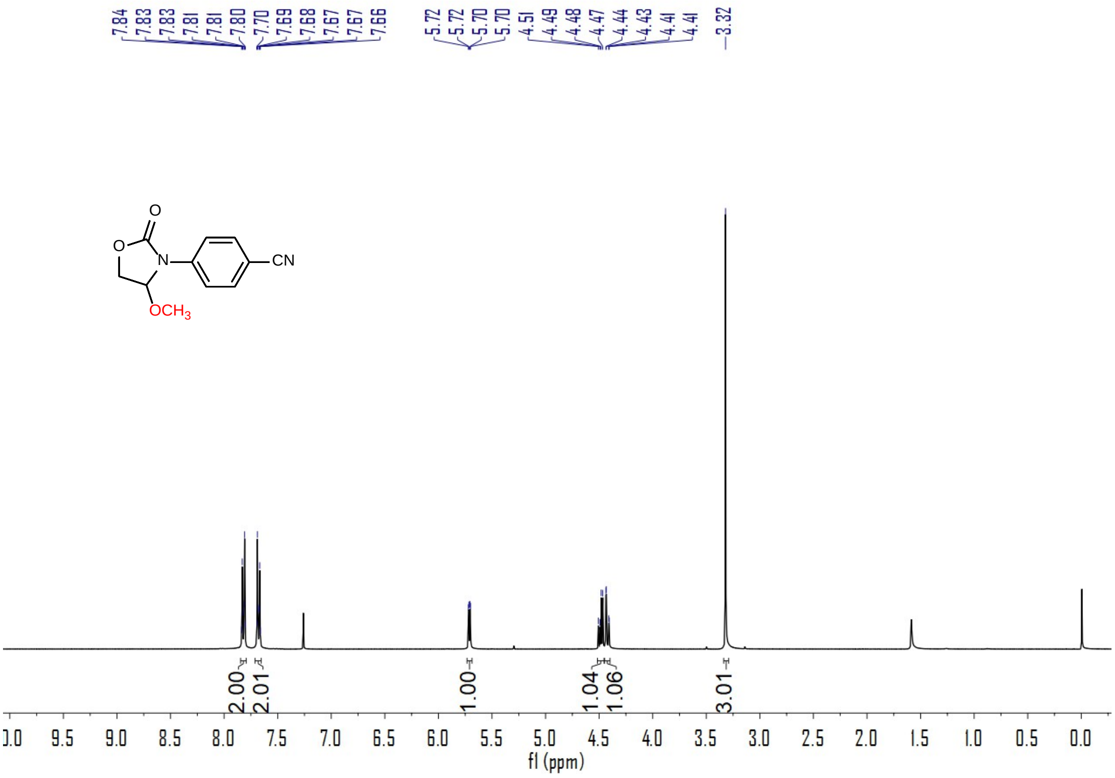

Supplementary Fig. 64. ^{1}H NMR spectrum of compound 2aa.

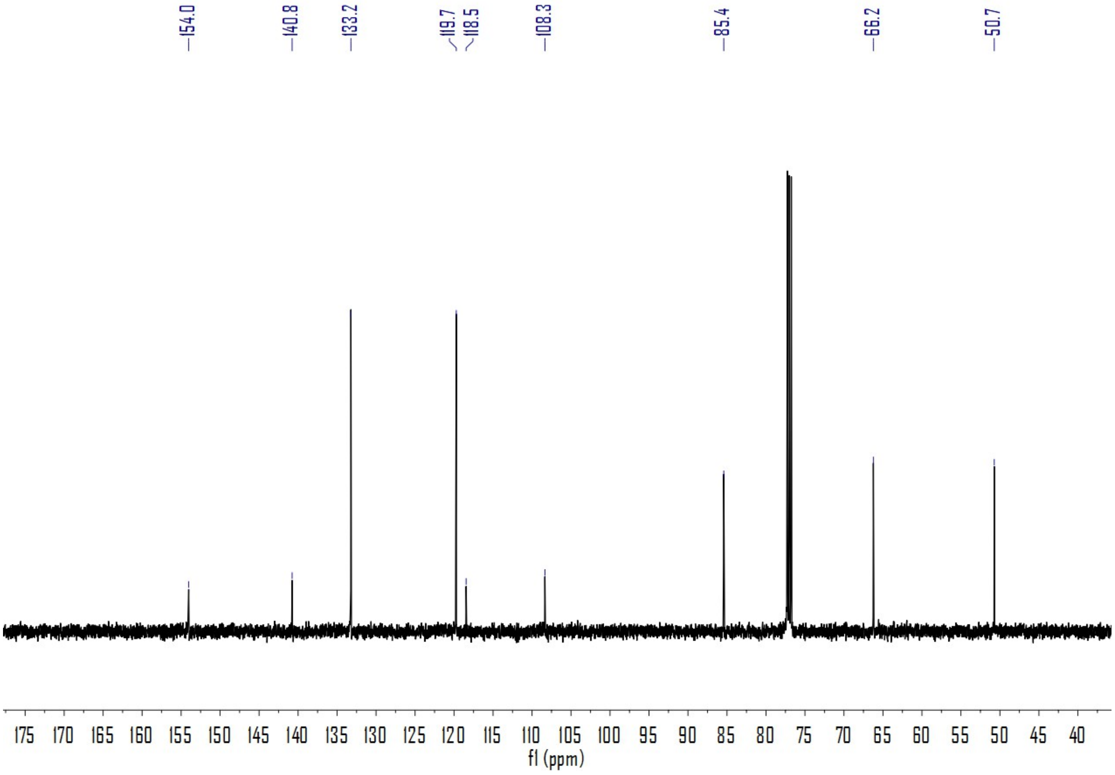

Supplementary Fig. 65. ^{13}C NMR spectrum of compound 2aa.

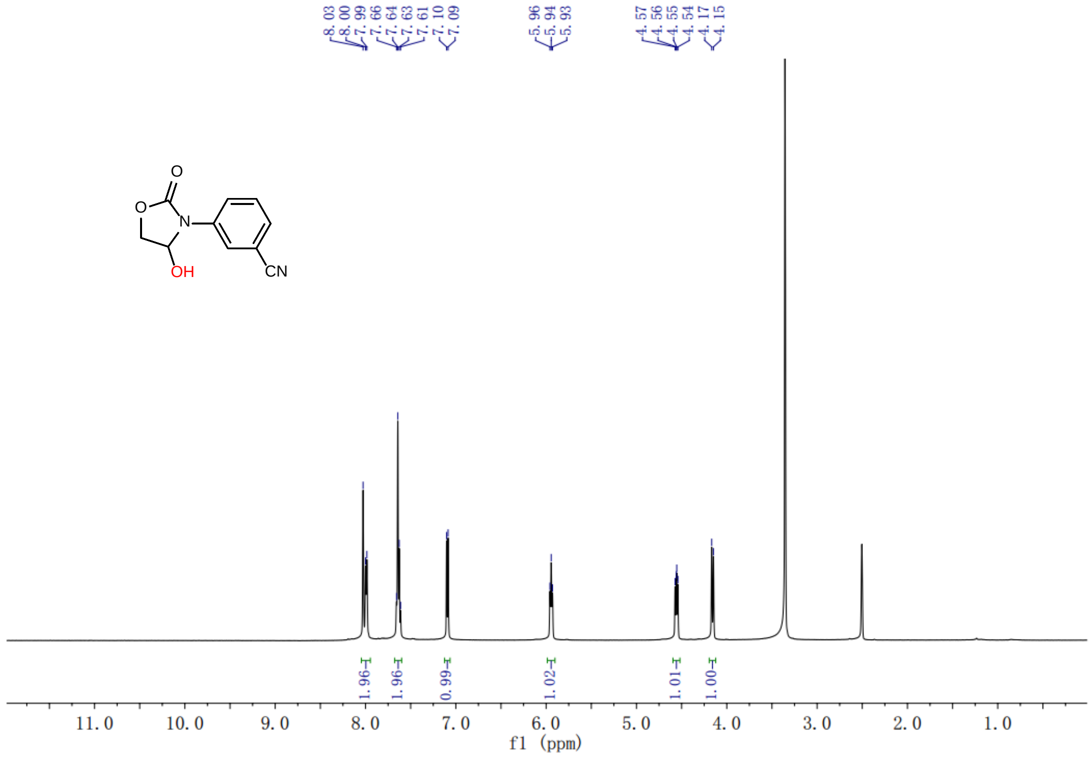

Supplementary Fig. 66. ^{1}H NMR spectrum of compound 2b.

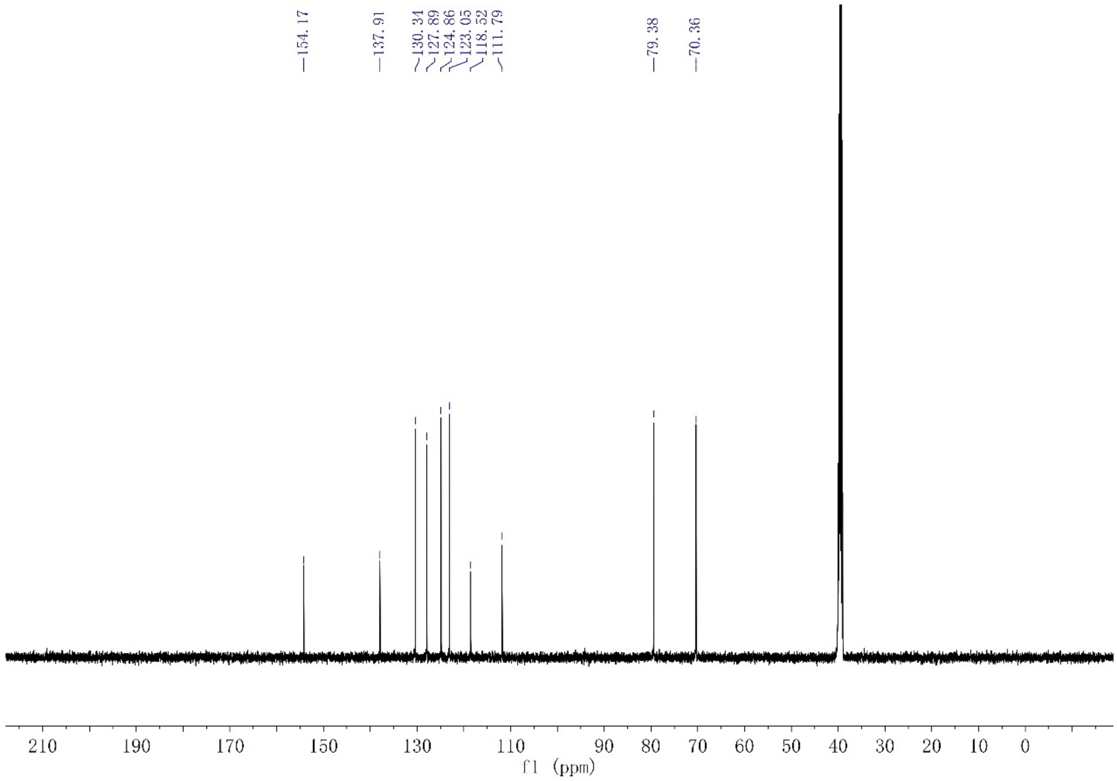

Supplementary Fig. 67. ^{13}C NMR spectrum of compound 2b.

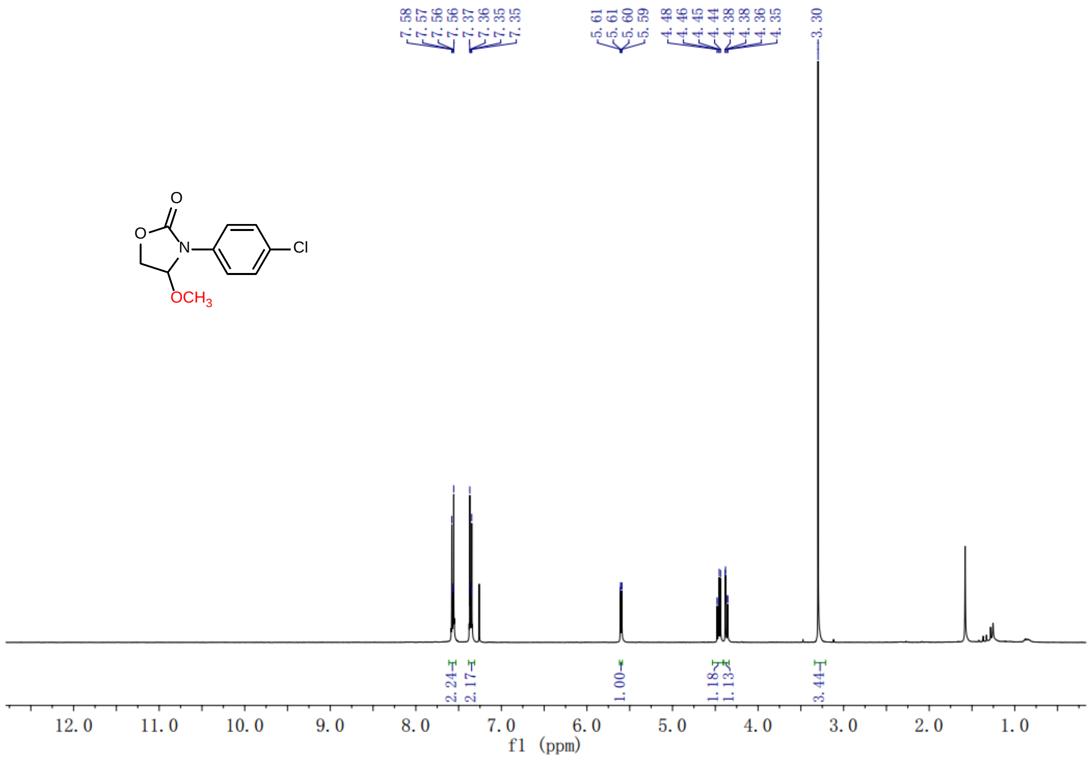

Supplementary Fig. 68. ^{1}H NMR spectrum of compound 2c.

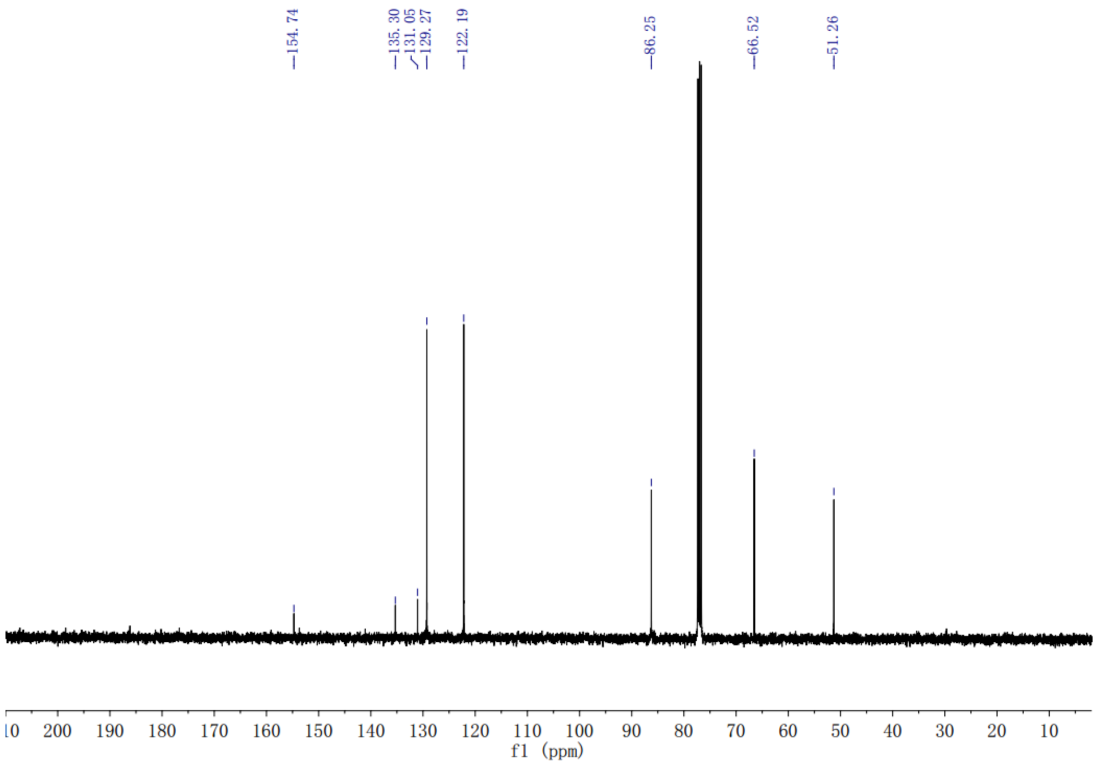

Supplementary Fig. 69. ^{13}C NMR spectrum of compound 2c.

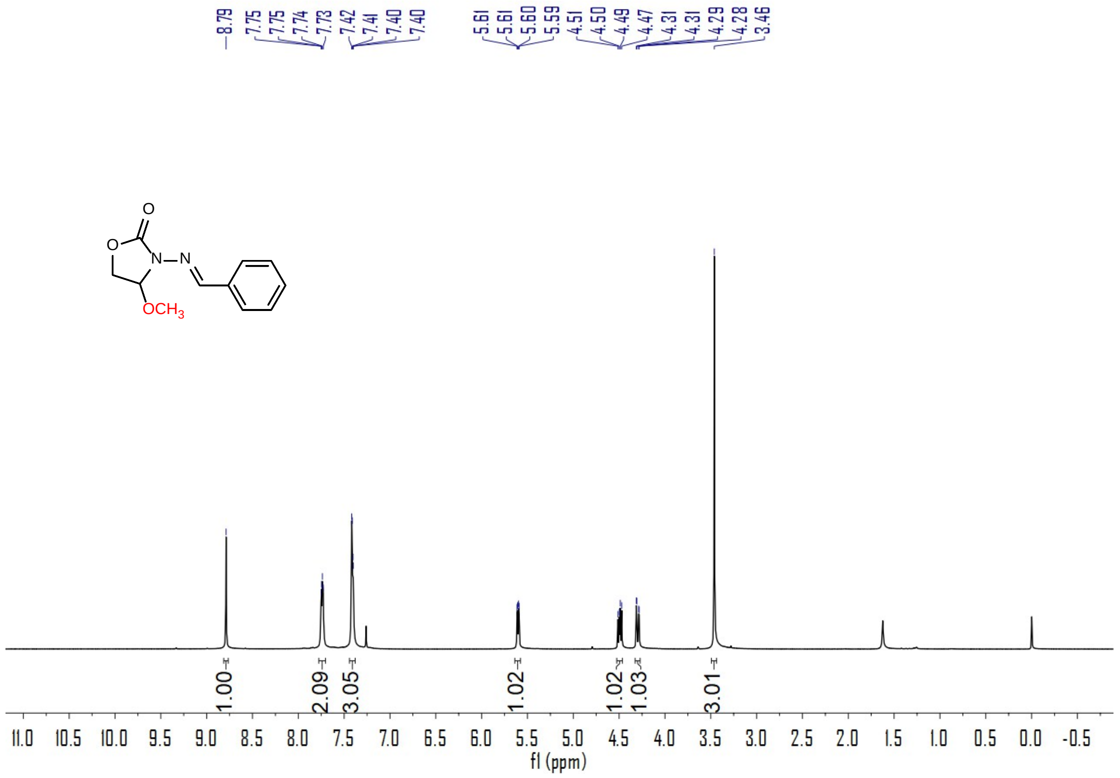

Supplementary Fig. 70. ^{1}H NMR spectrum of compound 2d.

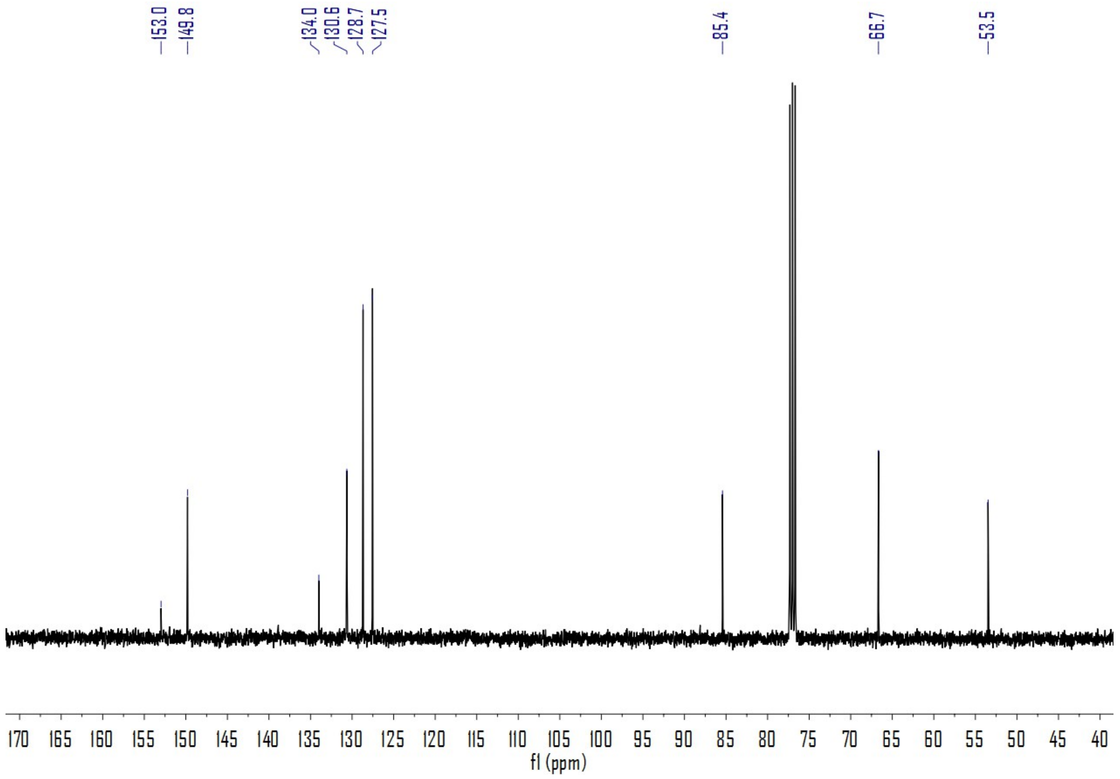

Supplementary Fig. 71. ^{13}C NMR spectrum of compound 2d.

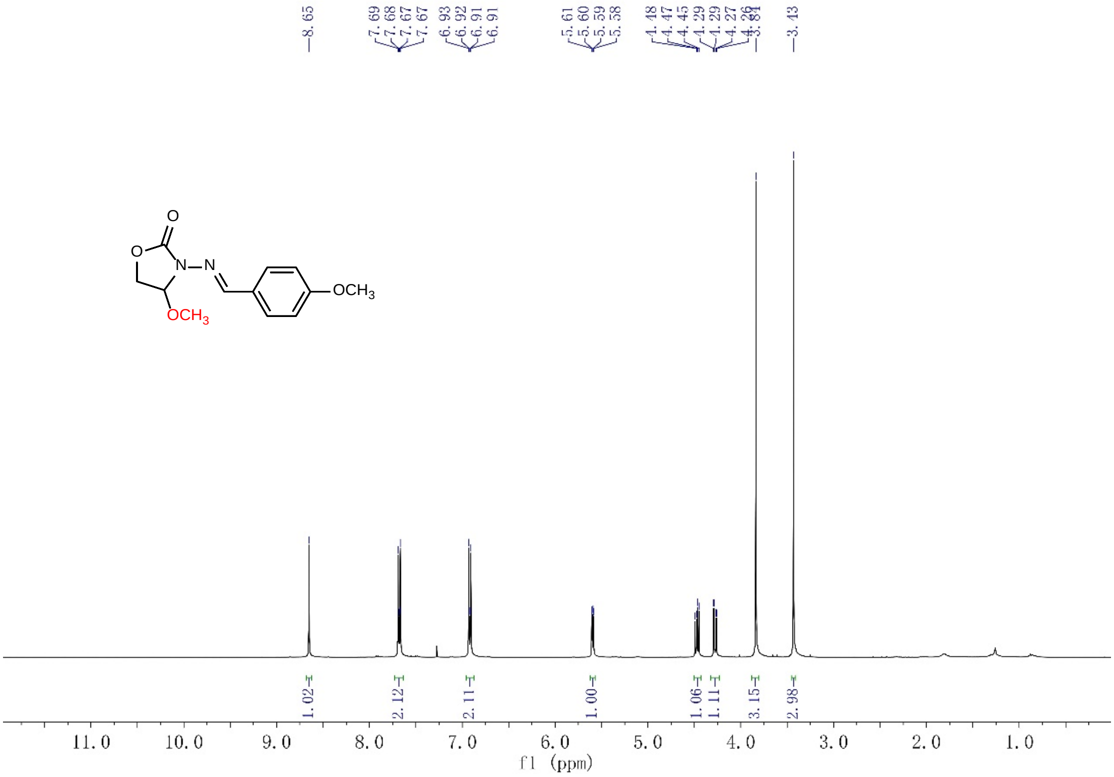

Supplementary Fig. 72. ^{1}H NMR spectrum of compound 2e.

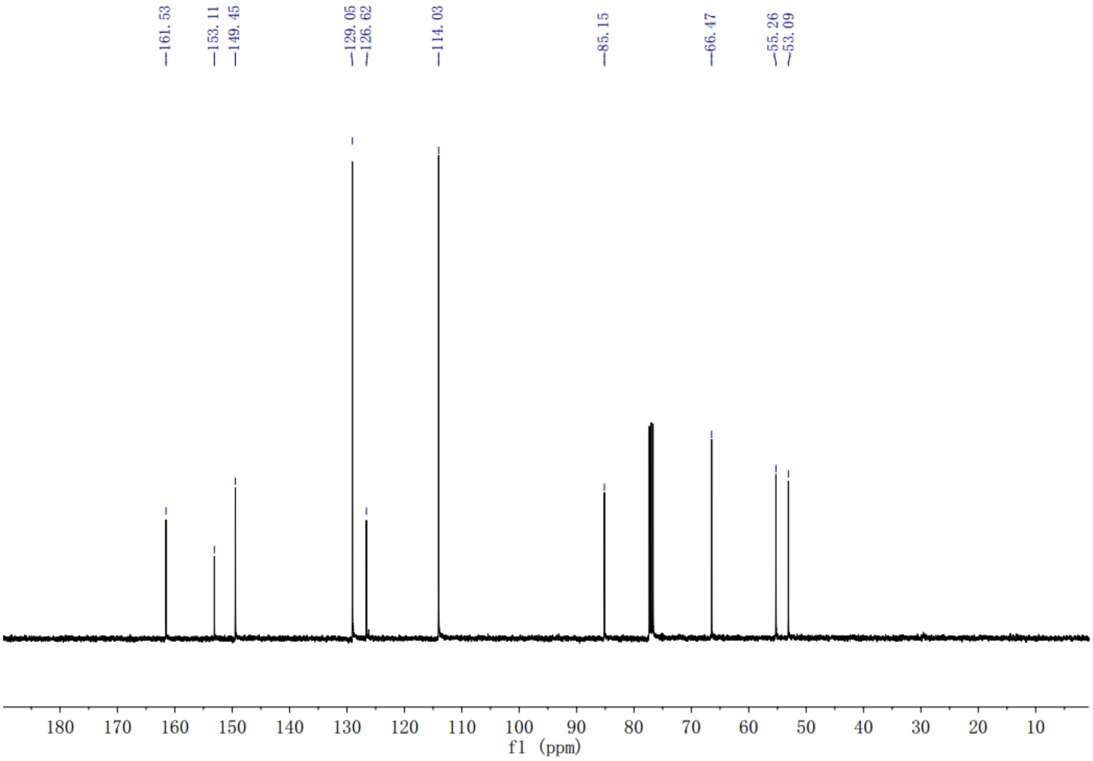

Supplementary Fig. 73. ^{13}C NMR spectrum of compound 2e.

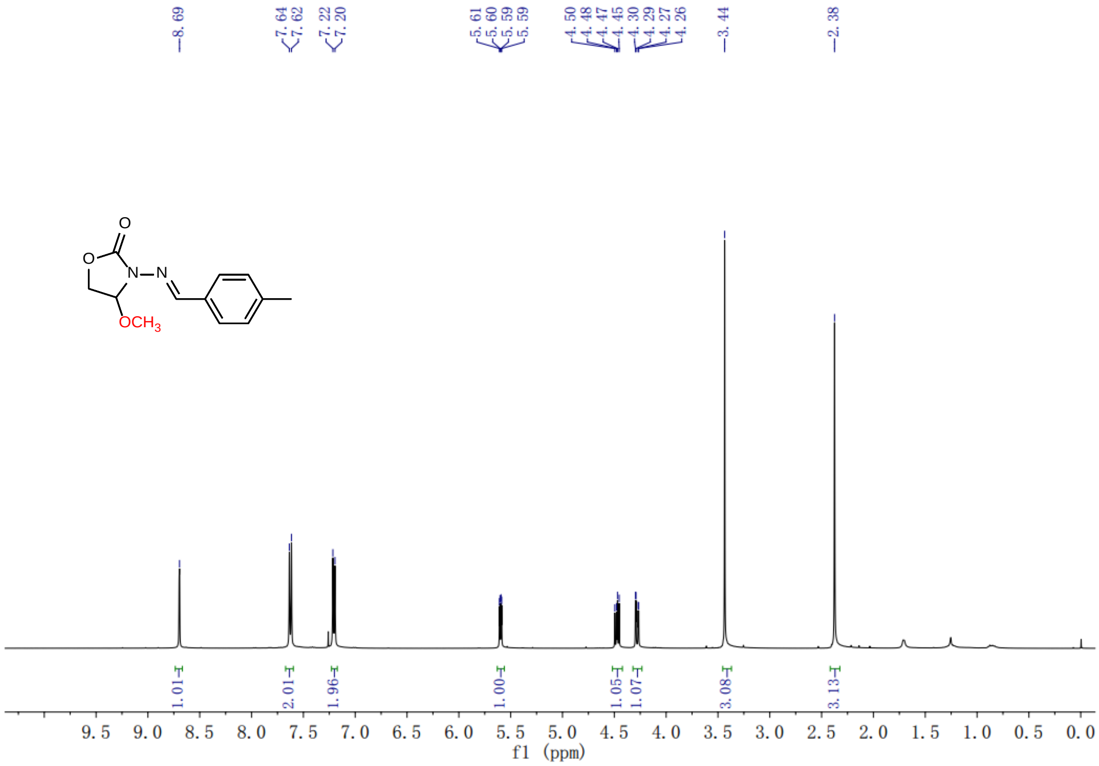

Supplementary Fig. 74. ^{1}H NMR spectrum of compound 2f.

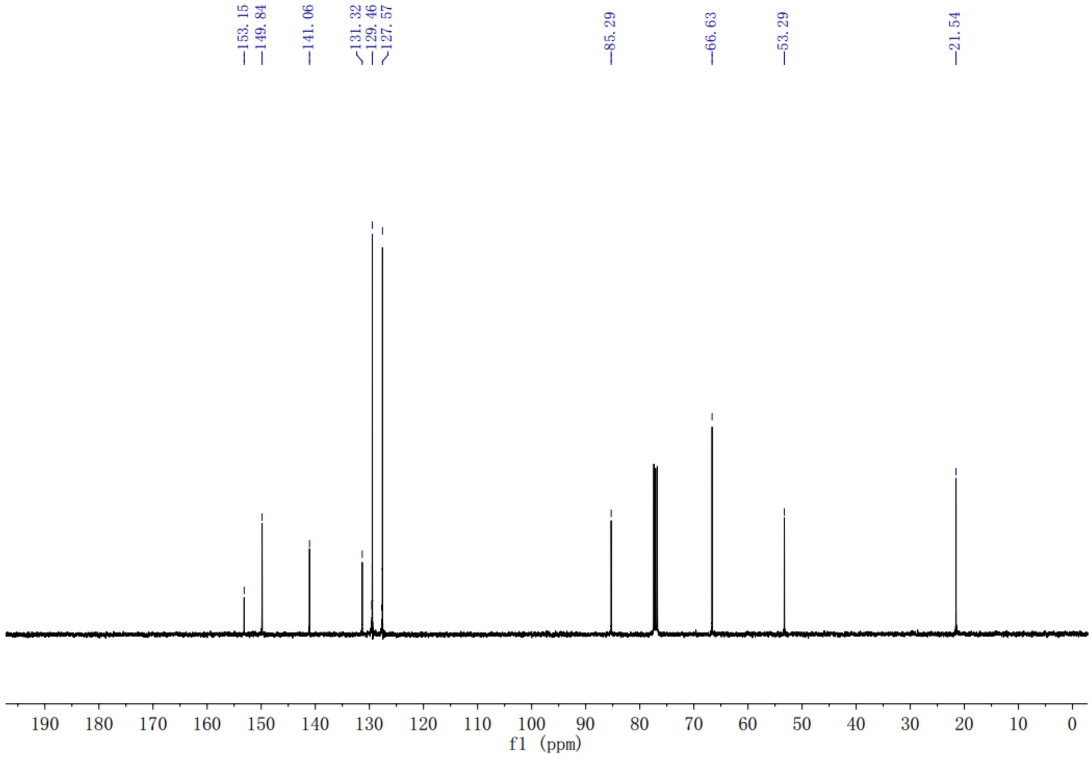

Supplementary Fig. 75. ^{13}C NMR spectrum of compound 2f.

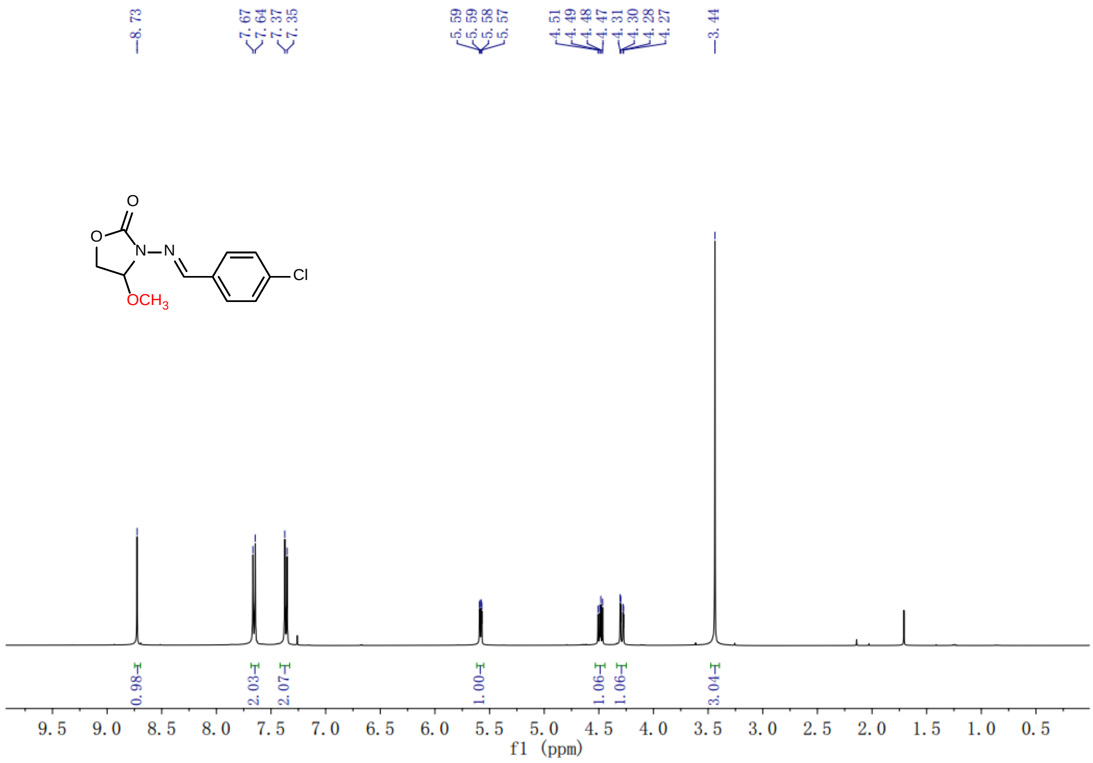

Supplementary Fig. 76. ^{1}H NMR spectrum of compound 2g.

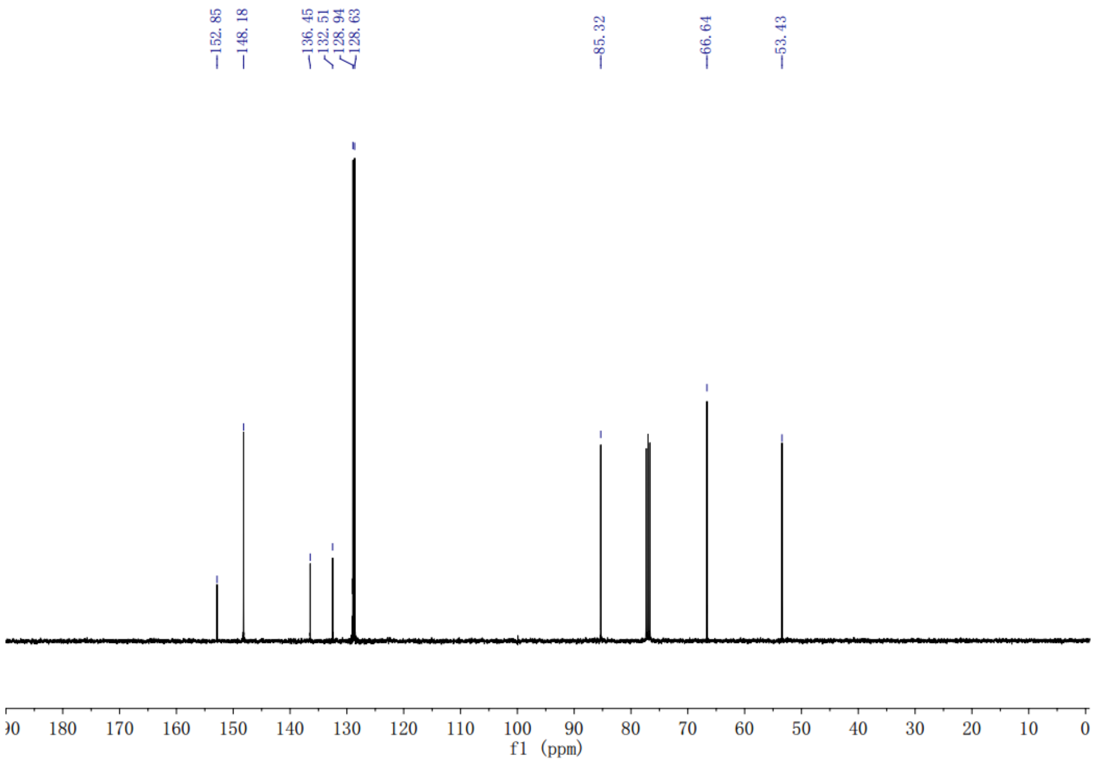

Supplementary Fig. 77. ^{13}C NMR spectrum of compound 2g.

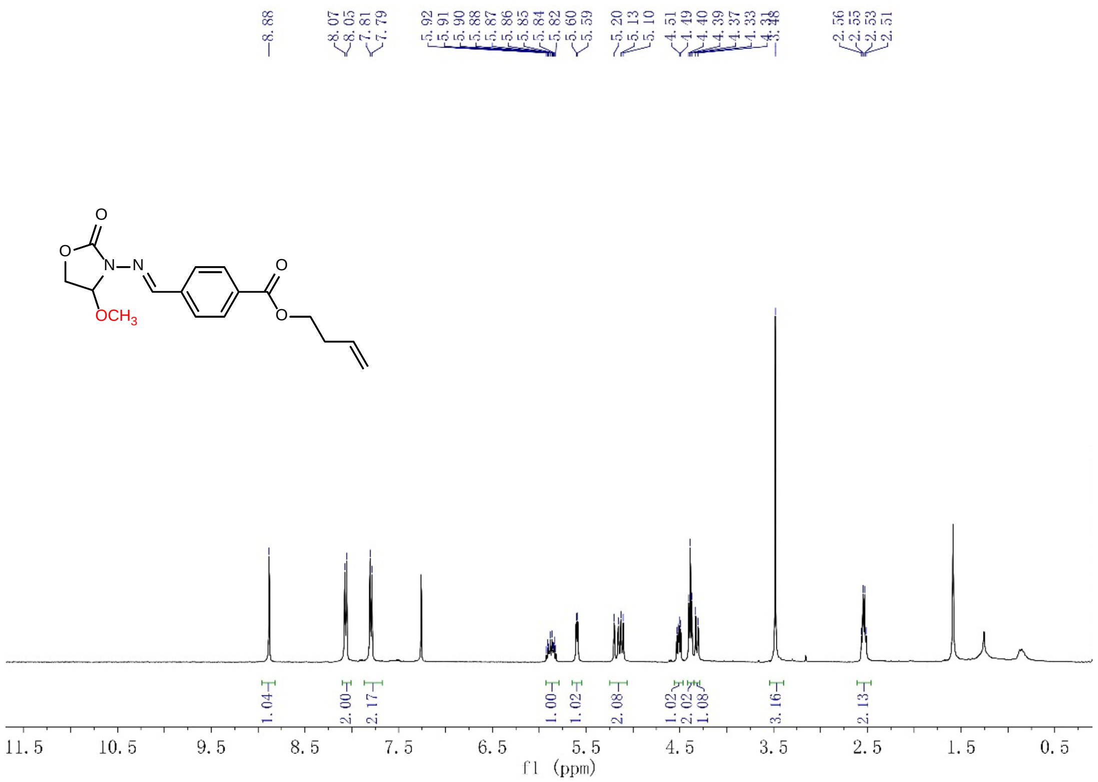

Supplementary Fig. 78. ^{1}H NMR spectrum of compound 2h.

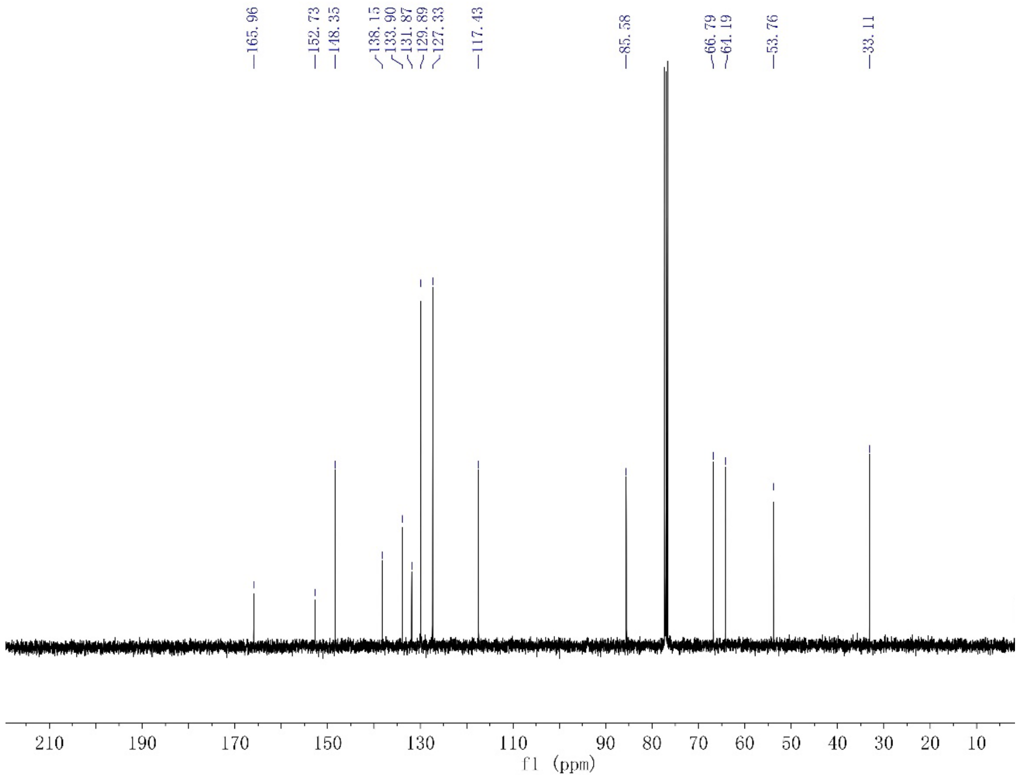

Supplementary Fig. 79. ^{13}C NMR spectrum of compound 2h.

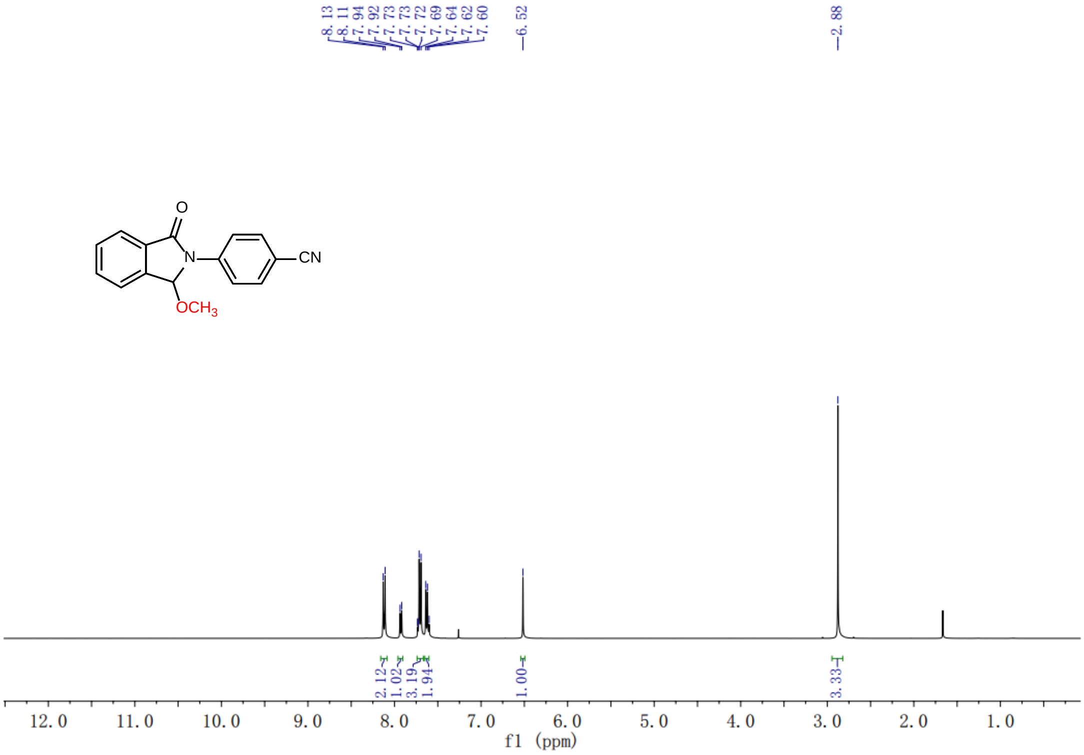

Supplementary Fig. 80. ^{1}H NMR spectrum of compound 2i.

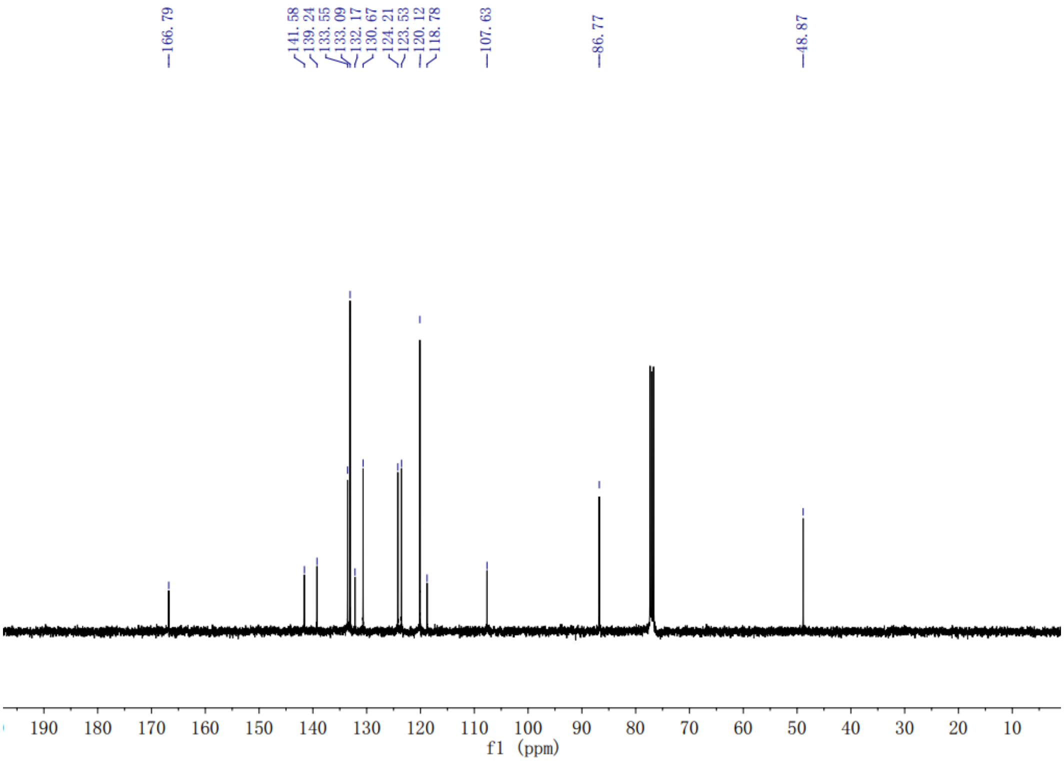

Supplementary Fig. 81. ^{13}C NMR spectrum of compound 2i.

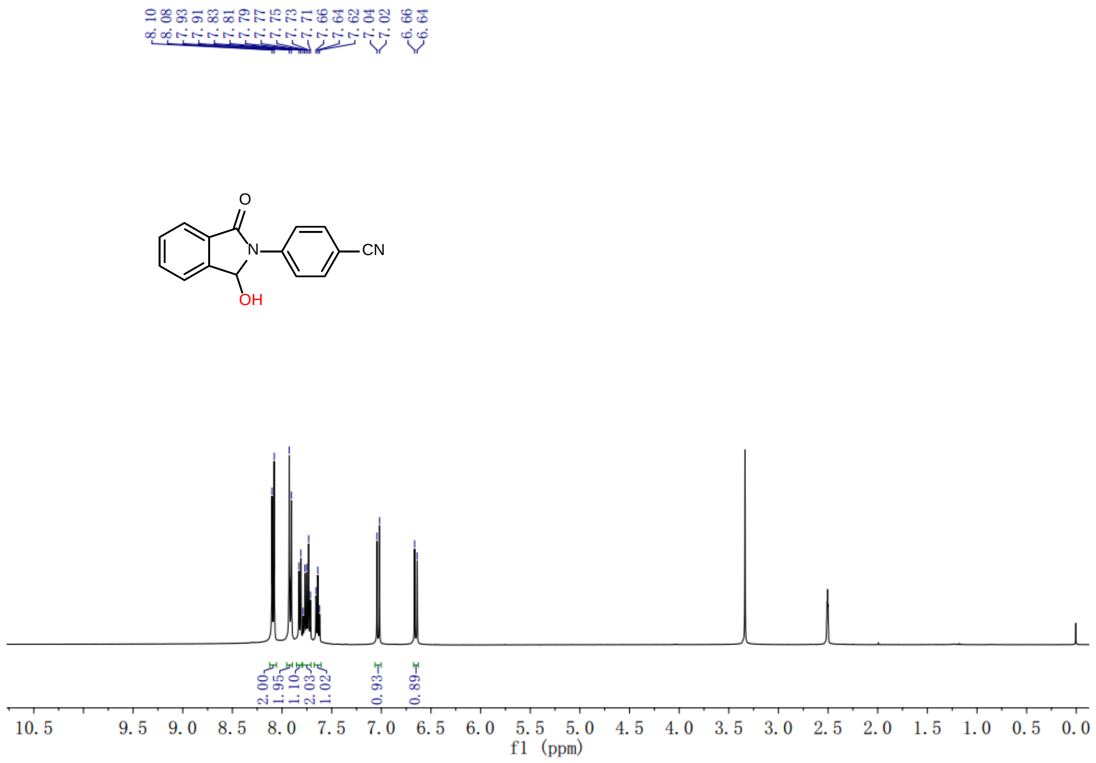

Supplementary Fig. 82. ^{1}H NMR spectrum of compound 2ia.

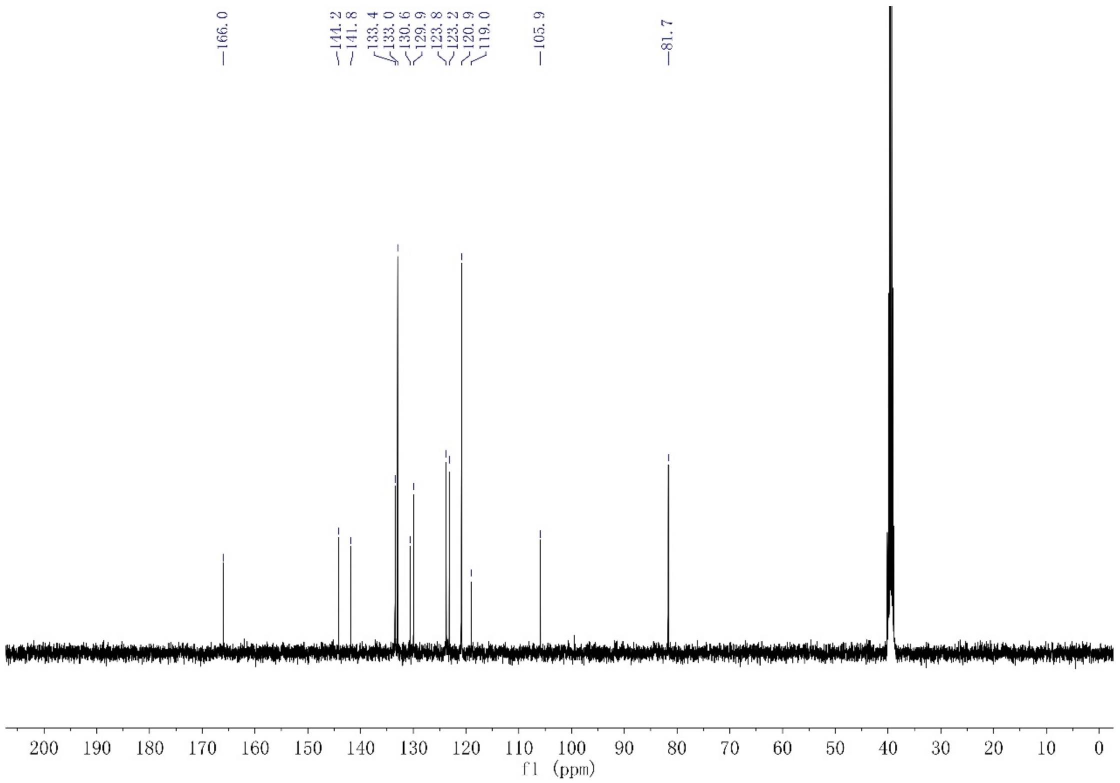

Supplementary Fig. 83. ^{13}C NMR spectrum of compound 2ia.

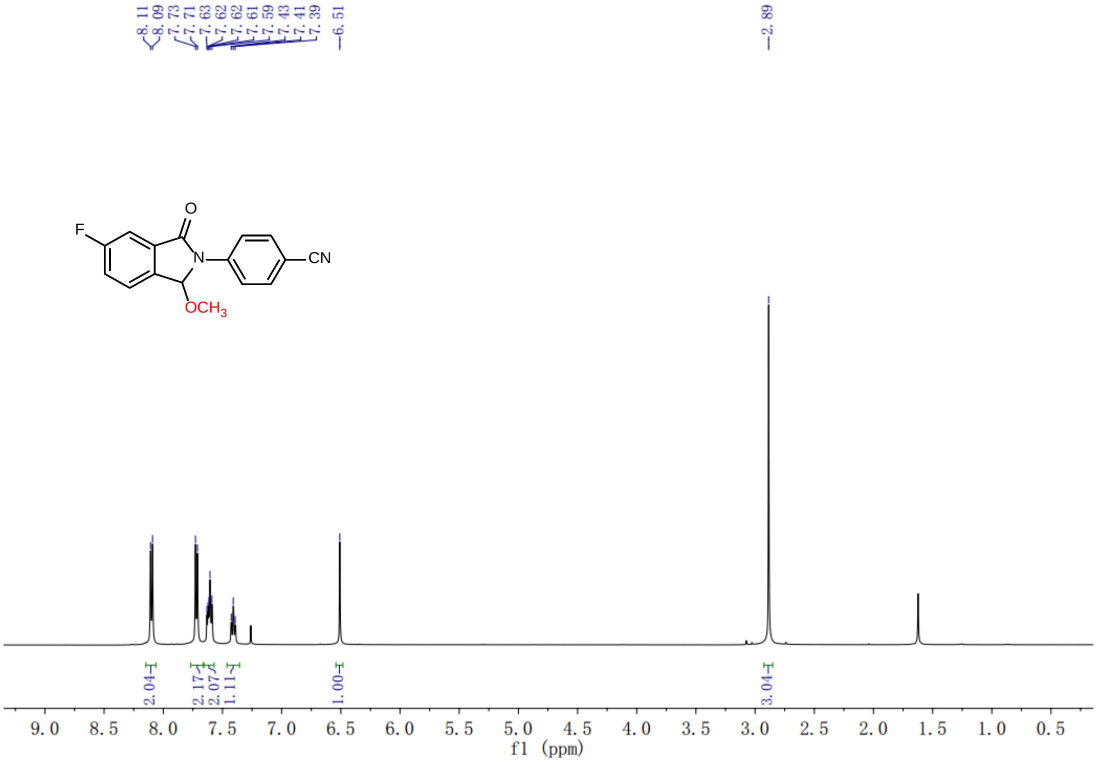

Supplementary Fig. 84. ^{1}H **NMR spectrum of compound 2j.**

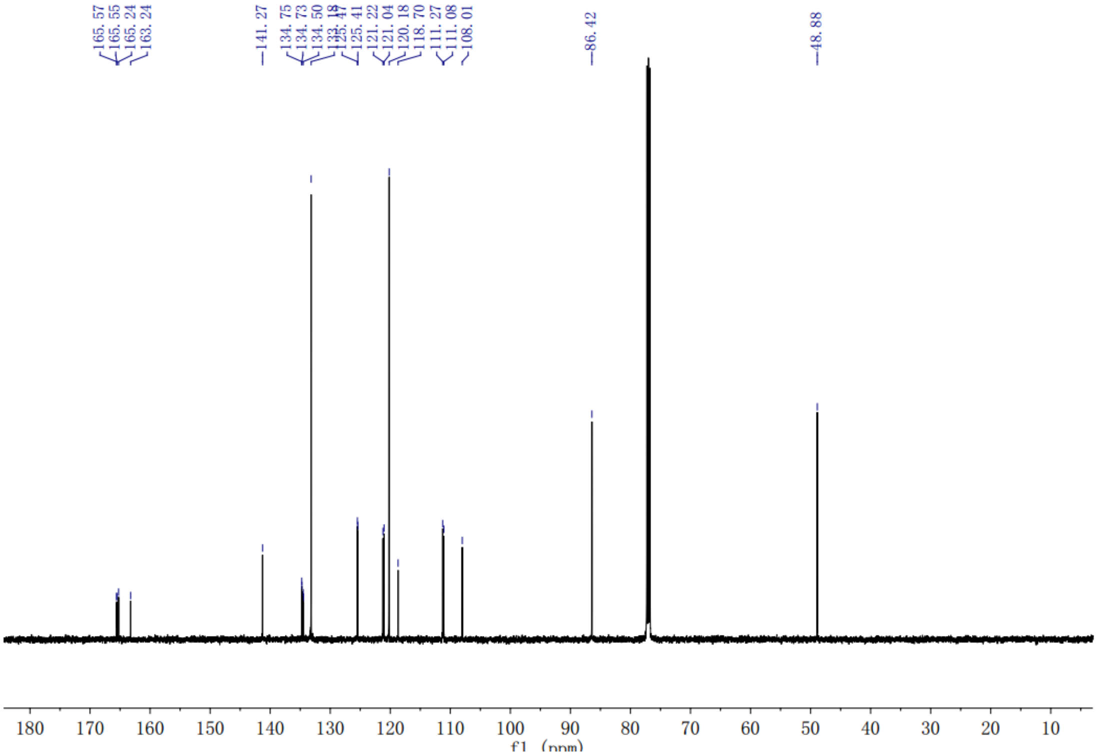

Supplementary Fig. 85. ^{13}C **NMR spectrum of compound 2j.**

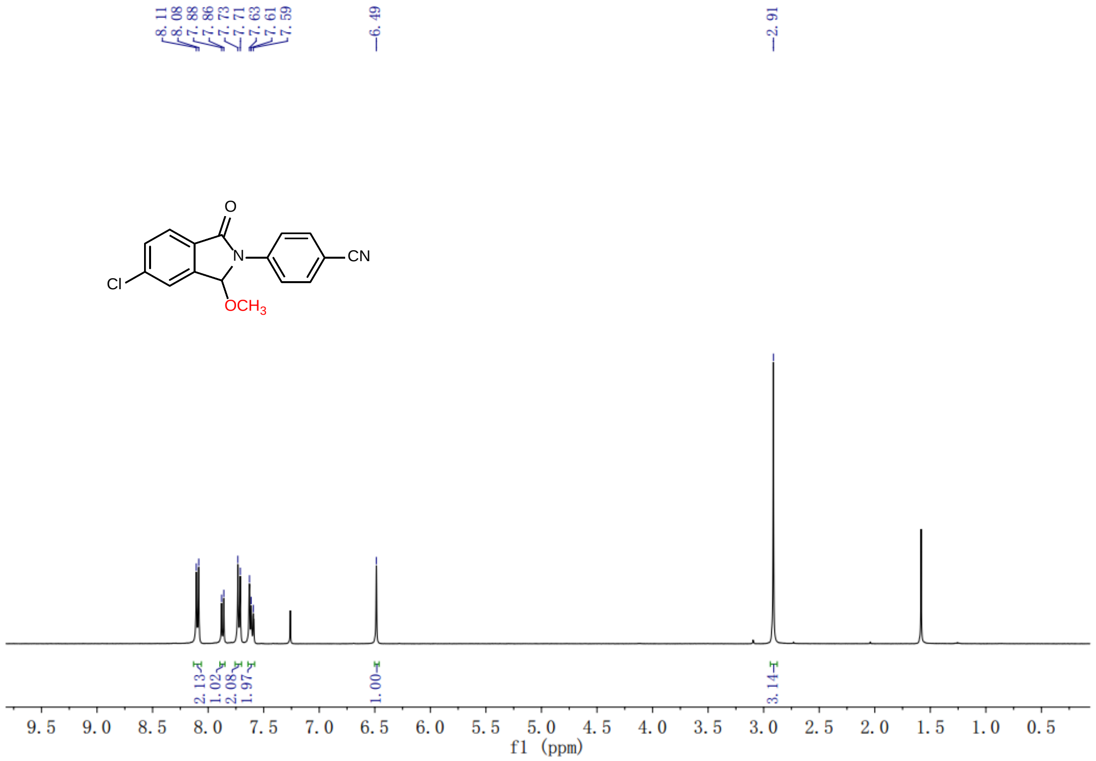

Supplementary Fig. 86. ^{1}H NMR spectrum of compound 2k.

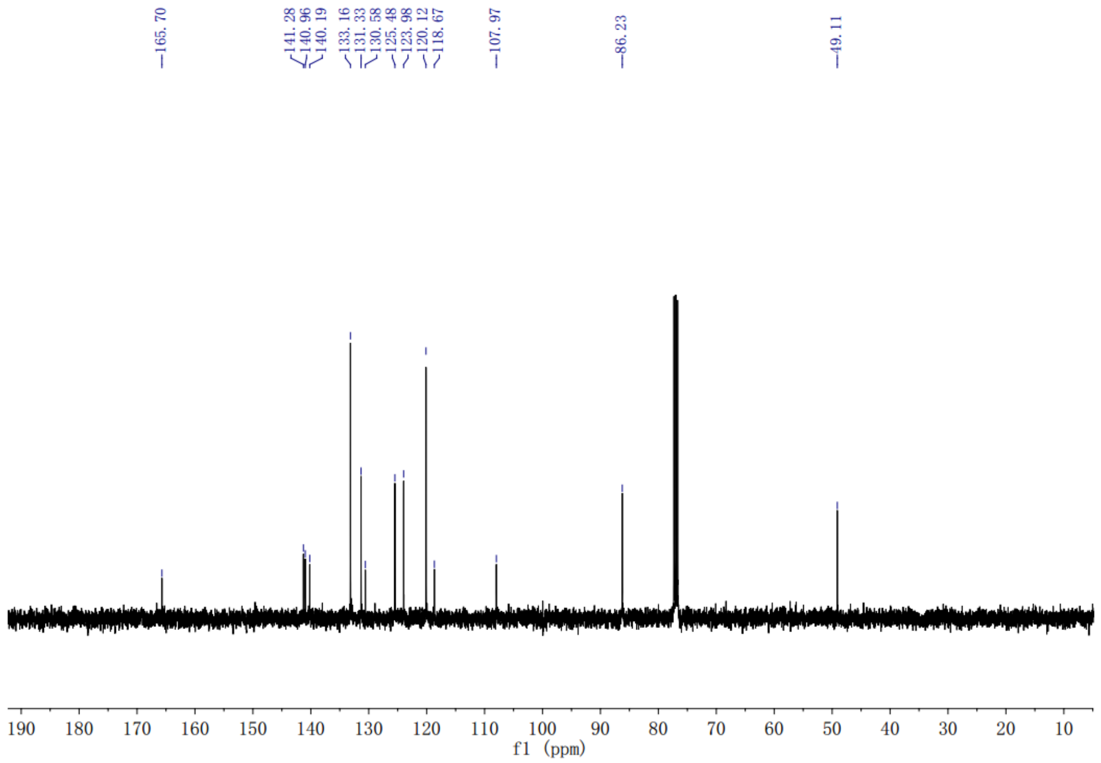

Supplementary Fig. 87. ^{13}C NMR spectrum of compound 2k.

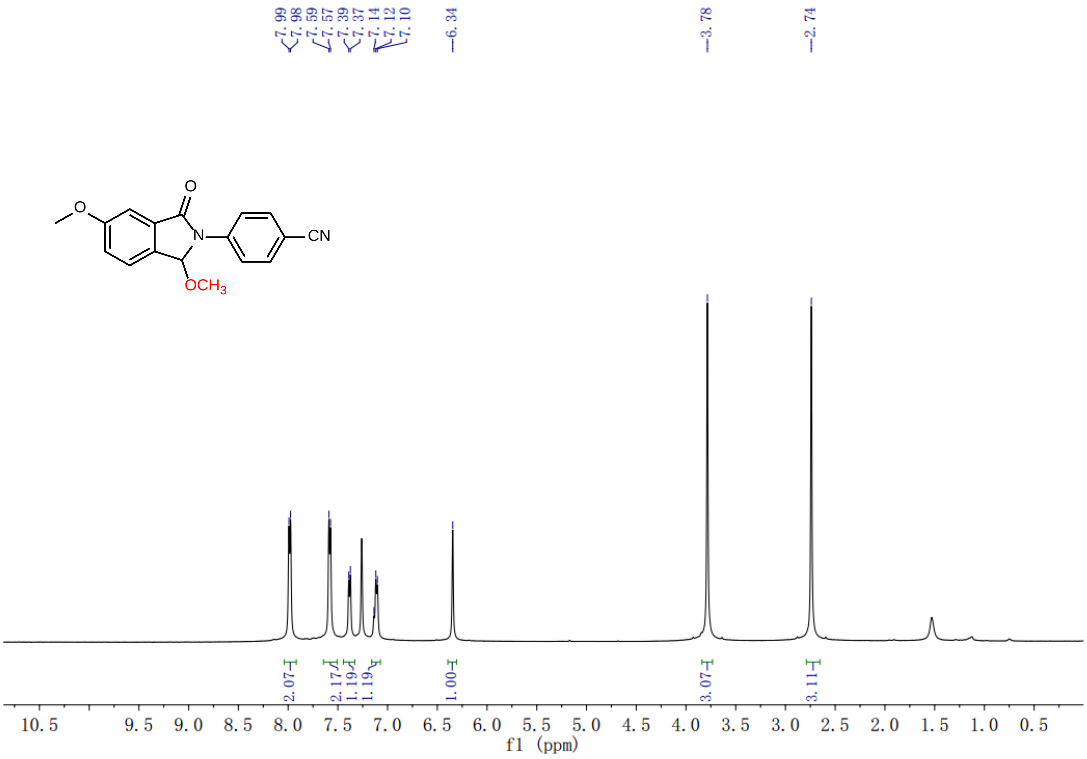

Supplementary Fig. 88. ^{1}H NMR spectrum of compound 2l.

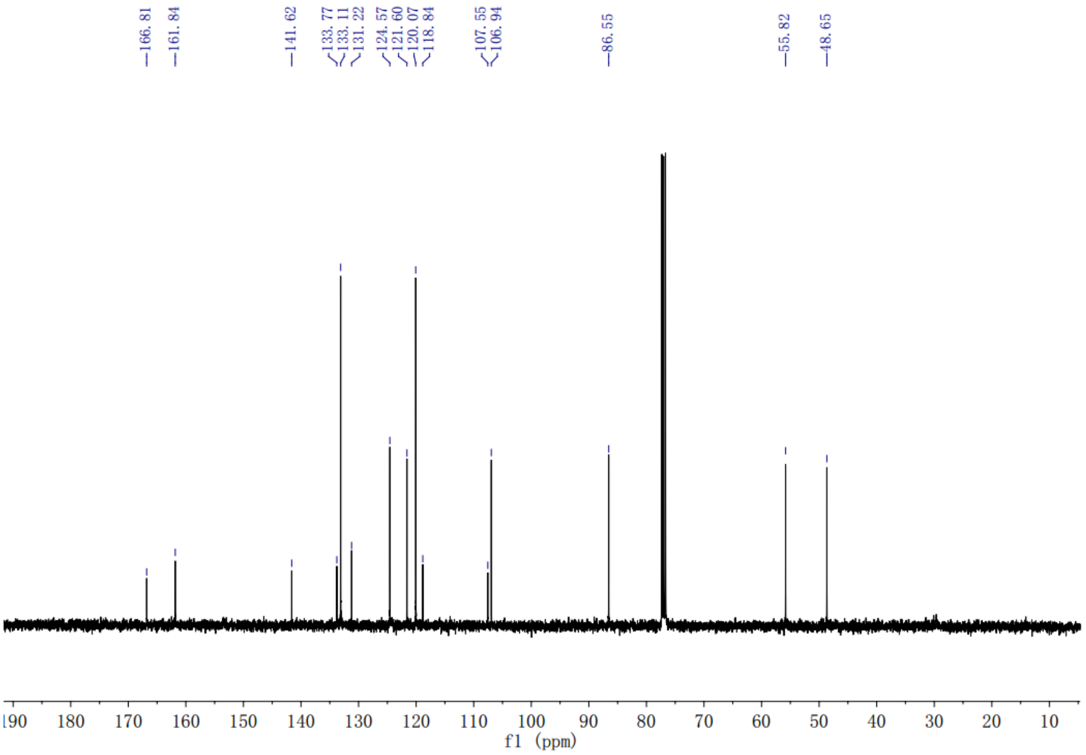

Supplementary Fig. 89. ^{13}C NMR spectrum of compound 2l.

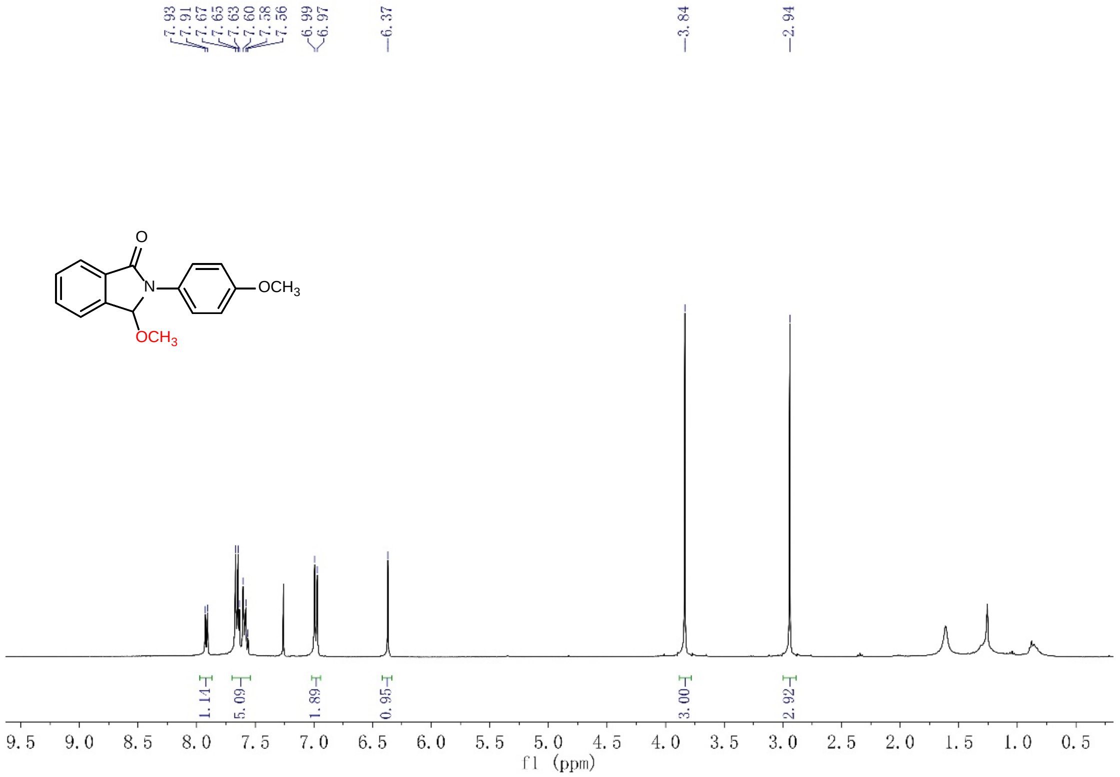

Supplementary Fig. 90. ^{1}**H NMR spectrum of compound 2m.**

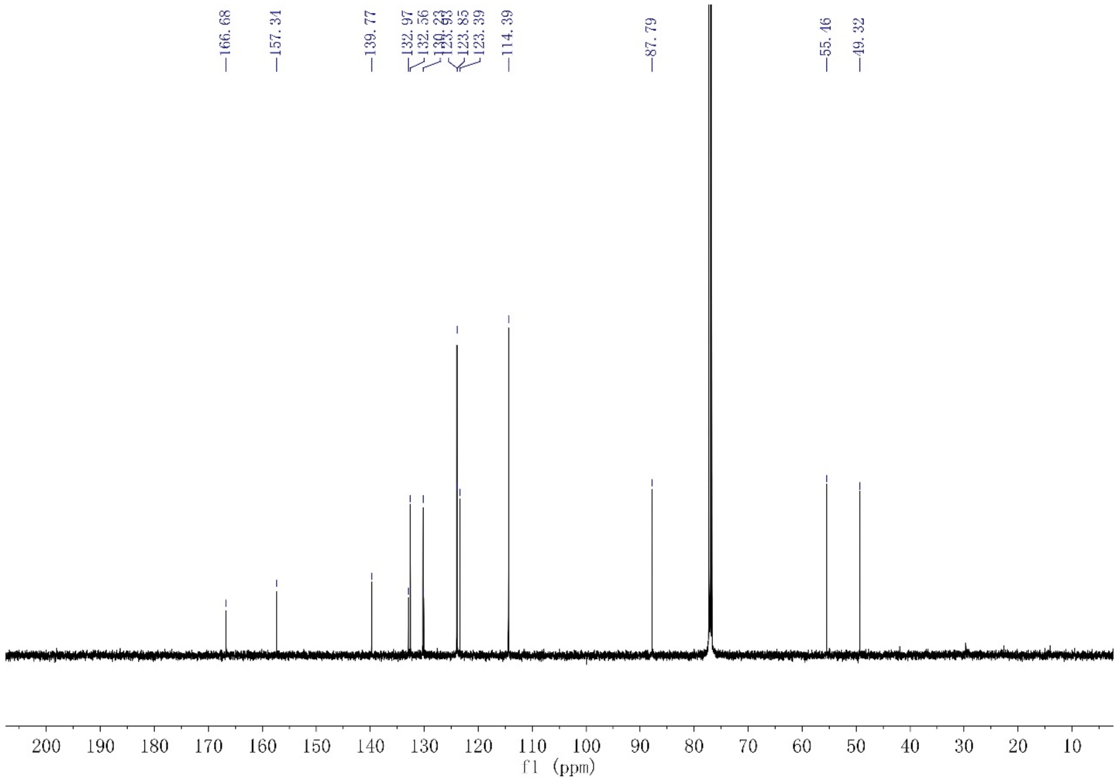

Supplementary Fig. 91. ^{13}**C NMR spectrum of compound 2m.**

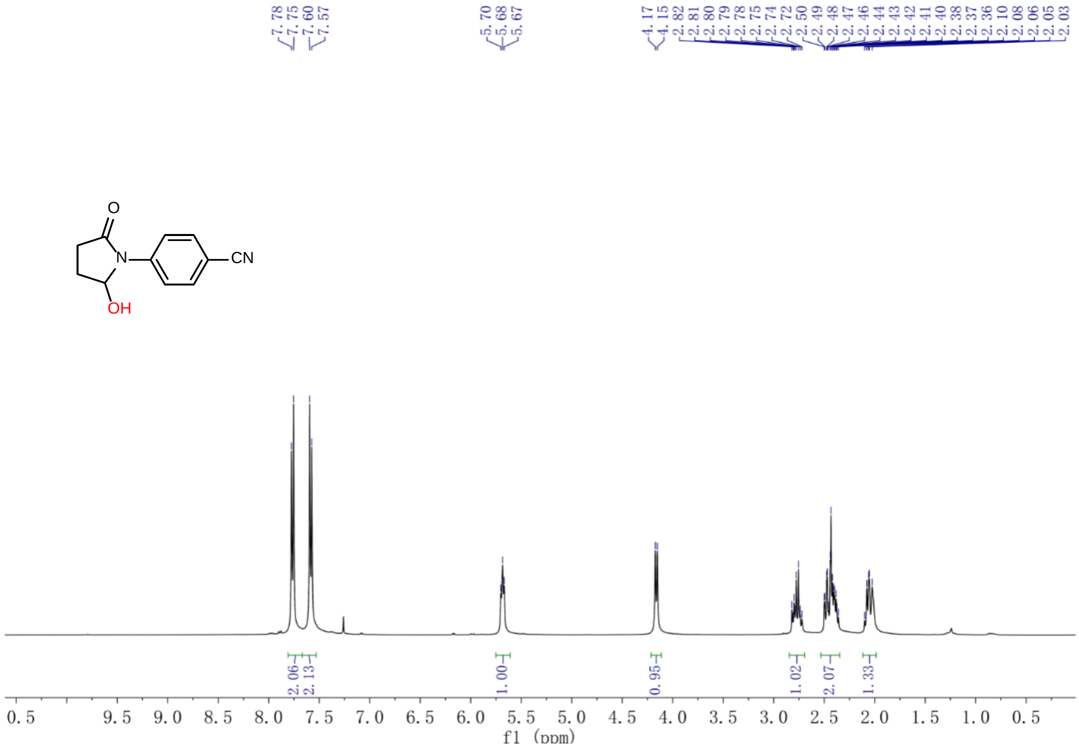

Supplementary Fig. 92. ^{1}**H NMR spectrum of compound 2n.**

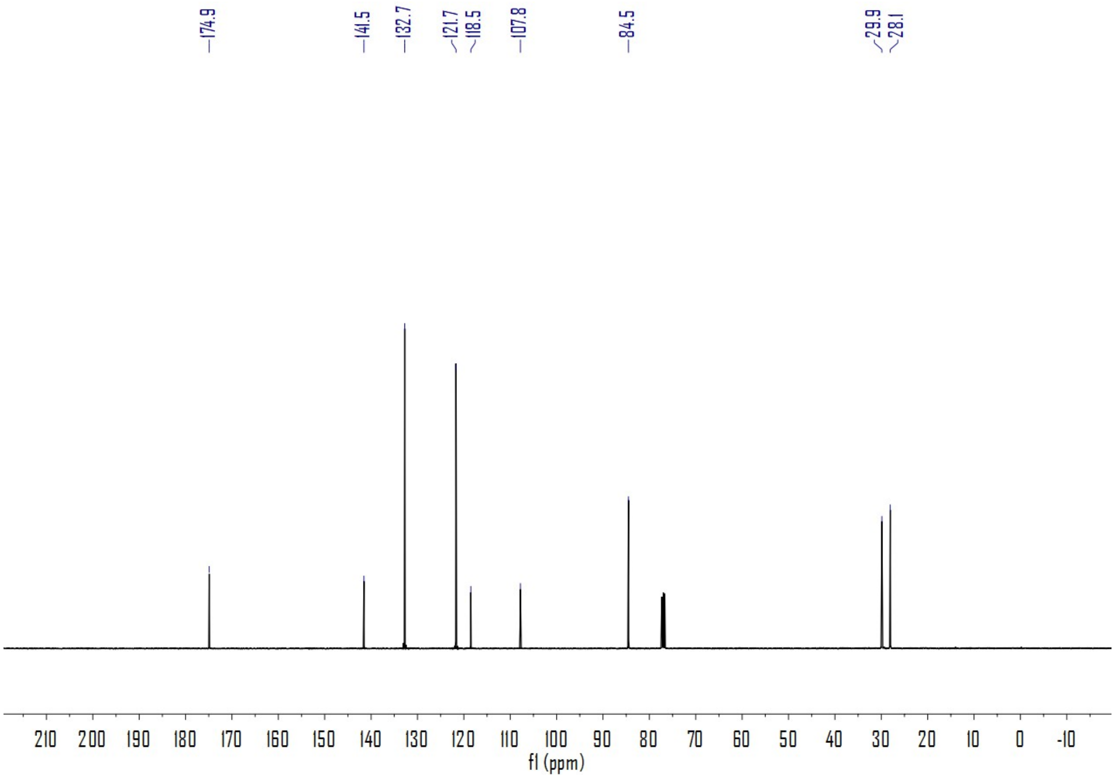

Supplementary Fig. 93. ^{13}**C NMR spectrum of compound 2n.**

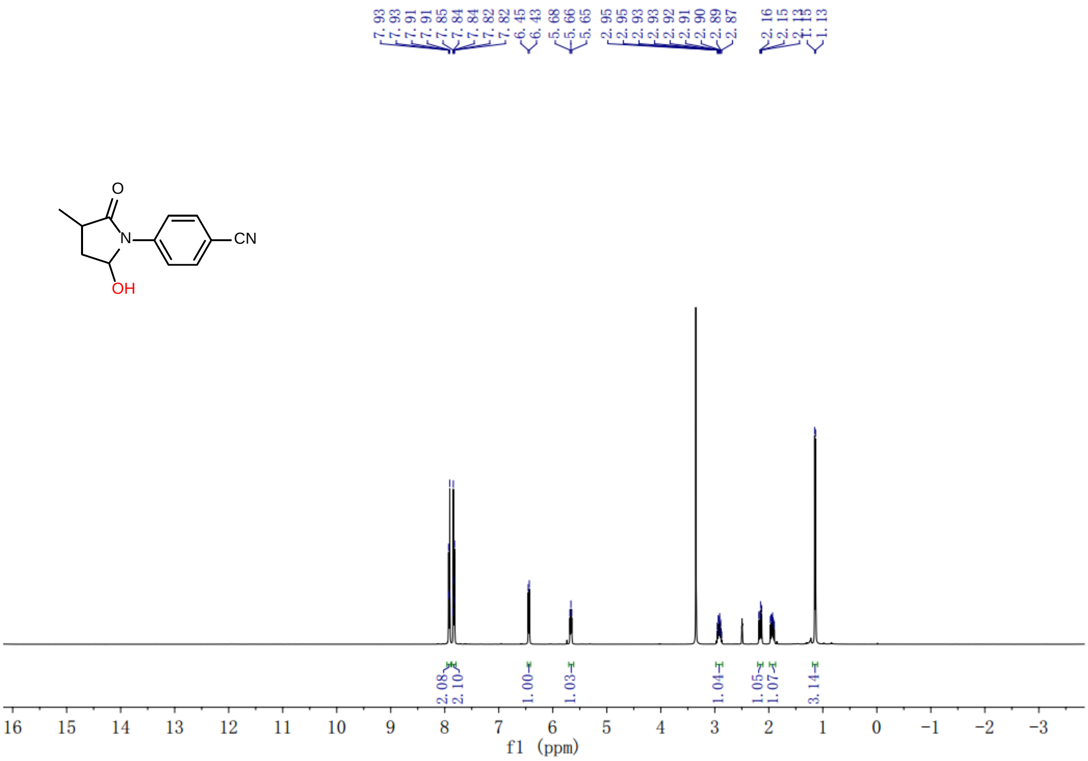

Supplementary Fig. 94. ^{1}**H NMR spectrum of compound 2o.**

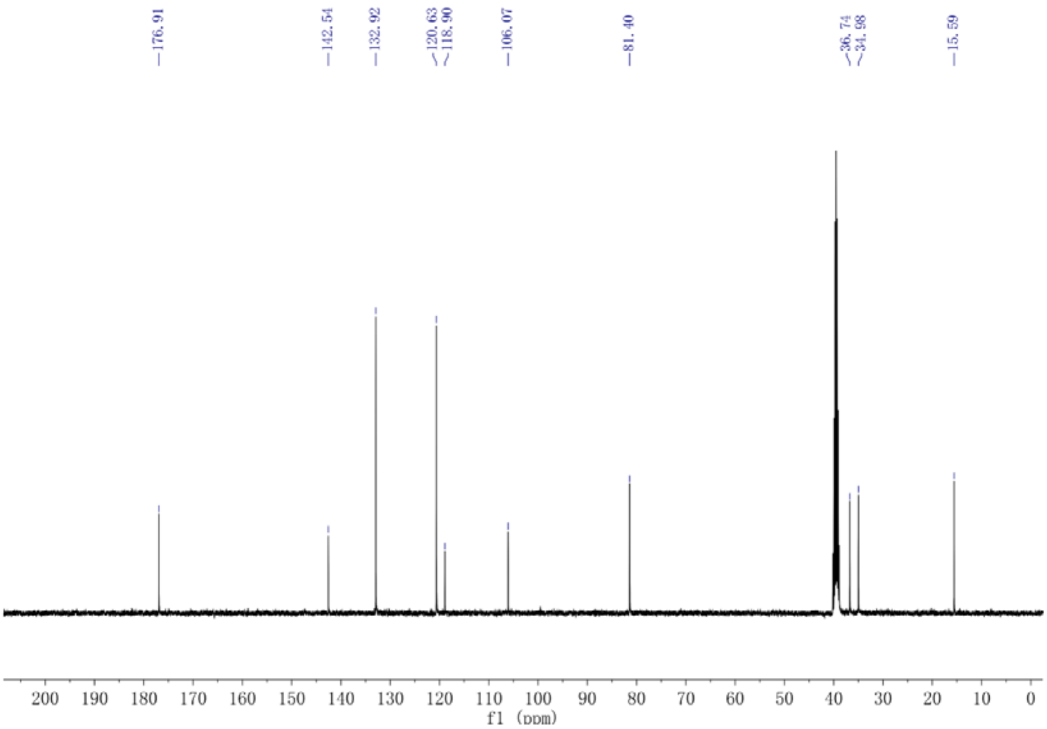

Supplementary Fig. 95. ^{13}**C NMR spectrum of compound 2o.**

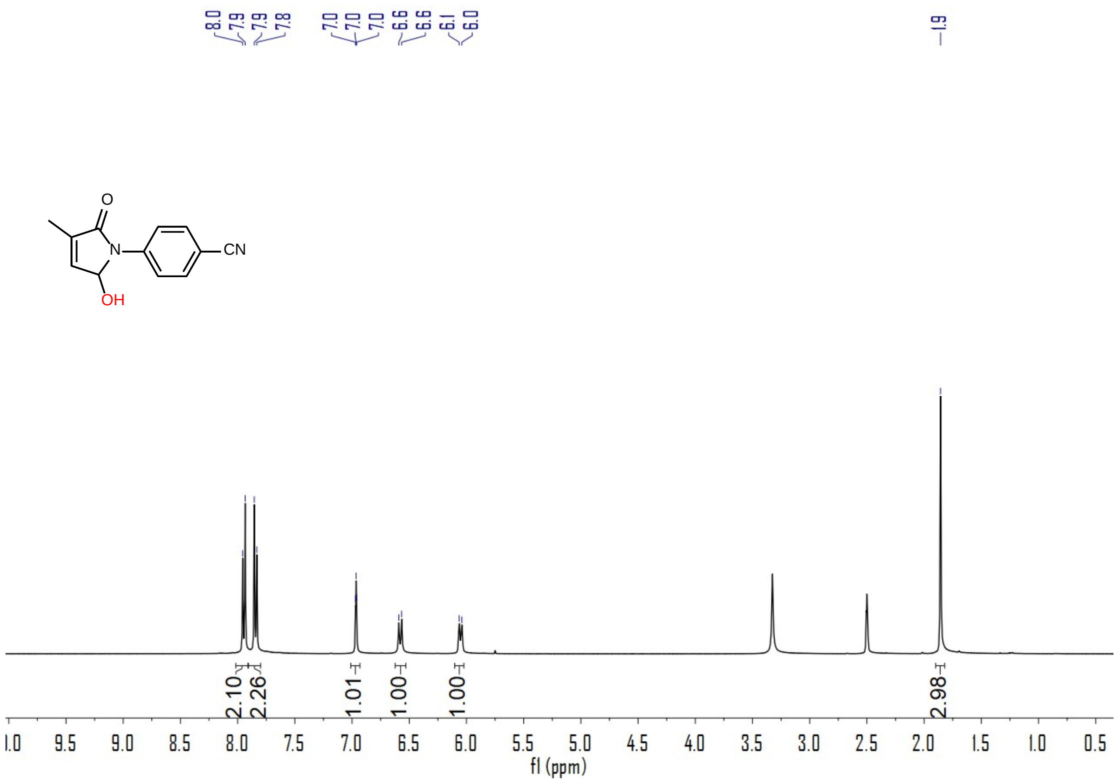

Supplementary Fig. 96. ^{1}H NMR spectrum of compound 2p.

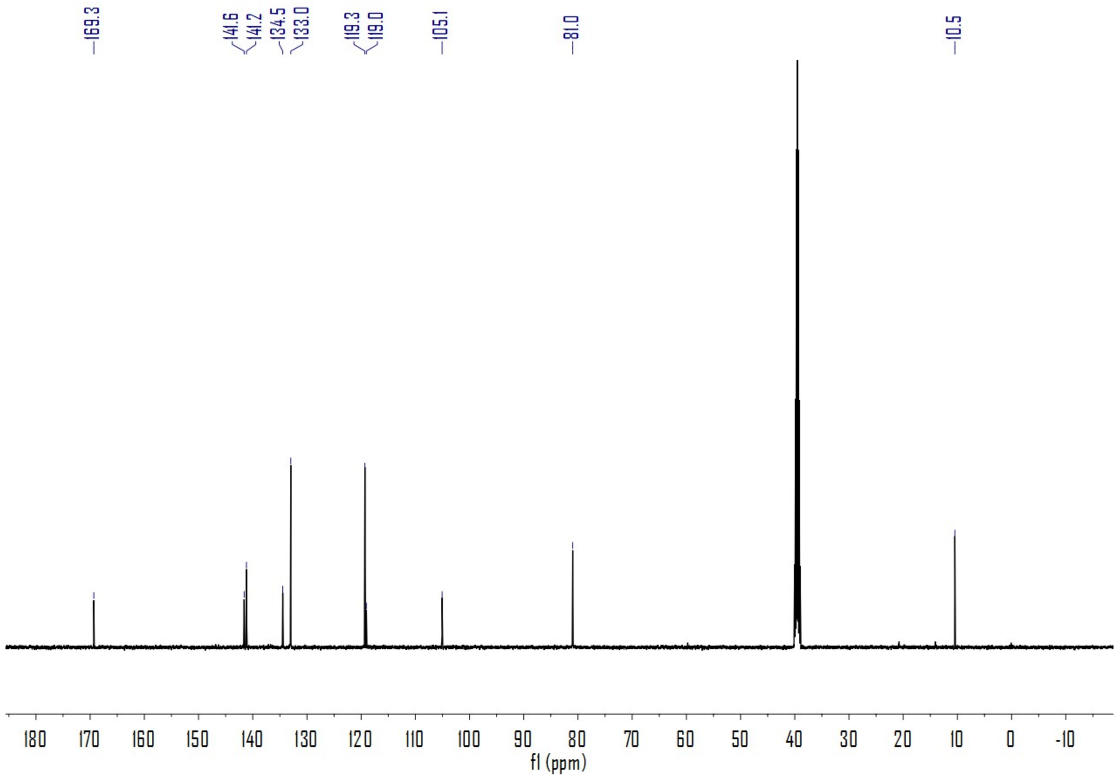

Supplementary Fig. 97. ^{13}C NMR spectrum of compound 2p.

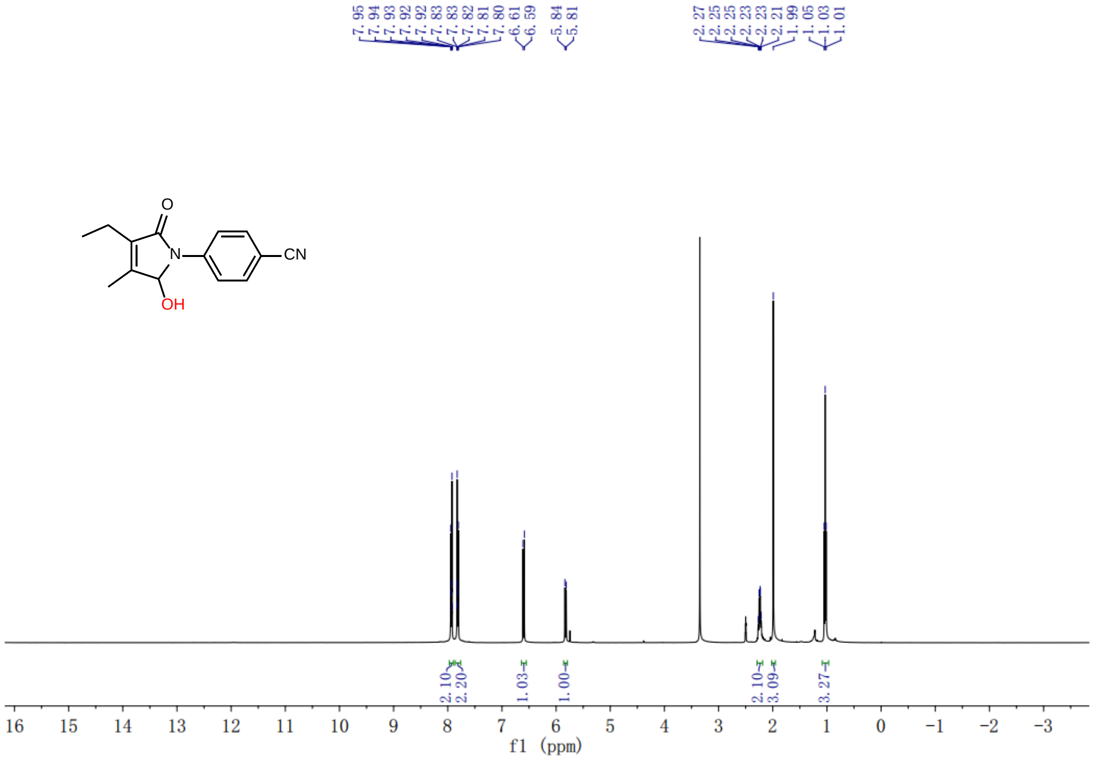

Supplementary Fig. 98. ^{1}**H NMR spectrum of compound 2q.**

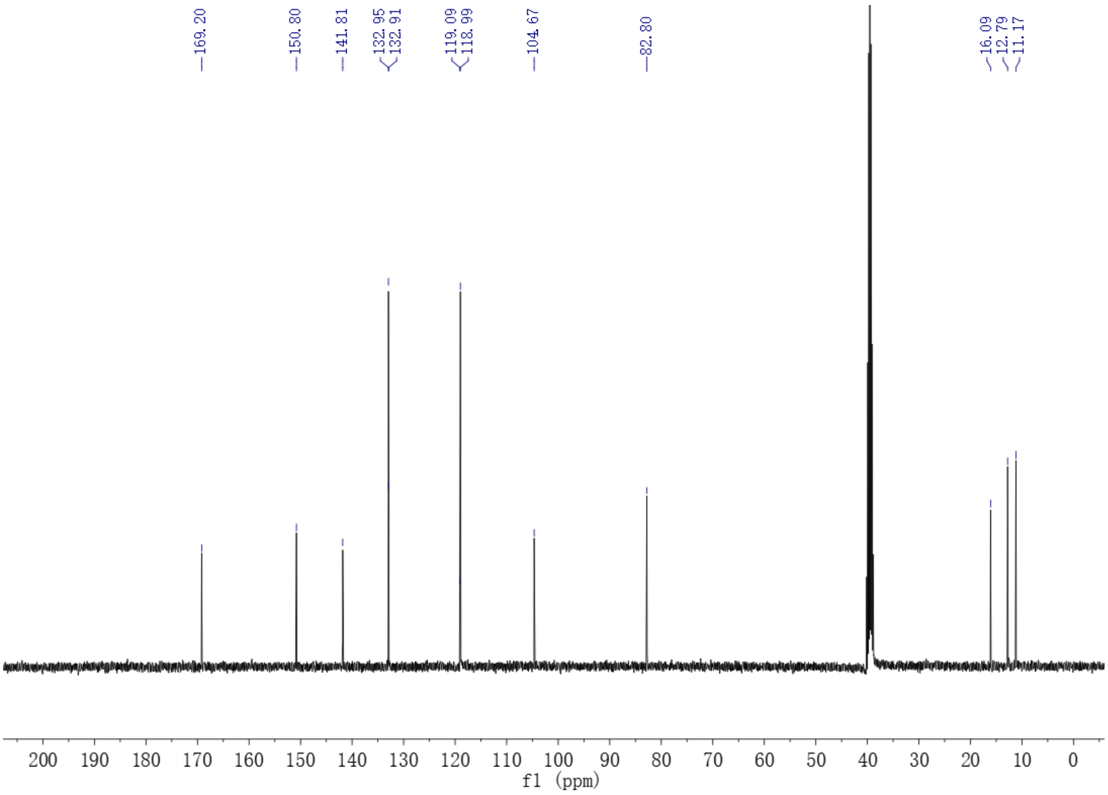

Supplementary Fig. 99. ^{1}**H NMR spectrum of compound 2q.**

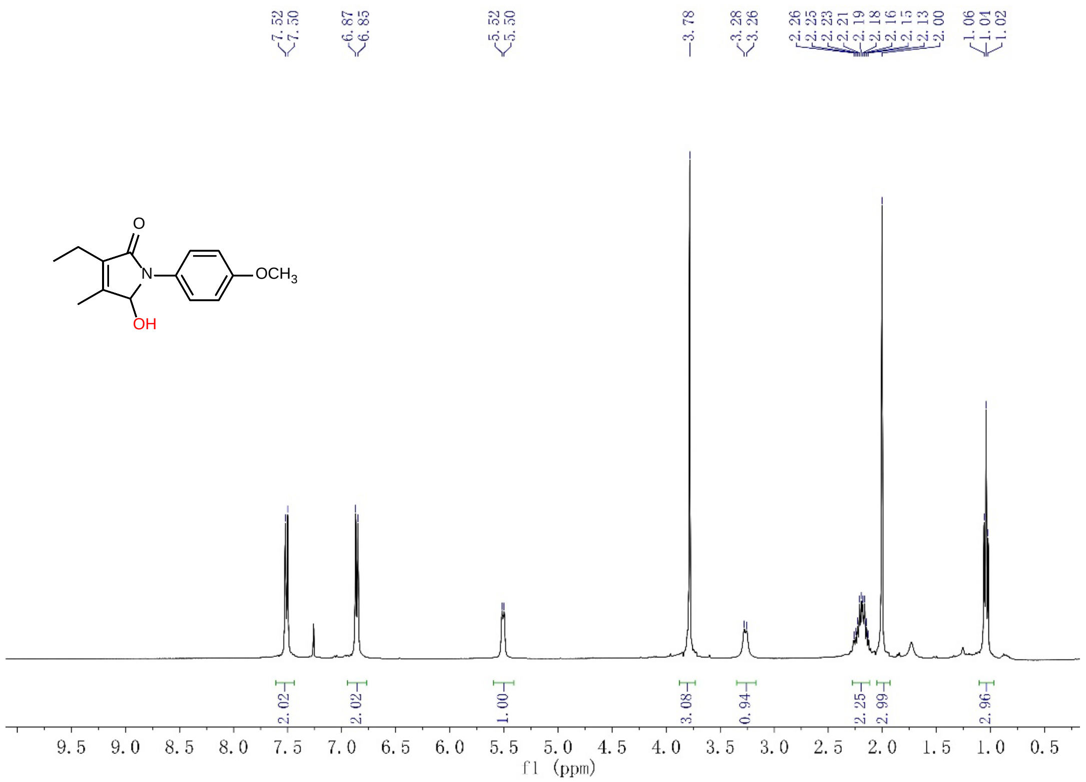

Supplementary Fig. 100. ^{1}**H NMR spectrum of compound 2r.**

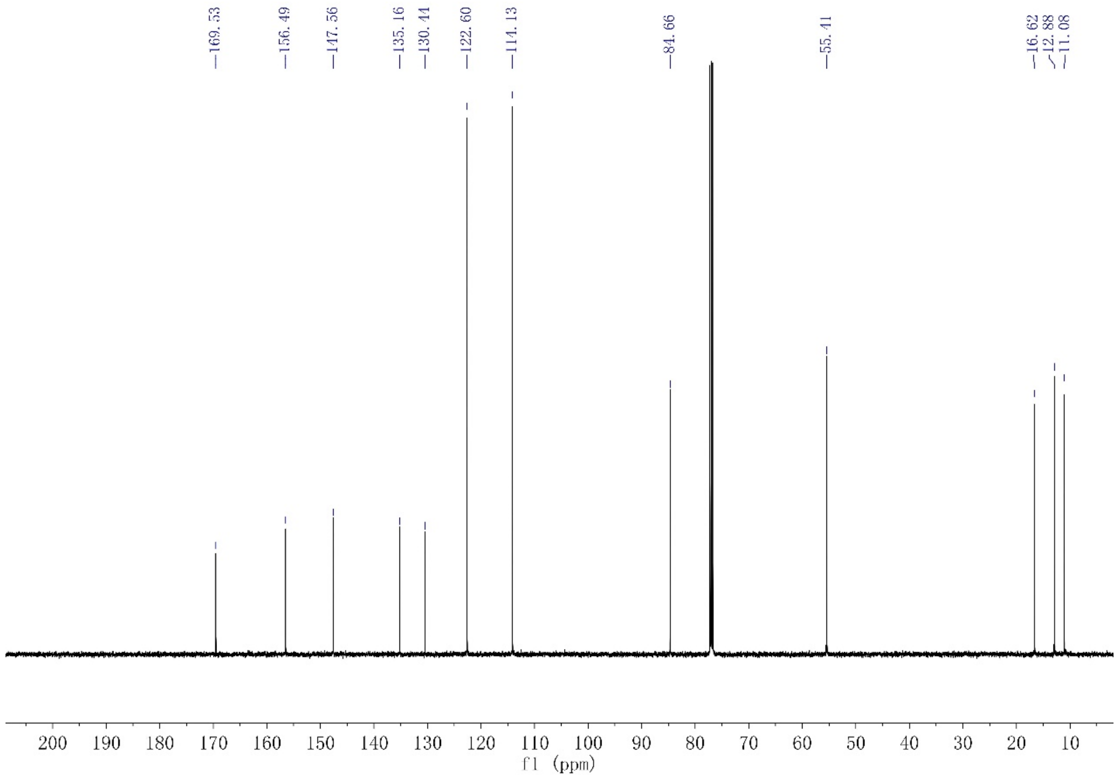

Supplementary Fig. 101. ^{13}**C NMR spectrum of compound 2r.**

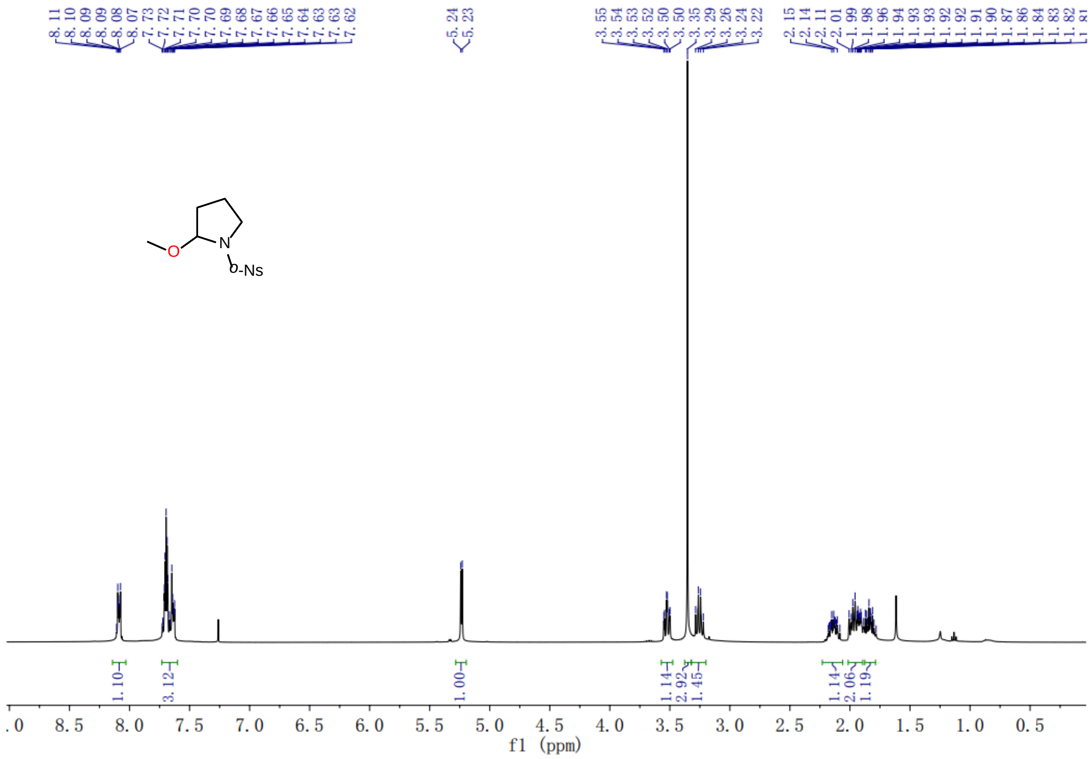

Supplementary Fig. 102. ^{1}**H NMR spectrum of compound 2sa.**

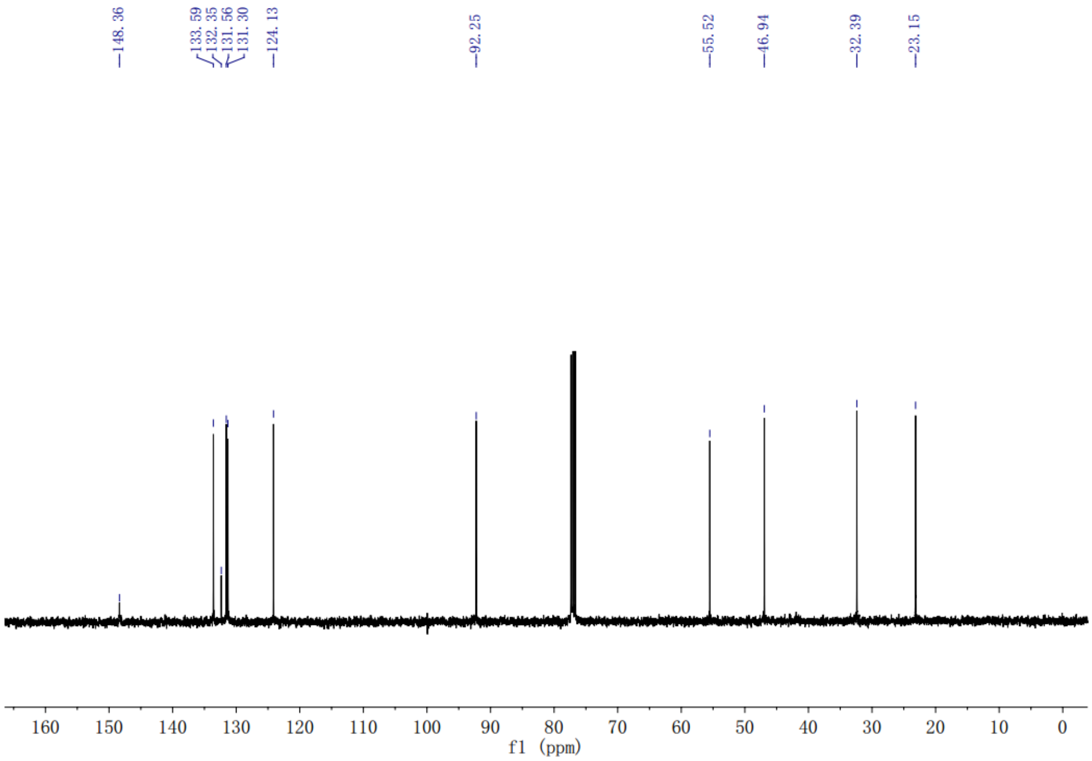

Supplementary Fig. 103. ^{13}**C NMR spectrum of compound 2sa.**

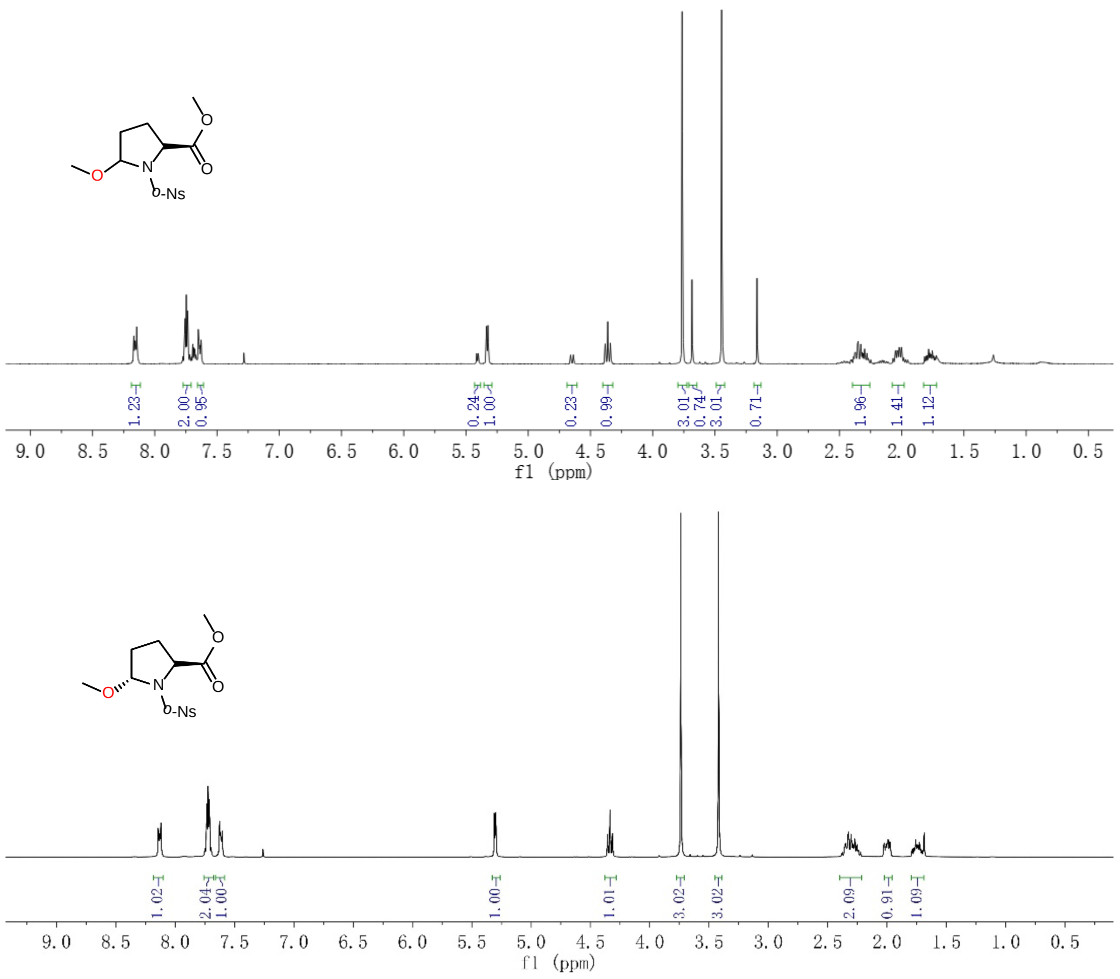

Supplementary Fig. 104. ^{1}**H NMR spectrum of compound 2ta.**

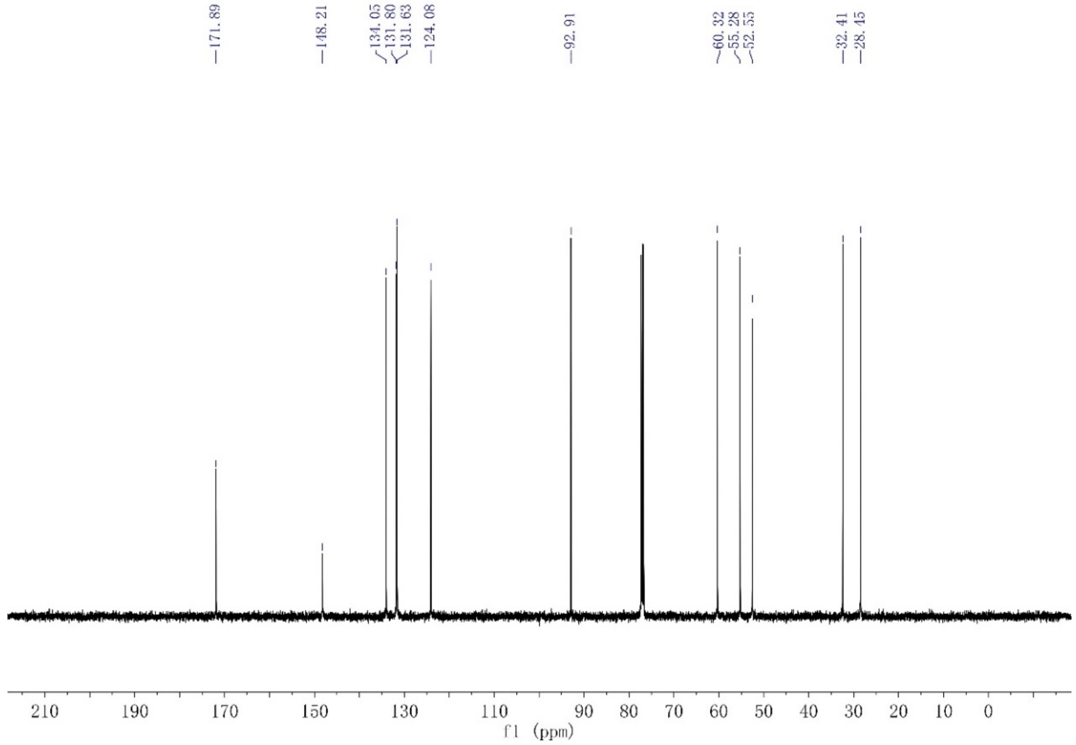

Supplementary Fig. 105. ^{13}**C NMR spectrum of compound 2ta.**

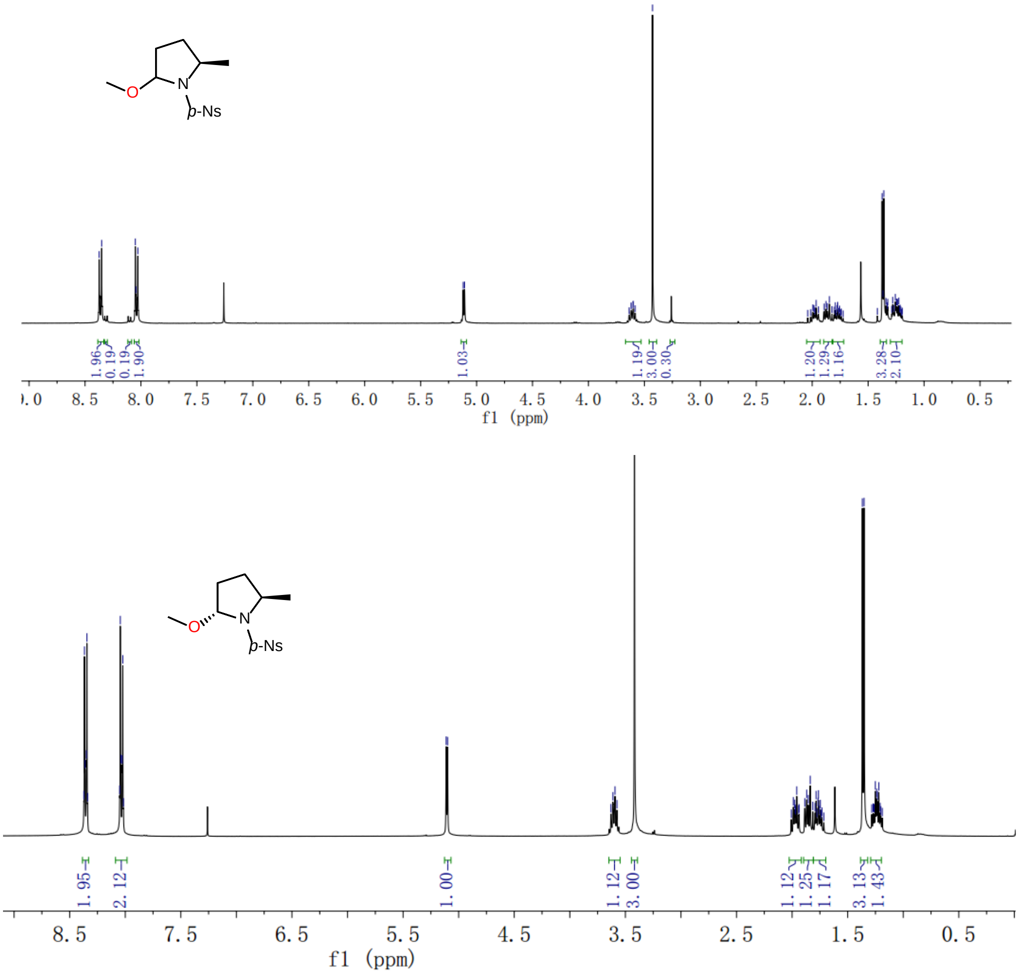

Supplementary Fig. 106. ^{1}H NMR spectrum of compound 2ua.

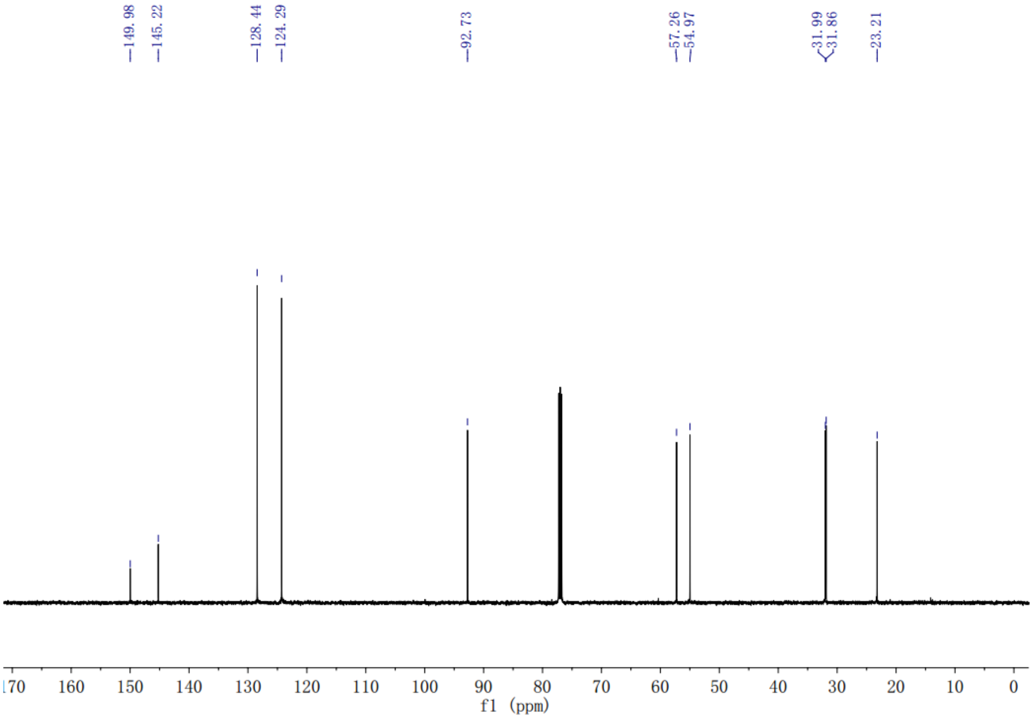

Supplementary Fig. 107. ^{13}C NMR spectrum of compound 2ua.

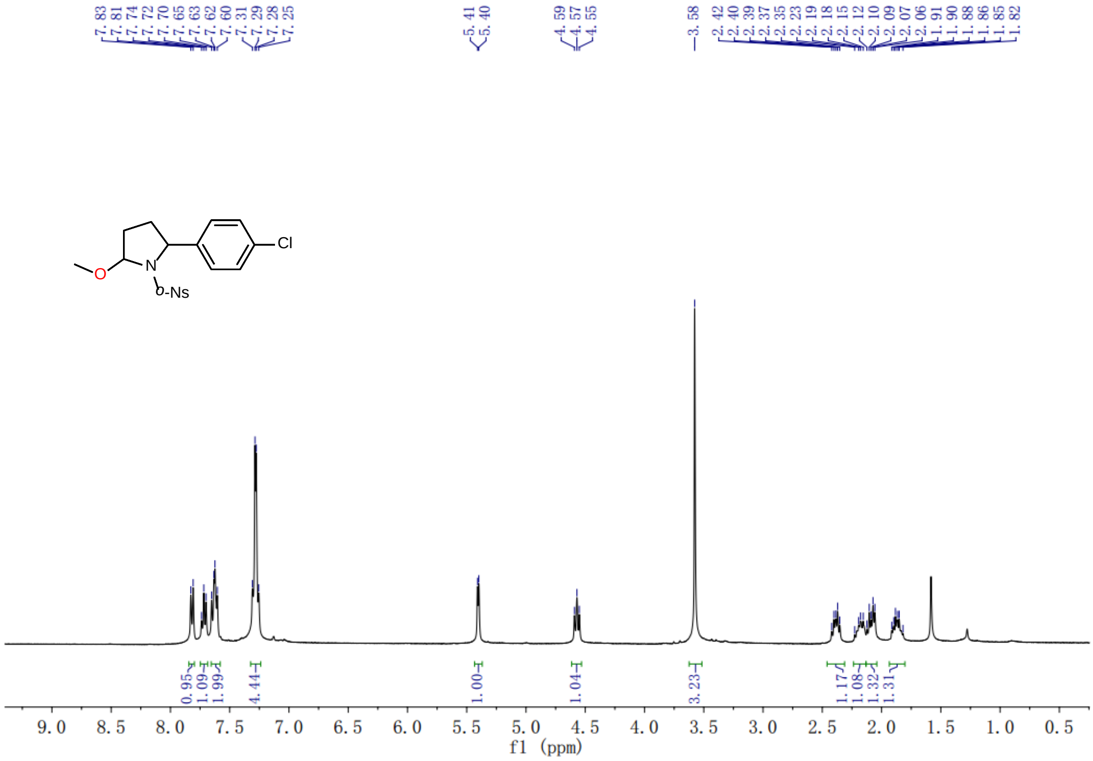

Supplementary Fig. 108. ^{1}H NMR spectrum of compound 2va.

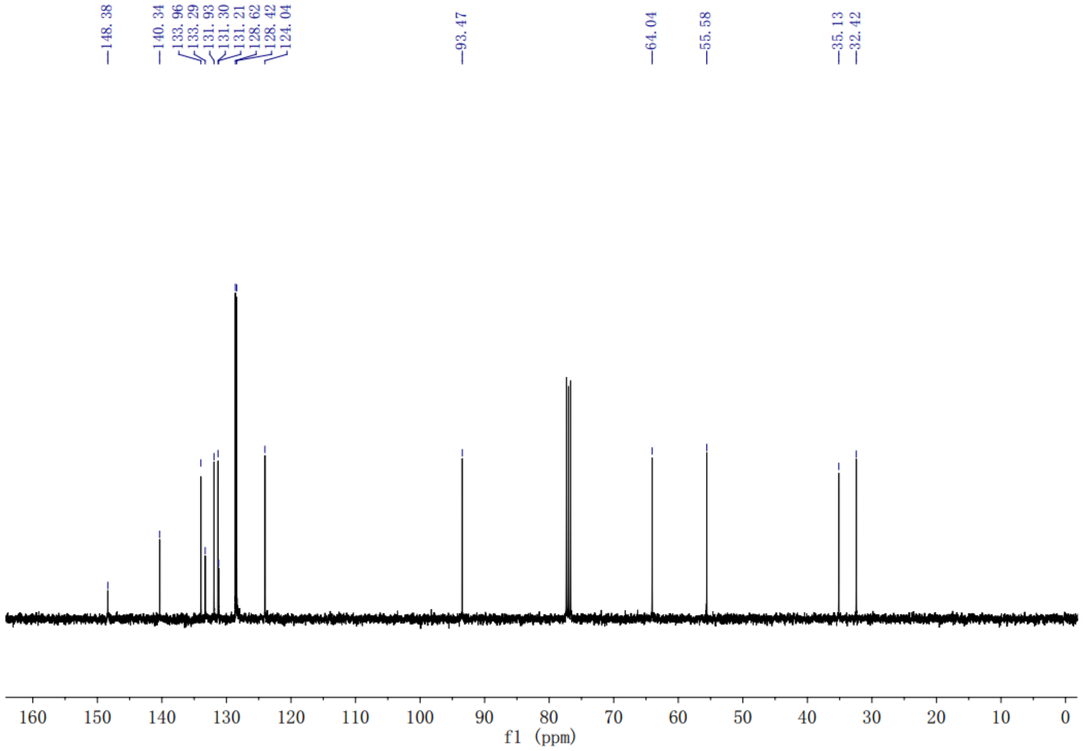

Supplementary Fig. 109. ^{13}C NMR spectrum of compound 2va.

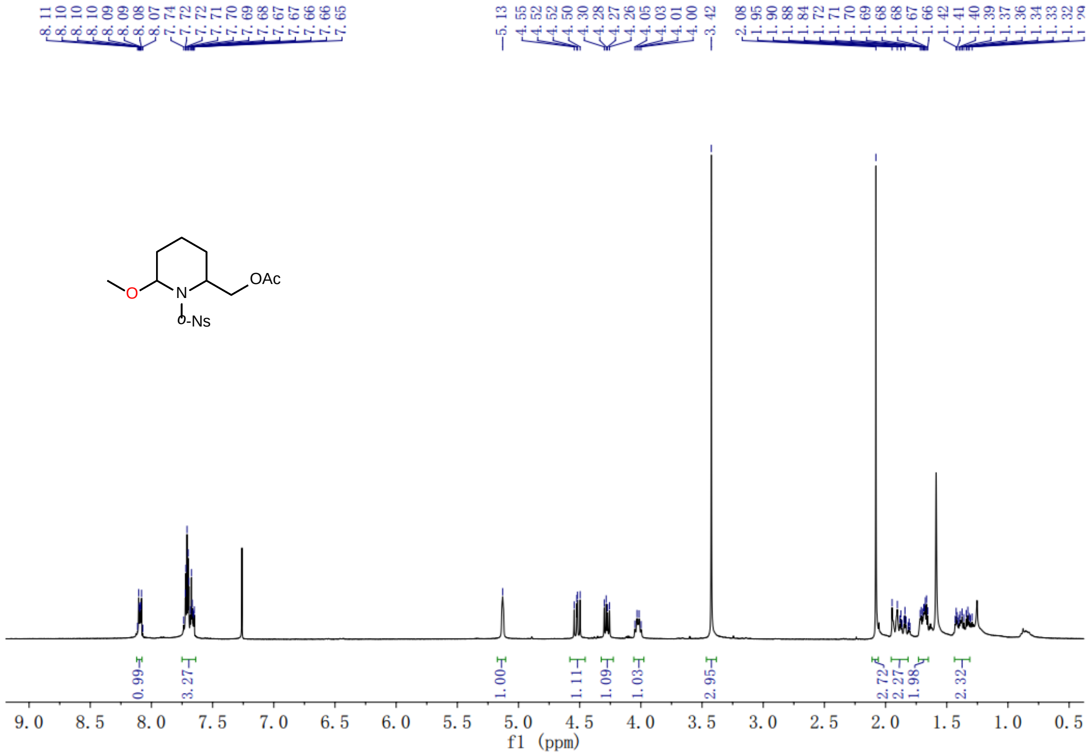

Supplementary Fig. 110. ^{1}H NMR spectrum of compound 2wa.

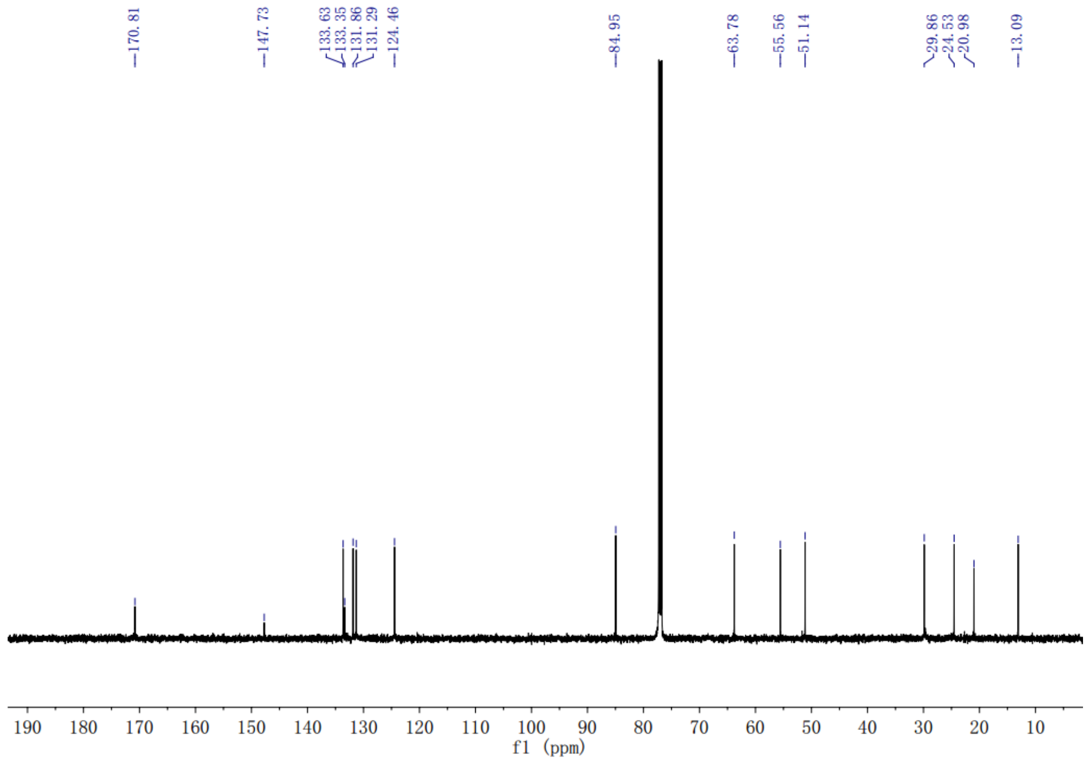

Supplementary Fig. 111. ^{13}C NMR spectrum of compound 2wa.

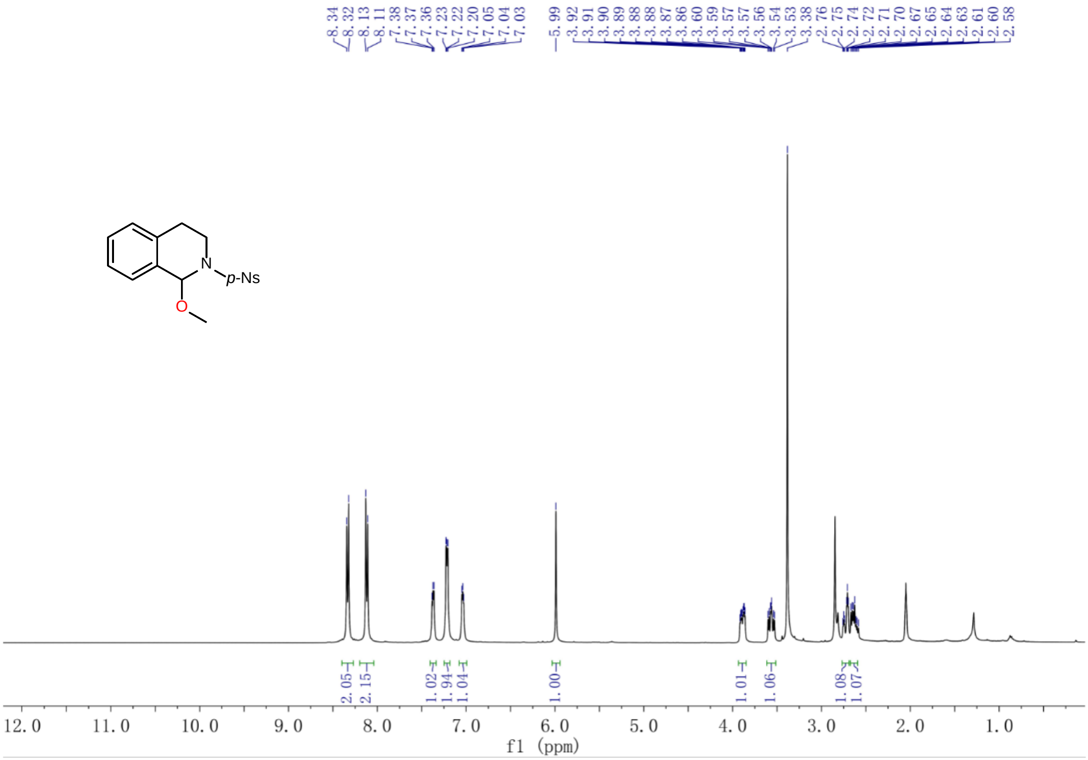

Supplementary Fig. 112. ^{1}H NMR spectrum of compound 2xa.

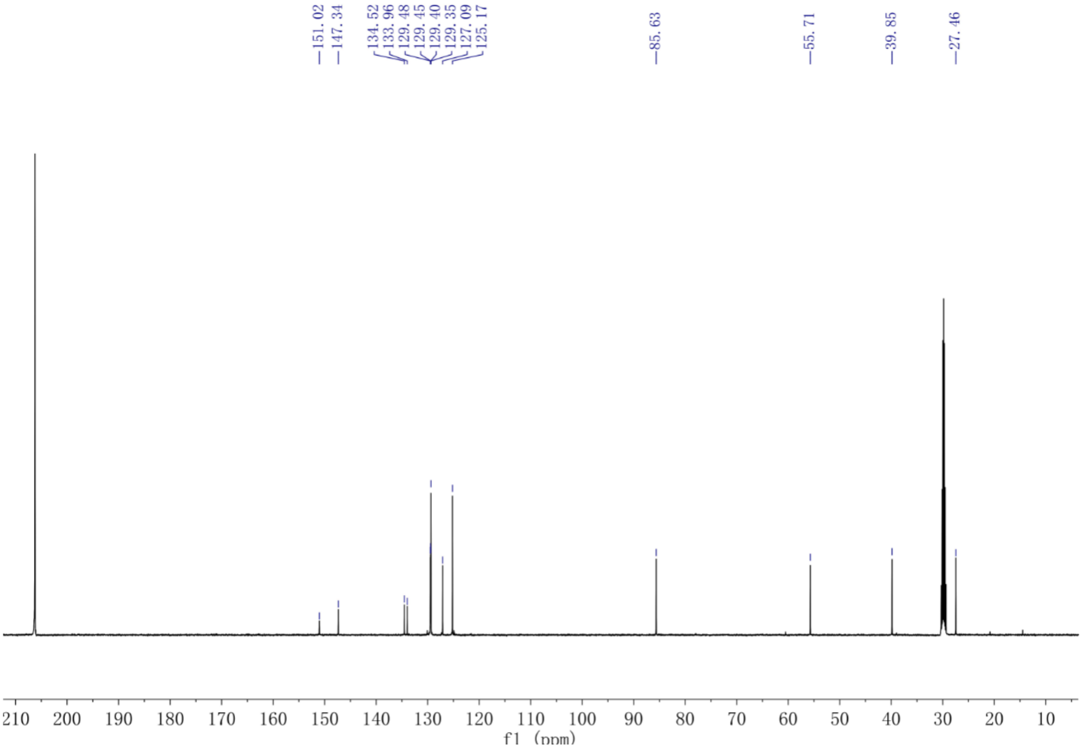

Supplementary Fig. 113. ^{13}C NMR spectrum of compound 2xa.

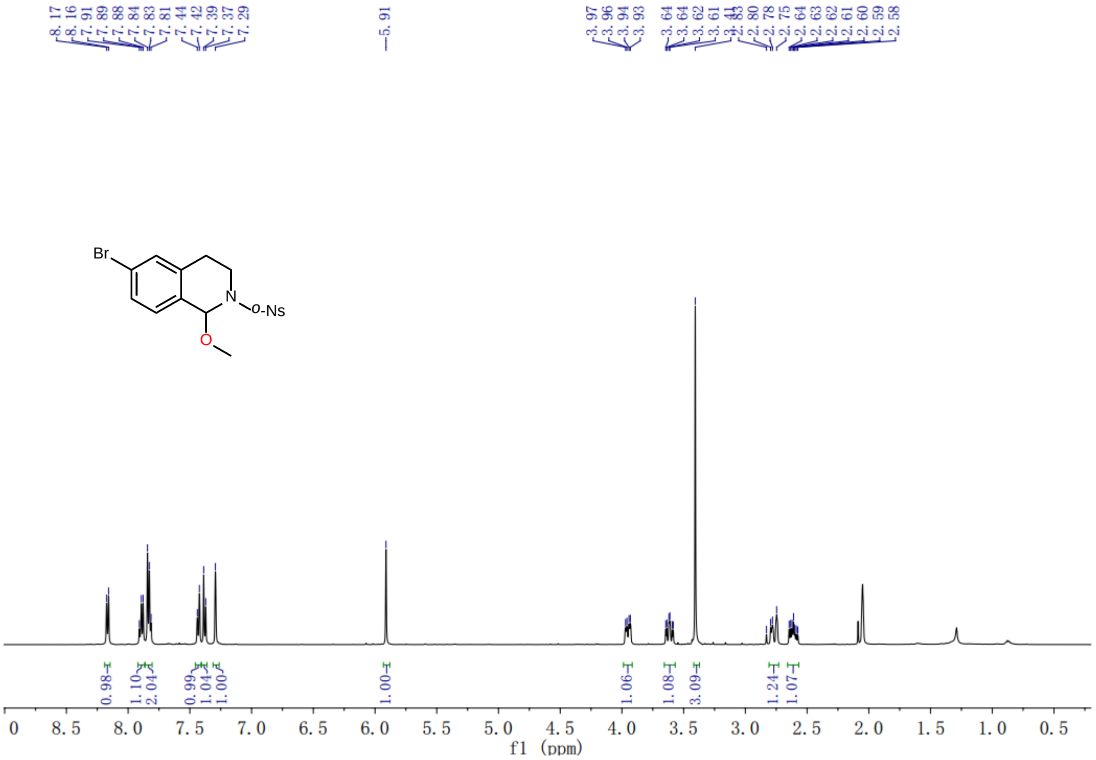

Supplementary Fig. 114. ^{1}**H NMR spectrum of compound 2ya.**

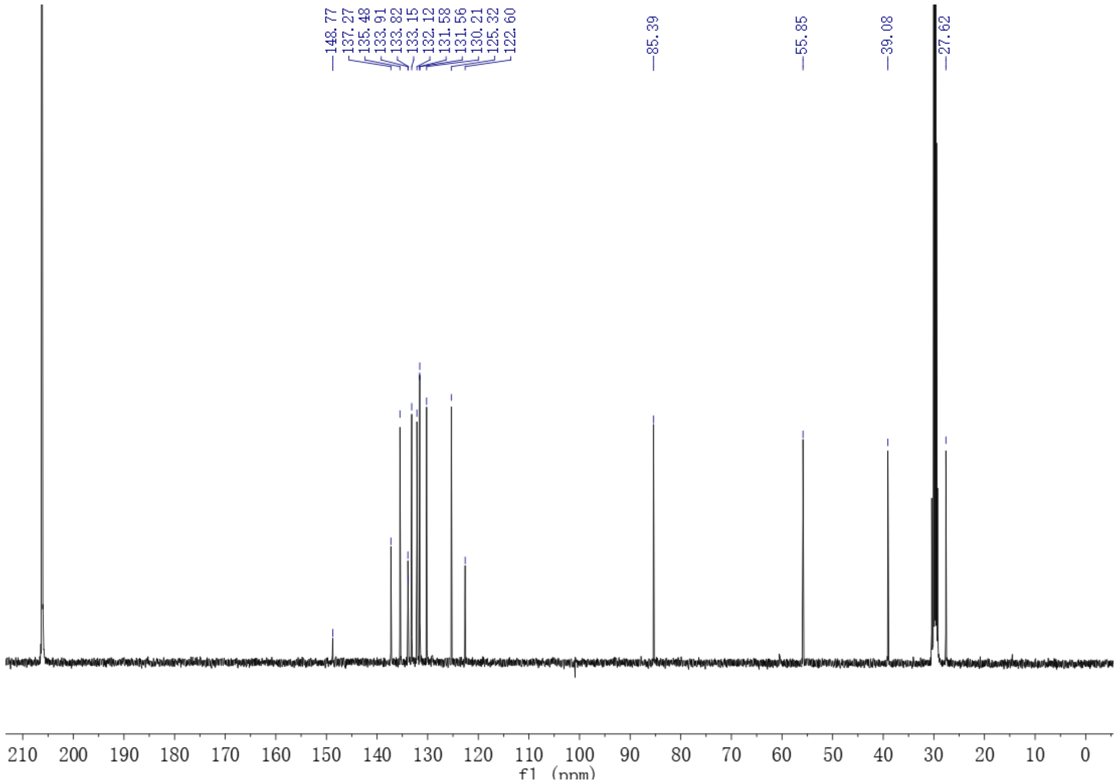

Supplementary Fig. 115. ^{13}**C NMR spectrum of compound 2ya.**

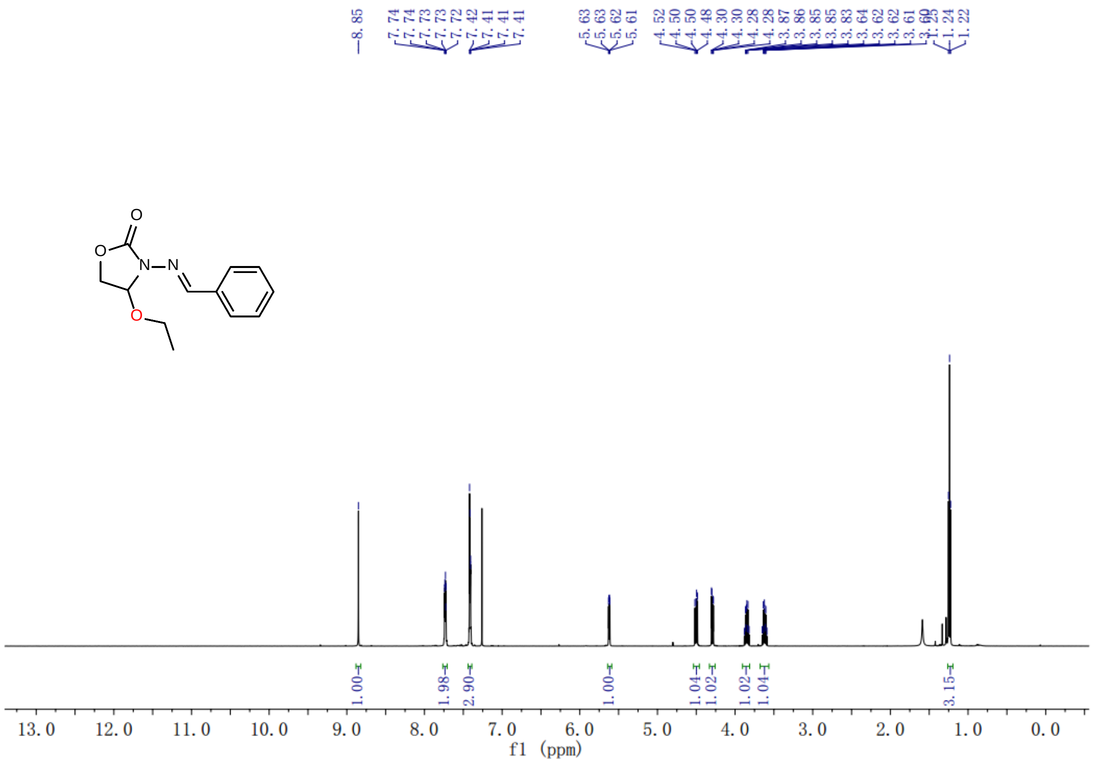

Supplementary Fig. 116. ^{1}H NMR spectrum of compound 2da.

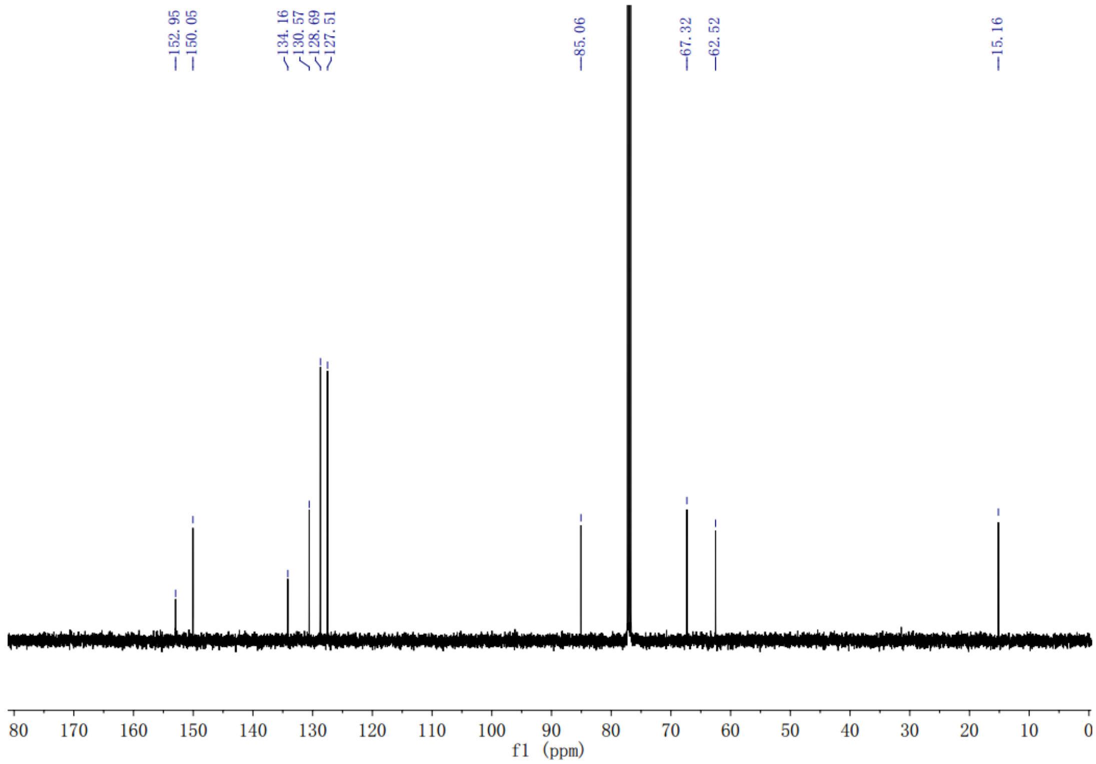

Supplementary Fig. 117. ^{13}C NMR spectrum of compound 2da.

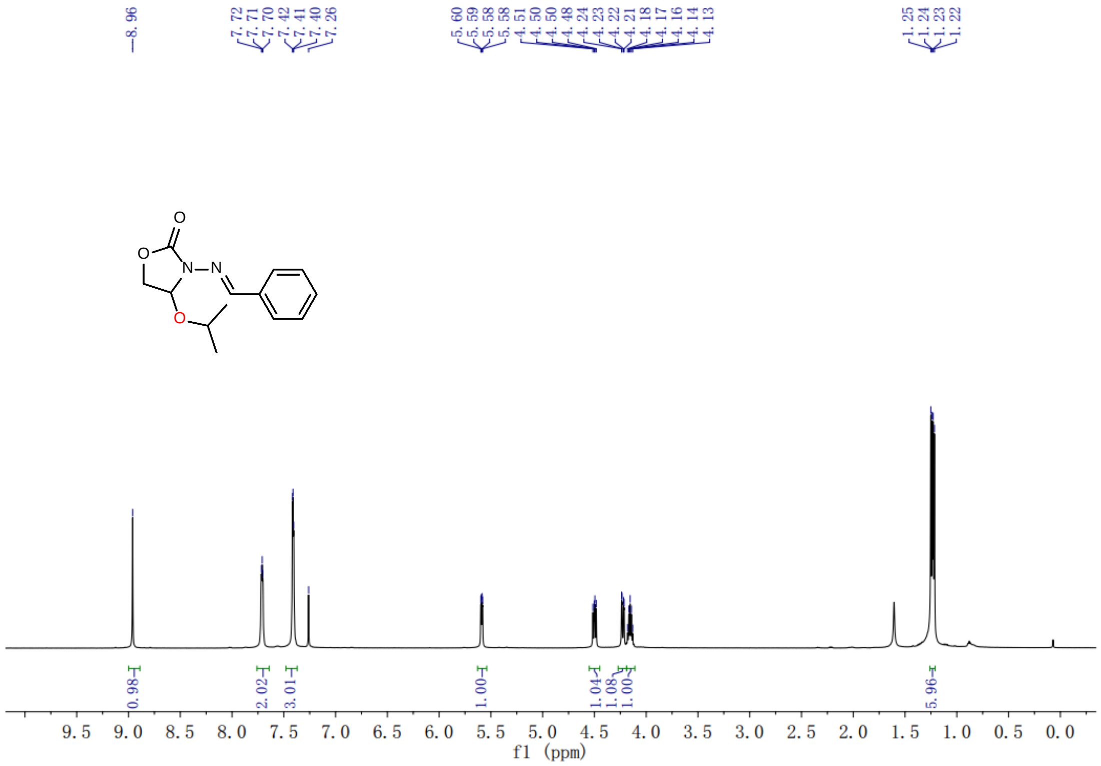

Supplementary Fig. 118. ^{1}H NMR spectrum of compound 2db.

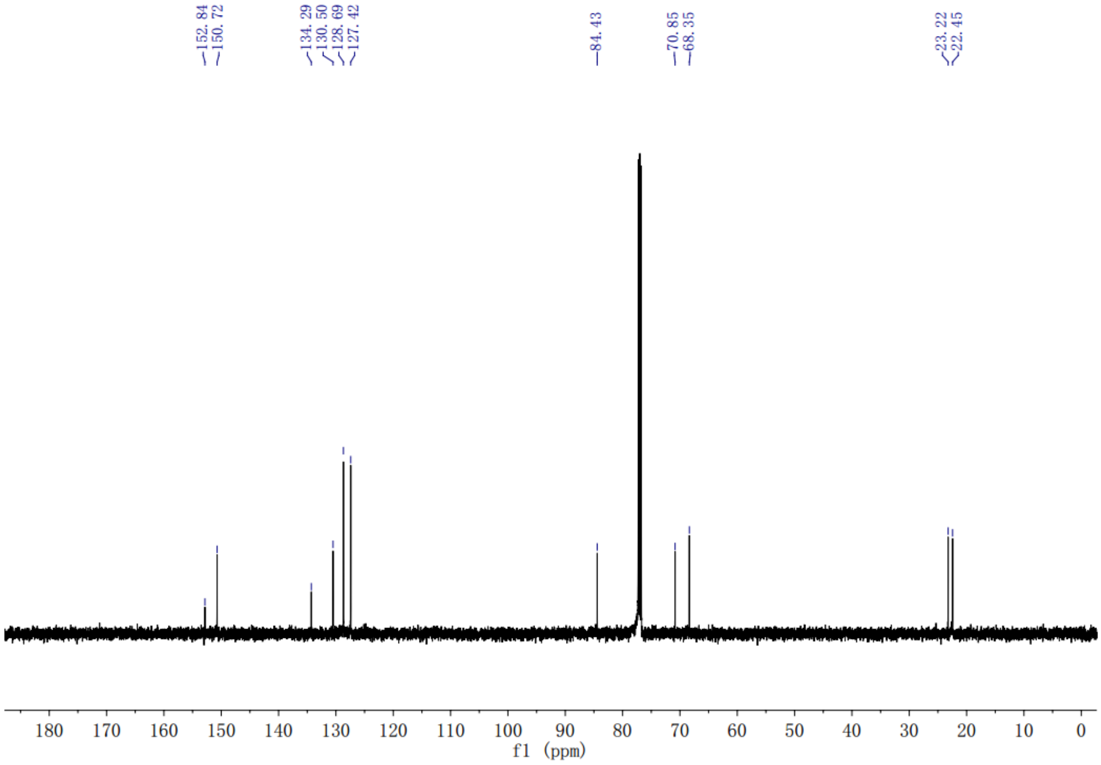

Supplementary Fig. 119. ^{13}C NMR spectrum of compound 2db.

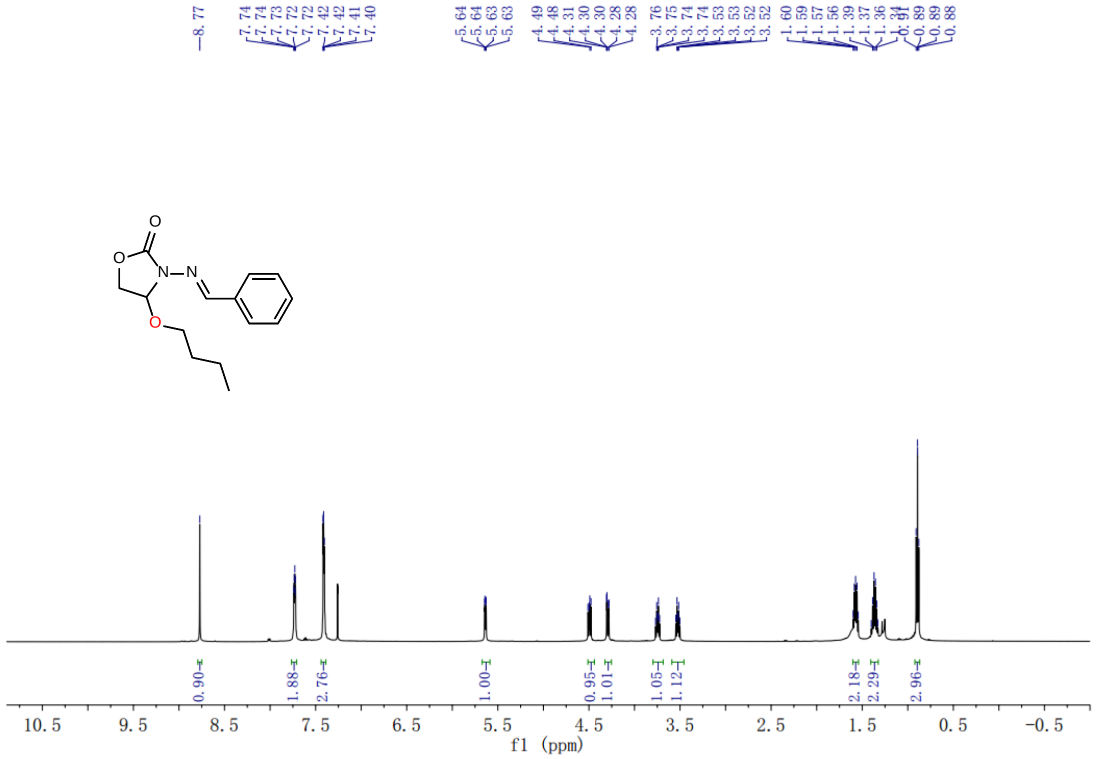

Supplementary Fig. 120. ^{1}H NMR spectrum of compound 2dc.

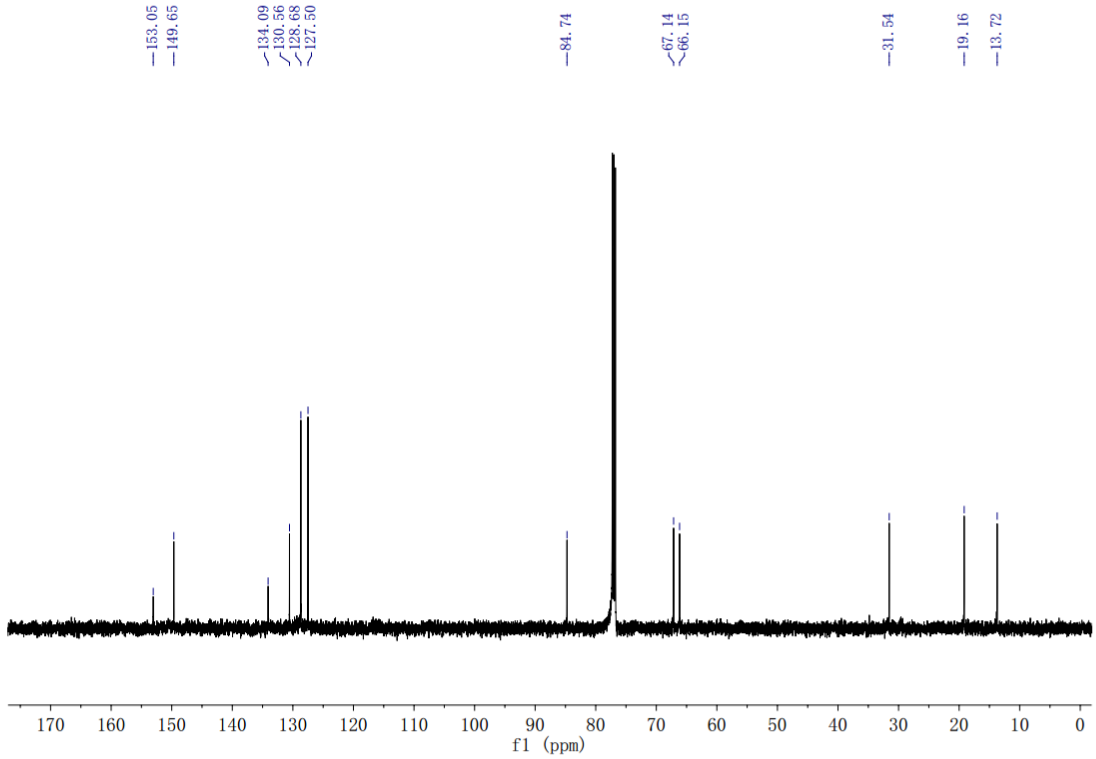

Supplementary Fig. 121. ^{13}C NMR spectrum of compound 2dc.

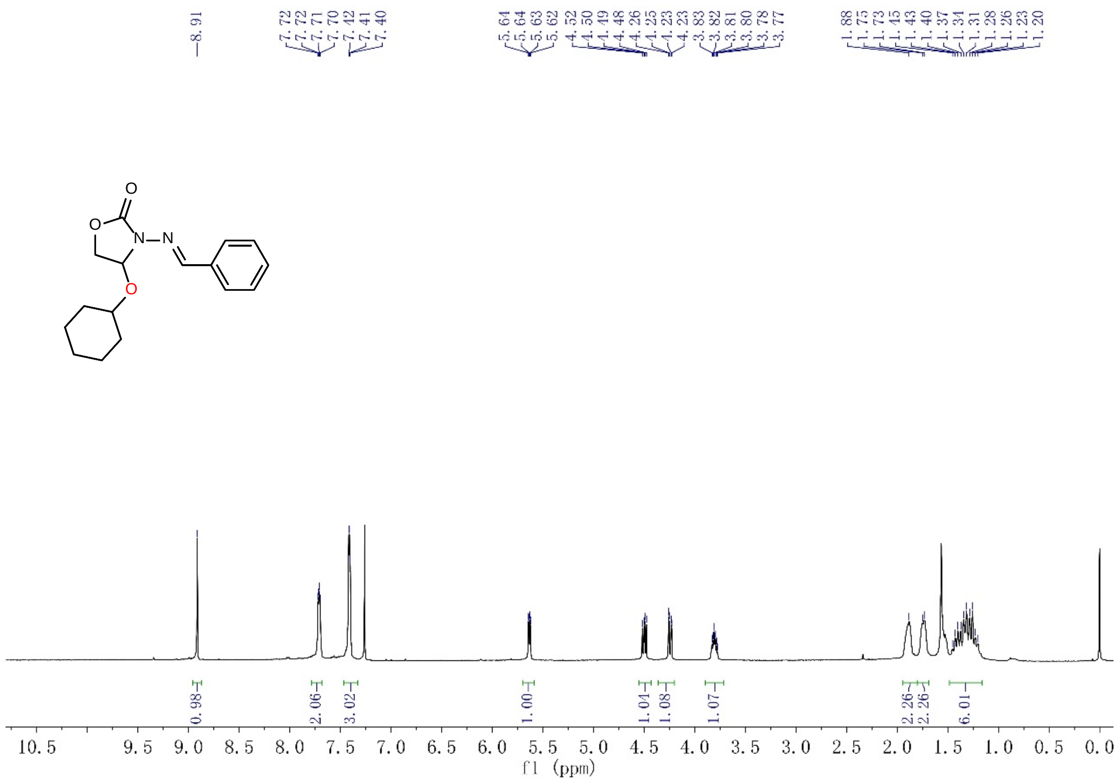

Supplementary Fig. 122. ^{1}H NMR spectrum of compound 2dd.

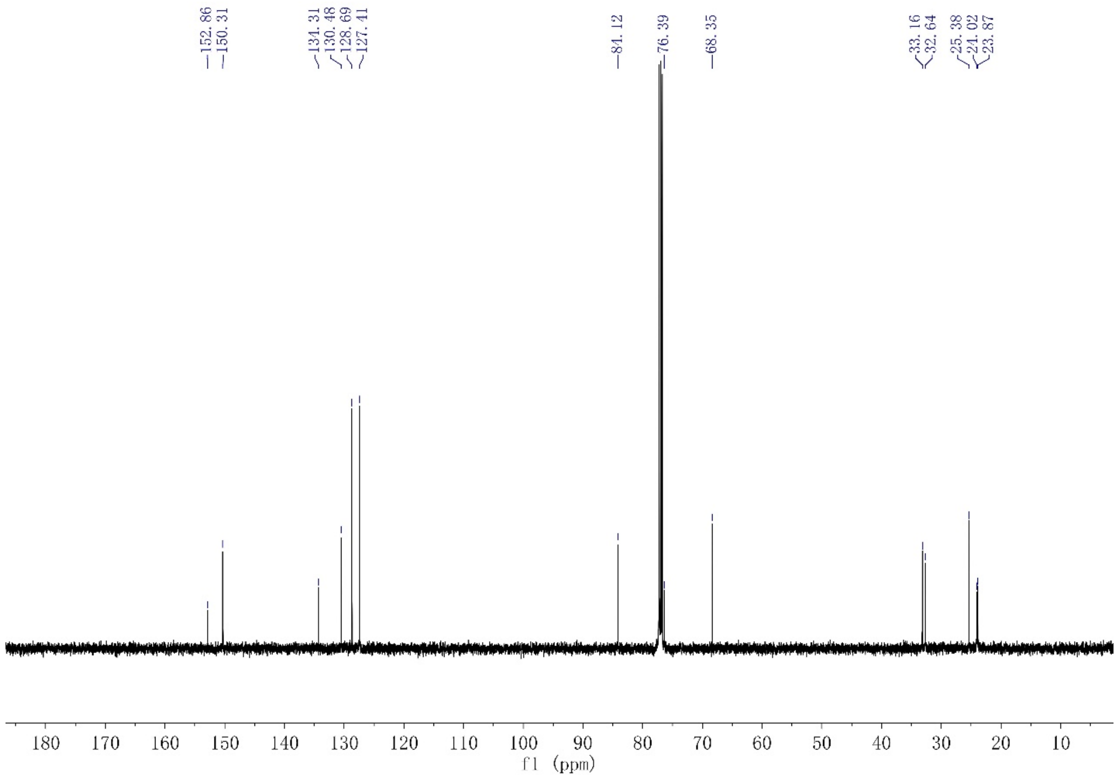

Supplementary Fig. 123. ^{13}C NMR spectrum of compound 2dd.

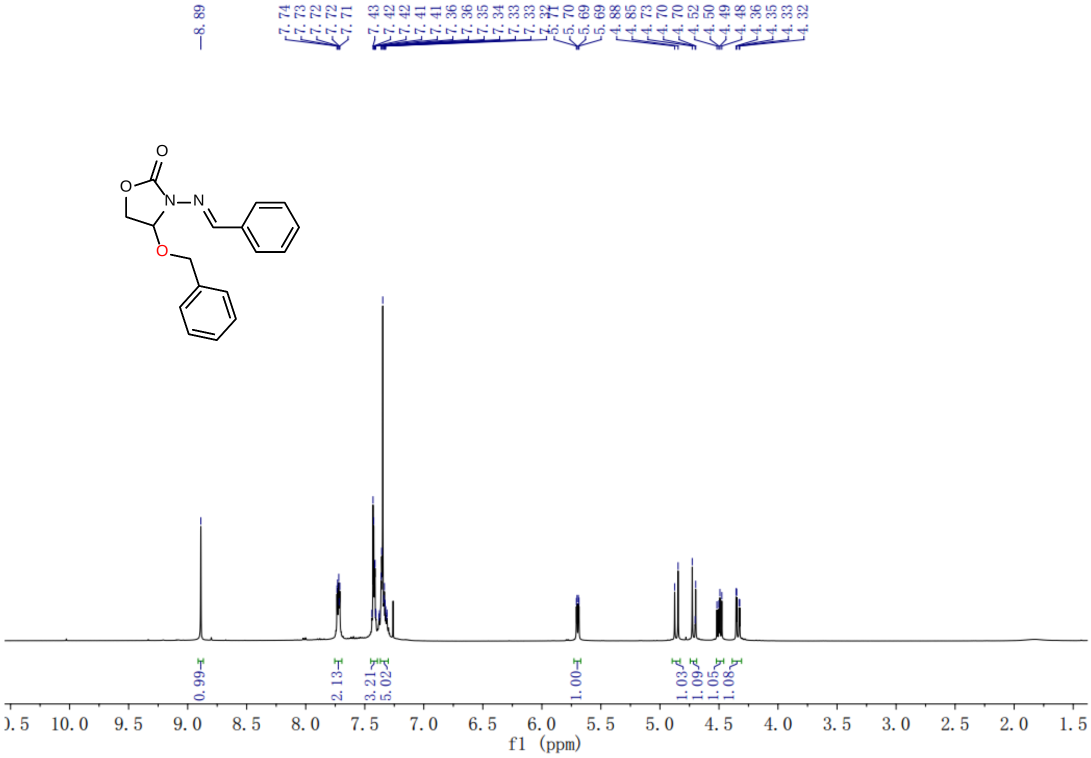

Supplementary Fig. 124. ^{1}H NMR spectrum of compound 2de.

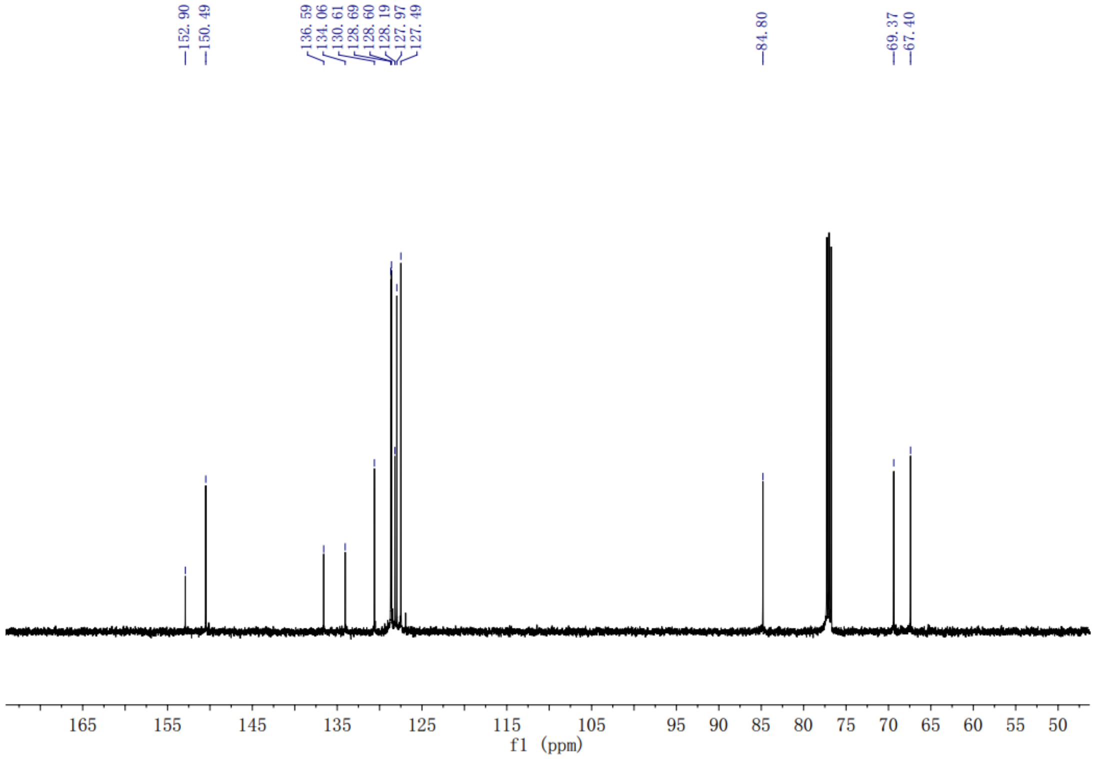

Supplementary Fig. 125. ^{13}C NMR spectrum of compound 2de.

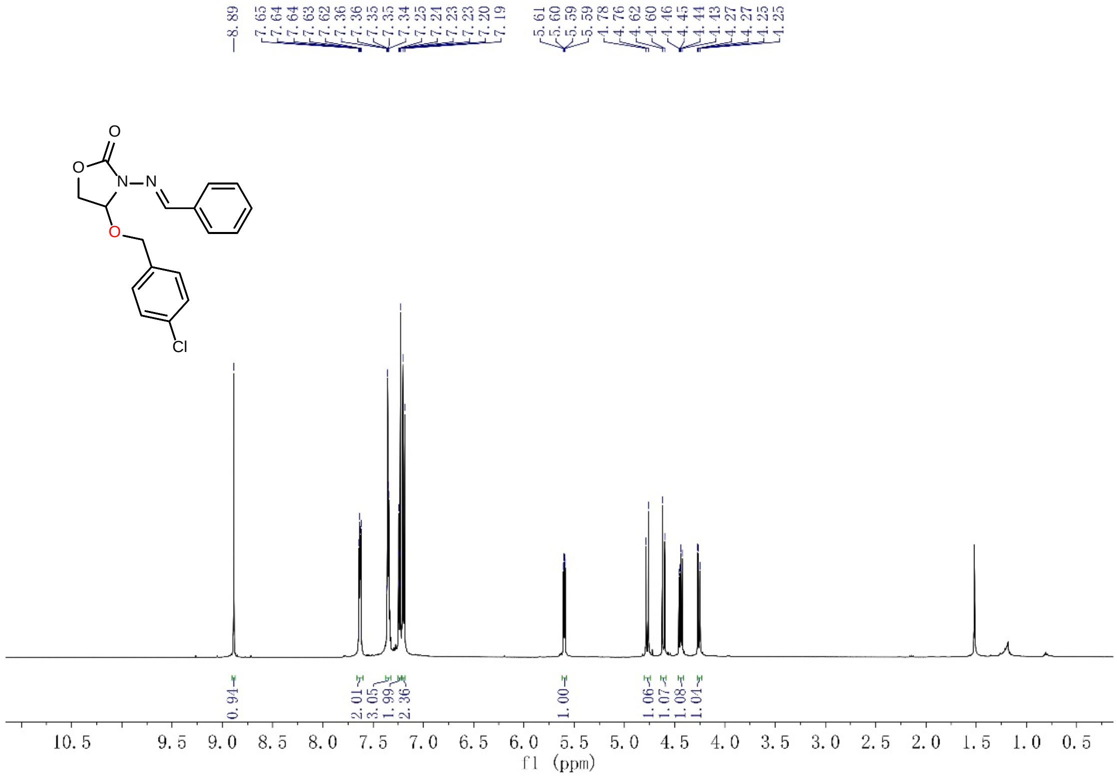

Supplementary Fig. 126. ^{1}H NMR spectrum of compound 2df.

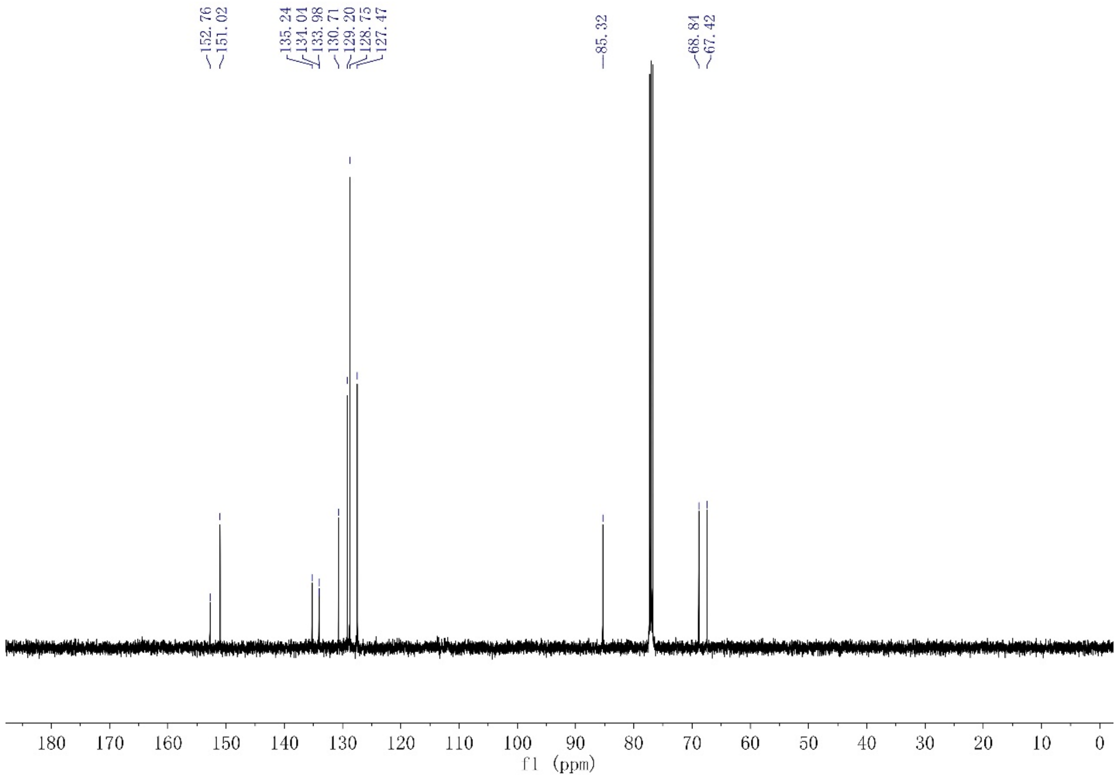

Supplementary Fig. 127. ^{13}C NMR spectrum of compound 2df.

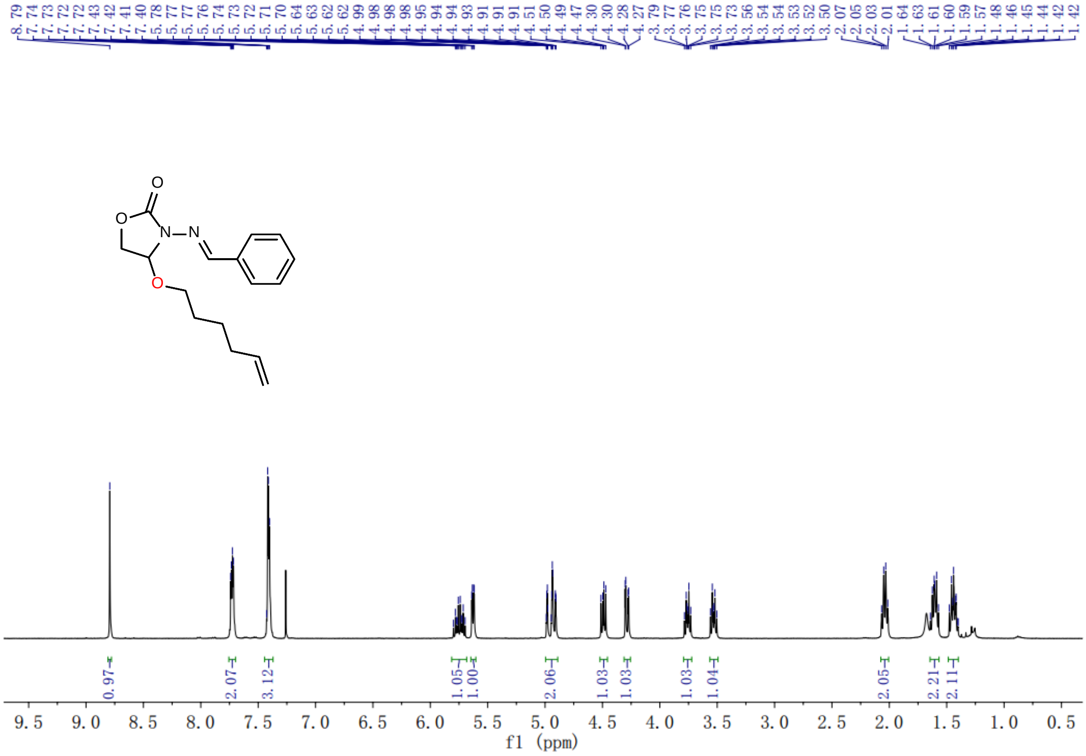

Supplementary Fig. 128. **^{1}H NMR spectrum of compound 2dg.**

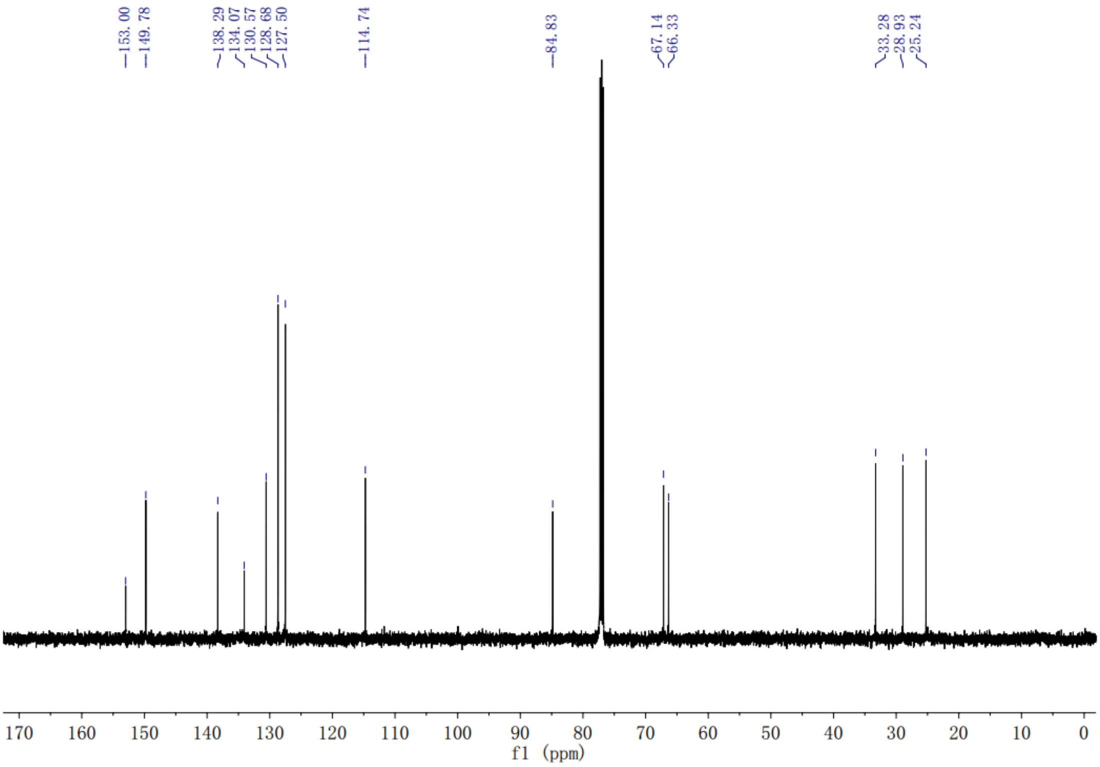

Supplementary Fig. 129. **^{13}C NMR spectrum of compound 2dg.**

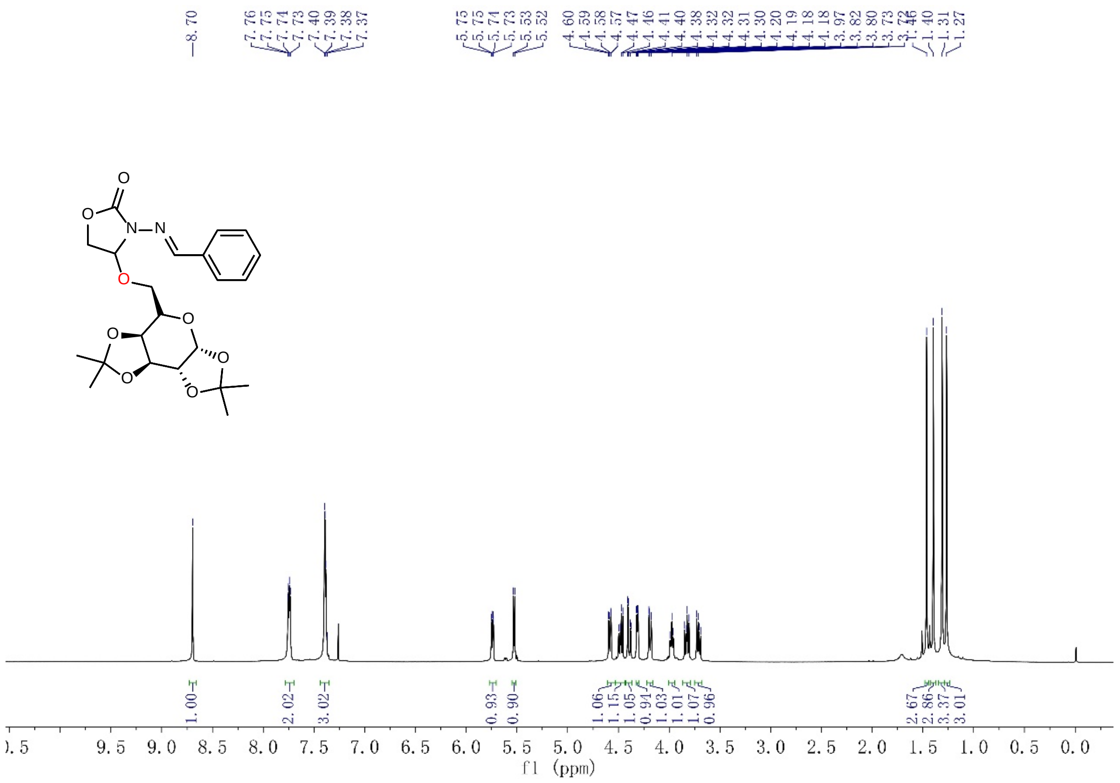

Supplementary Fig. 130. ^{1}H NMR spectrum of compound 2dh.

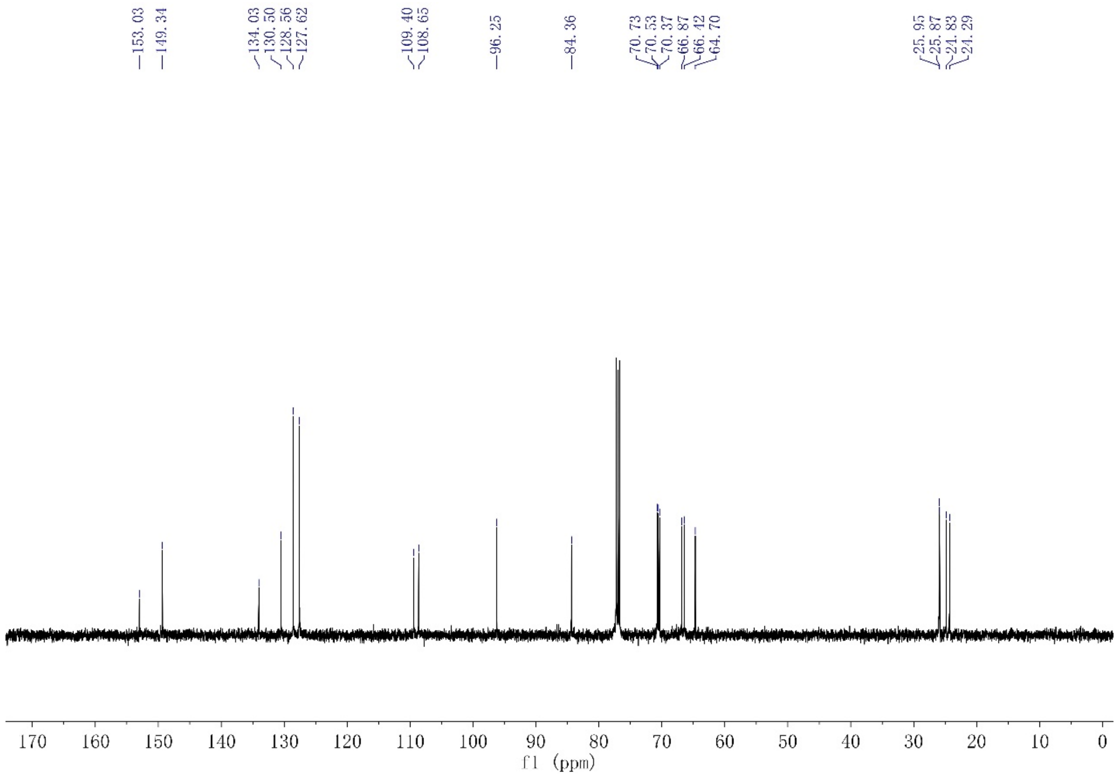

Supplementary Fig. 131. ^{13}C NMR spectrum of compound 2dh.

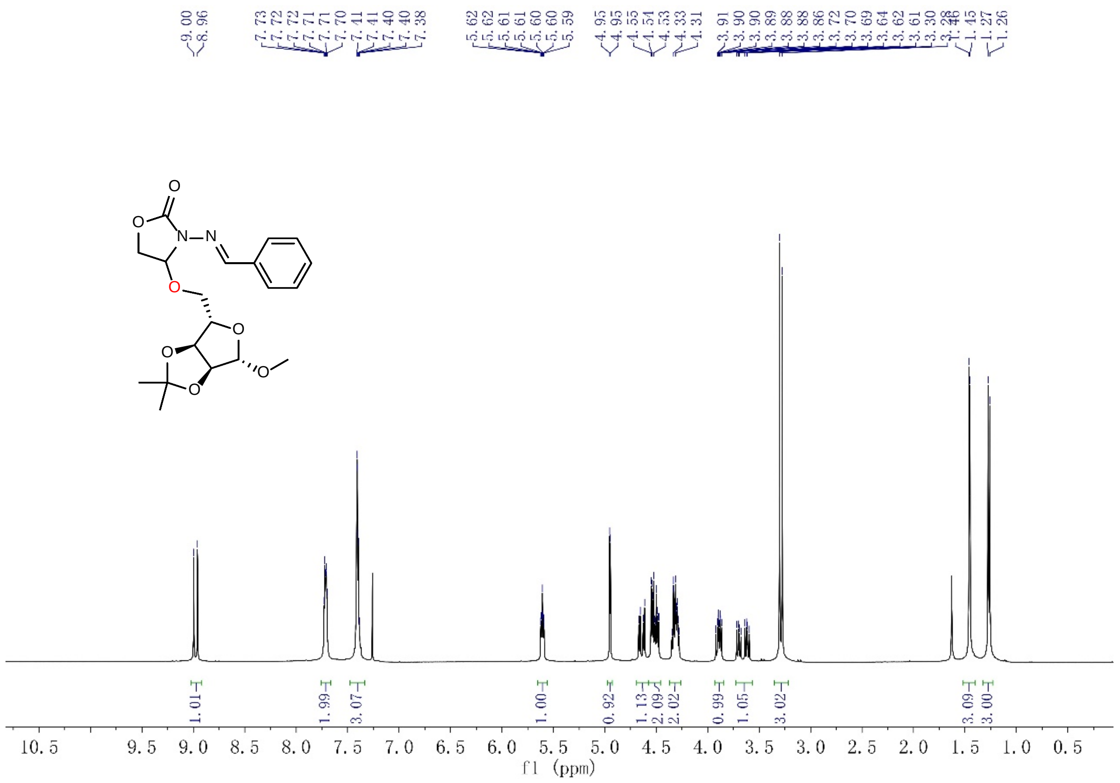

Supplementary Fig. 132. ^{1}H NMR spectrum of compound 2di.

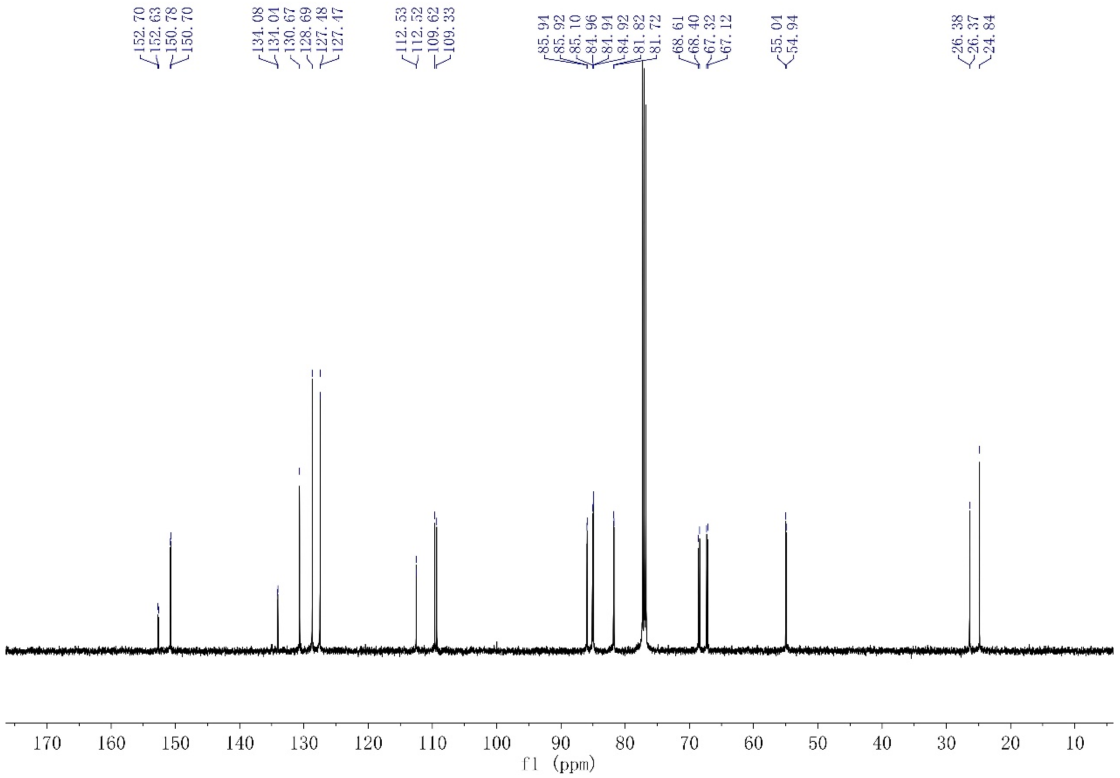

Supplementary Fig. 133. ^{13}C NMR spectrum of compound 2di.

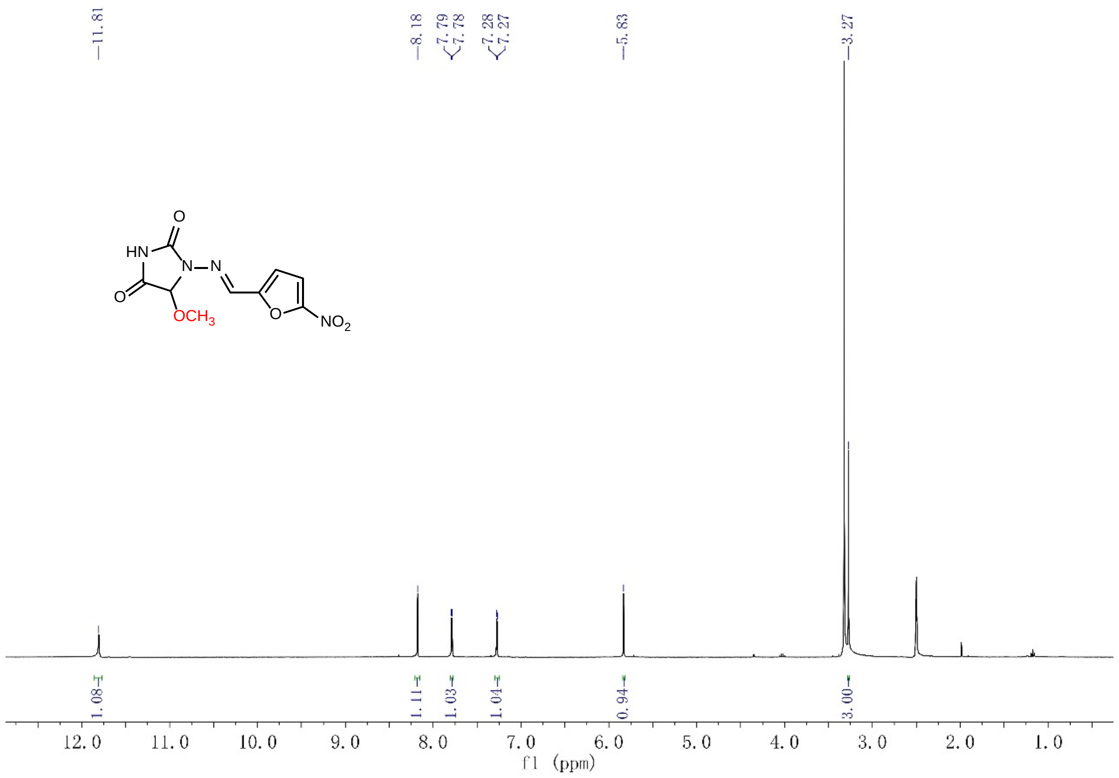

Supplementary Fig. 134. ^{1}H NMR spectrum of compound 2z1.

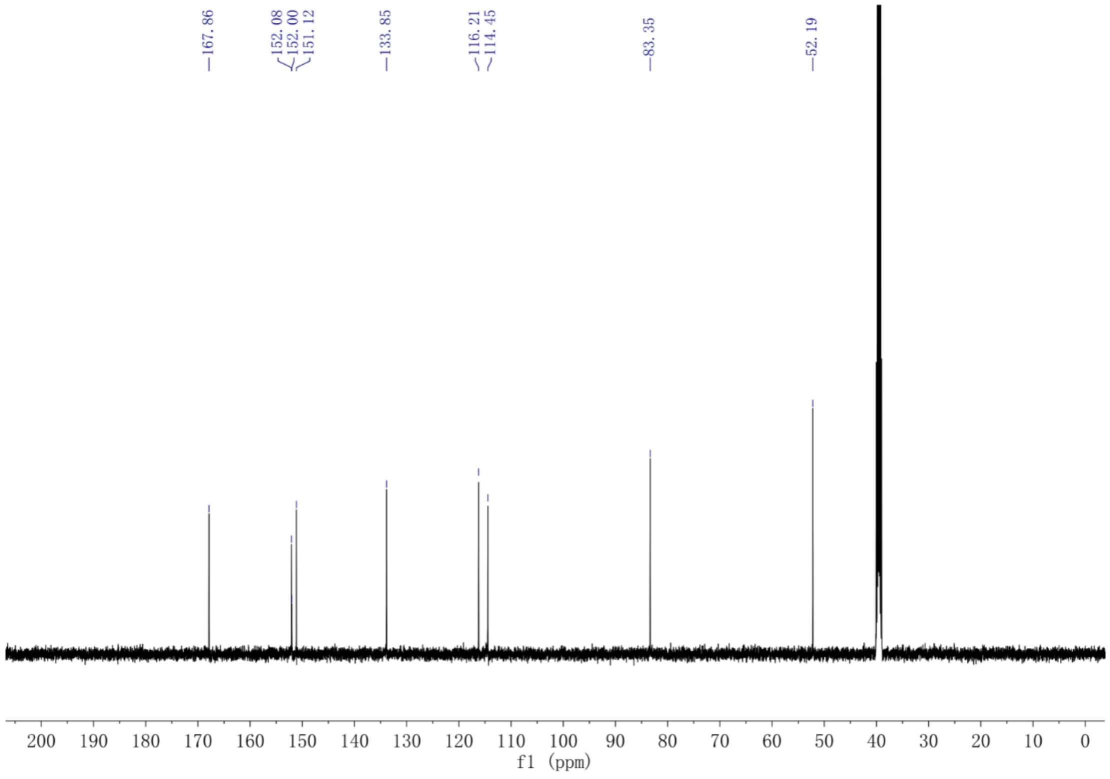

Supplementary Fig. 135. ^{13}C NMR spectrum of compound 2z1.

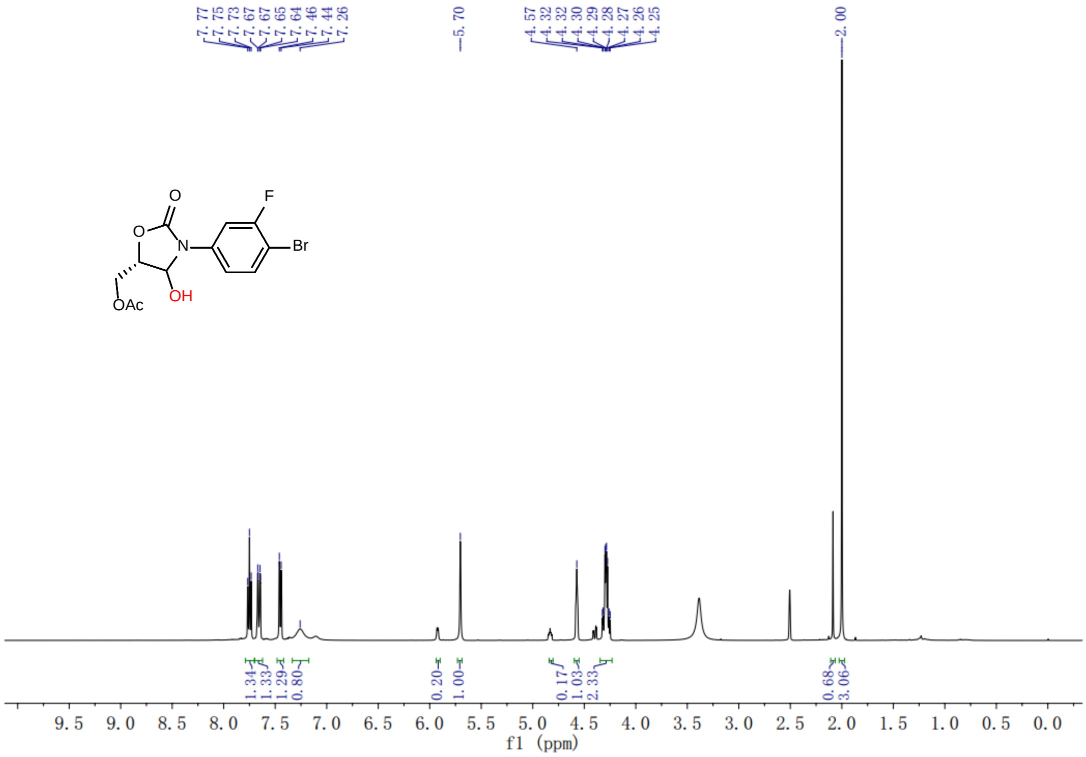

Supplementary Fig. 136. ^{1}H NMR spectrum of compound 2z2.

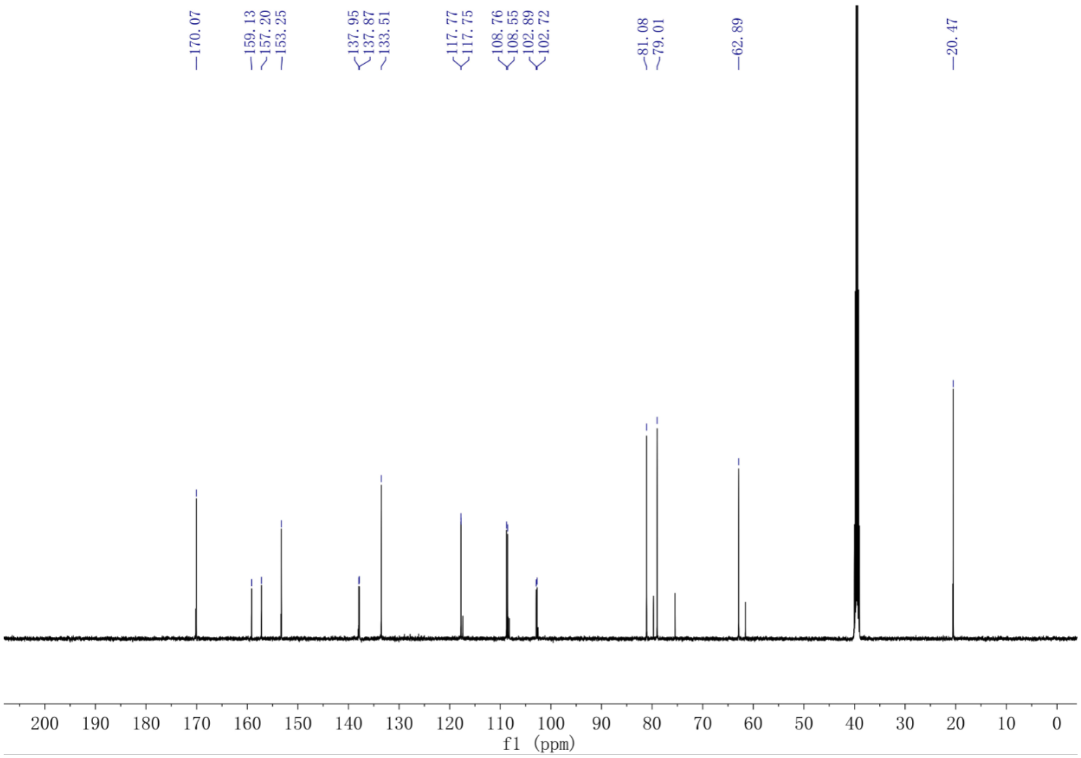

Supplementary Fig. 137. ^{13}C NMR spectrum of compound 2z2.

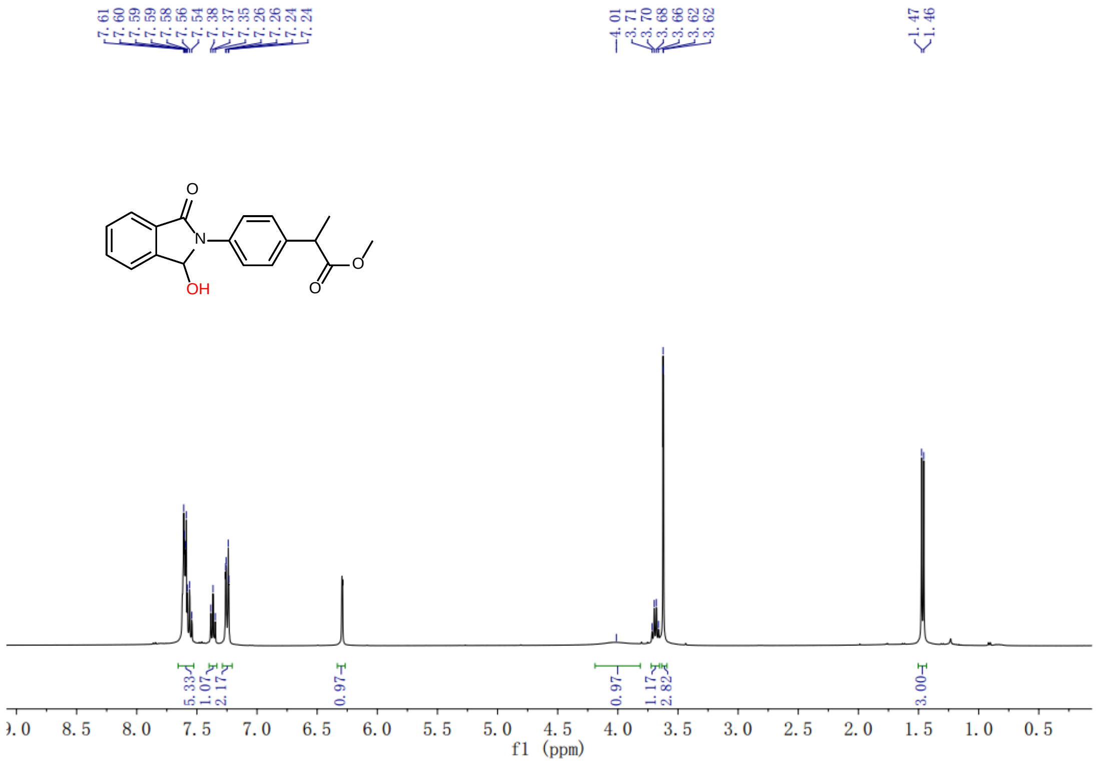

Supplementary Fig. 138. ^{1}H NMR spectrum of compound 2z3.

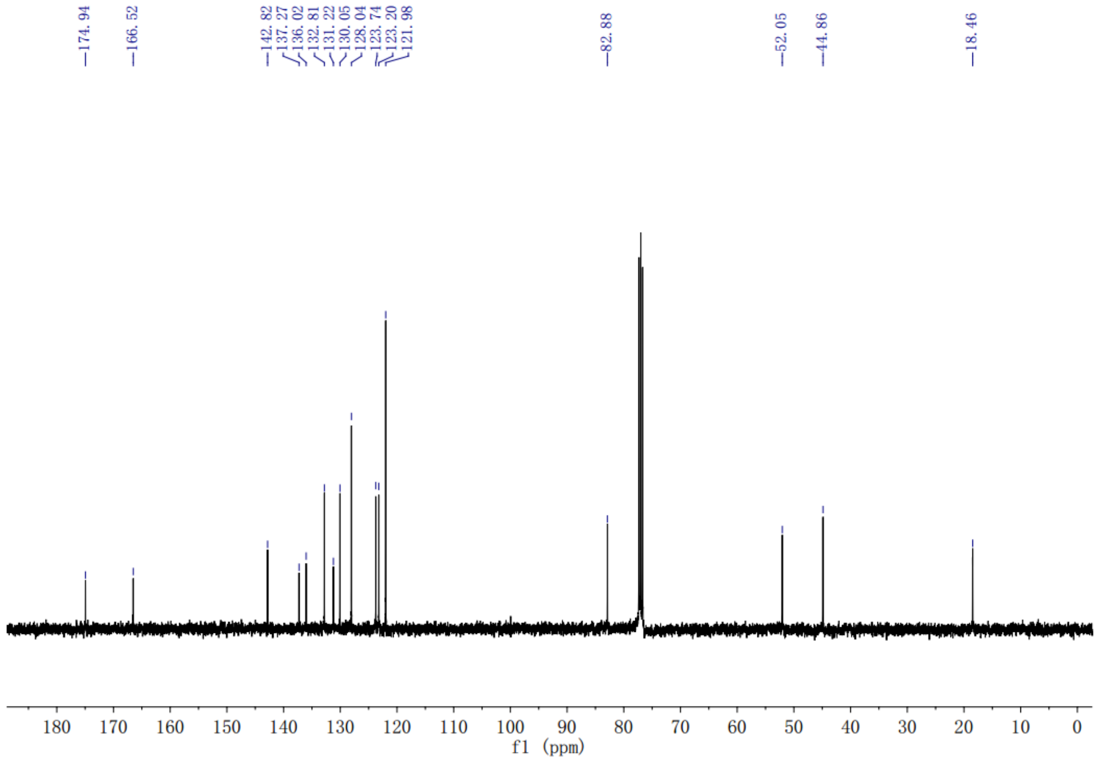

Supplementary Fig. 139. ^{13}C NMR spectrum of compound 2z3.

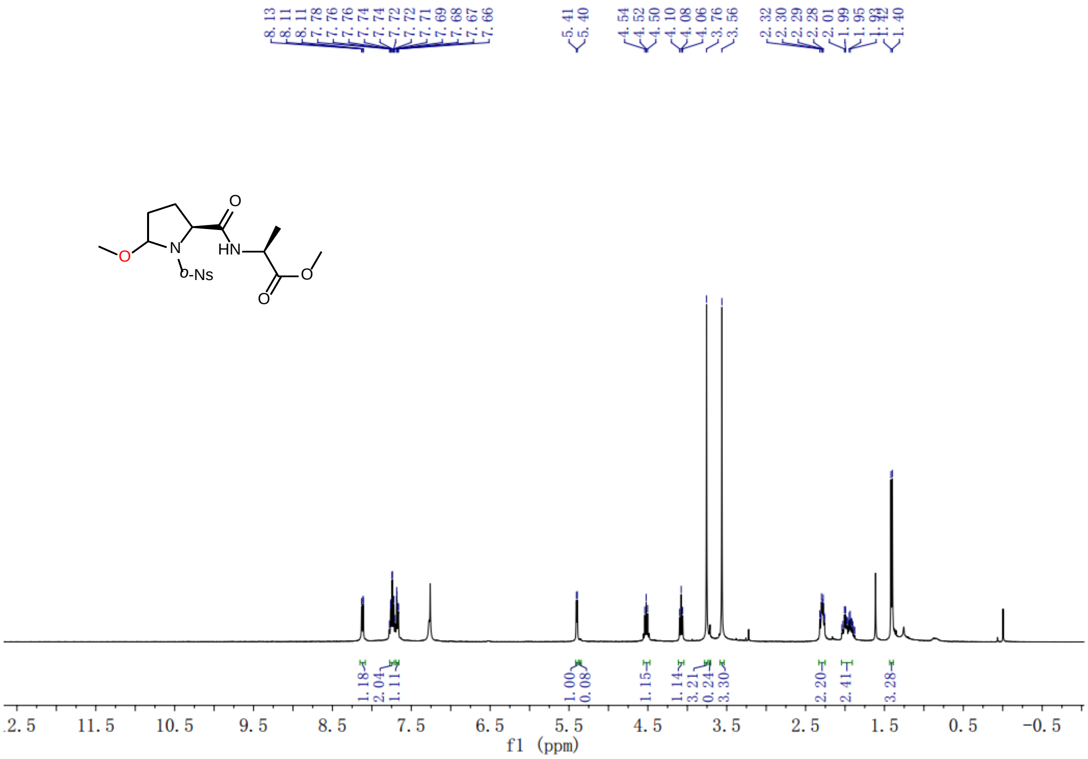

Supplementary Fig. 140. ^{1}H NMR spectrum of compound 2z4a.

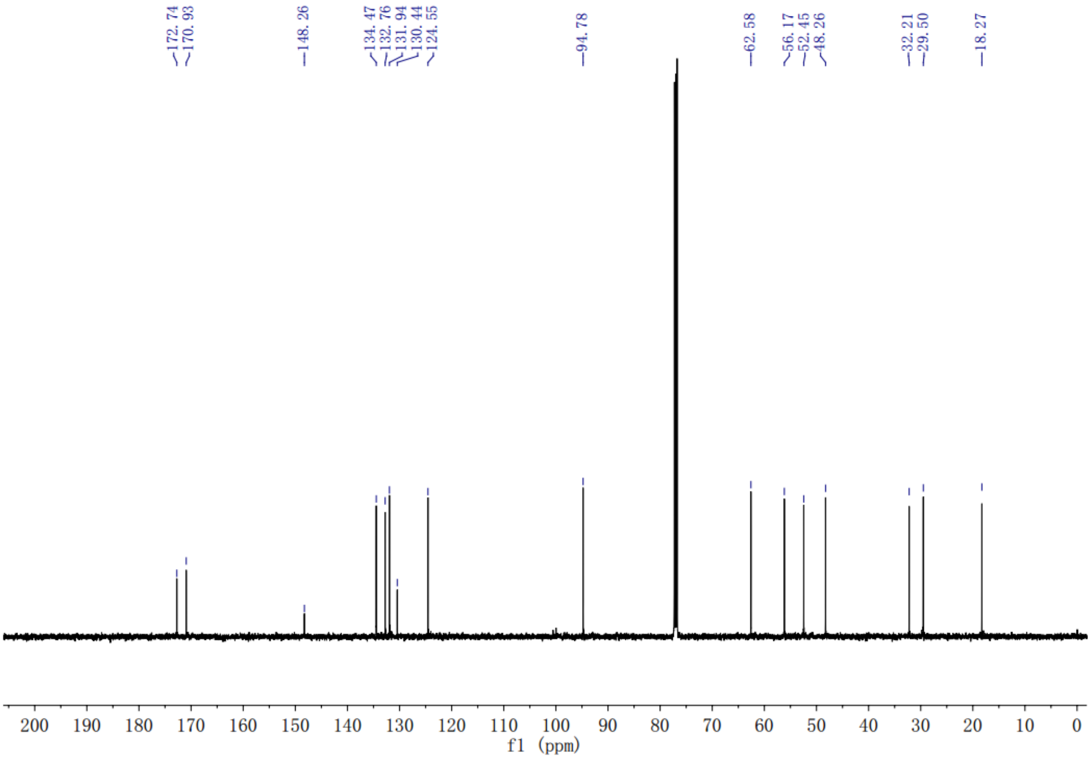

Supplementary Fig. 141. ^{1}H NMR spectrum of compound 2z4a.

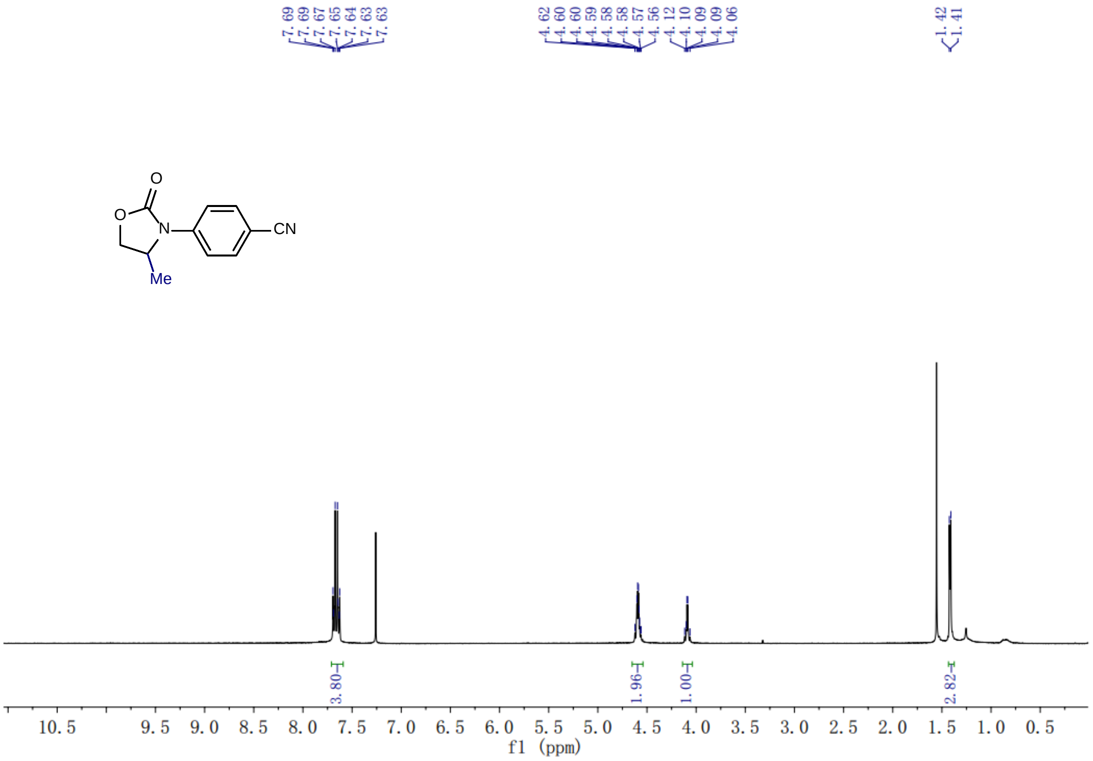

Supplementary Fig. 142. **^{1}H NMR spectrum of compound 3a.**

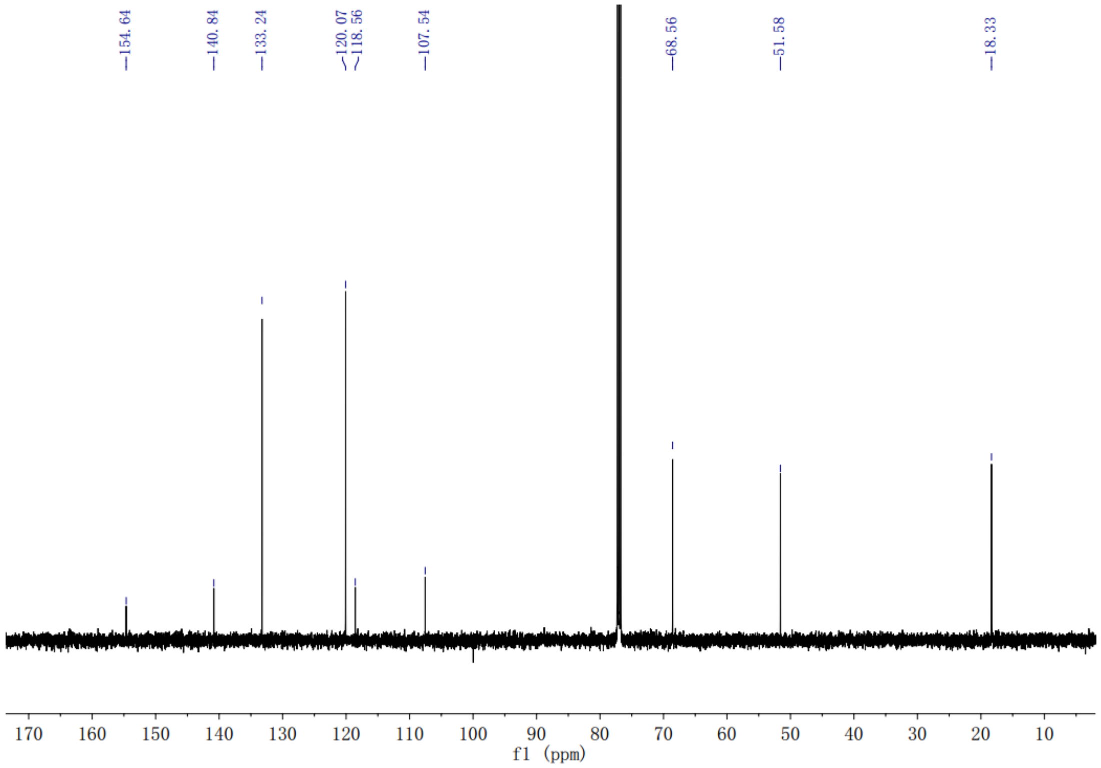

Supplementary Fig. 143. **^{13}C NMR spectrum of compound 3a.**

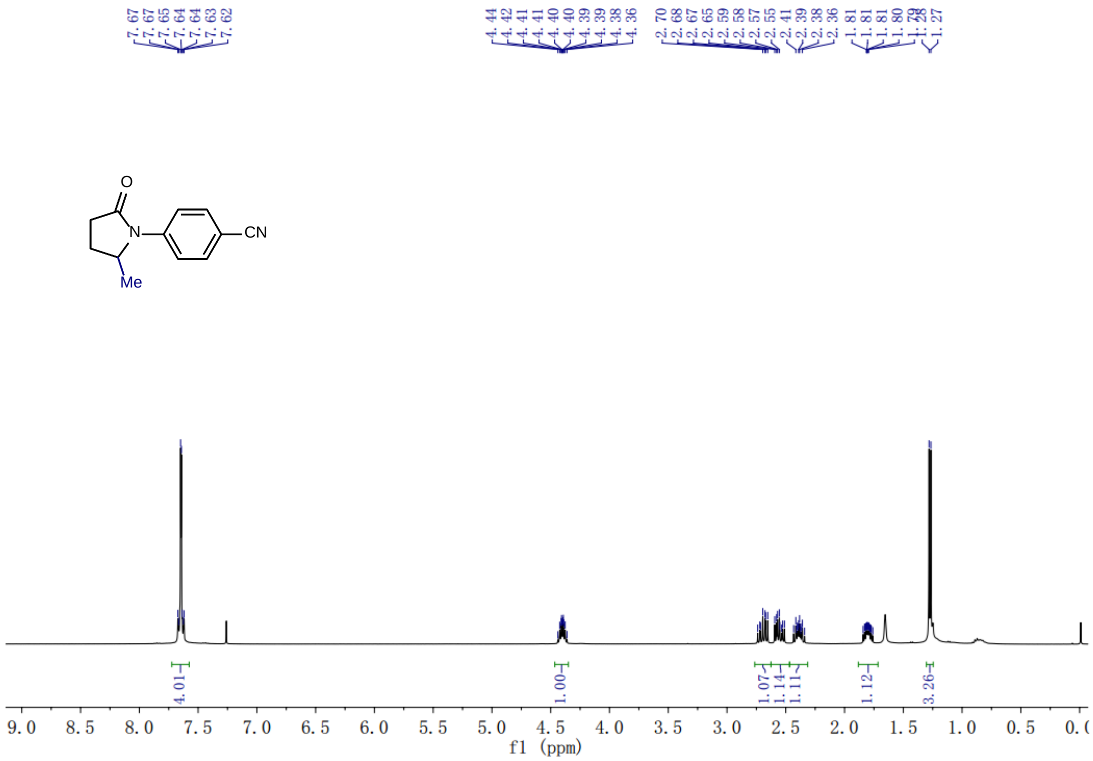

Supplementary Fig. 144. ^{1}H NMR spectrum of compound 3b.

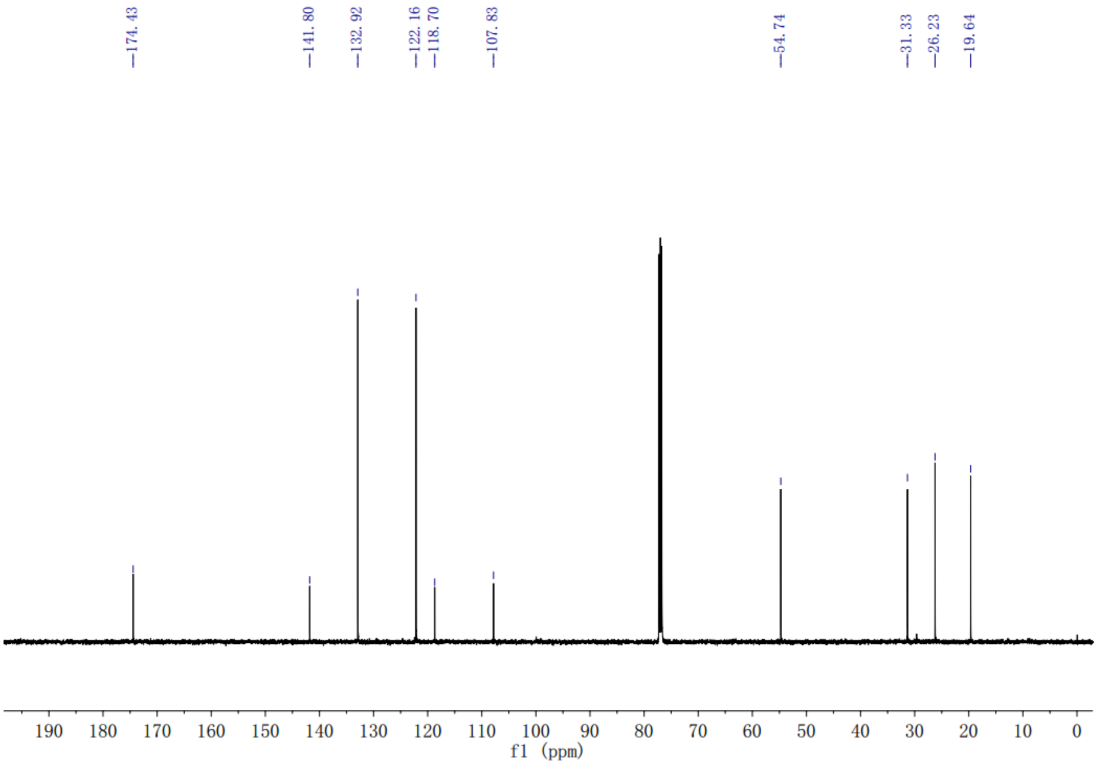

Supplementary Fig. 145. ^{13}C NMR spectrum of compound 3b.

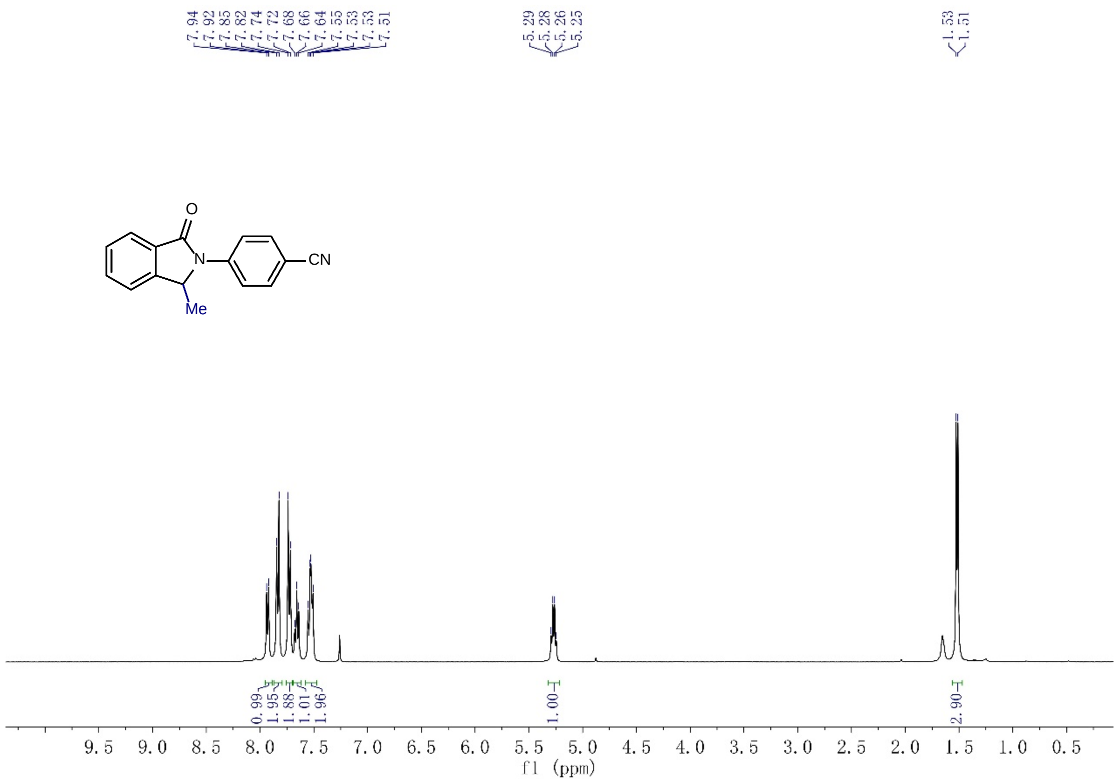

Supplementary Fig. 146. ^{1}**H NMR spectrum of compound 3c.**

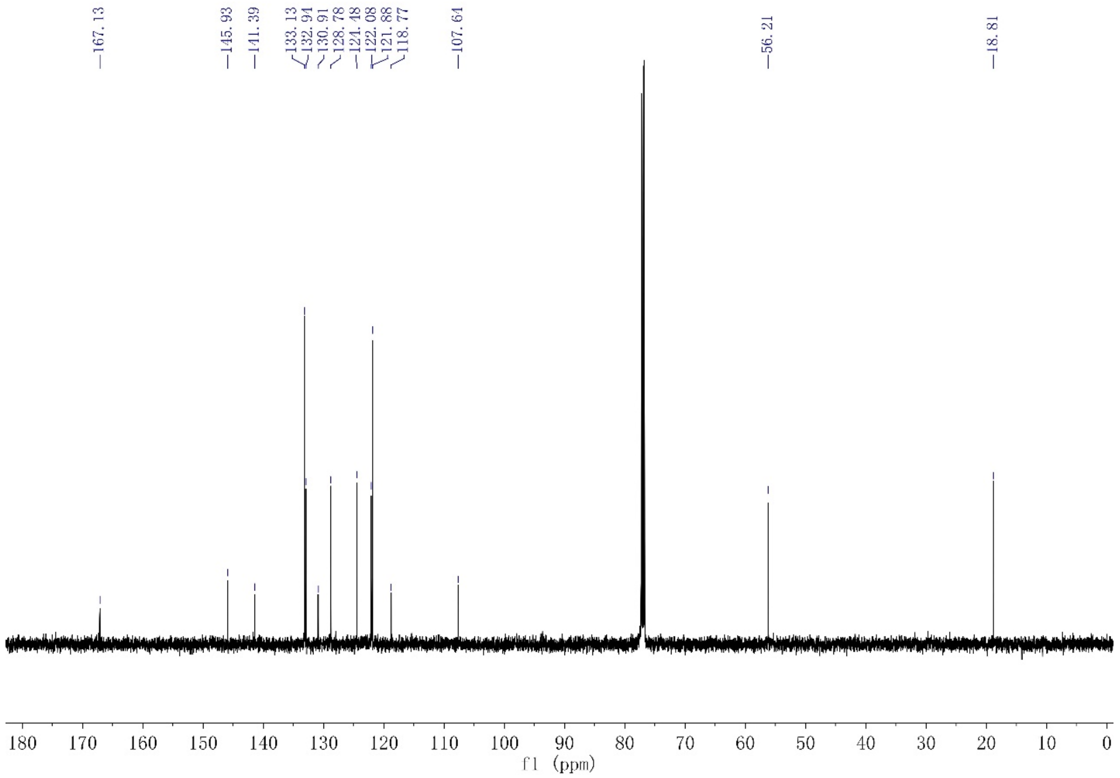

Supplementary Fig. 147. ^{13}**C NMR spectrum of compound 3c.**

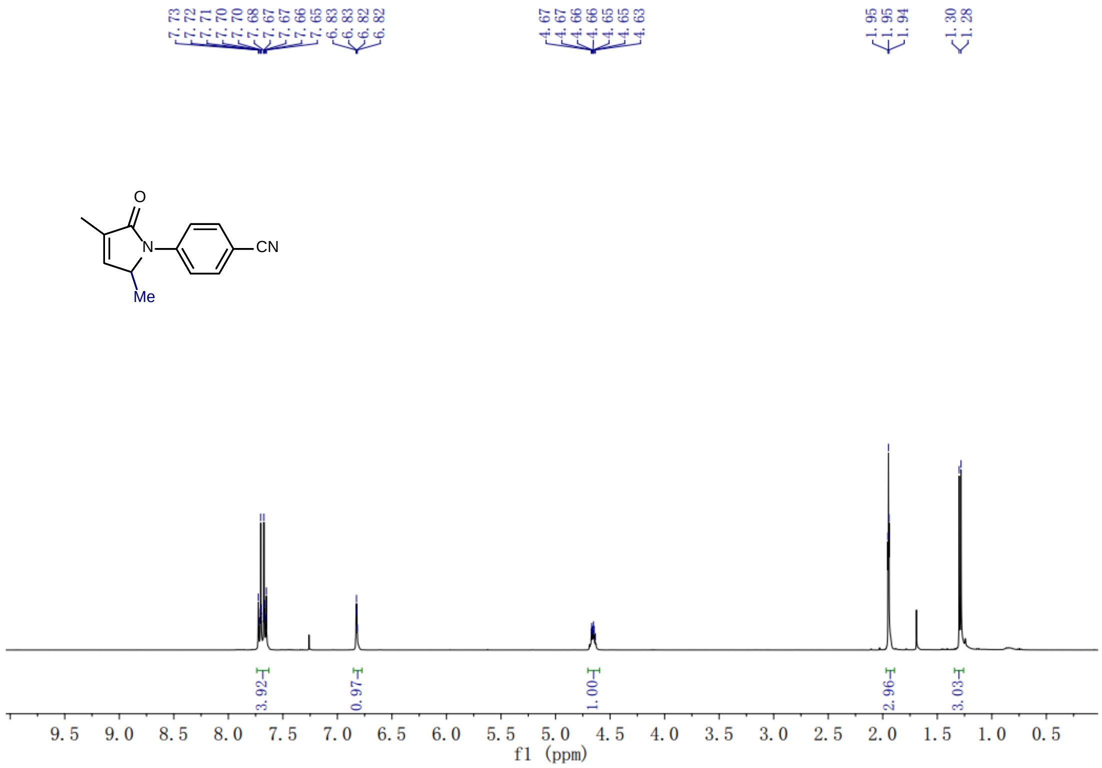

Supplementary Fig. 148. ^{1}**H NMR spectrum of compound 3d.**

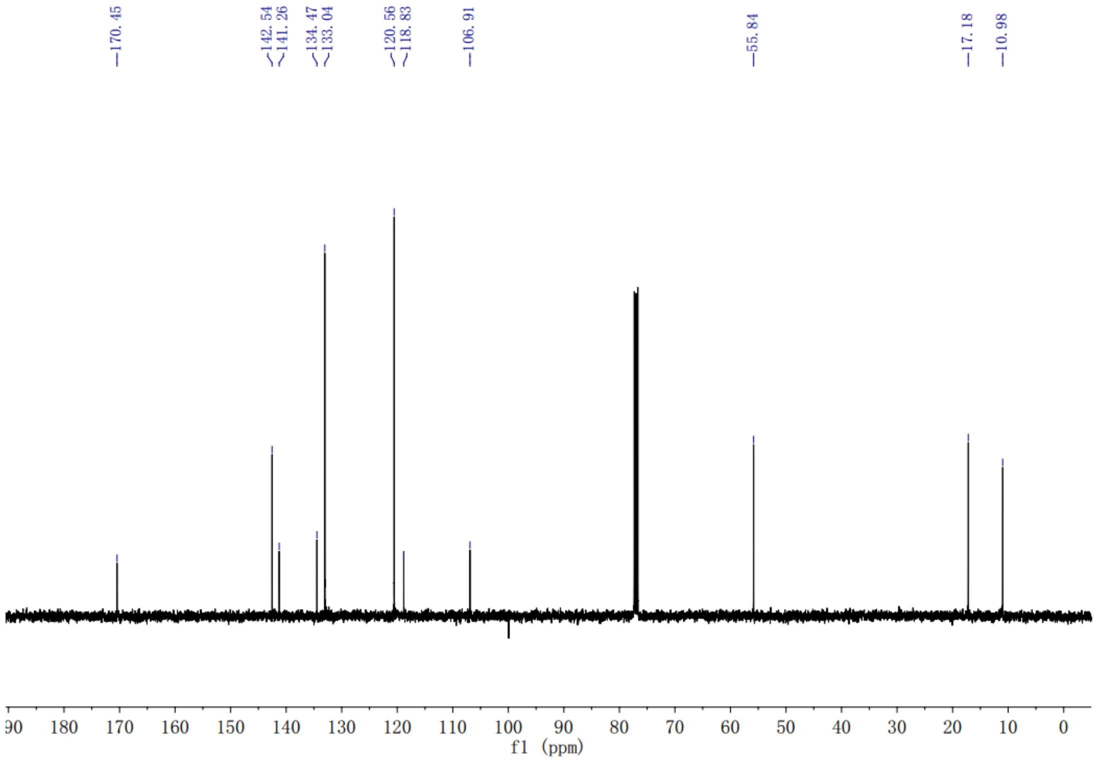

Supplementary Fig. 149. ^{13}**C NMR spectrum of compound 3d.**

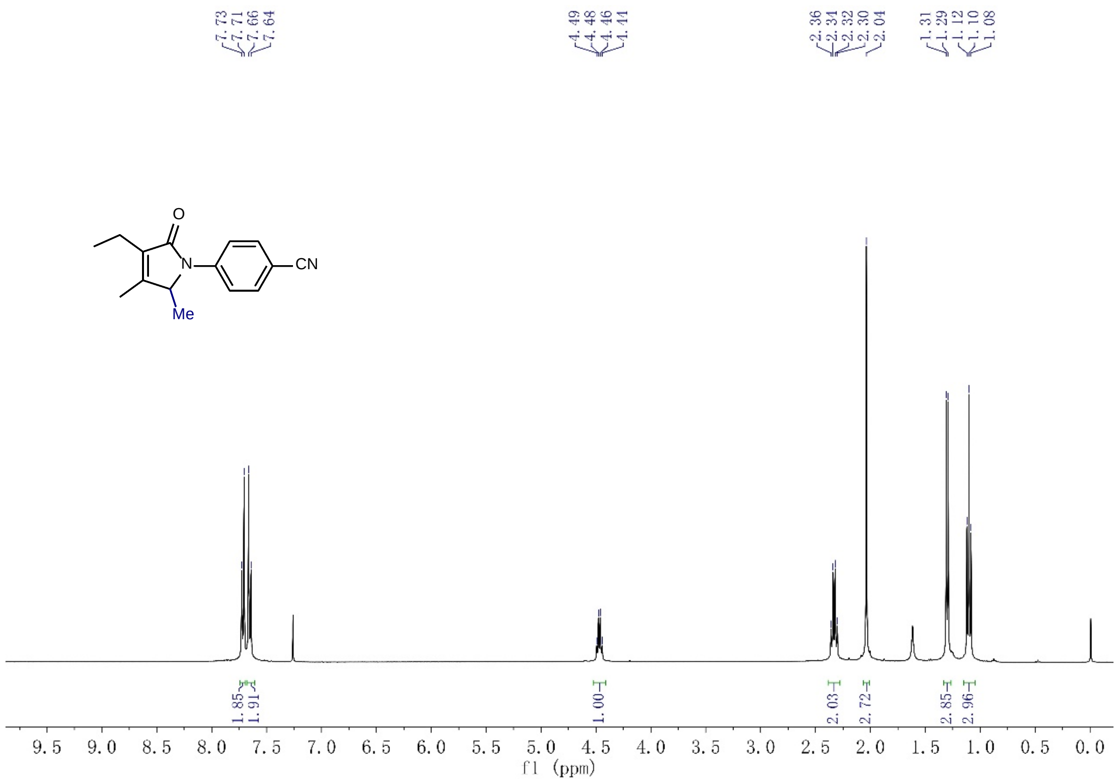

Supplementary Fig. 150. ^{1}H NMR spectrum of compound 3e.

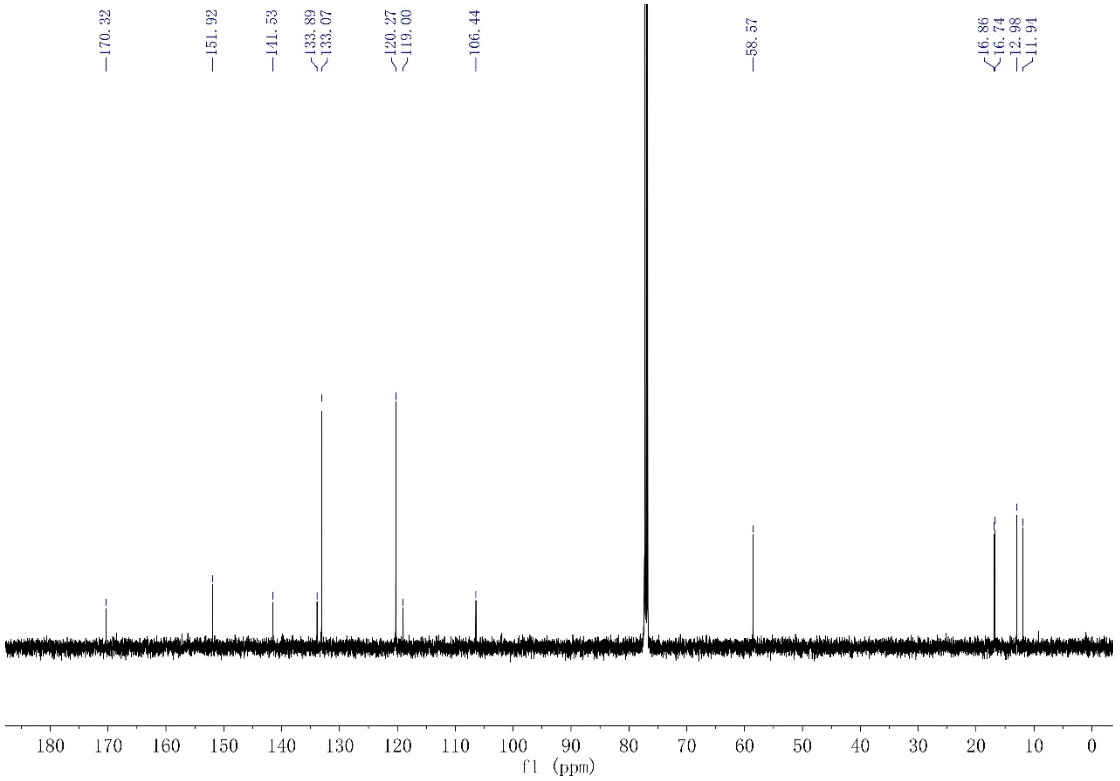

Supplementary Fig. 151. ^{13}C NMR spectrum of compound 3e.

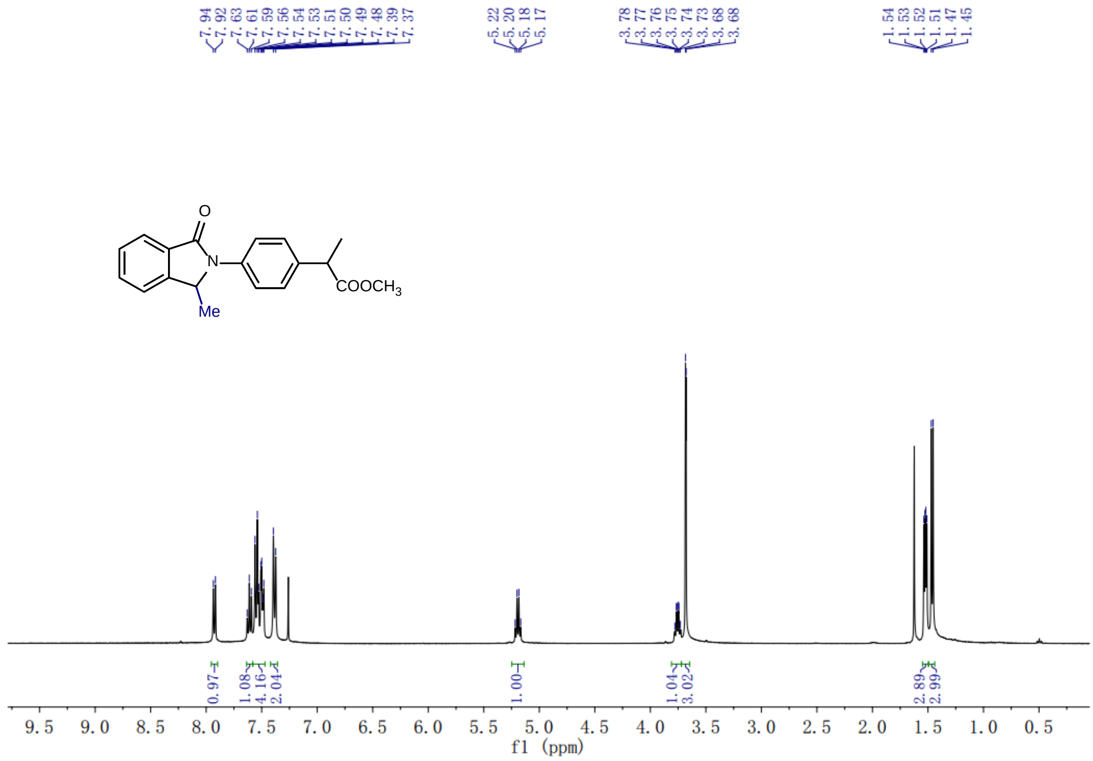

Supplementary Fig. 152. ^{1}**H NMR spectrum of compound 3f.**

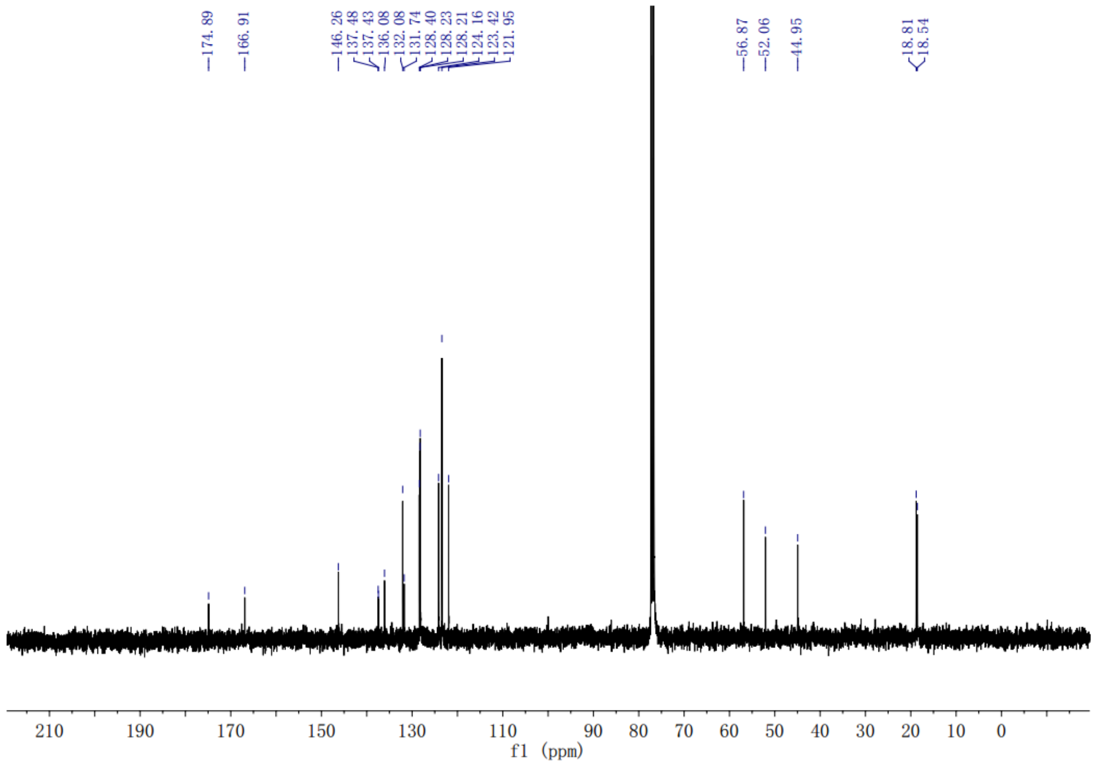

Supplementary Fig. 153. ^{13}**C NMR spectrum of compound 3f.**

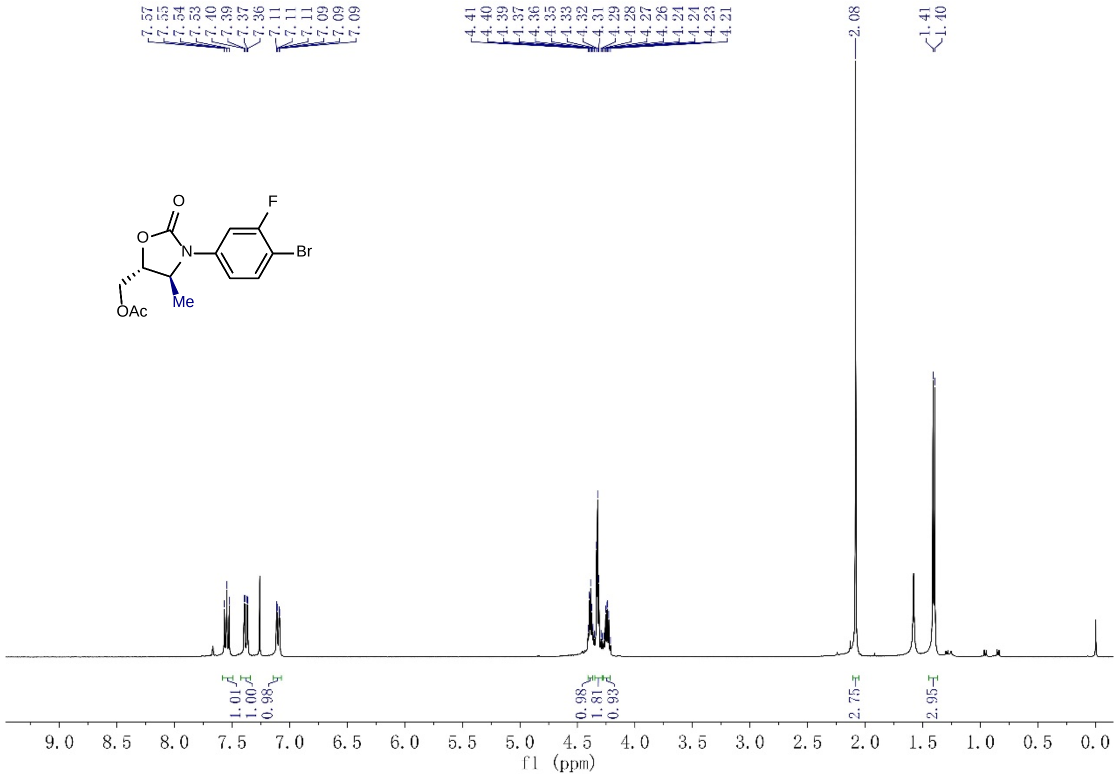

Supplementary Fig. 154. ^{1}H NMR spectrum of compound 3g.

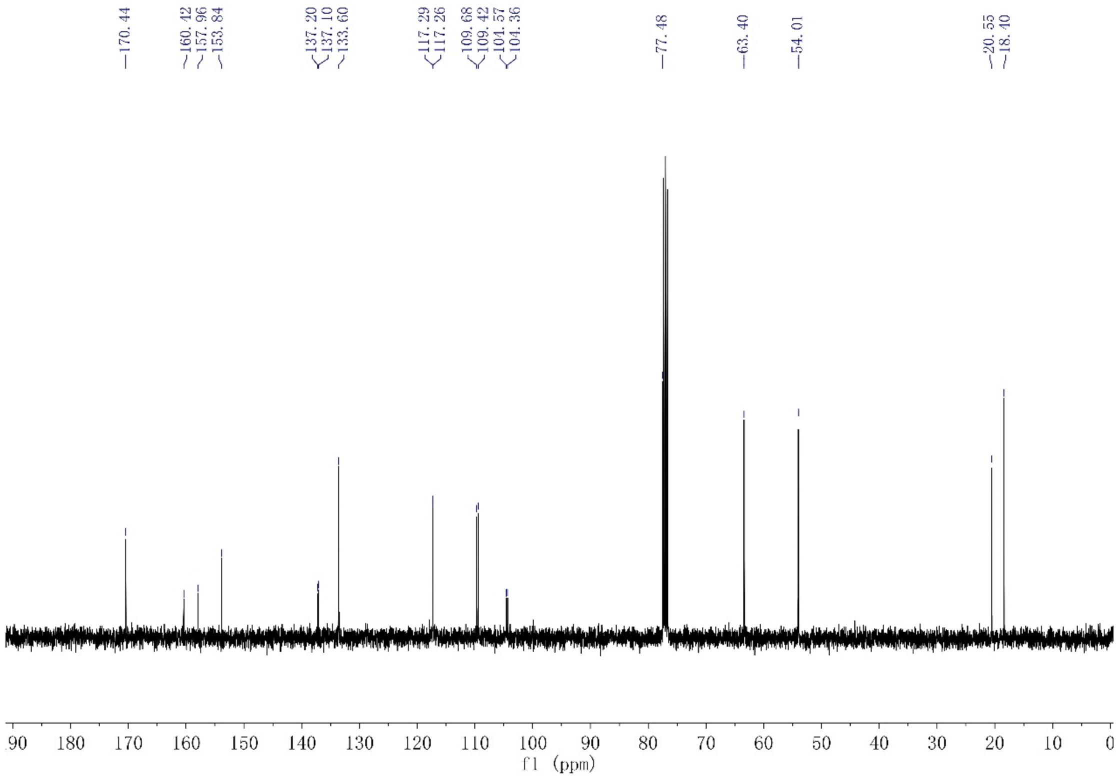

Supplementary Fig. 155. ^{13}C NMR spectrum of compound 3g.

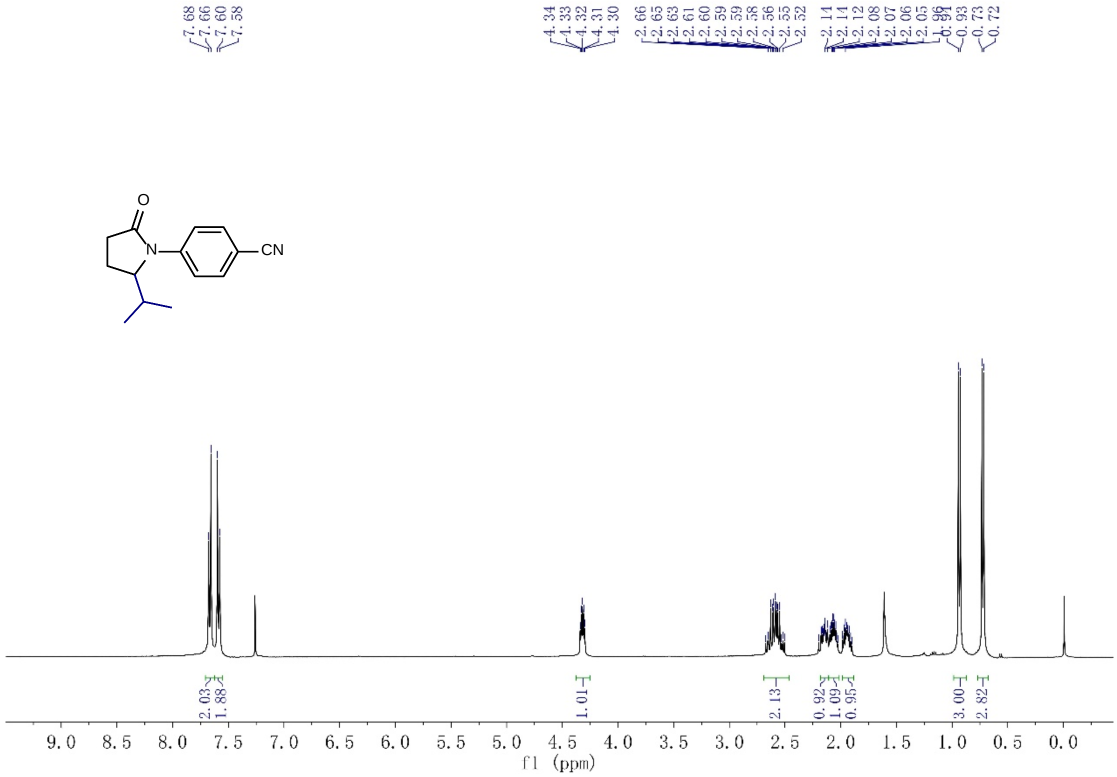

Supplementary Fig. 156. ^{1}H NMR spectrum of compound 3h.

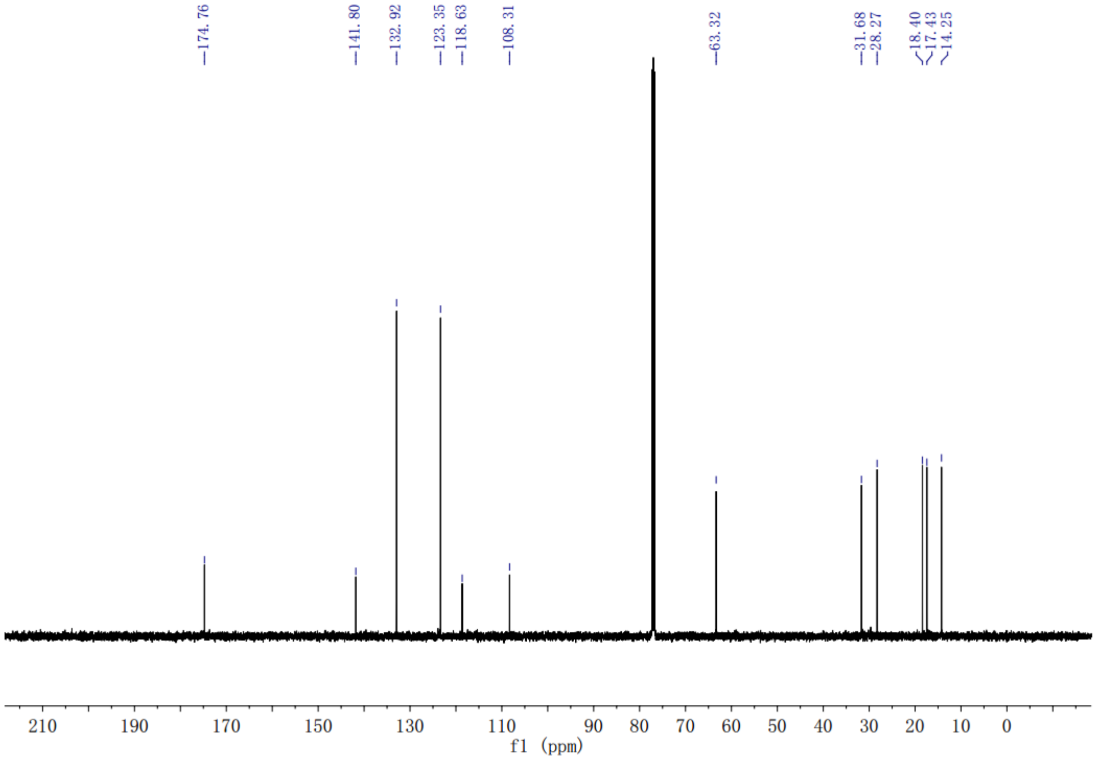

Supplementary Fig. 157. ^{13}C NMR spectrum of compound 3h.

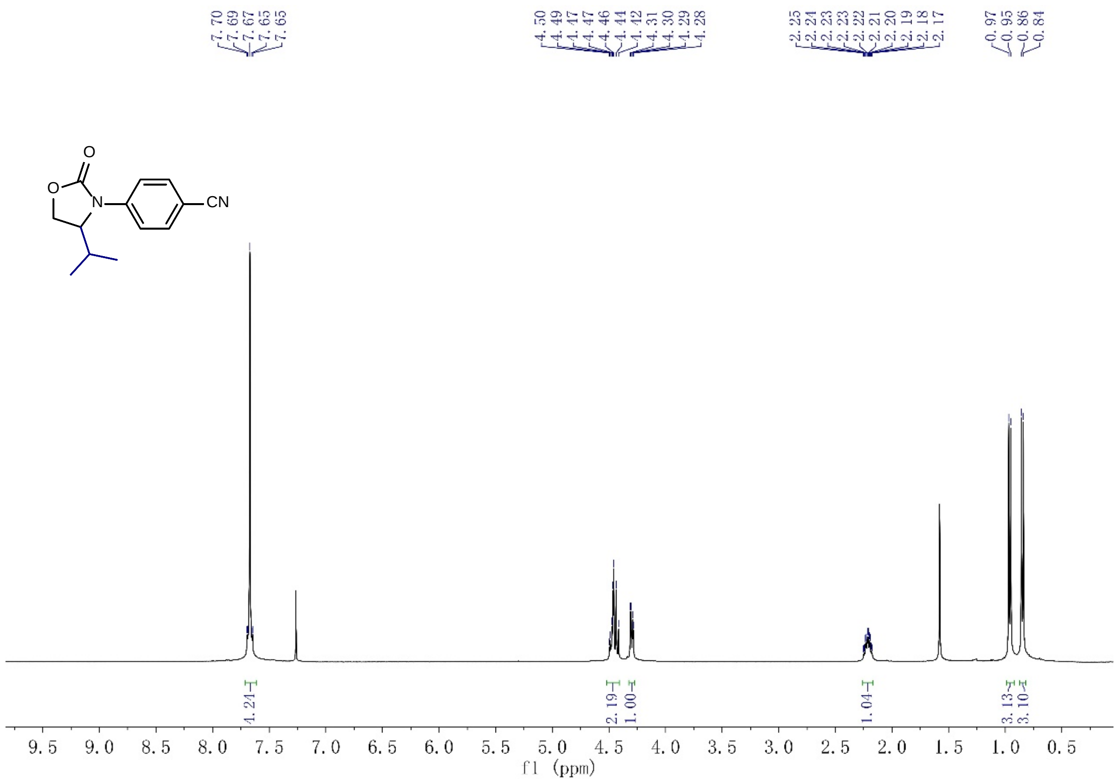

Supplementary Fig. 158. ^{1}H NMR spectrum of compound 3i.

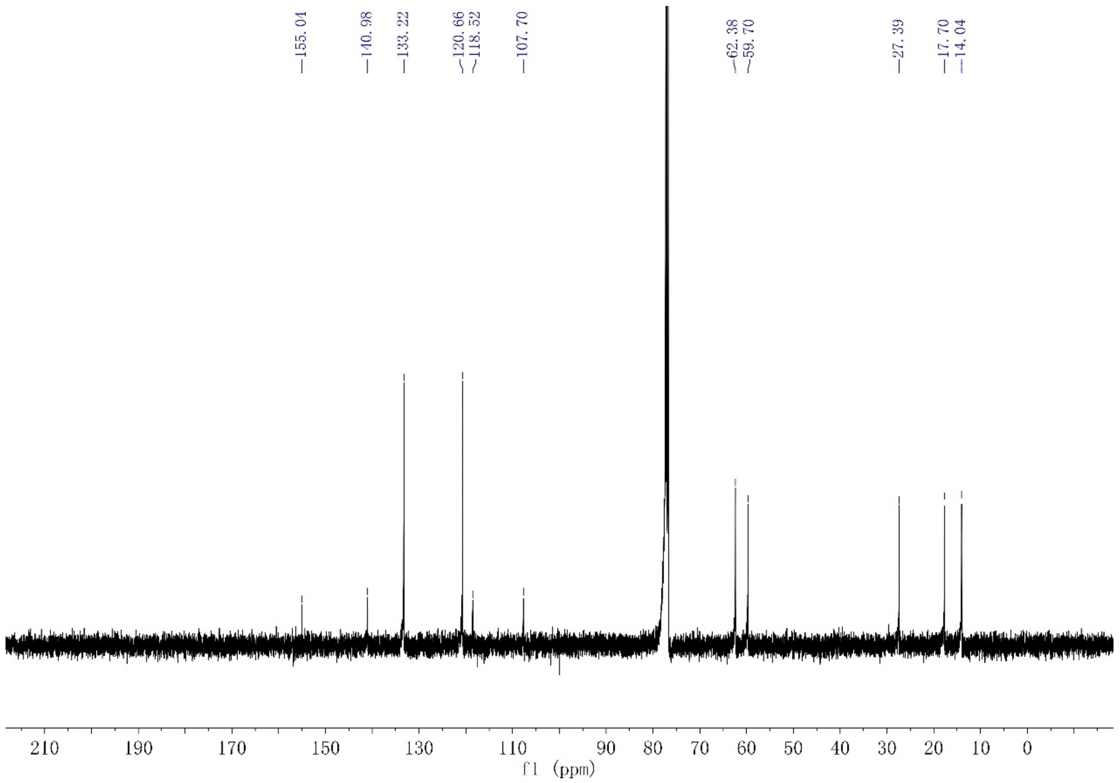

Supplementary Fig. 159. ^{13}C NMR spectrum of compound 3i.

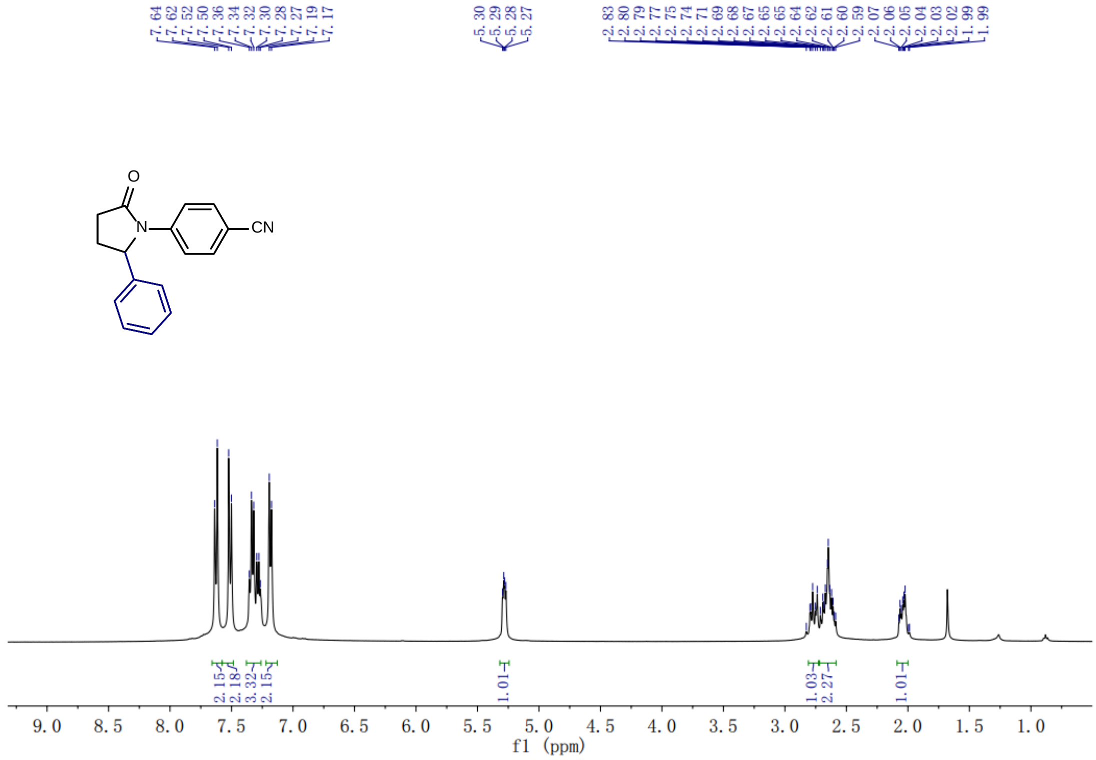

Supplementary Fig. 160. **^{1}H NMR spectrum of compound 3j.**

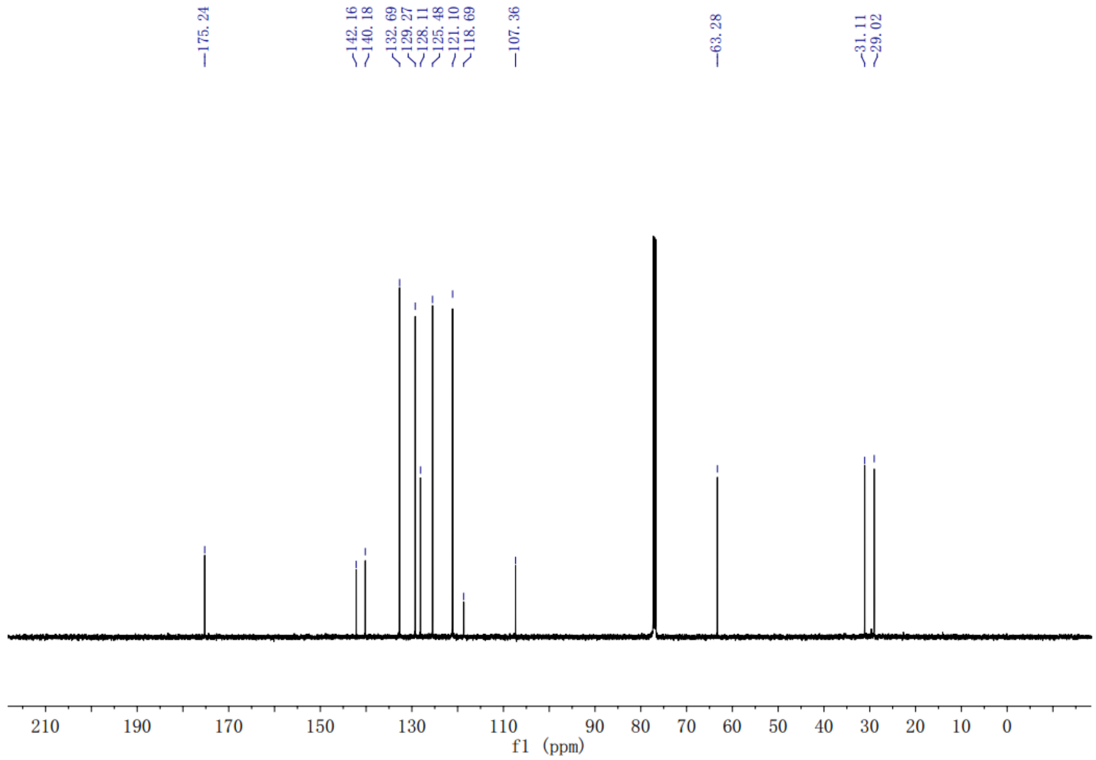

Supplementary Fig. 161. **^{13}C NMR spectrum of compound 3j.**

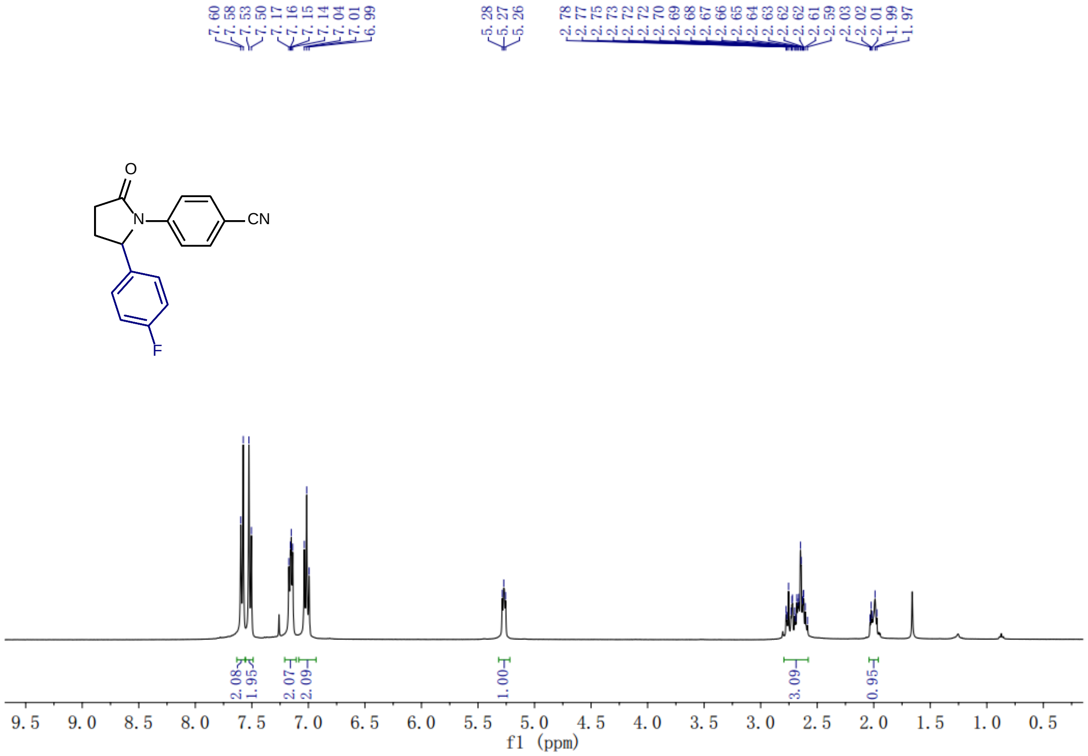

Supplementary Fig. 162. ^{1}H NMR spectrum of compound 3k.

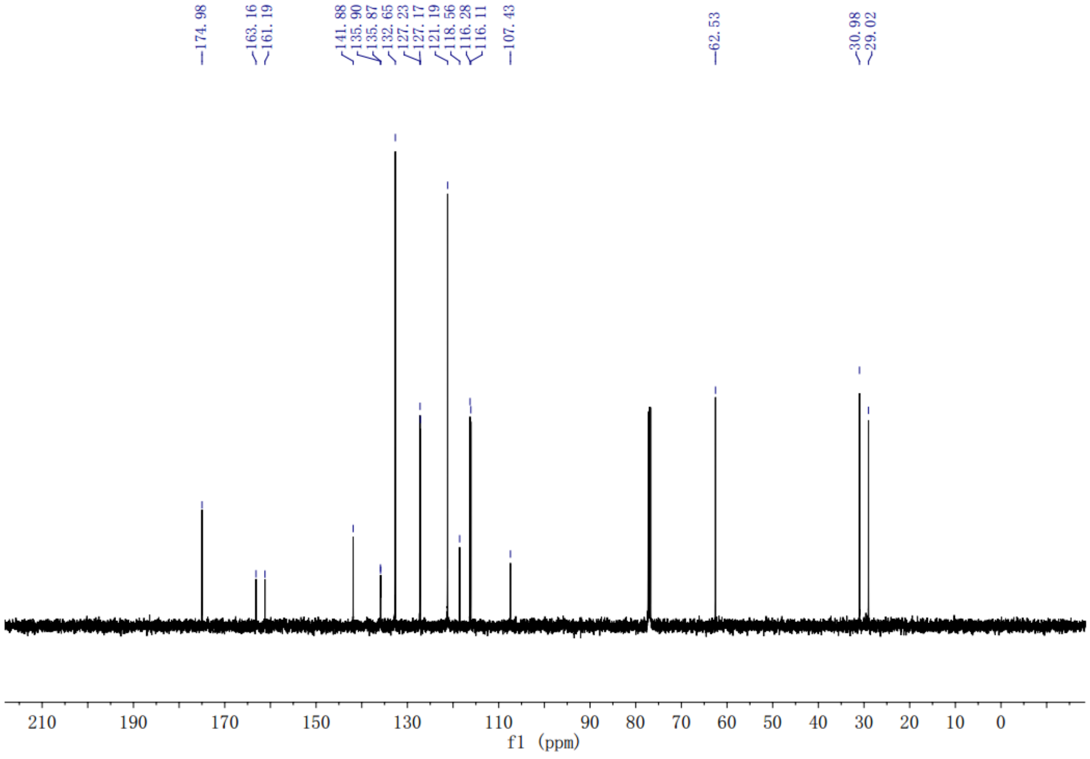

Supplementary Fig. 163. ^{13}C NMR spectrum of compound 3k.

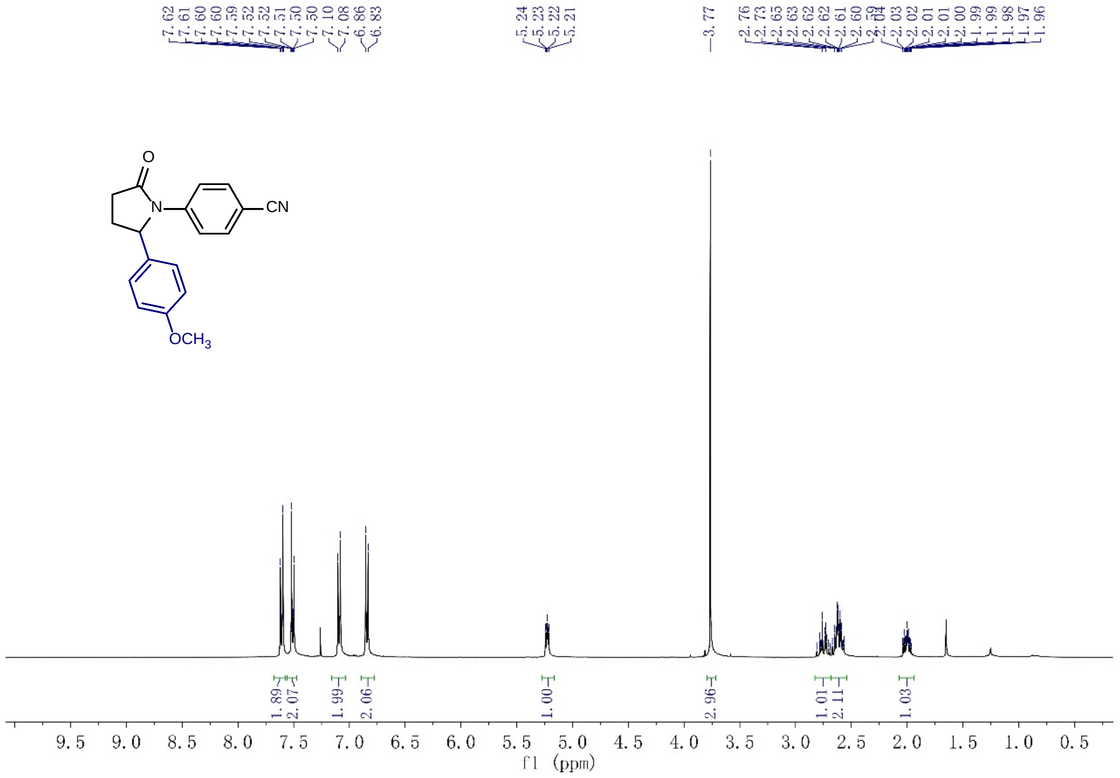

Supplementary Fig. 164. ^{1}H NMR spectrum of compound 3l.

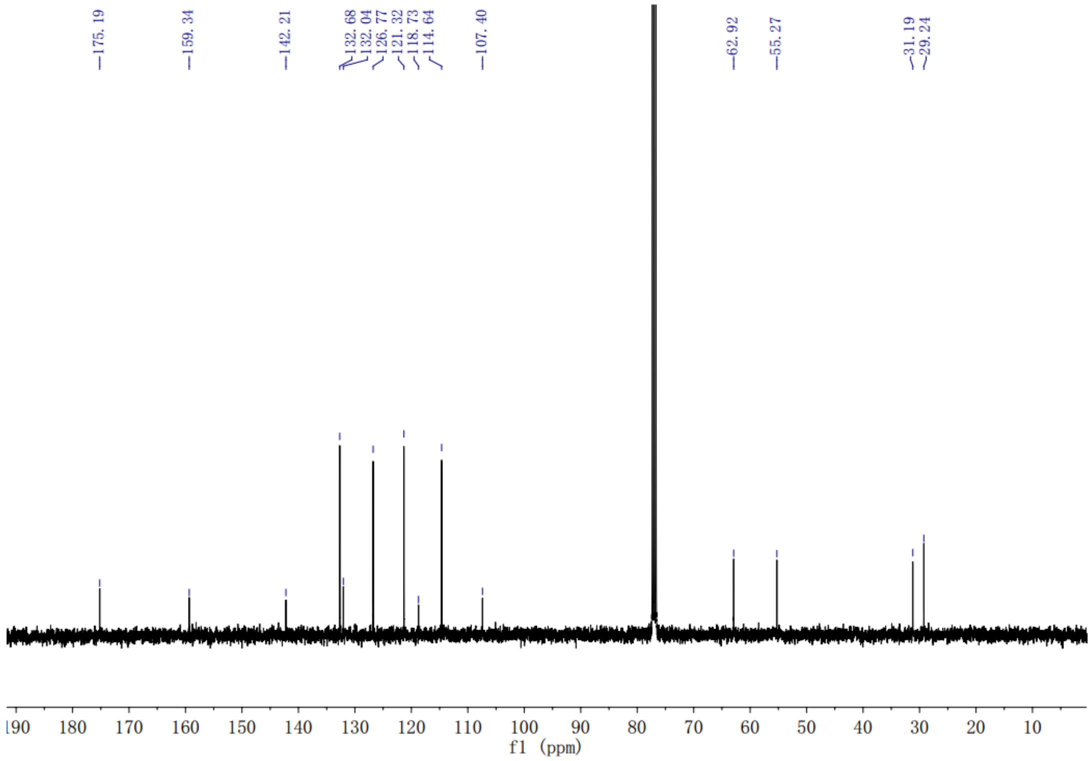

Supplementary Fig. 165. ^{13}C NMR spectrum of compound 3l.

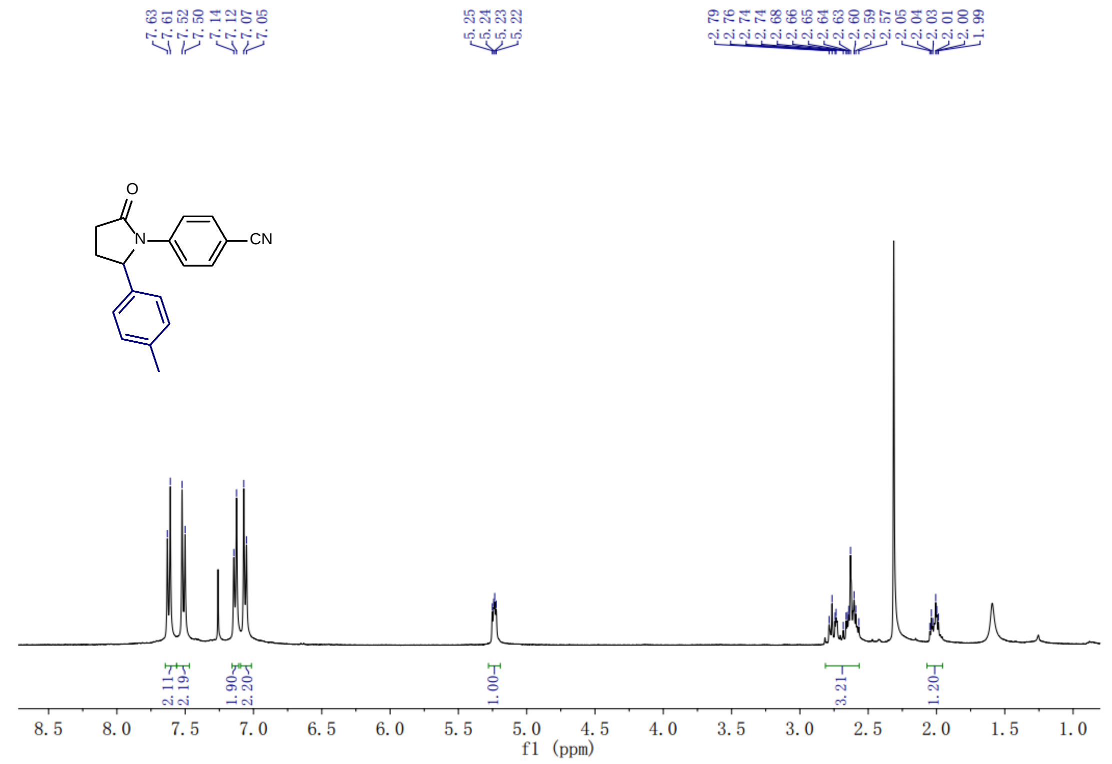

Supplementary Fig. 166. ^{1}H NMR spectrum of compound 3m.

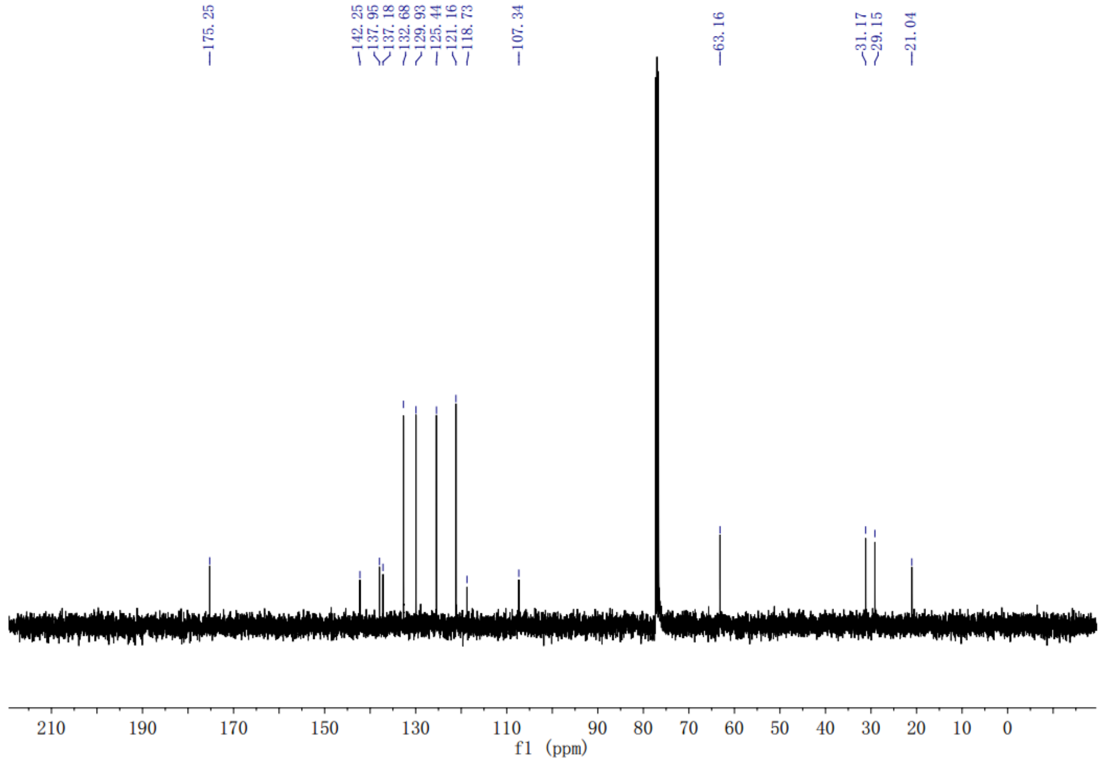

Supplementary Fig. 167. ^{13}C NMR spectrum of compound 3m.

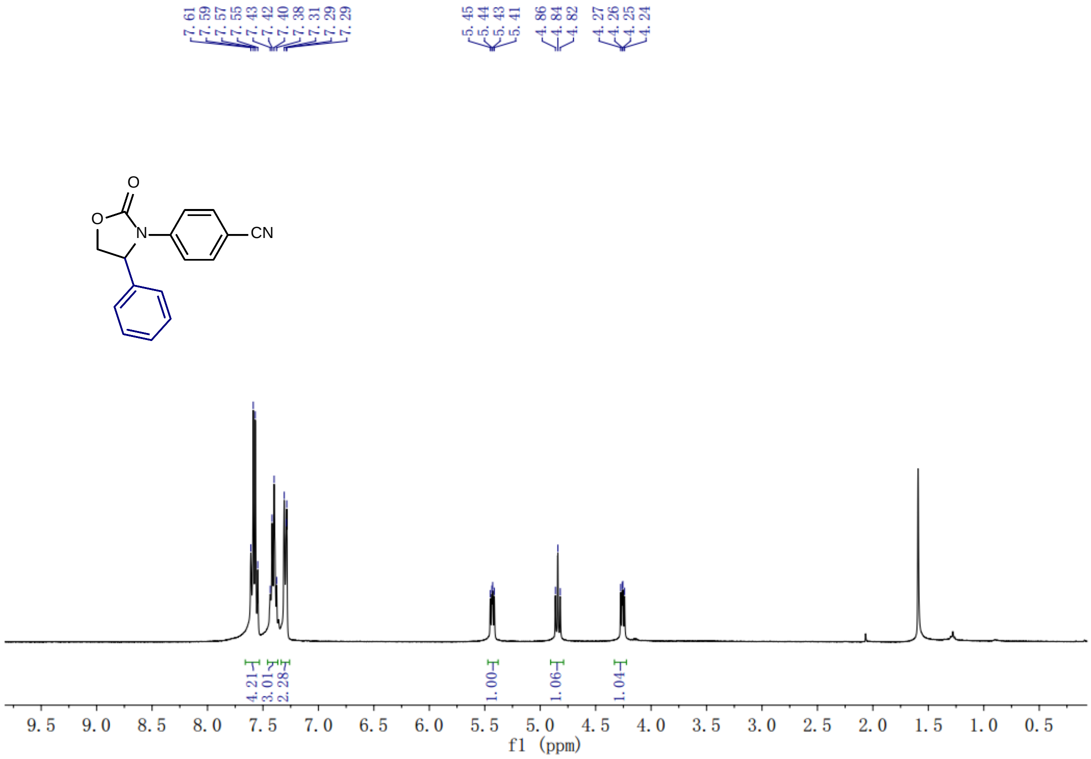

Supplementary Fig. 168. ^{1}H NMR spectrum of compound 3n.

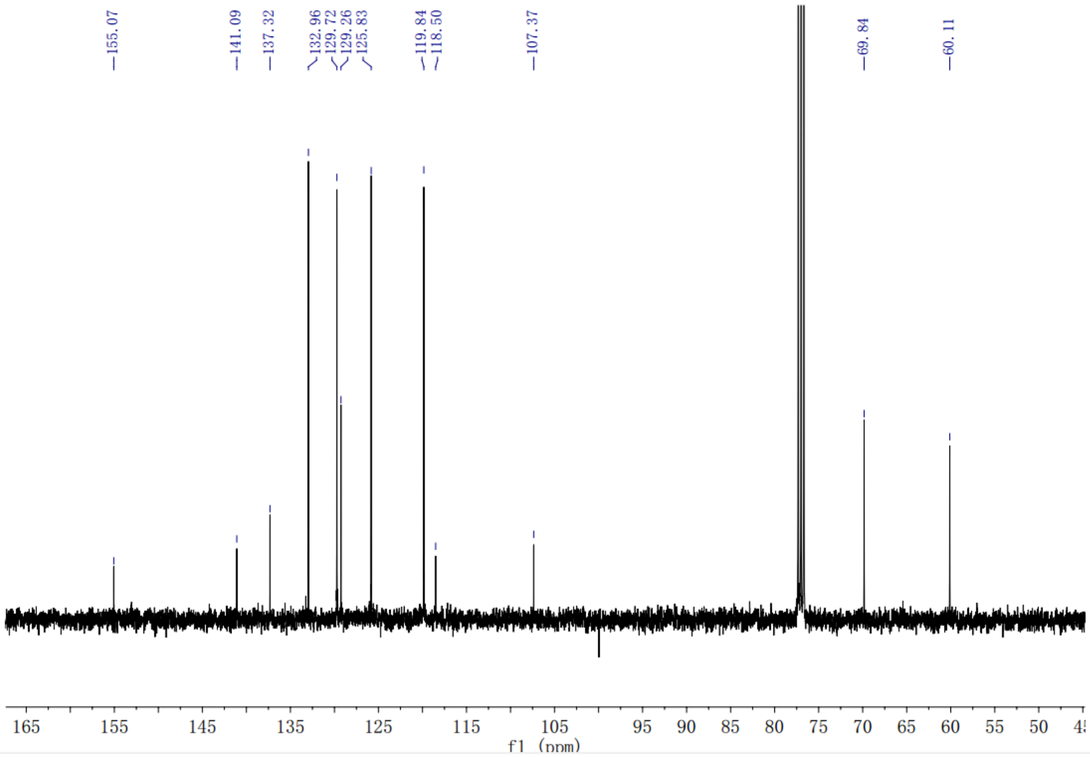

Supplementary Fig. 169. ^{13}C NMR spectrum of compound 3n.

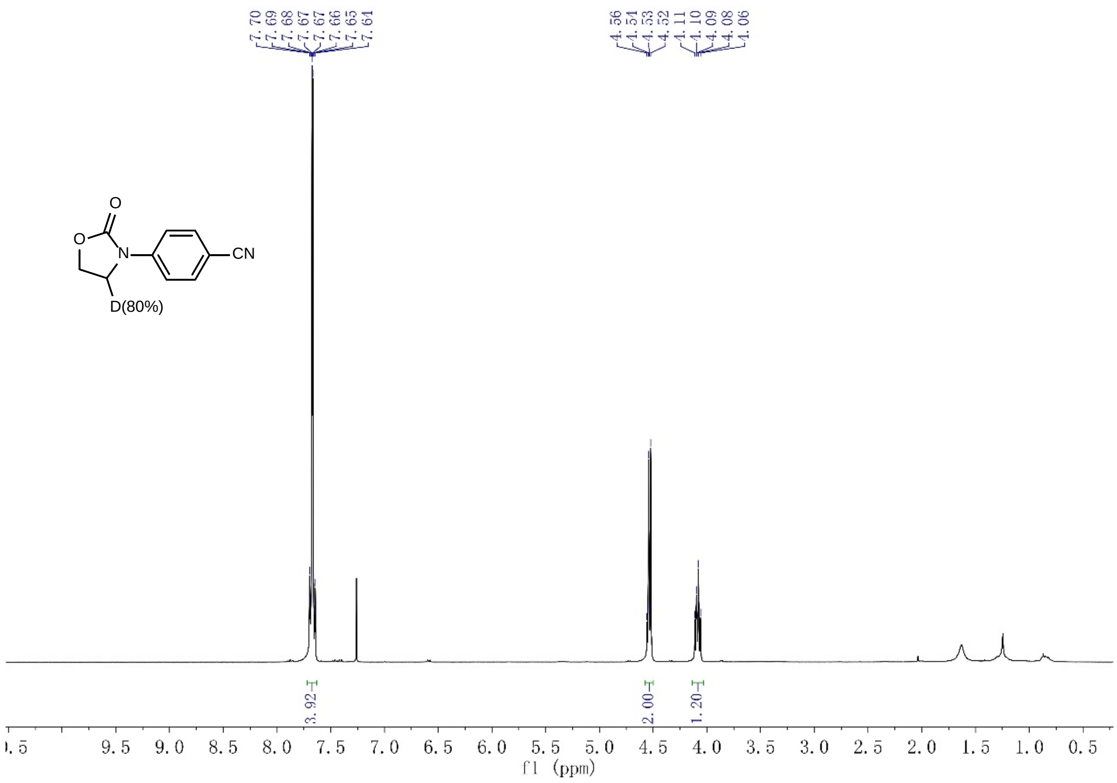

Supplementary Fig. 170. ^{1}**H NMR spectrum of compound 1a-[D].**

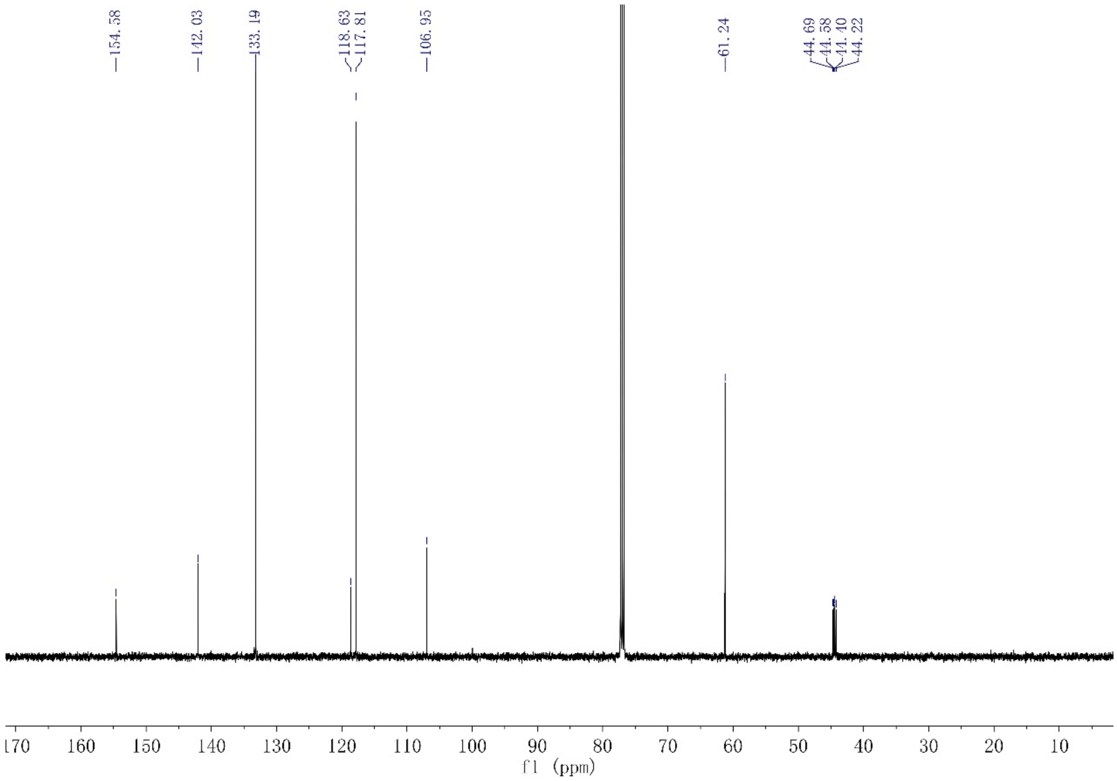

Supplementary Fig. 171. ^{13}**C NMR spectrum of compound 1a-[D].**

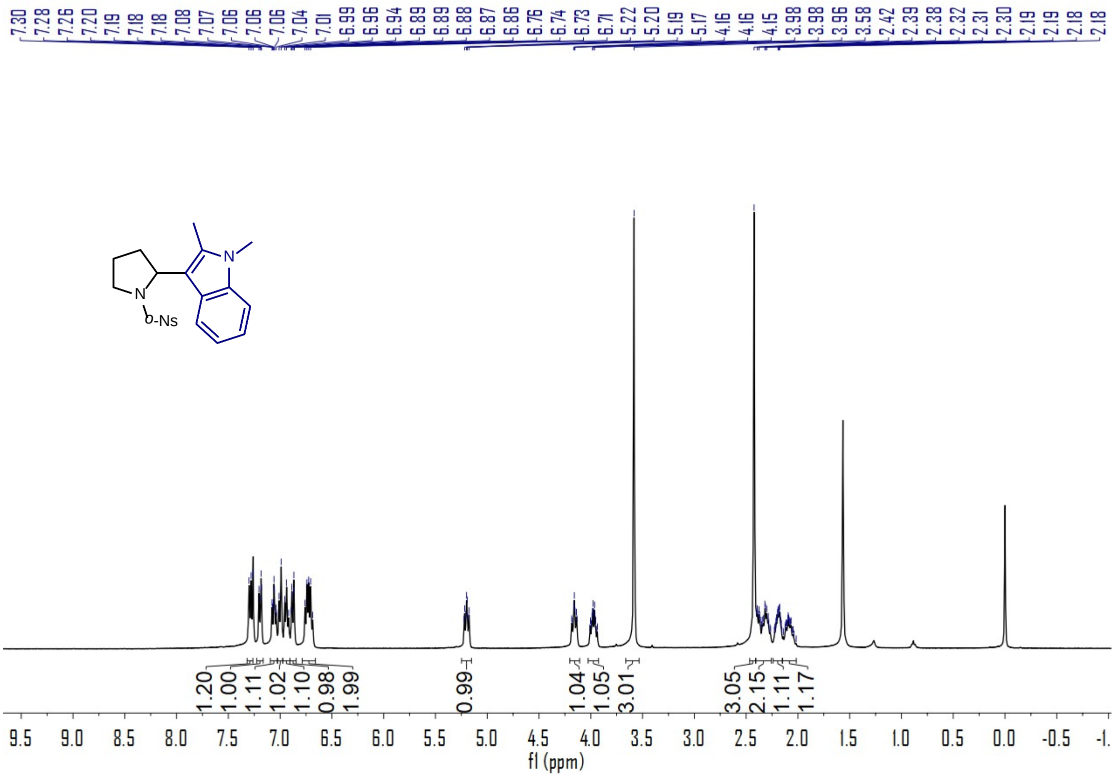

Supplementary Fig. 172. ^{1}H NMR spectrum of compound 3o.

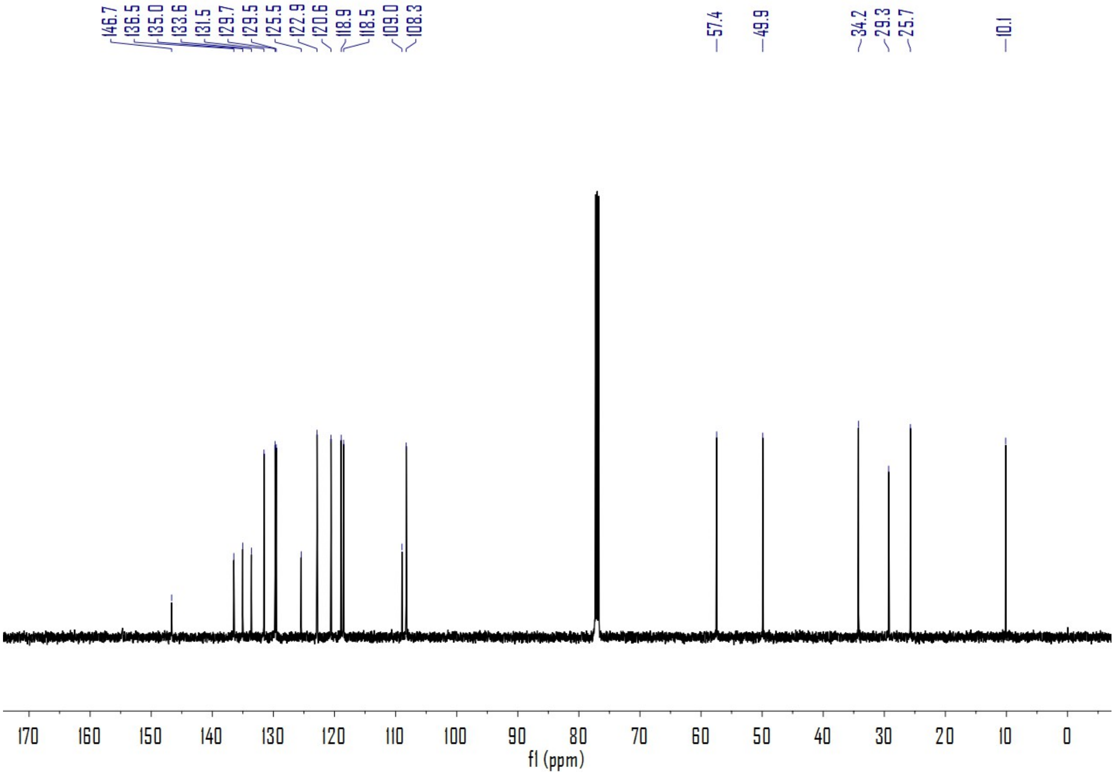

Supplementary Fig. 173. ^{13}C NMR spectrum of compound 3o.

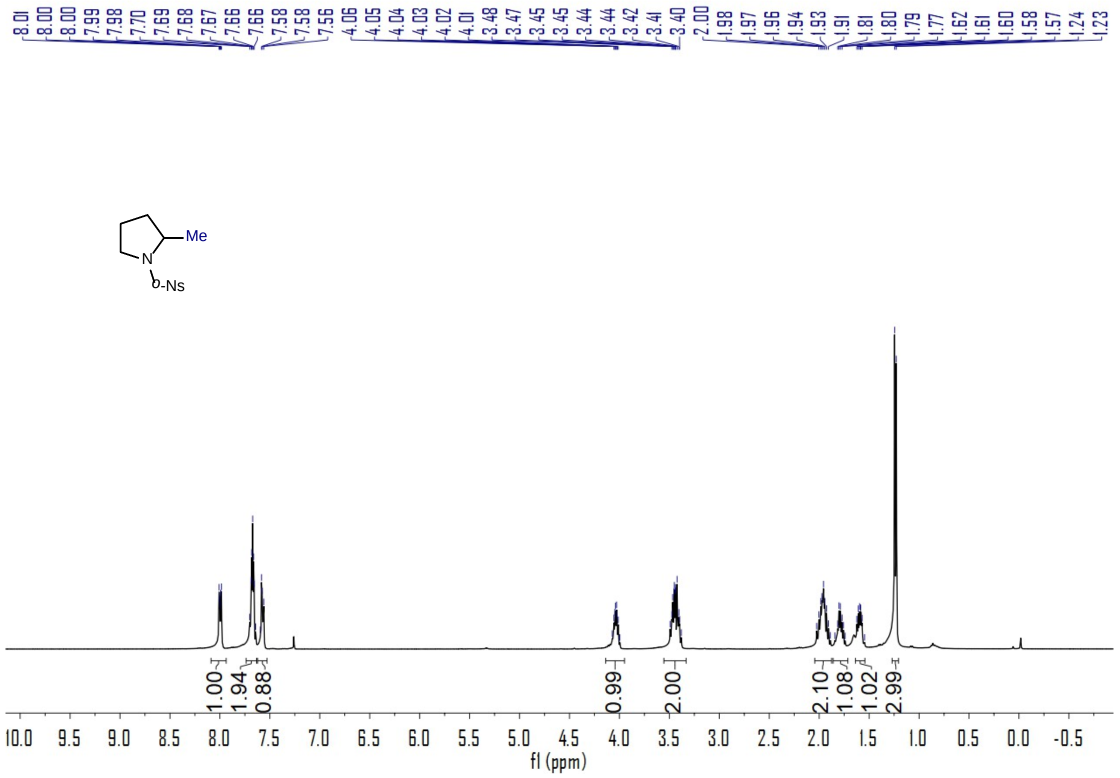

Supplementary Fig. 174. ^{1}H NMR spectrum of compound 3p.

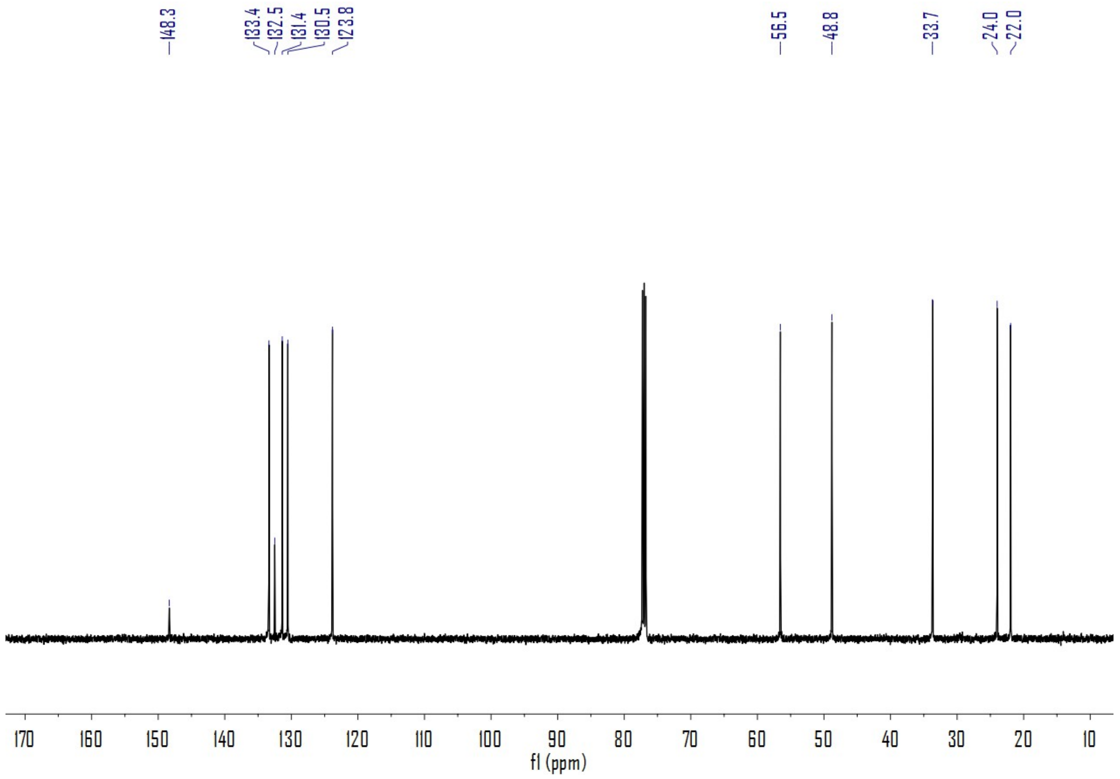

Supplementary Fig. 175. ^{13}C NMR spectrum of compound 3p.

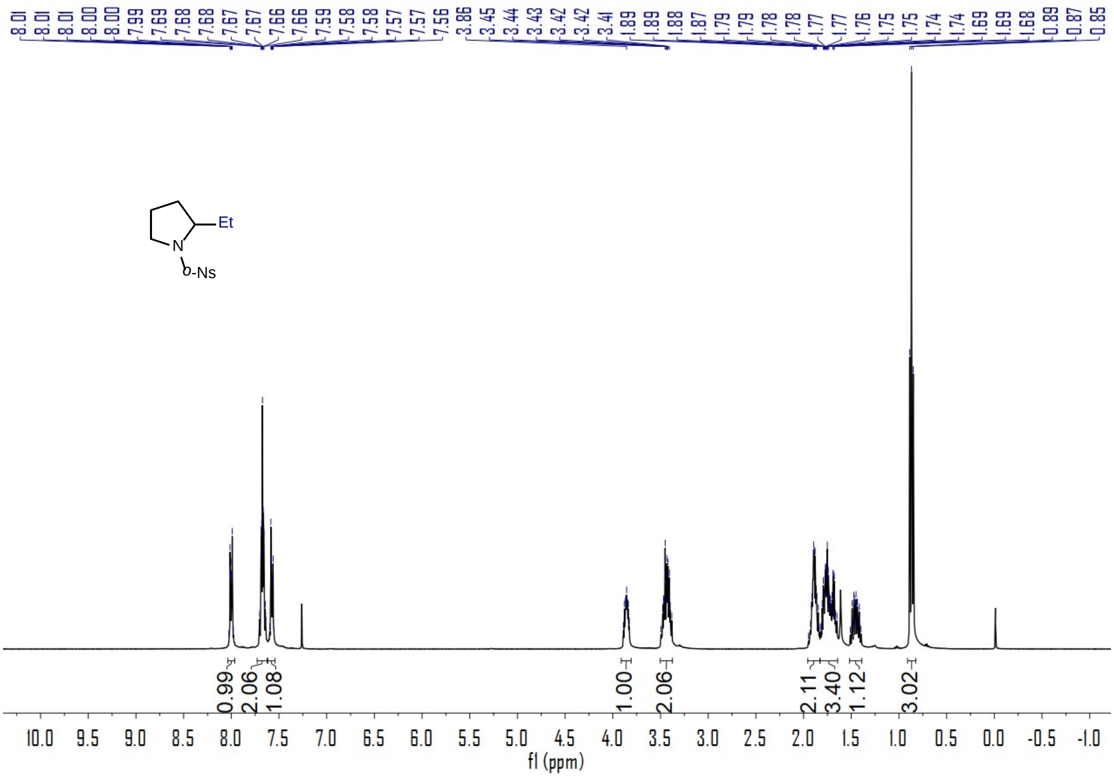

Supplementary Fig. 176. ^{1}**H NMR spectrum of compound 3q.**

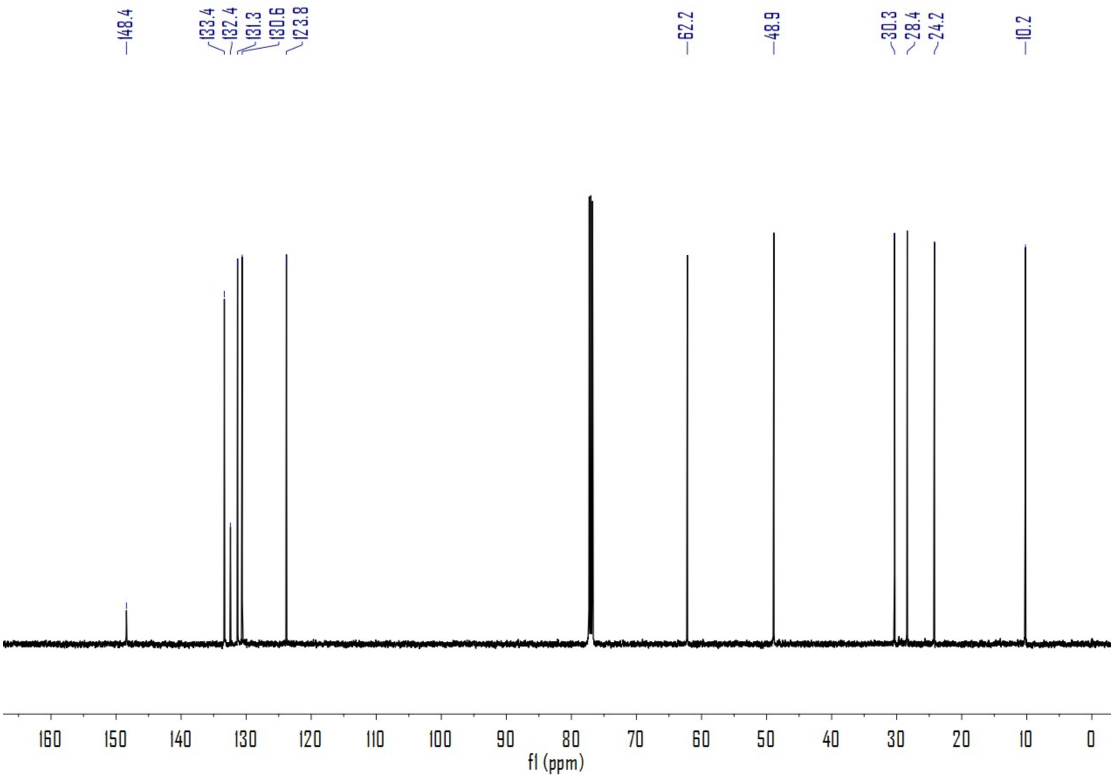

Supplementary Fig. 177. ^{13}**C NMR spectrum of compound 3q.**

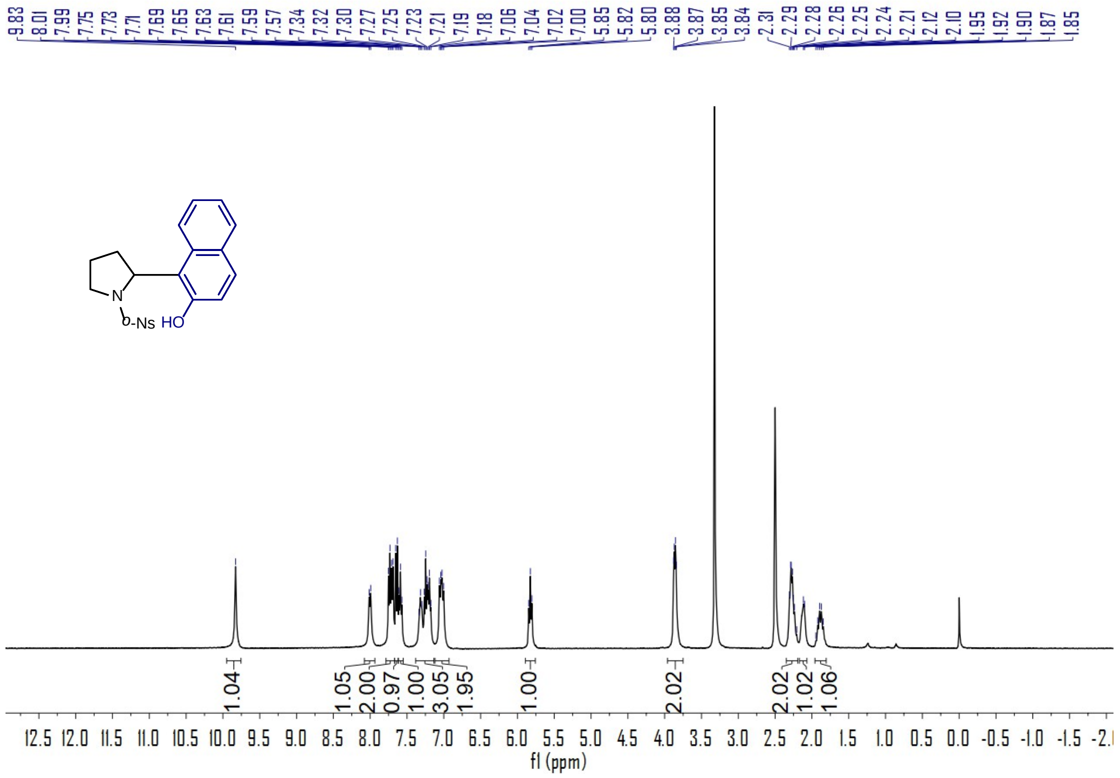

Supplementary Fig. 178. ^{1}H NMR spectrum of compound 3r.

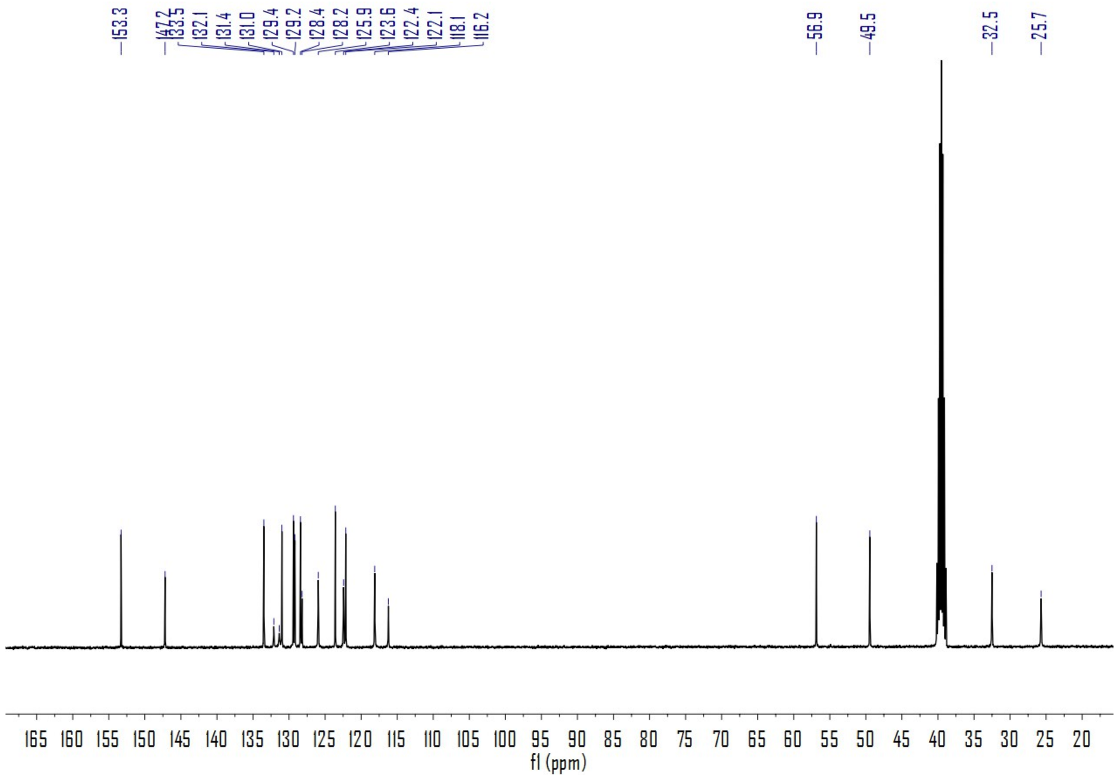

Supplementary Fig. 179. ^{13}C NMR spectrum of compound 3r.

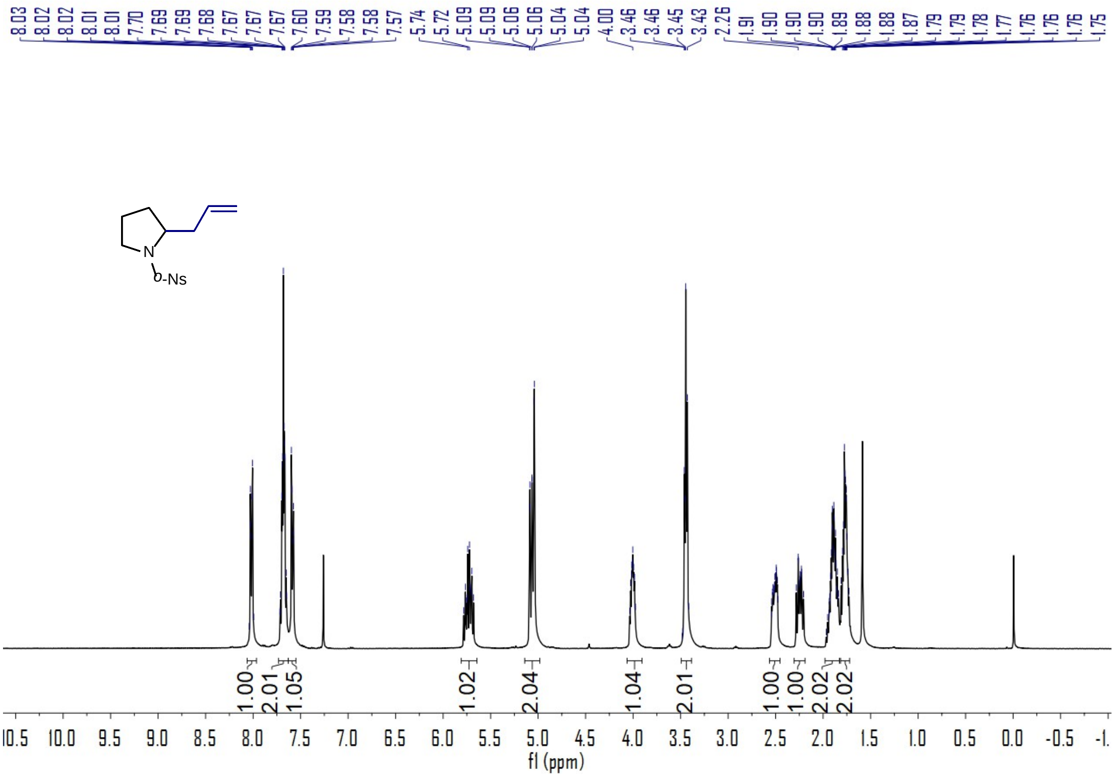

Supplementary Fig. 180. ^{1}H NMR spectrum of compound 3s.

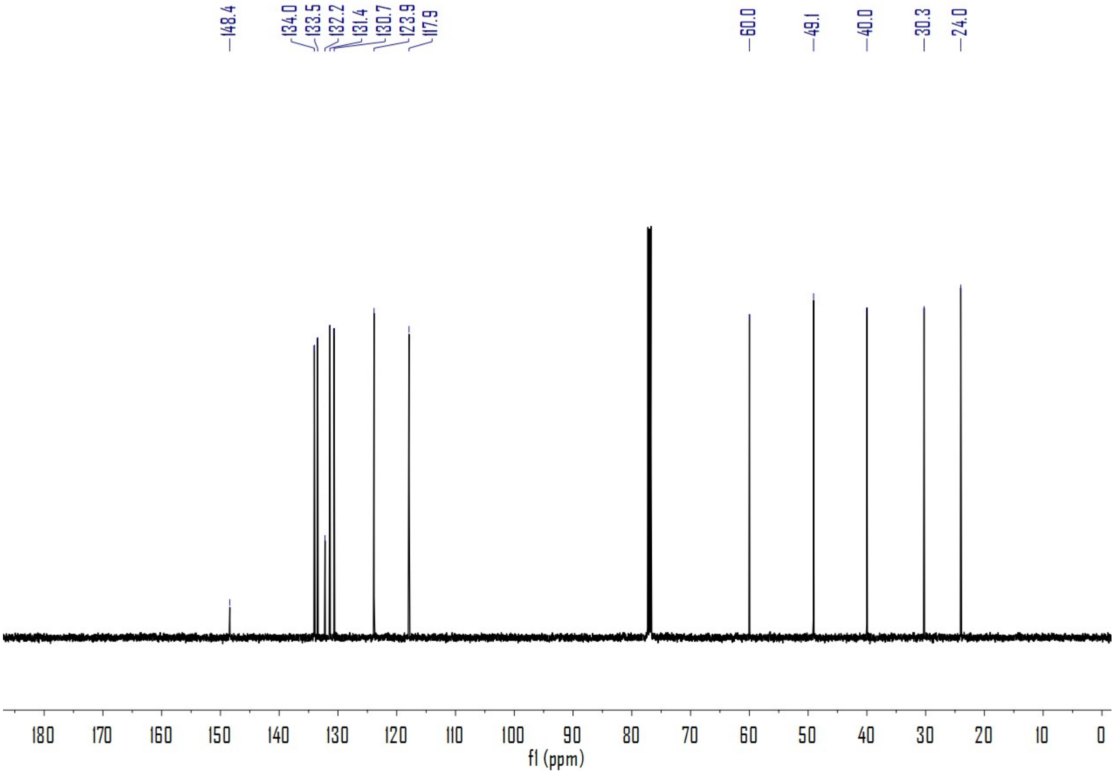

Supplementary Fig. 181. ^{13}C NMR spectrum of compound 3s.

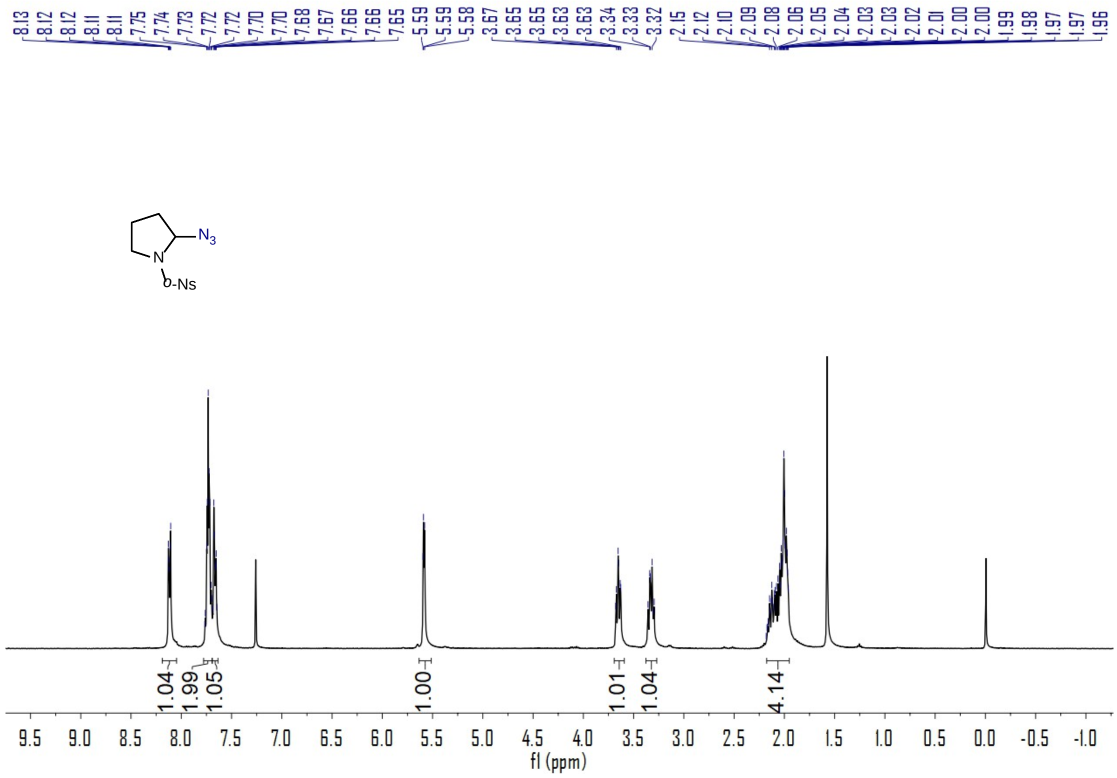

Supplementary Fig. 182. ^{1}**H NMR spectrum of compound 3t.**

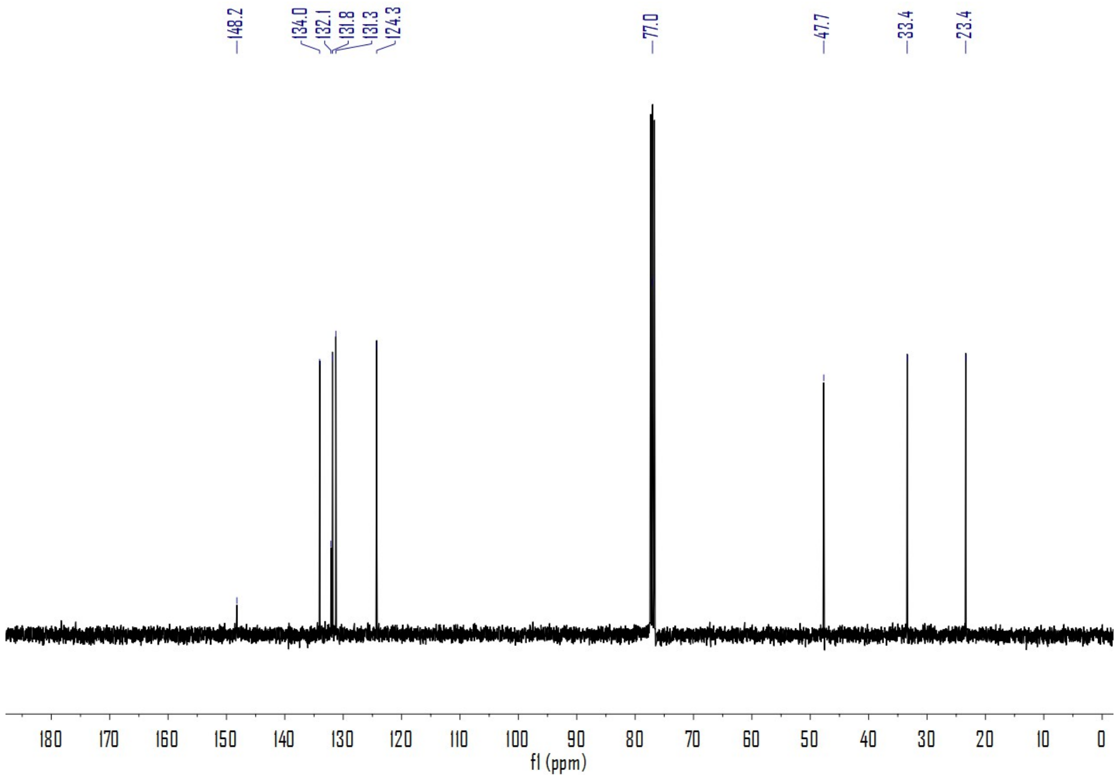

Supplementary Fig. 183. ^{13}**C NMR spectrum of compound 3t.**

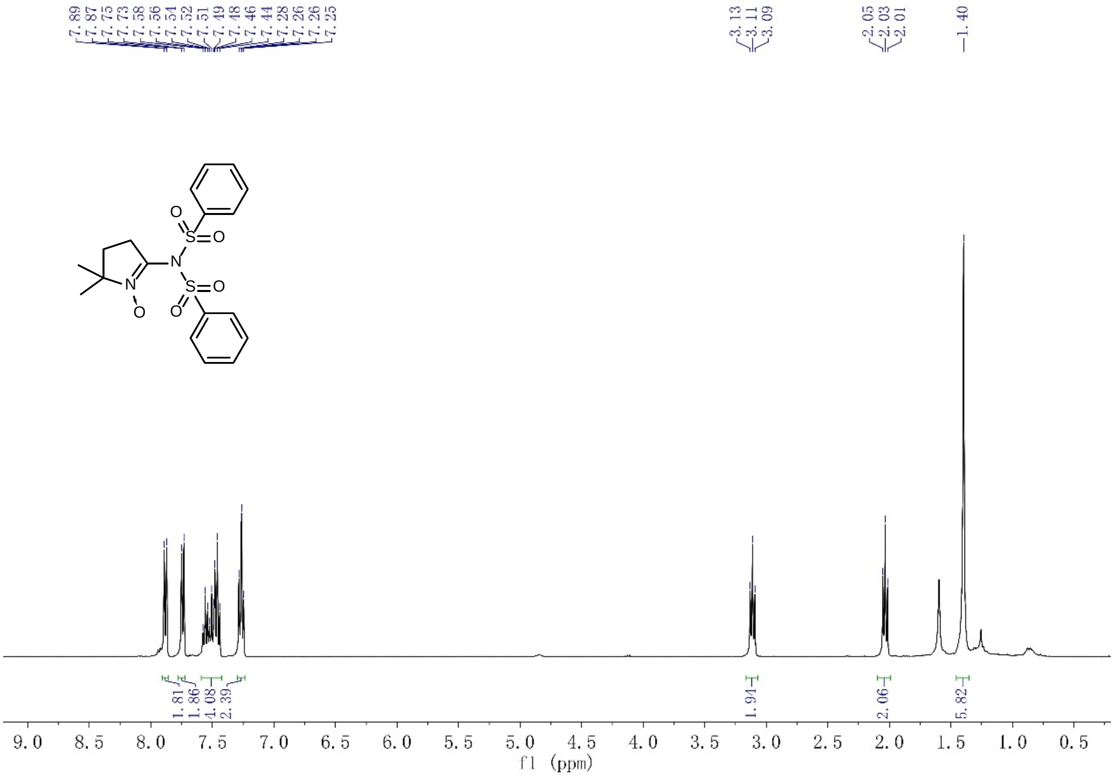

Supplementary Fig. 184. ^{1}H NMR spectrum of compound 4.

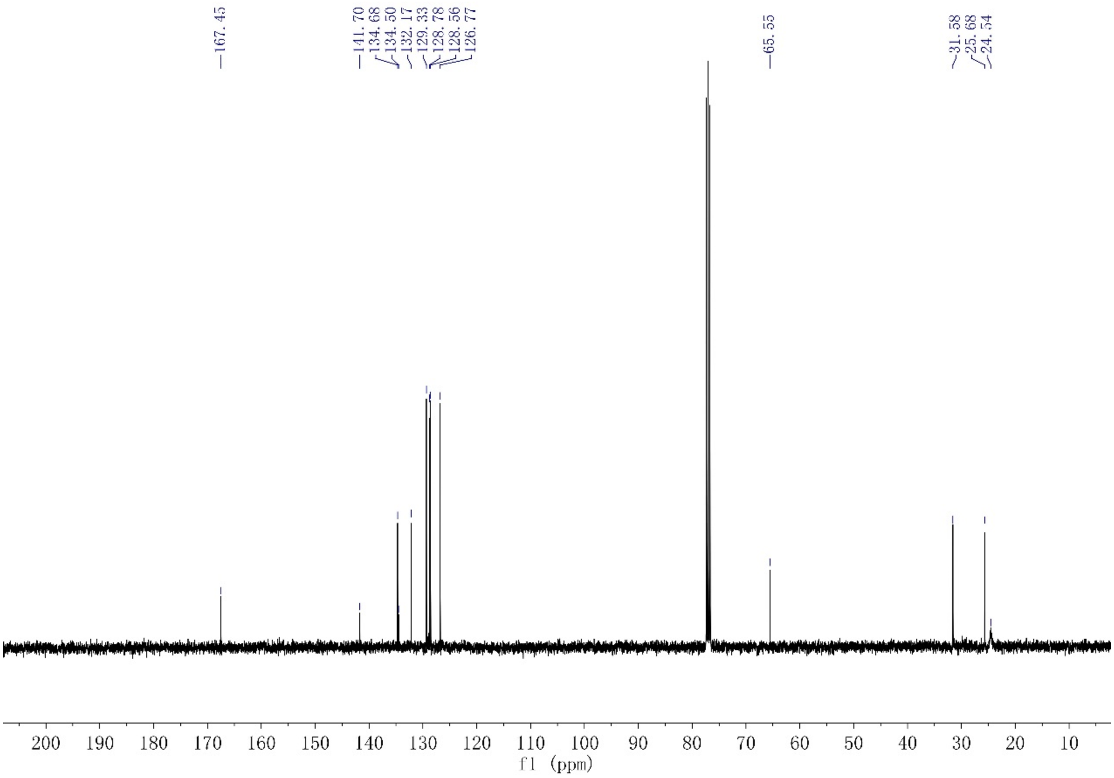

Supplementary Fig. 185. ^{13}C NMR spectrum of compound 4.

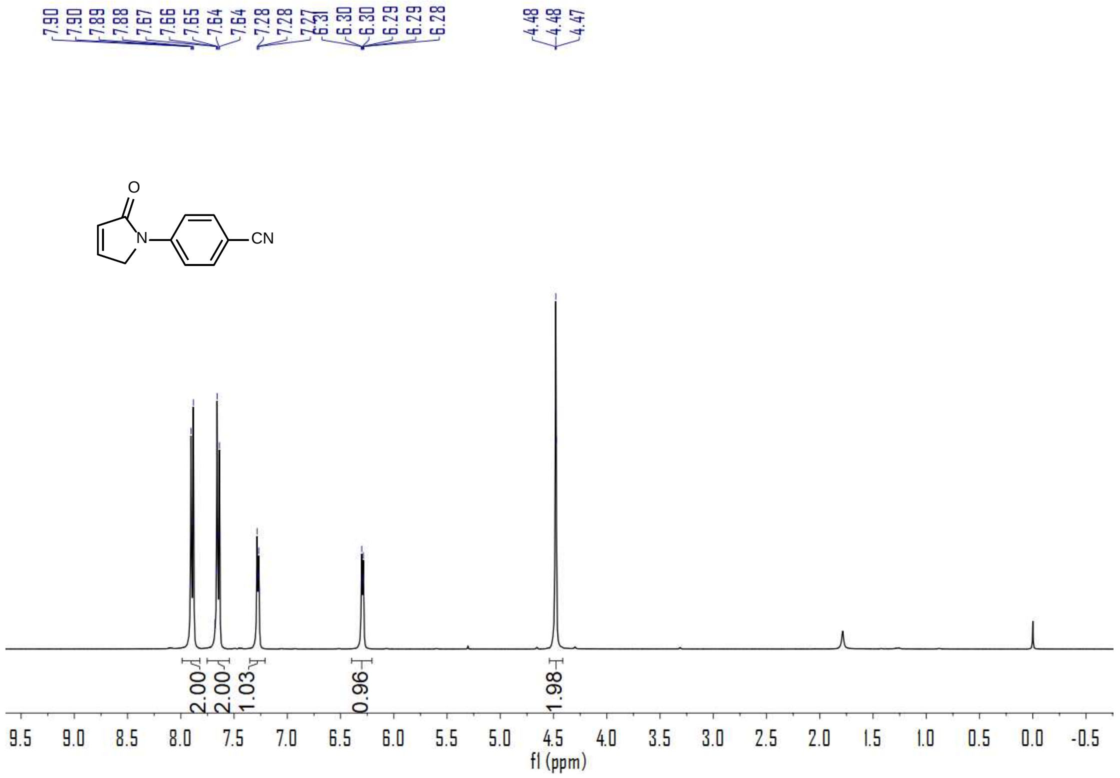

Supplementary Fig. 186. ^{1}H NMR spectrum of compound S0.

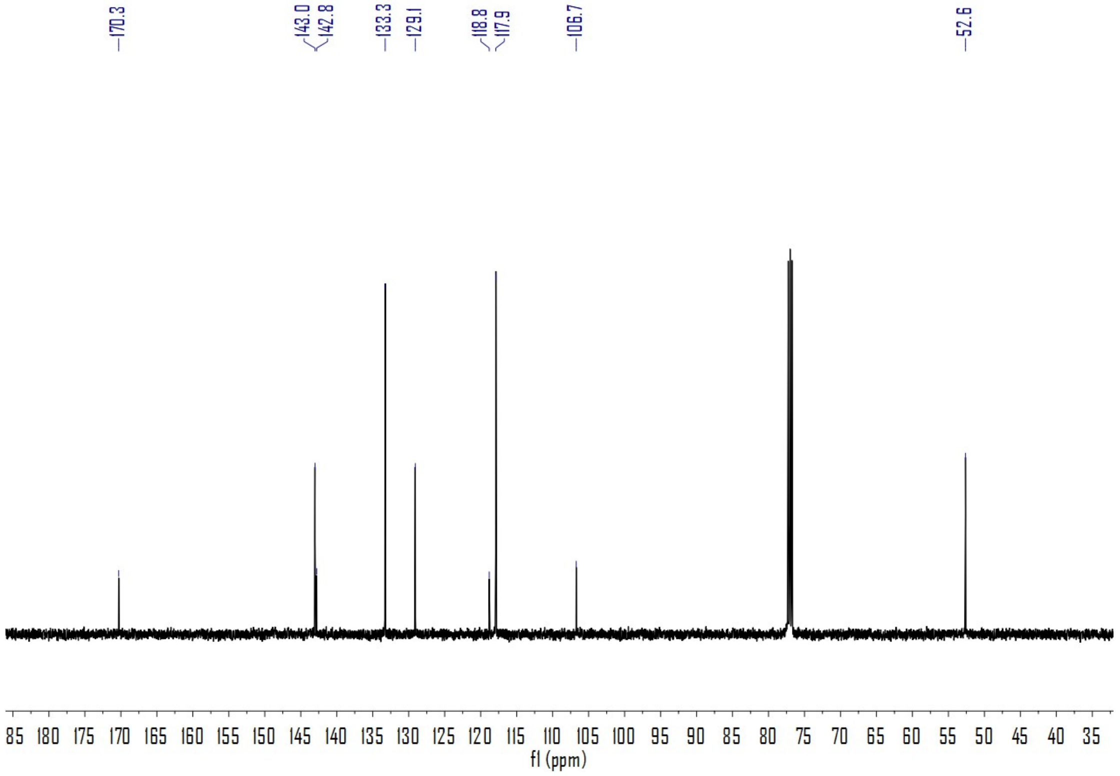

Supplementary Fig. 187. ^{13}C NMR spectrum of compound 4.

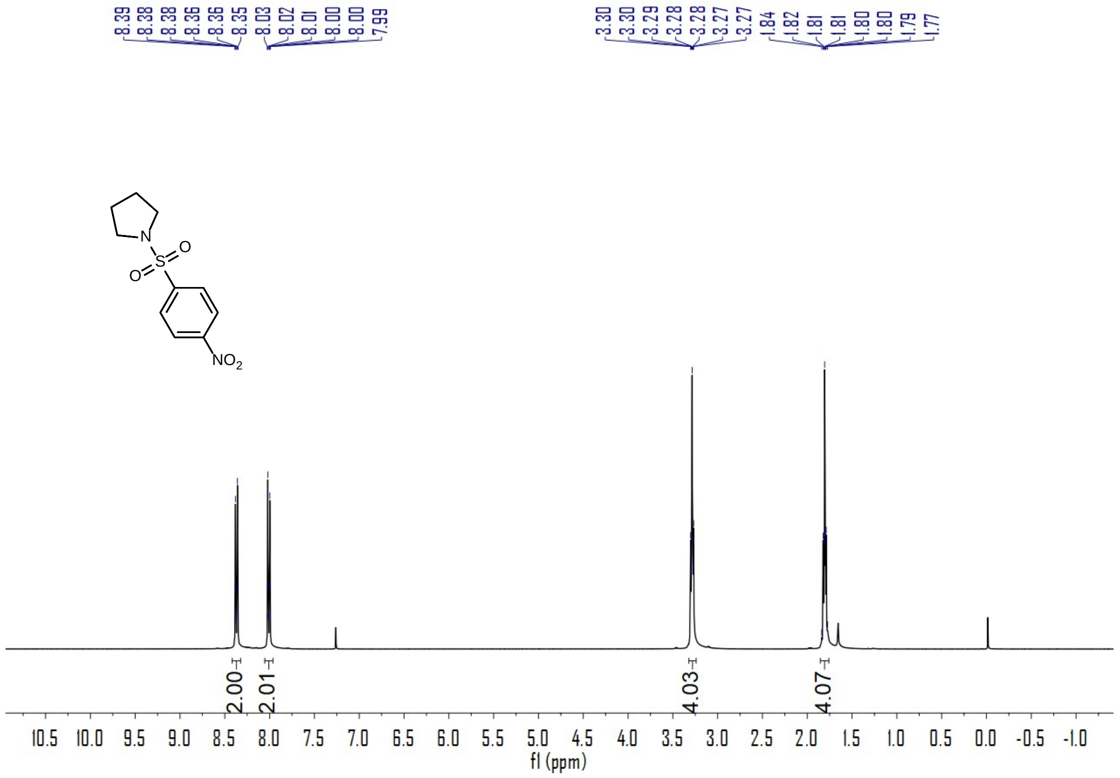

Supplementary Fig. 188. ^{1}H NMR spectrum of compound S1.

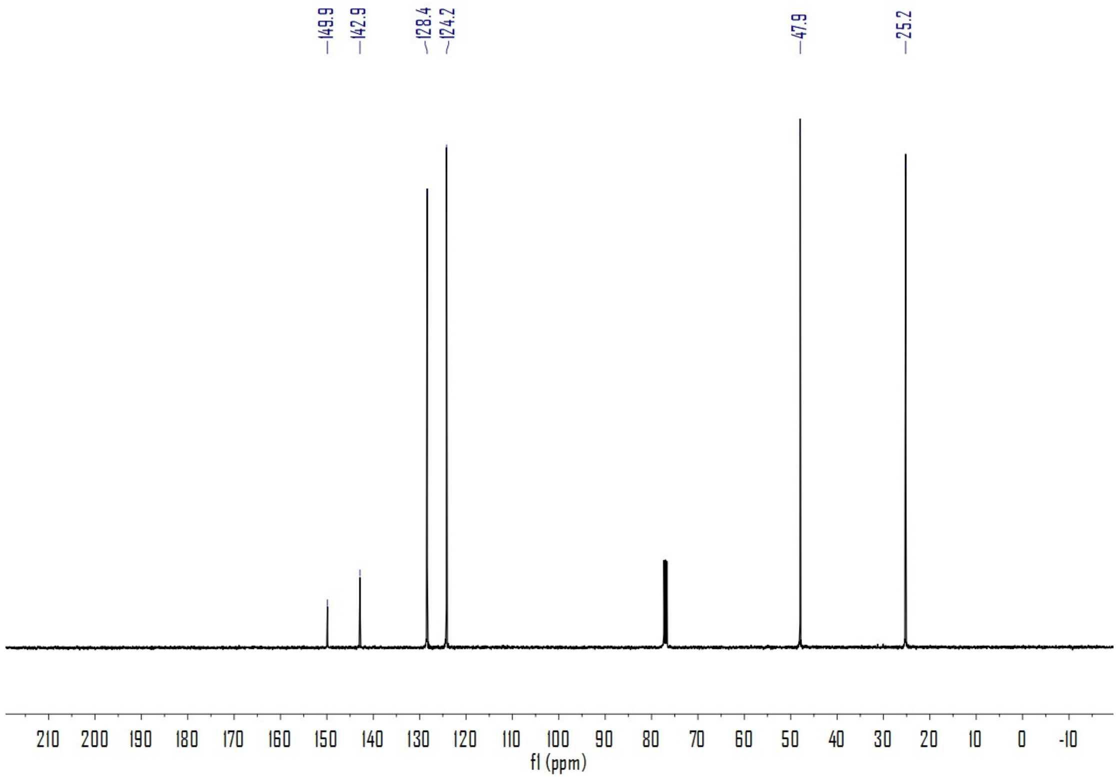

Supplementary Fig. 189. ^{13}C NMR spectrum of compound S1.

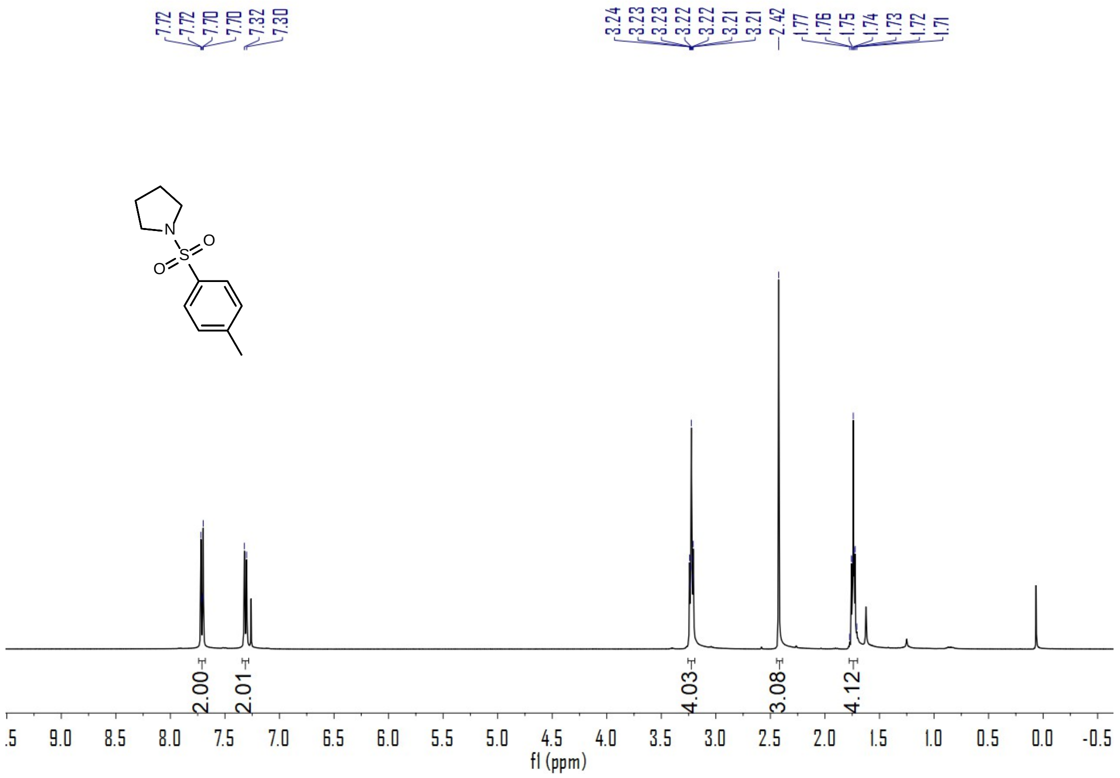

Supplementary Fig. 190. ^{1}**H NMR spectrum of compound S2.**

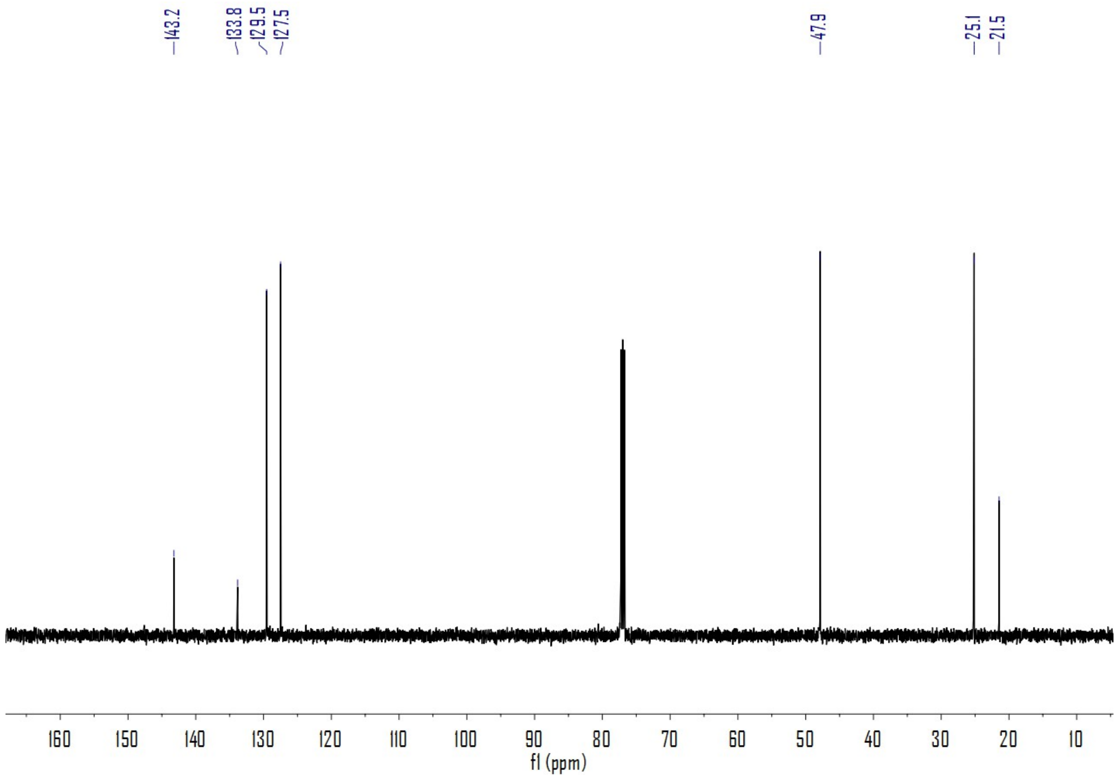

Supplementary Fig. 191. ^{13}**C NMR spectrum of compound S2.**

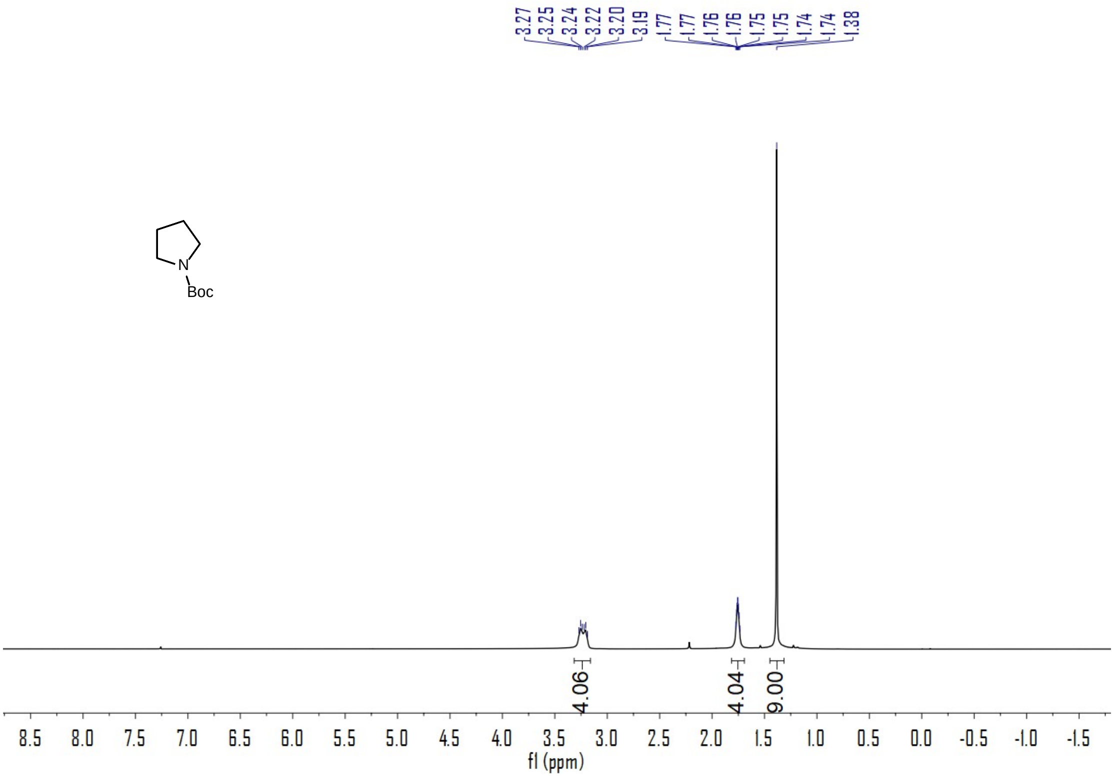

Supplementary Fig. 192. ^{1}H NMR spectrum of compound S3.

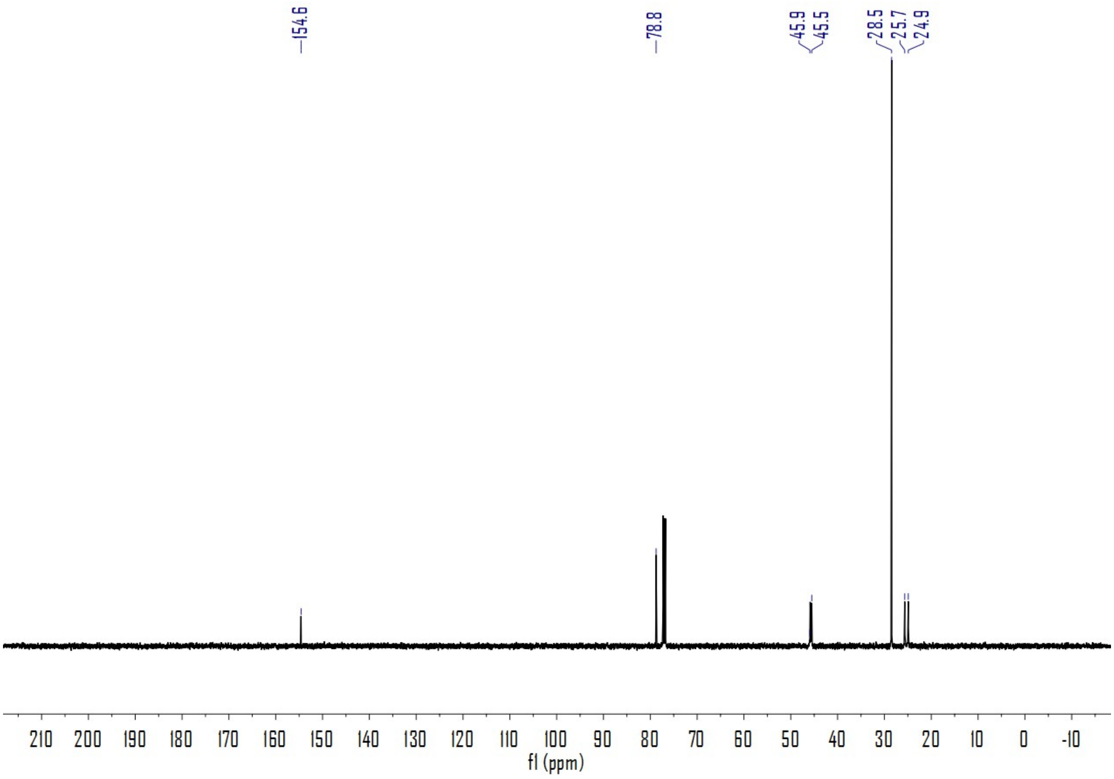

Supplementary Fig. 193. ^{13}C NMR spectrum of compound S3.

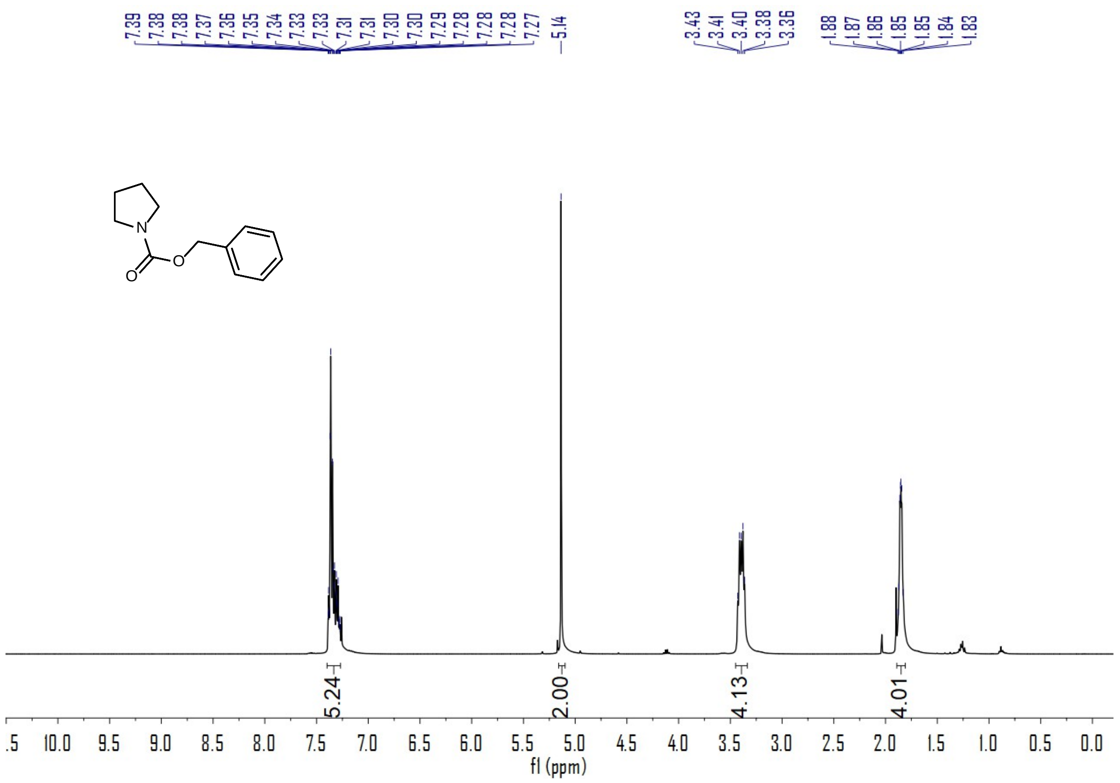

Supplementary Fig. 194. ^{1}H NMR spectrum of compound S4.

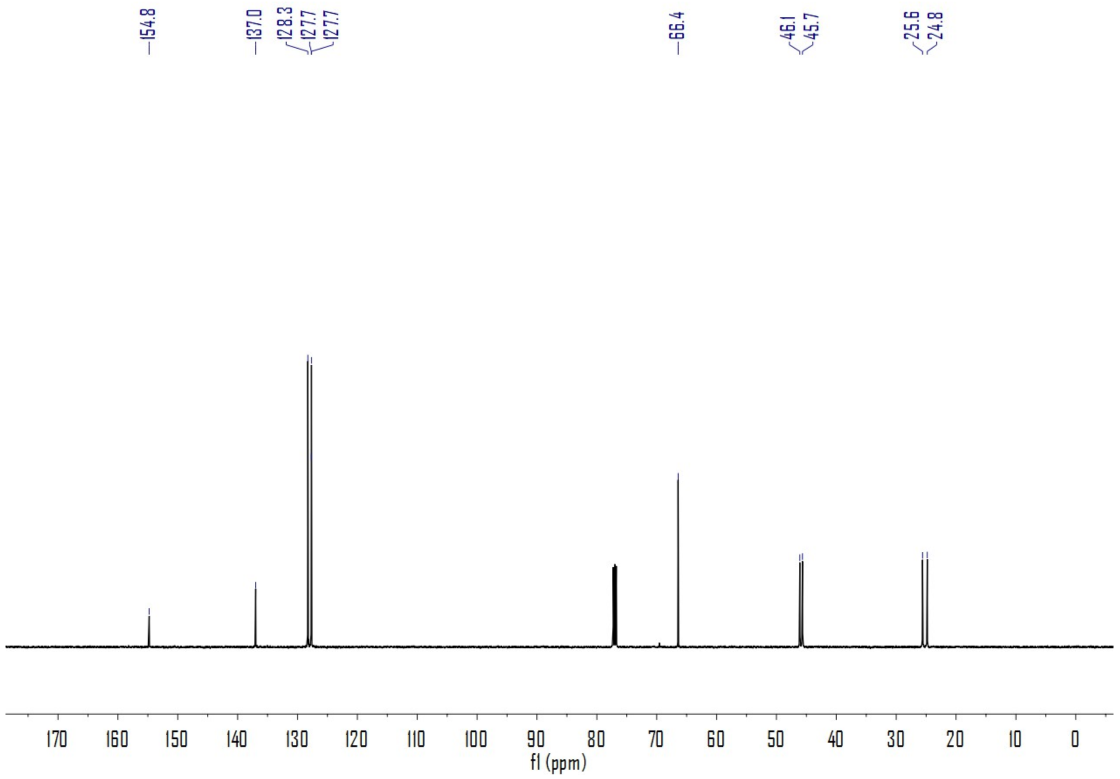

Supplementary Fig. 195. ^{13}C NMR spectrum of compound S4.

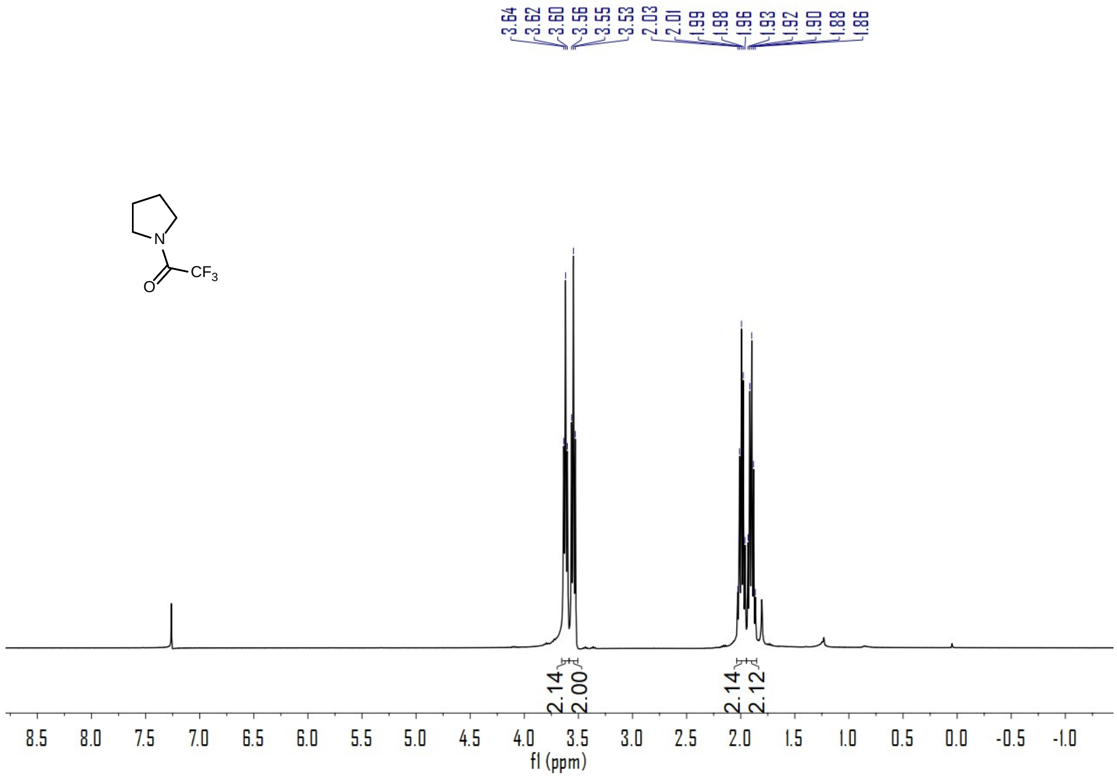

Supplementary Fig. 196. ^{1}**H NMR spectrum of compound S5.**

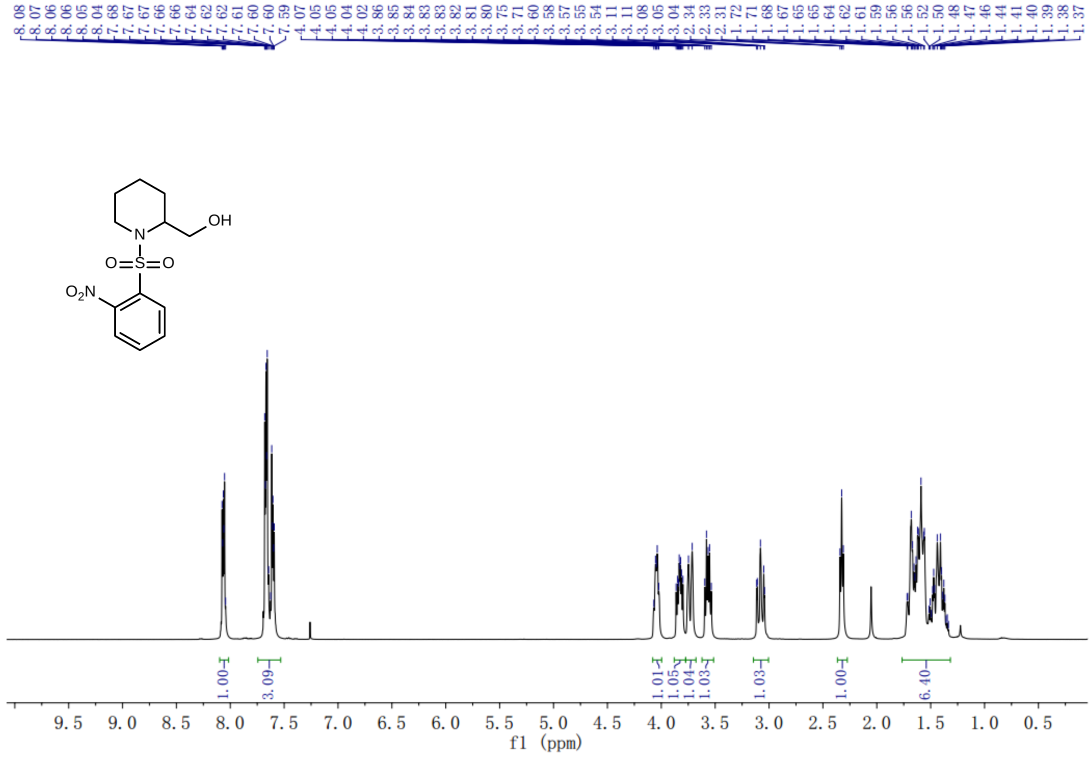

Supplementary Fig. 197. ^{1}H NMR spectrum of compound S6.

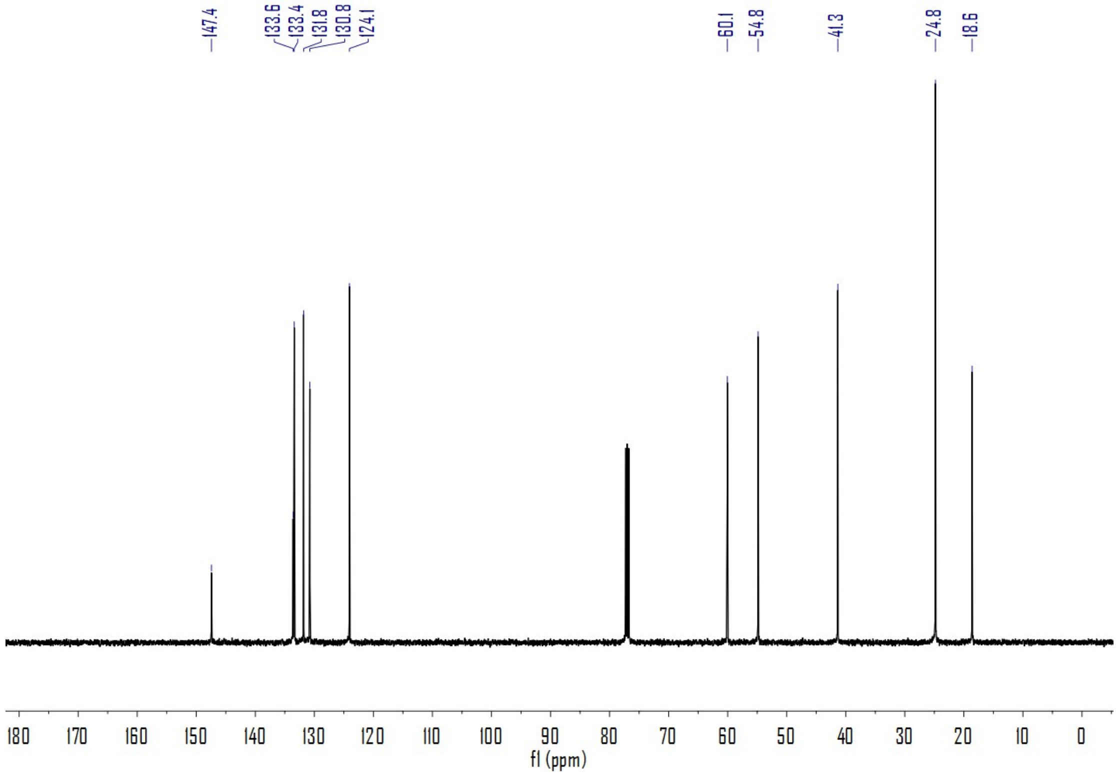

Supplementary Fig. 198. ^{13}C NMR spectrum of compound S6.

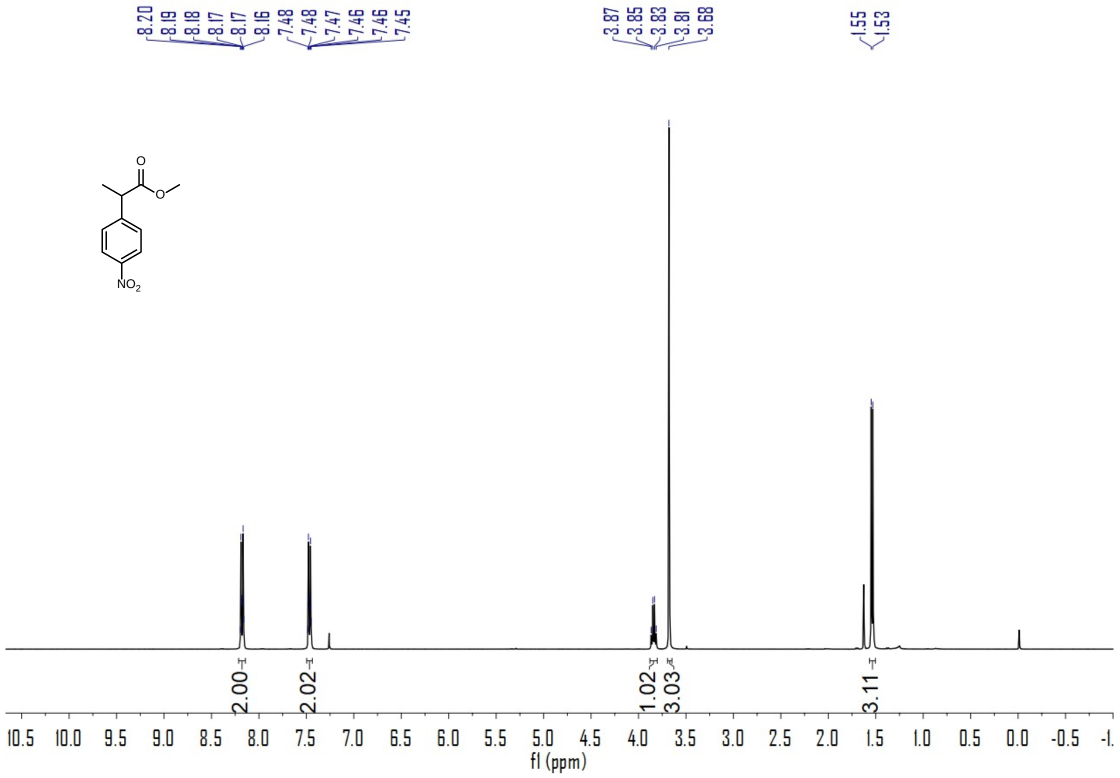

Supplementary Fig. 199. ^{1}**H NMR spectrum of compound S8.**

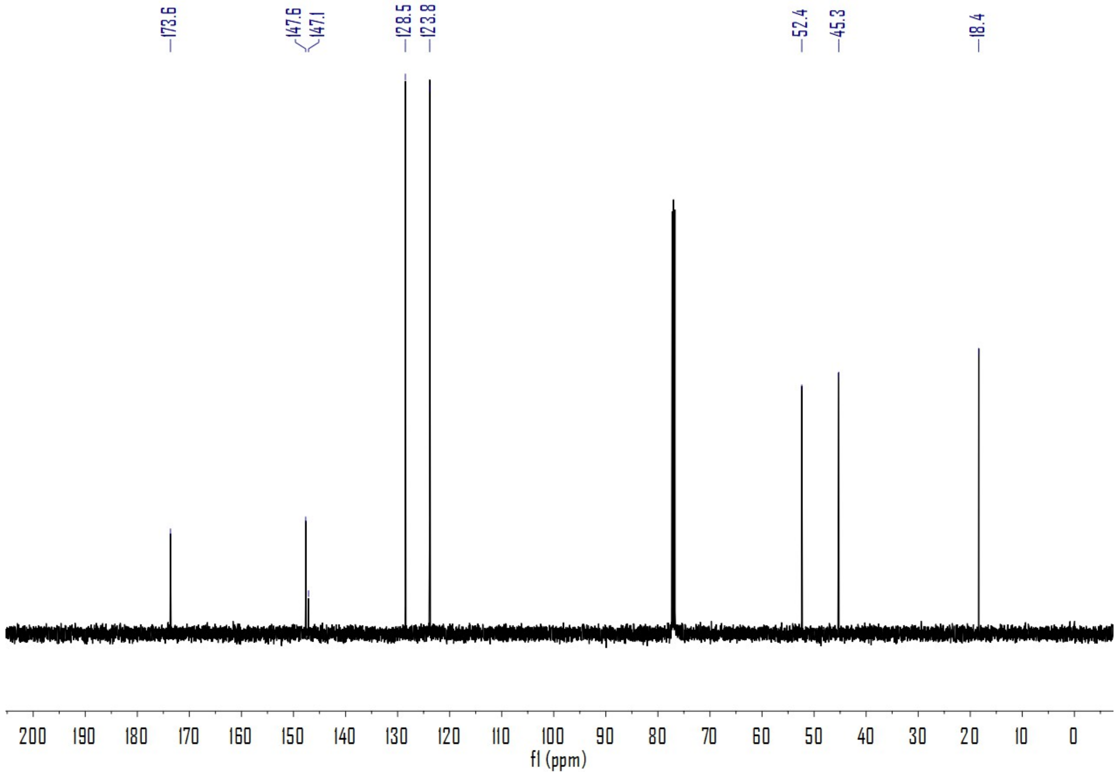

Supplementary Fig. 200. ^{13}**C NMR spectrum of compound S8.**

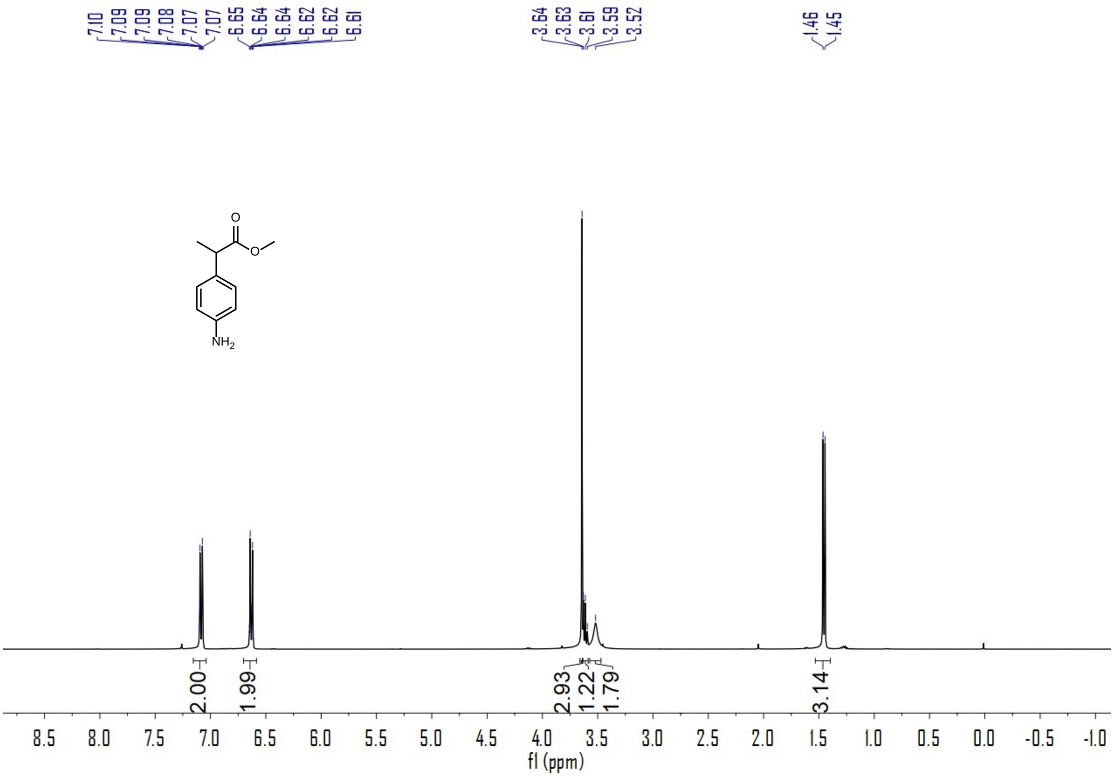

Supplementary Fig. 201. ^{1}**H NMR spectrum of compound S9.**

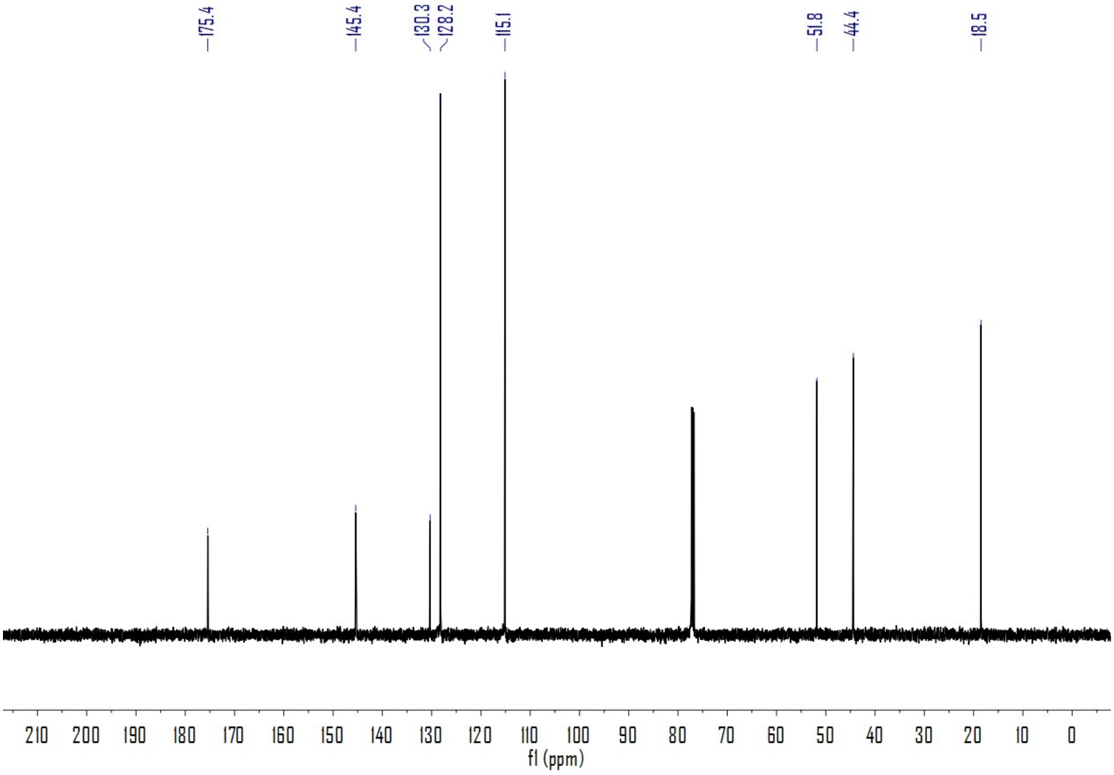

Supplementary Fig. 202. ^{13}**C NMR spectrum of compound S9.**

Supplementary References

1. Mallesham, B., Rajesh, B. M., Reddy, P. R., Srinivas, D. & Trehan, S. Highly Efficient CuI-catalyzed coupling of aryl bromides with oxazolidinones using Buchwald's protocol: A short route to linezolid and toloxatone. *Org. Lett.* **5**, 963-965 (2003).
2. Cook, G. R., Kargbo, R. & Maity, B. Catalytic enantioselective indium-mediated allylation of hydrazones. *Org. Lett.* **7**, 2767-2770 (2005).
3. Liu, L., Wang, Z., Zhang, H., Wang, W., Zhang, J. & Tang, Y. Iron-catalyzed aerobic C-H functionalization of pyrrolones. *Chem. Commun.* **51**, 9531-9534 (2015).
4. Feng, K., Quevedo, R. E., Kohrt, J. T., Oderinde, M. S., Reilly, U. & White, M. C. Late-stage oxidative C(sp^3)-H methylation. *Nature* **580**, 621-627 (2020).
5. Abdul-Rashed, S., Alachouzos, G., Brennessel, W. W. & Frontier, A. J. One-Pot double-annulation strategy for the synthesis of unusual fused bis-heterocycles. *Org. Lett.* **22**, 4350-4354 (2020).
6. Zhou, W., Zhang, Y.-X., Nie, X.-D., Si, C.-M., Sun, X. & Wei, B.-G. Approach to chiral 1-substituted isoquinolone and 3-substituted isoindolin-1-one by addition-cyclization process. *J. Org. Chem.* **83**, 9879-9889 (2018).
7. Collado, I., Ezquerra, J. & Pedregal, C. Stereoselective addition of Grignard-derived organocopper reagents to N-acyliminium ions: Synthesis of enantiopure 5- and 4,5-substituted prolinates. *J. Org. Chem.* **60**, 5011-5015 (1995).
